# Clickable azide-functionalized bromoarylaldehydes – synthesis and photophysical characterization

**DOI:** 10.3762/bjoc.16.139

**Published:** 2020-07-14

**Authors:** Dominik Göbel, Marius Friedrich, Enno Lork, Boris J Nachtsheim

**Affiliations:** 1 Institute for Organic and Analytical Chemistry, University of Bremen, Leobener Straße 7, 28359 Bremen, Germany; 2 Department of Organic Chemistry, Technical University Kaiserslautern, Erwin-Schrödinger-Straße Geb.54, 67663 Kaiserslautern, Germany; 3 Institute for Inorganic and Crystallographic Chemistry, University of Bremen, Leobener Straße NW2, 28359 Bremen, Germany

**Keywords:** bromoarylaldehydes, click-chemistry, fluorenes, fluorescence, phosphorescence

## Abstract

Herein, we present a facile synthesis of three azide-functionalized fluorophores and their covalent attachment as triazoles in Huisgen-type cycloadditions with model alkynes. Besides two *ortho*- and *para*-bromo-substituted benzaldehydes, the azide functionalization of a fluorene-based structure will be presented. The copper(I)-catalyzed azide–alkyne cycloaddition (CuAAC) of the so-synthesized azide-functionalized bromocarbaldehydes with terminal alkynes, exhibiting different degrees of steric demand, was performed in high efficiency. Finally, we investigated the photophysical properties of the azide-functionalized arenes and their covalently linked triazole derivatives to gain deeper insight towards the effect of these covalent linkers on the emission behavior.

## Introduction

**Scheme 1:**
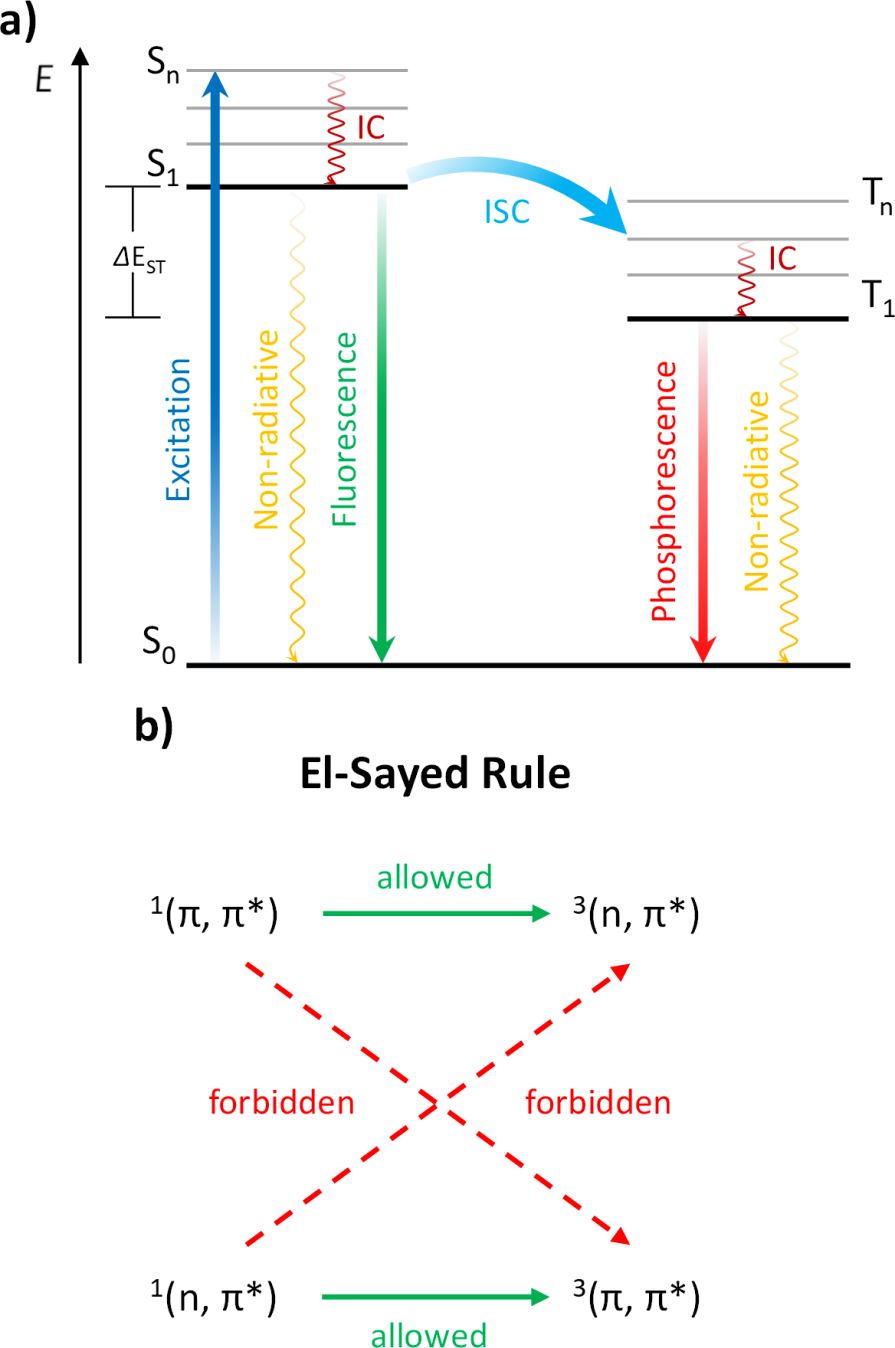
a) Schematic depiction of the Jablonski diagram. b) Schematic representation of El-Sayed’s rule.

Small organic luminophores exhibiting room-temperature phosphorescence (RTP) have attracted great attention due to promising applications in optoelectronic devices [1–8], biological imaging [9–12] and chemical sensing [13–15]. Referring to the Jablonski diagram (see Scheme 1a) [16–17], upon excitation from the singlet ground state (S_0_) to higher singlet states (S_n_), followed by internal conversion (IC), either non-radiative or radiative decay to S_0_ can occur. While the latter decay (fluorescence) takes place without a change in the electron spin, phosphorescence is defined as the radiative transition from the lowest excited triplet state (T_1_) to the singlet ground state (S_0_) [18–21]. Triplet state excitons are generated by the spin-forbidden intersystem crossing (ISC) process from the first excited singlet state (S_1_). Pursuant to the El-Sayed rule (see Scheme 1b) [22–23], ISC is spin allowed from ^1^(n,π*) to ^3^(π,π*) and from ^1^(π,π*) to ^3^(n,π*) excited states, while ISC is spin-forbidden from ^1^(n,π*) to ^3^(*n*,π*) and from ^1^(π,π*) to ^3^(π,π*) excited states, owing to the poor orbital overlap, resulting in a decreased spin-orbit coupling.

Quenching processes of triplet states, induced by molecular motions, oxygen, or humidity, restrict the versatile application of such organic materials [24]. Thus, low temperatures [25–27] or inert conditions [28] are necessary to facilitate an afterglow emission.

Most phosphorescence studies are focused on metal complexes due to a strong heavy atom-induced spin-orbit coupling [29–33]. Considering the high price and the toxicity of many metal complexes, pure organic phosphors are highly desirable [16–17,34–37]. Two approaches are applied to achieve organic phosphors: (1) introduction of nonmetal heavy atoms, such as halogens (Br or I) or functionalities containing lone pairs, in particular carbonyl groups, nitrogen, sulfur, and phosphorus derivatives which facilitate the ISC process from S_1_ to T_n_ and thus increase the spin-orbital coupling [38–47]. Also, decreasing the singlet–triplet splitting energy (Δ*E*_ST_) caused by intramolecular charge-transfer (ICT) interactions is an approved method [48–49]. (2) Significant reduction of non-radiative transitions can be achieved by the host–guest method [50–52] or by crystallization [53–57]. In difference to the liquid phase, the highly ordered packing and the restriction of molecular motions in the crystalline state favor a persistent luminescence.

**Figure 1:**
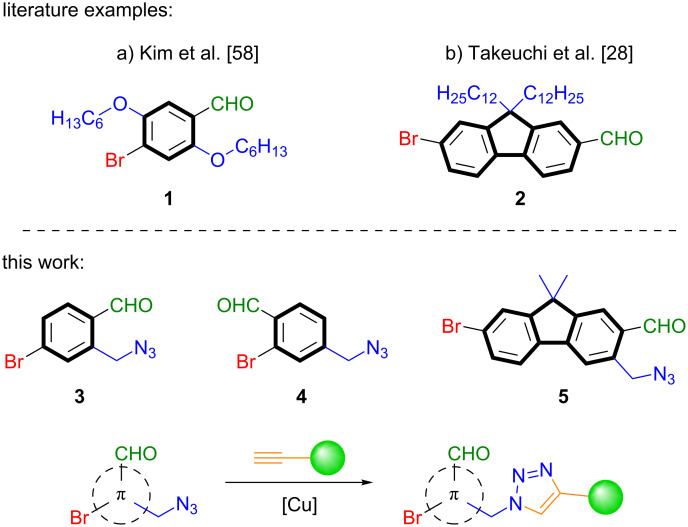
Top: literature examples of organic compounds showing RTP in the crystalline state (a) and in solution (b). Bottom: azide-linked derivatives presented in this work.

The promotion of ISC processes through intermolecular halogen bonding to generate efficient RTP was initially investigated by Kim et al. [58]. They developed the minimalistic 2,5-dihexyloxy-4-bromobenzaldehyde (**1**) [59–63] which showed a weak fluorescence in solution, but exhibited a green phosphorescence with a quantum yield of Φ_P_ = 2.9% in the crystalline state: this behavior was caused by intermolecular halogen bonds from the carbonyl-oxygen atom to an adjacent bromine atom (Figure 1a).

Despite multifarious examples of RTP in the crystalline state, purely organic compounds showing RTP in solution are rare [64–69]. Takeuchi et al. [28] reported a bromofluorenecarbaldehyde **2** which shows blue fluorescence in chloroform at 298 K under air and green phosphorescence under argon with a phosphorescence quantum yield of Φ_P_ = 5.9% (Figure 1b). This observation is reasoned by a strong (π,π*) character of the T_2_ state.

Although phosphorescent organic compounds are well investigated with respect to their photophysical properties in the crystalline state, in solution or physically embedded in polymer matrices, there is a significant lack of possibilities [61] for their targeted covalent attachment to higher structures. This is due to missing synthetic strategies to incorporate suitable linkable functionalities into those luminophores.

Our group is highly interested in the de novo synthesis of small organic luminophores and in this regard, we recently developed efficient methods for the synthesis of ESIPT-based luminophores [70–72]. Herein, we present the efficient functionalization of derivatives of the potent luminophores **1** and **2** with “clickable” azide functionalities to target the structures **3–5** and further investigated the influence of this functionalization, both in the unlinked azide state and the linked triazole state, on the emission properties of these compounds (Figure 1).

## Results and Discussion

### Syntheses *para*- and *ortho*-bromobenzaldehyde **3** and **4**

**Scheme 2:**
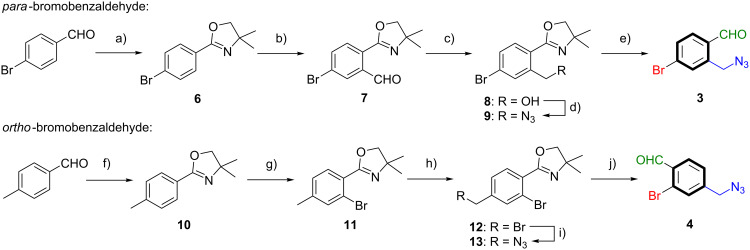
Reaction conditions for *para*-bromobenzaldehyde **3**: a) 1) 2-amino-2-methylpropan-1-ol, 4 Å MS, CH_2_Cl_2_, 25 °C, 18 h; 2) NBS, CH_2_Cl_2_, 25 °C, 5 h, 91%; b) 1) TMPMgCl·LiCl, THF, 25 °C, 4 h; 2) DMF, THF, 0 °C to 25 °C, 1.5 h; c) NaBH_4_, THF/MeOH 1:1 v/v, 0 °C, 1 h, 81% (2 steps); d) DPPA, DBU, PhMe, 25 °C, 18 h, 98%; e) 1) MeOTf, CH_2_Cl_2_, 25 °C, 2.5 h; 2) NaBH_4_, THF/MeOH 4:1 v/v, 0 °C, 2.5 h; 3) oxalic acid, THF/H_2_O 4:1 v/v, 25 °C, 20 h, 78%. Overall yield from 4-bromobenzaldehyde to **3**: 56% (5 steps). Reaction conditions for *ortho*-bromobenzaldehyde **4**: f) 1) 2-amino-2-methylpropan-1-ol, 4 Å MS, CH_2_Cl_2_, 25 °C, 18 h; 2) NBS, CH_2_Cl_2_, 25 °C, 4 h, 98%; g) 1) TMPMgCl·LiCl, THF, 25 °C, 4 h; 2) (CBrCl_2_)_2_, THF, 0 °C to 25 °C, 10 h, 76%; h) NBS, AIBN, CCl_4_, 100 °C, 7 h, 66%; i) NaN_3_, DMF, 25 °C, 4 h, 99%; j) 1) MeOTf, CH_2_Cl_2_, 25 °C, 2.5 h; 2) NaBH_4_, THF/MeOH 4:1 v/v, 0 °C, 2.5 h; 3) oxalic acid, THF/H_2_O 4:1 v/v, 25 °C, 20 h, 85%. Overall yield from 4-methylbenzaldehyde to **4**: 41% (5 steps).

We initiated our synthetic investigations towards azide-functionalized *para*-bromobenzaldehyde **3** with a two-step sequence. Condensation with 2-amino-2-methylpropan-1-ol and oxidation with NBS yielded oxazoline **6** in a good yield. Directed *ortho*-metalation utilizing TMPMgCl·LiCl under mild conditions and subsequent smooth formylation with DMF afforded benzaldehyde **7** (see Scheme 2). Due to rapid decomposition of **7** under ambient and acidic conditions, rapid aqueous work-up was conducted, followed by reduction with NaBH_4_, yielding the corresponding primary alcohol **8** in 81% yield over two steps. The transformation to azide **9** was accomplished by deprotonation using 1,8-diazabicyclo[5.4.0]undec-7-ene (DBU) and reaction with diphenylphosphoryl azide (DPPA) in excellent yield. Finally, the oxazoline group, which acted as directing and protecting group, was removed in a three-step sequence of *N*-methylation, reduction of the in situ formed iminium ion and acidic hydrolysis. This afforded the azide-functionalized *para*-bromobenzaldehyde **3** in 78% yield and an overall yield of 56% (starting from 4-bromobenzaldehyde).

Azide-functionalized *ortho*-bromobenzaldehyde **4** was prepared by a similar route as aldehyde **3**. Initially, oxazoline formation from 4-methylbenzaldehyde yielded 2-aryloxazoline **10** in almost quantitative yield. The introduction of the *ortho-*bromine substituent was again accomplished by metalation using TMPMgCl·LiCl and subsequent reaction with 1,2-dibromotetrachloroethane to afford **11** in 76% yield. A second bromination at the benzylic position provided the dibrominated derivative **12** in 66% yield. The substitution reaction of benzyl bromide with sodium azide delivered the primary azide **13** in quantitative yield. Again, the final back-conversion of the oxazoline group to the corresponding aldehyde afforded azide-functionalized *ortho*-bromobenzaldehyde **4** in 85% yield and an overall yield of 41% (starting from 4-methylbenzaldehyde).

### Bromofluorenecarbaldehyde **5**

**Scheme 3:**
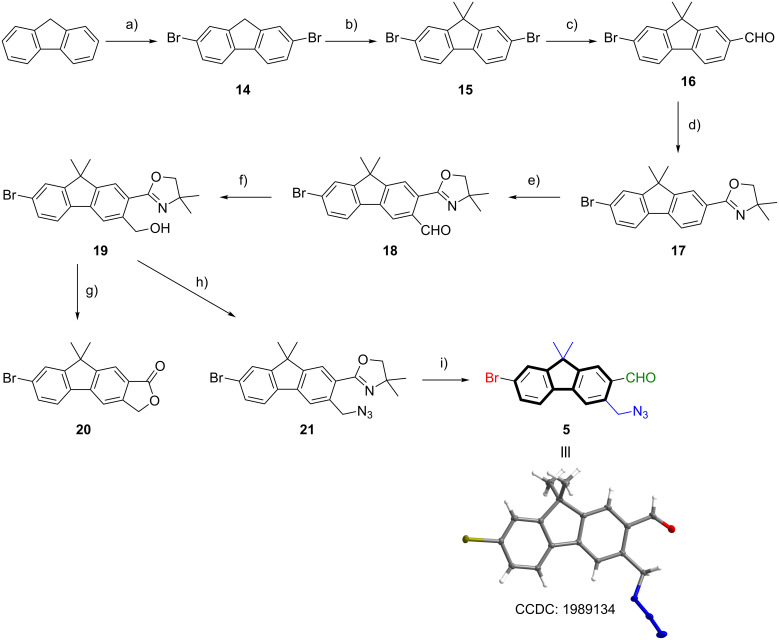
Reaction conditions: a) Br_2_, Fe powder, CHCl_3_, 0 °C, 4 h, 99%; b) KOH, KI, MeI, DMSO, 25 °C, 18 h, 92%; c) 1) *n-*BuLi, THF, ‒78 °C, 1 h; 2) DMF, THF, −78 °C to 25 °C, 10 h, 90%; d) 1) 2-amino-2-methylpropan-1-ol, 4 Å MS, CH_2_Cl_2_, 25 °C, 18 h; 2) NBS, CH_2_Cl_2_, 25 °C, 3 h, 92%; e) 1) TMPMgCl·LiCl, THF, 25 °C, 4 h; 2) DMF, THF, ‒15 °C to 25 °C, 2 h; f) NaBH_4_, THF/MeOH 1:1 v/v, 0 °C, 1.5 h, 79% (2 steps); g) HCl (4 N), 120 °C, 6 h, 91%; h) 1) DPPA, DBU, PhMe, 25 °C, 15 h; 2) NaN_3_, PhMe, 60 °C, 4 h, 87%; i) 1) MeOTf, CH_2_Cl_2_, 25 °C, 2.5 h; 2) NaBH_4_, THF/MeOH 4:1 v/v, 0 °C, 2.5 h; 3) oxalic acid, THF/H_2_O 4:1 v/v, 25 °C, 20 h, 86%. Overall yield to **5**: 45% (8 steps). The molecular structure of **5** shows 50% probability ellipsoids.

The synthetic route to azide-functionalized 7-bromofluorene-2-carbaldehyde **5** started from unfunctionalized fluorene. Double bromination to **14**, followed by double methylation of the methylene bridge to **15** and a lithiation/formylation sequence afforded 7-bromofluorene-2-carbaldehyde **16** in excellent yield over three steps. Conversion to the 2-aryloxazoline **17** was accomplished in 92% yield using the same method as described for the synthesis of **6** and **10**. *ortho*-Metalation with TMPMgCl·LiCl [70] and conversion of the magnesiated species with DMF to carbaldehyde **18** was followed by reduction with NaBH_4_ to give the primary alcohol **19**. In contrast to benzaldehyde **7**, carbaldehyde **18** showed no decomposition at ambient temperature. While acidic hydrolysis of **19** provided exocyclic γ-lactone **20**, the substitution reaction with DPPA/NaN_3_ yielded the primary azide in 87% yield. In accordance to previous deprotection reactions, fluorene **21** was converted by means of a three-step sequence to the desired azide-functionalized 7-bromofluorene-2-carbaldehyde **5** in 86% yield and an overall yield of 45% (starting from fluorene). The molecular structure of **5** could be verified by X-ray diffraction (XRD, see Scheme 3).

### Derivatization of fluorenyl methanol **19**

**Scheme 4:**
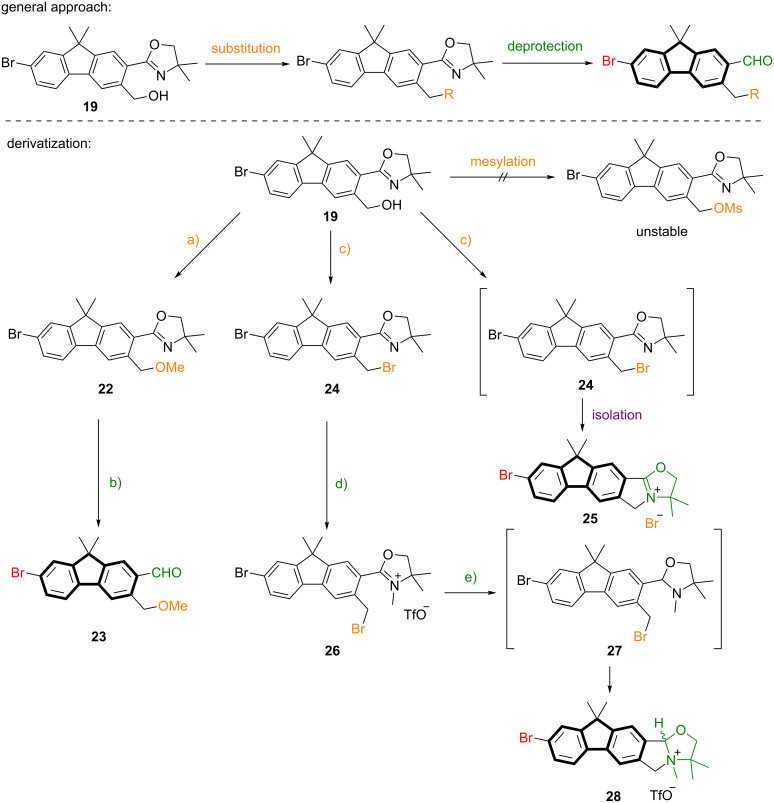
Reaction conditions: a) 1) NaH, THF, 0 °C, 30 min; 2) MeI, THF, 0 °C to 25 °C, 2 h, 99%; b) 1) MeOTf, CH_2_Cl_2_, 25 °C, 3 h; 2) NaBH_4_, THF/MeOH 4:1 v/v, 0 °C, 3 h; 3) oxalic acid, THF/H_2_O 4:1 v/v, 25 °C, 24 h, 75%. Overall yield from fluorene to **23**: 44% (8 steps). c) CBr_4_, PPh_3_, CH_2_Cl_2_, 0 °C to 25 °C, 2 h, 95%; or NBS, PPh_3_, CH_2_Cl_2_, 0 °C to 25 °C, 2 h, 91%; or PBr_3_, CH_2_Cl_2_, 0 °C to 25 °C, 2 h, 92%; d) MeOTf, CH_2_Cl_2_, 25 °C, 3 h; e) NaBH_4_, THF/MeOH 4:1 v/v, 0 °C, 3 h. Overall yield from **24** to **28**: 56% (2 steps).

To gain deeper insights into the emission behavior of fluorenes bearing different functional groups in the side chains, fluorenylmethanol **19** was subjected to derivatization reactions (see Scheme 4). Deprotonation and subsequent methylation afforded methoxy derivative **22**, which was then converted into the methoxy-functionalized 7-bromofluorene-2-carbaldehyde **23** in 75% yield. Implementation of potent leaving groups in the side chain as complement linkable functionalities via S_N_-reaction was initiated by mesylation. Unfortunately, the mesylated fluorene showed such a high reactivity that rapid decomposition occurred. However, bromination was conducted by various substitution methods delivering benzyl bromide **24**, which upon isolation cyclized to iminium bromide **25** in high yield. To suppress this unexpected cyclization, careful fine-adjustment of the work-up conditions were made. Here, upon complete formation of **24**, rapid filtration of the reaction mixture through a plug of neutral alumina, solvent evaporation and quick conversion in the next step was successfully applied. Deprotection to the carbaldehyde was performed using the well applied three-step methylation/reduction/hydrolysis sequence. Methylation of the oxazoline nitrogen afforded iminium salt **26**, which was reduced to oxazolidine **27**. Again, similar to **25**, cyclization took place and ammonium triflate **28** was isolated in 56% yield (starting from **24**). In contrast to the cyclization of oxazoline **24**, oxazolidine **27** cyclized already during the reaction, caused by the increased basicity of the ring nitrogen.

### CuAAC reactions of bromocarbaldehydes

**Scheme 5:**
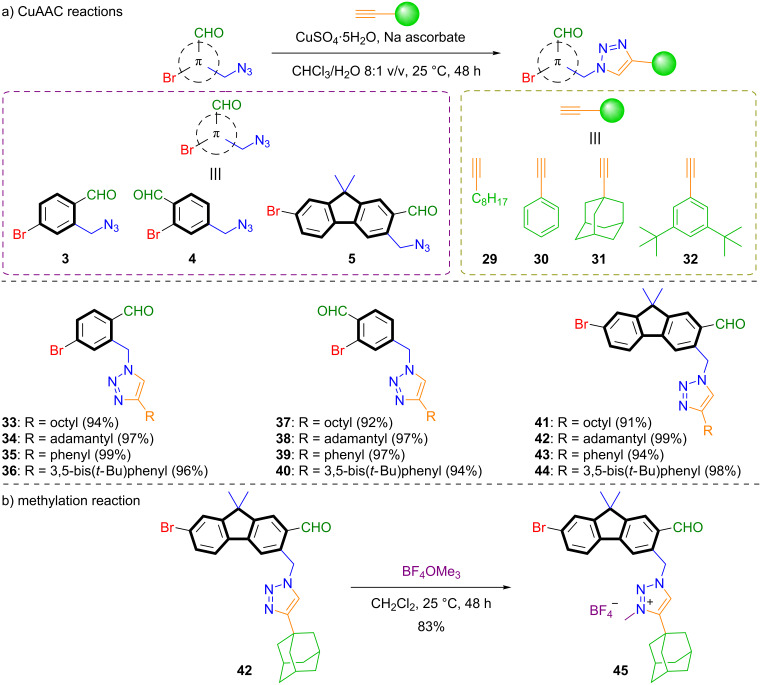
a) CuAAC reactions of azide-functionalized bromocarbaldehydes **3**, **4** and **5** with terminal alkynes to triazoles **33**–**44**. General reaction conditions for CuAAC reactions: Azide (1.00 equiv), terminal alkyne (1.05 equiv), CuSO_4_·5H_2_O (0.1 equiv), sodium ascorbate (0.50 equiv), CHCl_3_, (0.1 M) and water (12.5 mM) at 25 °C for 48 h. b) Methylation reaction of adamantly-substituted triazole **42** with Meerwein′s salt (trimethyloxonium tetrafluoroborate).

We further investigated the reactivity of azide-functionalized bromocarbaldehydes **3**, **4**, and **5** in copper(I)-catalyzed azide–alkyne cycloaddition reactions (CuAAC). For this, we treated the azide-functionalized luminophores with alkynes exhibiting different degrees of steric demand, including 1-decyne (**29**), phenylacetylene (**30**), 1-ethynyladamantane (**31**) and 1,3-di-*tert*-butyl-5-ethynylbenzene (**32**, see Scheme 5). All triazoles **33**–**44**, based on the bromocarbaldehydes **3**, **4**, and **5** were successfully isolated in excellent yields of >90%. As a further model functionalization, the sterically demanding adamantyl substituted triazole **42** was subjected to a methylation reaction with Meerwein′s salt (trimethyloxonium tetrafluoroborate) to deliver the *N*-methylated triazole **45** in 83% yield.

### Photophysical properties

**Figure 2:**
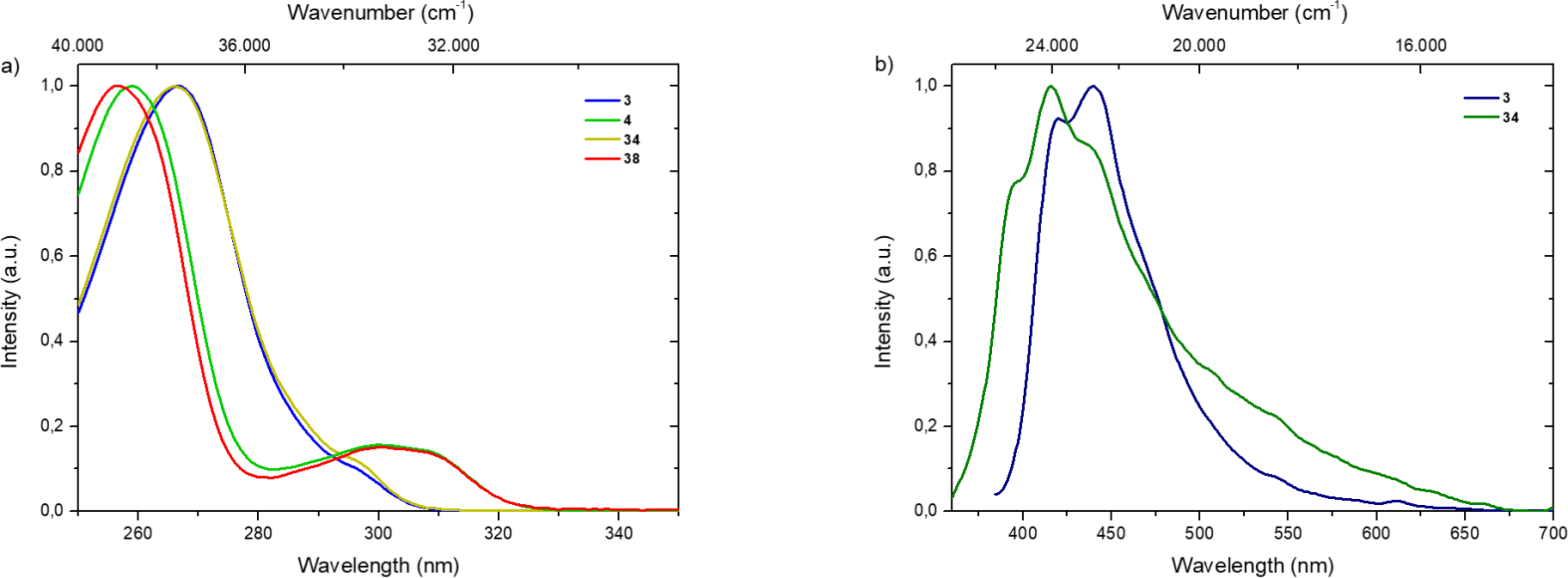
a) Normalized UV–vis absorption spectra of **3** (blue line), **34** (olive line), **4** (green line) and **38** (red line) in CHCl_3_ (*c* = 10^−5^ mol·L^−1^). b) Normalized emission spectra of **3** (navy line), **34** (green line) in the solid-state.

Finally, we examined the photophysical properties of both the azides and the triazoles. UV–vis absorption measurements of *para*-bromobenzaldehyde **3** and *ortho*-bromobenzaldehyde **4** as well as the corresponding triazoles **33**–**40** were conducted in chloroform. Intense absorption bands below 270 nm were observed, which could be attributed to typical π–π* transitions derived from the single-benzene core. In addition, unstructured absorption bands above 290 nm were observed, while *ortho*-derivatives exhibited more broadened bands than the *para*-derivatives (see Figure 2a and Figures S1–S11 in Supporting Information File 1). The emission spectra for *para*-bromocarbaldehyde **3** and adamantyltriazole **34** in the solid-state show maxima at 440 nm (for compound **3**) and 416 nm (for compound **34**) (see Figure 2b). Additionally, fluorescence lifetimes (τ) of **3** and **34** were determined by time correlated single photon counting (TCSPC), indicating low lifetimes of 2.21 ns (for compound **3**) and 3.23 ns (for compound **34**) for lower populated species (see Figure S6, Supporting Information File 1). Quantum yields (Φ) of both derivatives are below 1% in the solid-state. In chloroform, no luminescence was detected for the *para* derivatives. All *ortho* derivatives exhibited luminescence neither in solution nor in the solid-state [73].

These observations for the *para*- and *ortho*-bromobenzaldehydes indicate that the phosphorescence is quenched in these systems, yielding only a weak fluorescence. This may be owing to the azide moiety, either as a functional group or as part of the triazole heterocycle. Similar observations were already made for luminescent materials [74–75].

**Figure 3:**
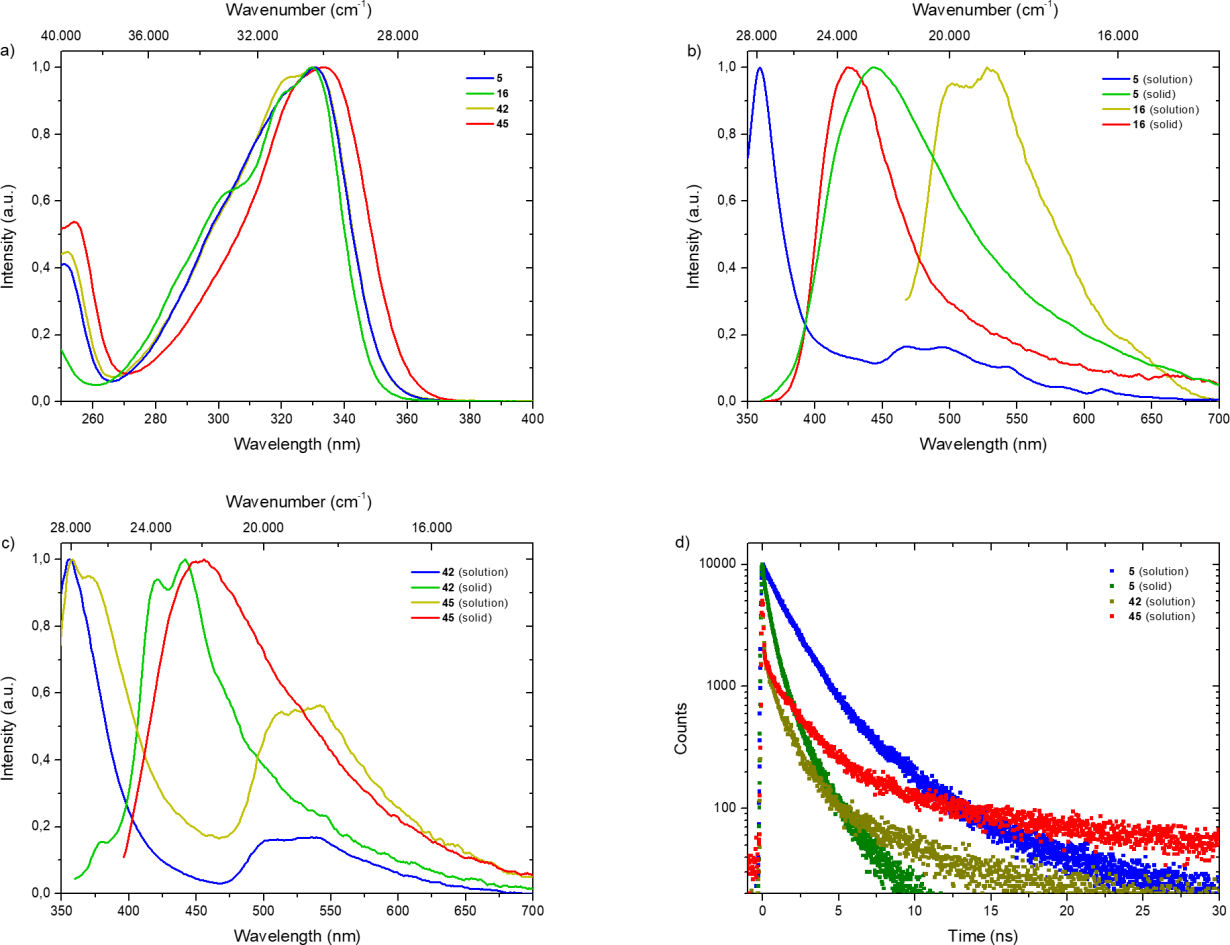
a) Normalized UV–vis absorption spectra of **5** (blue line), **16** (green line), **42** (olive line) and **45** (red line) in CHCl_3_. b) Normalized emission spectra of **5** (in solution, blue line and in the solid-state, green line) and **16** (in solution, olive line and in the solid-state, red line). c) Normalized emission spectra of **42** (in solution, blue line and in the solid-state, green line) and **45** (in solution, olive line and in the solid-state, red line). d) Time resolved emission decay curves of **5** (in solution, blue dots and in the solid-state, green dots), **42** (in solution, olive dots) and **45** (in solution, red dots) at 25 °C. In all diluted measurements (*c* = 10^−5^ mol·L^−1^) CHCl_3_ was used as solvent and solutions were sparged with argon for 30 minutes.

Fluorene derivatives were subjected to photophysical measurements as well. UV–vis absorption spectra of bromofluorenecarbaldehydes **5** and **16** show intense absorption bands at 331 and 330 nm. Furthermore, adamantyltriazole **42** and the corresponding tetrafluoroborate salt **45** exhibit similar absorption properties, while the methylated species **45** shows a comparable slightly red-shifted absorption band (see Figure 3a and Figures S12–S14 in Supporting Information File 1). Emission measurements of carbaldehydes **5** and **16** revealed that the solid-state emission bands are more blue-shifted than the emission bands in solution (see Figure 3b). Similar observations were made for adamantyltriazole **42** and tetrafluoroborate salt **45** with maxima at 510 nm and 540 nm (see Figure 3c). However, TCSPC demonstrated that solely fluorescence was observed in all physical states for all investigated compounds with a maximum lifetime τ of 11.6 ns for methylated triazole **45** in chloroform (see Figure 3d). Quantum yields Φ were determined to be <1% for all structures. Further fluorene derivatives – methoxymethyl carbaldehyde **23**, iminium bromide **25** and ammonium triflate **28** – exhibit intense absorption maxima around 320 nm (see Figure S13, Supporting Information File 1). For compounds **25** and **28**, the emission maxima in the solid-state were red-shifted compared to the emission bands in chloroform solution (see Figures S31 and S33, Supporting Information File 1). Aldehyde **23** exhibits a deep violet emission maximum at 360 nm in chloroform solution and no emission in the solid-state (see Figure S30, Supporting Information File 1). Lifetimes τ were defined up to 8.66 ns for iminium bromide **25**, with quantum yields Φ below 1% (see Figure S32, Supporting Information File 1). The absence of any long living triplet species in all fluorene derivatives in solution as well as in the solid-state again indicates undesirable quenching events, induced by the azide functionalities, similar to *para*- and *ortho*-bromobenzaldehydes.

## Conclusion

In summary, azide-functionalized *ortho*- and *para*-bromobenzaldehydes and fluorene derivatives were successfully synthesized. The azide bearing arenes were efficiently linked with even sterically highly demanding alkynes in CuAAC. Initial photophysical investigations of azide-functionalized bromocarbaldehydes and fluorenes revealed that the azide moiety and the triazole heterocycle efficiently quench phosphorescent transitions. Efforts of installing other functional groups suitable for covalent connections or modifications of the alkyl bridge between the arene and the azide are underway in our laboratories.

## Supporting Information

File 1 Detailed experimental procedures, characterization data, photophysical properties, and copies of NMR spectra.

## References

[1] Kabe R, Notsuka N, Yoshida K, Adachi C (2016). Adv Mater (Weinheim, Ger).

[2] Tao Y, Yang C, Qin J (2011). Chem Soc Rev.

[3] Goushi K, Yoshida K, Sato K, Adachi C (2012). Nat Photonics.

[4] Zhang Q, Li B, Huang S, Nomura H, Tanaka H, Adachi C (2014). Nat Photonics.

[5] Chaudhuri D, Wettach H, van Schooten K J, Liu S, Sigmund E, Höger S, Lupton J M (2010). Angew Chem, Int Ed.

[6] Chaudhuri D, Sigmund E, Meyer A, Röck L, Klemm P, Lautenschlager S, Schmid A, Yost S R, Van Voorhis T, Bange S (2013). Angew Chem, Int Ed.

[7] Murawski C, Leo K, Gather M C (2013). Adv Mater (Weinheim, Ger).

[8] Baldo M A, O'Brien D F, You Y, Shoustikov A, Sibley S, Thompson M E, Forrest S R (1998). Nature.

[9] Gao R, Mei X, Yan D, Liang R, Wei M (2018). Nat Commun.

[10] Miao Q, Xie C, Zhen X, Lyu Y, Duan H, Liu X, Jokerst J V, Pu K (2017). Nat Biotechnol.

[11] Zhen X, Tao Y, An Z, Chen P, Xu C, Chen R, Huang W, Pu K (2017). Adv Mater (Weinheim, Ger).

[12] Zhang G, Palmer G M, Dewhirst M W, Fraser C L (2009). Nat Mater.

[13] DeRosa C A, Seaman S A, Mathew A S, Gorick C M, Fan Z, Demas J N, Peirce S M, Fraser C L (2016). ACS Sens.

[14] Lehner P, Staudinger C, Borisov S M, Klimant I (2014). Nat Commun.

[15] Kwon M S, Lee D, Seo S, Jung J, Kim J (2014). Angew Chem, Int Ed.

[16] Xiao L, Fu H (2019). Chem – Eur J.

[17] Jia W, Wang Q, Shi H, An Z, Huang W (2020). Chem – Eur J.

[18] Kasha M (1947). Chem Rev.

[19] Lewis G N, Calvin M (1945). J Am Chem Soc.

[20] Lewis G N, Kasha M (1944). J Am Chem Soc.

[21] Lewis G N, Lipkin D, Magel T T (1941). J Am Chem Soc.

[22] Lower S K, El-Sayed M A (1966). Chem Rev.

[23] El‐Sayed M A (1963). J Chem Phys.

[24] Hirata S (2017). Adv Opt Mater.

[25] Menning S, Krämer M, Coombs B A, Rominger F, Beeby A, Dreuw A, Bunz U H F (2013). J Am Chem Soc.

[26] Yuan W Z, Shen X Y, Zhao H, Lam J W Y, Tang L, Lu P, Wang C, Liu Y, Wang Z, Zheng Q (2010). J Phys Chem C.

[27] Zhang G, Chen J, Payne S J, Kooi S E, Demas J N, Fraser C L (2007). J Am Chem Soc.

[28] Xu J, Takai A, Kobayashi Y, Takeuchi M (2013). Chem Commun.

[29] Schulze M, Steffen A, Würthner F (2015). Angew Chem, Int Ed.

[30] Hirata S, Totani K, Yamashita T, Adachi C, Vacha M (2014). Nat Mater.

[31] Pan Z, Lu Y-Y, Liu F (2012). Nat Mater.

[32] Tong B, Mei Q, Wang S, Fang Y, Meng Y, Wang B (2008). J Mater Chem.

[33] Liu Z W, Guan M, Bian Z Q, Nie D B, Gong Z L, Li Z B, Huang C H (2006). Adv Funct Mater.

[34] Zhao J, Chen K, Hou Y, Che Y, Liu L, Jia D (2018). Org Biomol Chem.

[35] Forni A, Lucenti E, Botta C, Cariati E (2018). J Mater Chem C.

[36] Baroncini M, Bergamini G, Ceroni P (2017). Chem Commun.

[37] Mukherjee S, Thilagar P (2015). Chem Commun.

[38] Shi H, Song L, Ma H, Sun C, Huang K, Lv A, Ye W, Wang H, Cai S, Yao W (2019). J Phys Chem Lett.

[39] Li J-A, Zhou J, Mao Z, Xie Z, Yang Z, Xu B, Liu C, Chen X, Ren D, Pan H (2018). Angew Chem, Int Ed.

[40] Xiong Y, Zhao Z, Zhao W, Ma H, Peng Q, He Z, Zhang X, Chen Y, He X, Lam J W Y (2018). Angew Chem, Int Ed.

[41] Gu L, Shi H, Miao C, Wu Q, Cheng Z, Cai S, Gu M, Ma C, Yao W, Gao Y (2018). J Mater Chem C.

[42] Cai S, Shi H, Zhang Z, Wang X, Ma H, Gan N, Wu Q, Cheng Z, Ling K, Gu M (2018). Angew Chem, Int Ed.

[43] Cai S, Shi H, Tian D, Ma H, Cheng Z, Wu Q, Gu M, Huang L, An Z, Peng Q (2018). Adv Funct Mater.

[44] Cai S, Shi H, Li J, Gu L, Ni Y, Cheng Z, Wang S, Xiong W-w, Li L, An Z (2017). Adv Mater (Weinheim, Ger).

[45] Yang Z, Mao Z, Zhang X, Ou D, Mu Y, Zhang Y, Zhao C, Liu S, Chi Z, Xu J (2016). Angew Chem, Int Ed.

[46] Gong Y, Chen G, Peng Q, Yuan W Z, Xie Y, Li S, Zhang Y, Tang B Z (2015). Adv Mater (Weinheim, Ger).

[47] An Z, Zheng C, Tao Y, Chen R, Shi H, Chen T, Wang Z, Li H, Deng R, Liu X (2015). Nat Mater.

[48] Yu Z, Wu Y, Peng Q, Sun C, Chen J, Yao J, Fu H (2016). Chem – Eur J.

[49] Chen X, Xu C, Wang T, Zhou C, Du J, Wang Z, Xu H, Xie T, Bi G, Jiang J (2016). Angew Chem, Int Ed.

[50] Lin Z, Kabe R, Nishimura N, Jinnai K, Adachi C (2018). Adv Mater (Weinheim, Ger).

[51] Li D, Lu F, Wang J, Hu W, Cao X-M, Ma X, Tian H (2018). J Am Chem Soc.

[52] Kabe R, Adachi C (2017). Nature.

[53] Gan N, Wang X, Ma H, Lv A, Wang H, Wang Q, Gu M, Cai S, Zhang Y, Fu L (2019). Angew Chem, Int Ed.

[54] Yang J, Ren Z, Chen B, Fang M, Zhao Z, Tang B Z, Peng Q, Li Z (2017). J Mater Chem C.

[55] Shimizu M, Shigitani R, Nakatani M, Kuwabara K, Miyake Y, Tajima K, Sakai H, Hasobe T (2016). J Phys Chem C.

[56] Xie Y, Ge Y, Peng Q, Li C, Li Q, Li Z (2017). Adv Mater (Weinheim, Ger).

[57] Bergamini G, Fermi A, Botta C, Giovanella U, Di Motta S, Negri F, Peresutti R, Gingras M, Ceroni P (2013). J Mater Chem C.

[58] Bolton O, Lee K, Kim H-J, Lin K Y, Kim J (2011). Nat Chem.

[59] Yu Y, Kwon M S, Jung J, Zeng Y, Kim M, Chung K, Gierschner J, Youk J H, Borisov S M, Kim J (2017). Angew Chem, Int Ed.

[60] Kwon M S, Jordahl J H, Phillips A W, Chung K, Lee S, Gierschner J, Lahann J, Kim J (2016). Chem Sci.

[61] Kwon M S, Yu Y, Coburn C, Phillips A W, Chung K, Shanker A, Jung J, Kim G, Pipe K, Forrest S R (2015). Nat Commun.

[62] Lee D, Jung J, Bilby D, Kwon M S, Yun J, Kim J (2015). ACS Appl Mater Interfaces.

[63] Bolton O, Lee D, Jung J, Kim J (2014). Chem Mater.

[64] Goudappagouda, Manthanath A, Wakchaure V C, Ranjeesh K C, Das T, Vanka K, Nakanishi T, Babu S S (2019). Angew Chem, Int Ed.

[65] Kuila S, Rao K V, Garain S, Samanta P K, Das S, Pati S K, Eswaramoorthy M, George S J (2018). Angew Chem, Int Ed.

[66] Yu Z, Wu Y, Xiao L, Chen J, Liao Q, Yao J, Fu H (2017). J Am Chem Soc.

[67] Huang C-H, Wu P-J, Chung K-Y, Chen Y-A, Li E Y, Chou P-T (2017). Phys Chem Chem Phys.

[68] Ventura B, Bertocco A, Braga D, Catalano L, d’Agostino S, Grepioni F, Taddei P (2014). J Phys Chem C.

[69] Koch M, Perumal K, Blacque O, Garg J A, Saiganesh R, Kabilan S, Balasubramanian K K, Venkatesan K (2014). Angew Chem, Int Ed.

[70] Göbel D, Clamor N, Nachtsheim B J (2018). Org Biomol Chem.

[71] Göbel D, Clamor N, Lork E, Nachtsheim B J (2019). Org Lett.

[72] Göbel D, Duvinage D, Stauch T, Nachtsheim B J (2020). J Mater Chem C.

[73] Sarkar S, Hendrickson H P, Lee D, DeVine F, Jung J, Geva E, Kim J, Dunietz B D (2017). J Phys Chem C.

[74] Xie S, Proietti G, Ramström O, Yan M (2019). J Org Chem.

[75] Lord S J, Lee H-l D, Samuel R, Weber R, Liu N, Conley N R, Thompson M A, Twieg R J, Moerner W E (2010). J Phys Chem B.

