# Supplementary material for: Clickable azide-functionalized bromoarylaldehydes – synthesis and photophysical characterization
Source: Beilstein J Org Chem. 2020 Jul 14;16:1683–92. doi: 10.3762/bjoc.16.139 (PMC7372231; doi:10.3762/bjoc.16.139)
Supplement: File 1 — Detailed experimental procedures, characterization data, photophysical properties, and copies of NMR spectra. [file Beilstein_J_Org_Chem-16-1683-s001.pdf]

BEILSTEIN JOURNAL OF ORGANIC CHEMISTRY

Supporting Information

for

Clickable azide-functionalized bromoarylaldehydes – synthesis and photophysical characterization

Dominik Göbel, Marius Friedrich, Enno Lork and Boris J. Nachtsheim

Beilstein J. Org. Chem. **2020,** *16,* 1683–1692. doi:10.3762/bjoc.16.139

Detailed experimental procedures, characterization data, photophysical properties, and copies of NMR spectra

License and Terms: This is a supporting information file under the terms of the Creative Commons Attribution License (http://creativecommons.org/licenses/by/4.0). Please note that the reuse, redistribution and reproduction in particular requires that the authors and source are credited.
The license is subject to the *Beilstein Journal of Organic Chemistry* terms and conditions: (https://www.beilstein-journals.org/bjoc)

Table of Content

1 General Information **S1**

2 Experimental Procedures **S3**

2.1 Preparation of Metalation Agents **S3**

2.1.1 Preparation of the Reagent *i*PrMgCl·LiCl **S3**

2.1.2 Preparation of the Reagent TMPMgCl·LiCl **S3**

2.2 Preparation of 2-(Azidomethyl)-4-bromobenzaldehyde (3) **S4**

2.2.1 2-(4-Bromophenyl)-4,4-dimethyl-4,5-dihydrooxazole (6) **S4**

2.2.2 5-Bromo-2-(4,4-dimethyl-4,5-dihydrooxazol-2-yl)benzaldehyde (7) **S5**

2.2.3 (5-Bromo-2-(4,4-dimethyl-4,5-dihydrooxazol-2-yl)phenyl)methanol (8) **S6**

2.2.4 2-(2-(Azidomethyl)-4-bromophenyl)-4,4-dimethyl-4,5-dihydrooxazole (9) **S6**

2.2.5 2-(Azidomethyl)-4-bromobenzaldehyde (3) **S7**

2.3 Preparation of 4-(Azidomethyl)-2-bromobenzaldehyde (4) **S8**

2.3.1 4,4-Dimethyl-2-(*p*-tolyl)-4,5-dihydrooxazole (10) **S8**

2.3.2 2-(4-(Bromomethyl)phenyl)-4,4-dimethyl-4,5-dihydrooxazole (S1) **S9**

2.3.3 2-(4-(Azidomethyl)phenyl)-4,4-dimethyl-4,5-dihydrooxazole (S2) **S10**

2.3.4 2-(2-Bromo-4-methylphenyl)-4,4-dimethyl-4,5-dihydrooxazole (11) **S10**

2.3.5 2-(2-Bromo-4-(bromomethyl)phenyl)-4,4-dimethyl-4,5-dihydrooxazole (12) **S11**

2.3.6 2-(4-(Azidomethyl)-2-bromophenyl)-4,4-dimethyl-4,5-dihydrooxazole (13) **S12**

2.3.7 4-(Azidomethyl)-2-bromobenzaldehyde (4) **S12**

2.4 Preparation of Highly Functionalized Fluorenes (5), (23), (25) & (28) **S14**

2.4.1 2,7-Dibromo-9*H*-fluorene (14) **S15**

2.4.2 2,7-Dibromo-9,9-dimethyl-9*H*-fluorene (15) **S15**

2.4.3 7-Bromo-9,9-dimethyl-9*H*-fluorene-2-carbaldehyde (16) **S16**

2.4.4 2-(7-Bromo-9,9-dimethyl-9*H*-fluoren-2-yl)-4,4-dimethyl-4,5-dihydrooxazole (17)... **S17**

2.4.5 7-Bromo-2-(4,4-dimethyl-4,5-dihydrooxazol-2-yl)-9,9-dimethyl-9*H*-fluorene-3-carbaldehyde (18) **S17**

2.4.6 (7-Bromo-2-(4,4-dimethyl-4,5-dihydrooxazol-2-yl)-9,9-dimethyl-9*H*-fluoren-3-yl)methanol (19) **S18**

2.4.7 7-Bromo-9,9-dimethyl-3,9-dihydro-1*H*-fluoreno[2,3-*c*]furan-1-one (20) **S19**

2.4.8 2-(3-(Azidomethyl)-7-bromo-9,9-dimethyl-9*H*-fluoren-2-yl)-4,4-dimethyl-4,5-dihydrooxazole (21) **S20**

2.4.9 3-(Azidomethyl)-7-bromo-9,9-dimethyl-9*H*-fluorene-2-carbaldehyde (5) **S21**

2.4.10 2-(7-Bromo-3-(methoxymethyl)-9,9-dimethyl-9*H*-fluoren-2-yl)-4,4-dimethyl-4,5-dihydrooxazole (22) **S22**

2.4.11 7-Bromo-3-(methoxymethyl)-9,9-dimethyl-9*H*-fluorene-2-carbaldehyde (23) **S22**

2.4.12 9-Bromo-3,3,11,11-tetramethyl-2,3,5,11-tetrahydroindeno[1,2-f]oxazolo[2,3-*a*]isoindol-4-ium bromide (25) S23
2.4.13 9-Bromo-3,3,4,11,11-pentamethyl-2,3,4,5,11,12b-hexahydroindeno[1,2-*f*]oxazolo[2,3-*a*]isoindol-4-ium trifluoromethanesulfonate (28) S25
2.4.14 Attempted Preparation of (7-bromo-2-(4,4-dimethyl-4,5-dihydrooxazol-2-yl)-9,9-dimethyl-9H-fluoren-3-yl)methyl methanesulfonate (S3) S26
2.5 Preparation of Sterically Hindered Alkynes S27
2.5.1 Preparation of (3*r*,5*r*,7*r*)-1-ethynyladamantane (31) S27
2.5.2 Preparation of 1,3-di-*tert*-butyl-5-ethynylbenzene (32) S28
2.5.2.1 3,5-Di-*tert*-butylbenzaldehyde (S4) S28
2.5.2.2 1,3-Di-*tert*-butyl-5-(2,2-dibromovinyl)benzene (S5) S29
2.5.2.3 1,3-Di-*tert*-butyl-5-ethynylbenzene (32) S30
2.6 Copper-Catalyzed Azide-Alkyne Cycloadditions (CuAAC) S31
2.6.1 General Procedure for the CuAACs (GP1) S31
2.6.2 4-Bromo-2-((4-octyl-1*H*-1,2,3-triazol-1-yl)methyl)benzaldehyde (33) S31
2.6.3 2-((4-((1*s*,3*s*)-Adamantan-1-yl)-1*H*-1,2,3-triazol-1-yl)methyl)-4-bromobenzaldehyde (34) S32
2.6.4 4-Bromo-2-((4-phenyl-1*H*-1,2,3-triazol-1-yl)methyl)benzaldehyde (35) S32
2.6.5 4-Bromo-2-((4-(3,5-di-*tert*-butylphenyl)-1*H*-1,2,3-triazol-1-yl)methyl)benzaldehyde (36) S33
2.6.6 2-Bromo-4-((4-octyl-1*H*-1,2,3-triazol-1-yl)methyl)benzaldehyde (37) S33
2.6.7 4-((4-((1*s*,3*s*)-Adamantan-1-yl)-1*H*-1,2,3-triazol-1-yl)methyl)-2-bromobenzaldehyde (38) S34
2.6.8 2-Bromo-4-((4-phenyl-1*H*-1,2,3-triazol-1-yl)methyl)benzaldehyde (39) S35
2.6.9 2-Bromo-4-((4-(3,5-di-*tert*-butylphenyl)-1*H*-1,2,3-triazol-1-yl)methyl)benzaldehyde (40) S35
2.6.10 7-Bromo-9,9-dimethyl-3-((4-octyl-1*H*-1,2,3-triazol-1-yl)methyl)-9*H*-fluorene-2-carbaldehyde (41) S36
2.6.11 3-((4-((3*r*,5*r*,7*r*)-Adamantan-1-yl)-1*H*-1,2,3-triazol-1-yl)methyl)-7-bromo-9,9-dimethyl-9*H*-fluorene-2-carbaldehyde (42) S37
2.6.12 7-Bromo-9,9-dimethyl-3-((4-phenyl-1*H*-1,2,3-triazol-1-yl)methyl)-9*H*-fluorene-2-carbaldehyde (43) S37
2.6.13 7-Bromo-3-((4-(3,5-di-*tert*-butylphenyl)-1*H*-1,2,3-triazol-1-yl)methyl)-9,9-dimethyl-9*H*-fluorene-2-carbaldehyde (44) S38
2.7 Methylation Reaction of Adamantyl substituted triazole (42) S39
2.7.1 4-((3*r*,5*r*,7*r*)-Adamantan-1-yl)-1-((7-bromo-2-formyl-9,9-dimethyl-9*H*-fluoren-3-yl)methyl)-3-methyl-1*H*-1,2,3-triazol-3-ium tetrafluoroborate (45) S39

3 Optical Properties S40

3.1 2-(Azidomethyl)-4-bromobenzaldehyde (3) & Derivatives S40

3.2 4-(Azidomethyl)-2-bromobenzaldehyde (4) & Derivatives S43

3.3 3-(Azidomethyl)-7-bromo-9,9-dimethyl-9*H*-fluorene-2-carbaldehyde (5) & Derivatives S45

4 Crystal Structure of 3-(Azidomethyl)-7-bromo-9,9-dimethyl-9*H*-fluorene-2-carbaldehyde (5) S62

4.1 Crystal Data for (5) S62

4.2 Crystal Packing Views of (5) S70

5 References S72

6 NMR-Spectra S75

1 General Information

Unless otherwise noted, all reactions were carried out under a nitrogen atmosphere using standard *Schlenk* techniques. All chemicals were purchased from commercial suppliers and either used as received or purified according to *Purification of Common Laboratory Chemicals*.[1] Anhydrous tetrahydrofuran (THF) and dichloromethane (CH_2Cl_2) were obtained from an *inert* PS-MD-6 solvent purification system. All other solvents were dried using standard methods.[1] Yields refer to isolated yields of compounds estimated to be > 95 % pure as determined by ^{1}H-NMR spectroscopy.

Thin layer chromatography was performed on fluorescence indicator marked precoated silica gel 60 plates (*Macherey-Nagel*, ALUGRAM Xtra SIL G/UV_{254}) and visualized by UV light (254 nm/366 nm) or staining solution. $KMnO_4$ staining solution was prepared using the following recipe: $KMnO_4$ (1.5 g), K_2CO_3 (10 g) and aqueous NaOH (1.25 mL, 10 %) in water (200 mL). Flash column chromatography was performed on silica gel (0.040 – 0.063 mm) with the solvents given in the procedures. Abbreviations for solvents used: CH = cyclohexane, EE = ethyl acetate. Retention factors were determined at chamber saturation at 25 °C. Developments were carried out between 3.0 – 3.5 cm. If not otherwise stated, crude products were adsorbed on silica gel (up to twice the amount of crude product mass) prior to purification by flash column chromatography.

NMR spectra were recorded on a *Bruker* Avance 360WB spectrometer, a *Bruker* Avance Neo 600 MHz spectrometer with BBO probe head and a *Bruker* Avance Neo 600 MHz spectrometer with TXI probe head at 23 °C. Chemical shifts for ^{1}H-NMR spectra are reported as δ (parts per million) relative to the residual proton signal of $CDCl_3$ at 7.26 ppm (s), MeOD-d_4 at 3.31 ppm (p). Chemical shifts for ^{13}C-NMR spectra are reported as δ (parts per million) relative to the signal of $CDCl_3$ at 77.0 ppm (t), MeOD-d_4 at 49.0 ppm (sept). Chemical shifts for ^{19}F-NMR spectra are reported as δ (parts per million) relative to the signal of $Si(CH_3)_4$ at 0.0 ppm. The following abbreviations are used to describe splitting patterns: br. = broad, s = singlet, d = doublet, dd = doublet of doublets, ddd = doublet of doublet of doublets, t = triplet, p = pentet, m = multiplet. Coupling constants *J* are given in Hertz.

ESI and APCI mass spectra were recorded on an *Advion* Expression CMS^L *via* ASAP probe or direct inlet. EI mass spectra were obtained from an Agilent 7890B GC System with an Agilent 5977A MSD mass spectrometer. As column a HP-5M5 (5%-phenyl)-methylpolysiloxane was used. High resolution (HR) EI mass spectra were recorded on a double focusing mass spectrometer ThermoQuest MAT 95 XL from *Finnigan MAT*. HR-ESI and HR-APCI mass spectra were recorded on a *Bruker* Impact II. All Signals are reported with the quotient from mass to charge m/z.

IR spectra were recorded on a *Nicolet* Thermo iS10 scientific spectrometer with a diamond ATR unit. The absorption bands are reported in cm^{-1} with indicated relative intensities: s (strong, 0 – 33 % T); m (medium, 34 – 66 % T), w (weak, 67 – 100 % T), and br (broad).

Melting points of solids, compounds that solidified after chromatography, were measured on a *Büchi* M-5600 Melting Point apparatus and are uncorrected. The measurements were performed with a heating rate of 5 °C/min and the melting points are reported in °C.

Low temperature reactions (–78 °C) were cooled using a *Julabo* FT902 cryostat. If not otherwise noted, solvents were removed on a *Büchi* Rotavapor R-300 with 40 °C water bath temperature.

Absorption measurements were performed either using an *Agilent* Cary 5000 UV-Vis-NIR spectrophotometer or a *Shimadzu* UV-2700 UV-Vis spectrophotometer. Emission measurements were performed using an *Edinburgh Instruments* FLS 1000 photoluminescence spectrometer. Absolute quantum yields were measured using an *Edinburgh* integrating sphere. TCSPC measurements were performed using a fast response MCP-PMT detector on the FLS 1000 and a 376 nm *Edinburgh* EPL Laser as excitation source with 10 – 20 MHz repetition rate and 80 ps pulse width. All measurements were performed at 23 °C room temperature in Quartz Cuvettes with 10 mm path length by *Hellma Analytics*.

Single crystals were grown as described in the procedure below. Intensity data of suitable crystals were collected on a *Bruker* Venture D8 diffractometer at 100 K with Mo-Kα (0.71073 Å) radiation. All structures were solved by direct methods and refined based on F^2 by use of the SHELX program package as implemented in Olex2.[2, 3] All non-hydrogen atoms were refined using anisotropic displacement parameters. Hydrogen atoms attached to carbon atoms were included in geometrically calculated positions using a rigid model. Crystal and refinement data are collected in Tables S4-22. Figures were created using Olex2. Crystallographic data for the structural analyses have been deposited with the Cambridge Crystallographic Data Centre. Copies of this information may be obtained free of charge from The Director, CCDC, 12 Union Road, Cambridge CB2 1EZ, UK (Fax: +44-1223-336033; e-mail: deposit@ccdc.cam.ac.uk or http://www.ccdc.cam.ac.uk).

2 Experimental Procedures

2.1 Preparation of Metalation Agents

2.1.1 Preparation of the Reagent *i*PrMgCl·LiCl

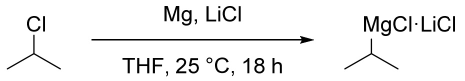

A slightly modified literature procedure was used.[4] LiCl (4.24 g, 100 mmol, 1.00 eq) was placed in a heat gun-dried and nitrogen-flushed *Schlenk* flask and heated *in vacuo* at 140 °C by heat gun for five hours. Magnesium turnings (2.67 g, 110 mmol, 1.10 eq) were placed in another heat gun-dried and nitrogen-flushed *Schlenk* flask and the dried LiCl and anhydrous THF (50 mL) were added. 2-Chloropropane (9.14 mL, 100 mmol, 1.00 eq) in anhydrous THF (50 mL) was slowly added at 25 °C through a dropping funnel. After approximately 1/5 of addition the mixture was slightly warmed with a heat gun until the reaction started (within ten minutes). When the reaction started the remaining solution was added dropwise and stirring was continued for 18 hours. After complete addition the temperature of the mixture rose until it started to boil. To remove excess of magnesium the grey solution was cannulated to another heat gun-dried and nitrogen-flushed *Schlenk* flask. The Grignard reagent was titrated[5] prior to use against I_2 (0.50 – 0.60 mmol) in anhydrous THF (2 mL) at 0 °C which resulted in a conversion of 92 – 96 %. Color change from dark violet to pale brown indicated the end of the titration.

2.1.2 Preparation of the Reagent TMPMgCl·LiCl

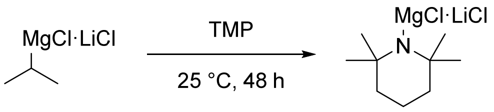

A slightly modified literature procedure was used.[6] A heat gun-dried and nitrogen-flushed *Schlenk* flask was charged with freshly titrated *i*PrMgCl·LiCl (75.0 mL, 90.0 mmol, 1.00 eq, 1.20 M). Freshly distilled TMP (16.0 mL, 94.5 mmol, 1.05 eq) was added through a rubber septum to the vigorously stirred Grignard solution *via* syringe pump (0.5 mL/min) at 25 °C. The reaction mixture was stirred at 25 °C for 48 hours, while the solution turned dark green. The base was titrated[7] prior to use against benzoic acid (122 mg, 1.00 mmol) using (4-phenylazo)diphenylamine (3 mg) as indicator in anhydrous THF (2.00 mL) at 0 °C which resulted in a conversion of 96 – 99 %. Color change from orange to dark violet indicated the end of the titration.

2.2 Preparation of 2-(Azidomethyl)-4-bromobenzaldehyde (3)

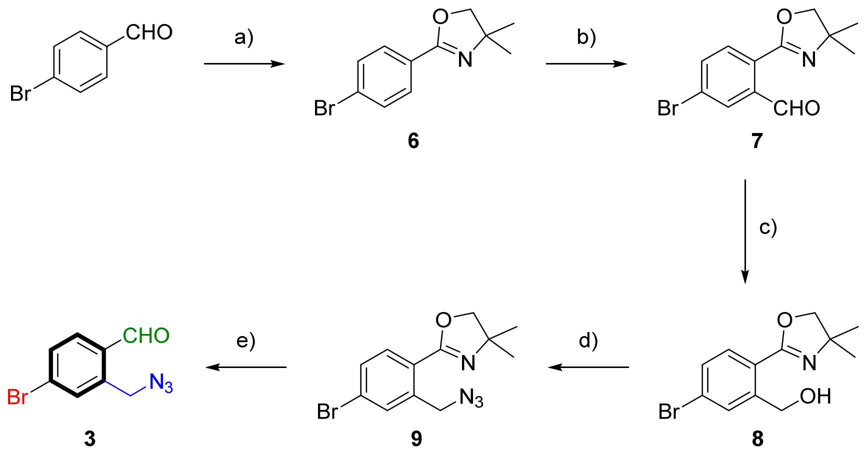

Reaction conditions: a) 1) 2-amino-2-methylpropan-1-ol, 4 Å MS, CH_2Cl_2, 25 °C, 18 h; 2) NBS, CH_2Cl_2, 25 °C, 5 h, 91 %; b) 1) TMPMgCl·LiCl, THF, 25 °C, 4 h; 2) DMF, THF, 0 °C to 25 °C, 1.5 h; c) $NaBH_4$, THF:MeOH 1:1, 0 °C, 1 h, 81 % (2 steps); d) DPPA, DBU, PhMe, 25 °C, 18 h, 98 %; e) 1) MeOTf, CH_2Cl_2, 25 °C, 2.5 h; 2) $NaBH_4$, THF:MeOH 4:1 v:v, 0 °C, 2.5 h; 3) oxalic acid, THF:H_2O 4:1 v:v, 25 °C, 20 h, 78 %. Overall yield: 56 % (5 steps).

2.2.1 2-(4-Bromophenyl)-4,4-dimethyl-4,5-dihydrooxazole (6)

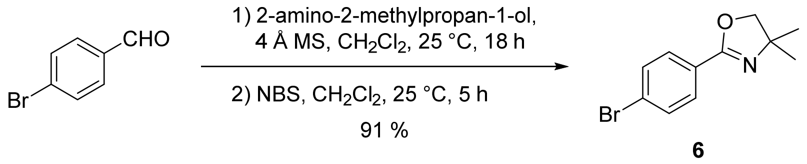

A modified literature procedure was used.[8] 4-Bromobenzaldehyde (5.55 g, 30.0 mmol, 1.00 eq) and 2-amino-2-methylpropan-1-ol (5.35 g, 60.0 mmol, 2.00 eq) were dissolved in CH_2Cl_2 (100 mL, 0.3 M) and 4 Å MS (20.0 g) was added. After 18 hours of slowly stirring at 25 °C NBS (10.7 g, 60.0 mmol, 2.00 eq) was added in one portion and stirring was continued for another five hours at 25 °C. All solids were filtered off and the organic phase was washed with saturated $NaHCO_3$ solution (100 mL). The combined aqueous phases were extracted with CH_2Cl_2 (3 × 50 mL). All organic phases were washed with saturated NaS_2O_3 solution (100 mL) and the aqueous phase was extracted with CH_2Cl_2 (50 mL). The combined organic phases were dried over anhydrous $MgSO_4$, filtered and the solvent was removed under reduced pressure. Purification by flash column chromatography (SiO_2, CH:EE 10:1 v:v) afforded **6** (6.97 g, 27.4 mmol, 91 %) as a colorless oil which solidified upon standing to form a pale brown solid.

R_f = 0.26 (SiO_2, CH:EE 10:1 v:v). **Mp.:** 38 – 40 °C (Lit.[9]: 37 – 38 °C). **^{1}H-NMR (360 MHz, $CDCl_3$):** δ = 7.83 – 7.74 (AA'XX', 2H), 7.57 – 7.47 (AA'XX', 2H), 4.09 (s, 2H), 1.36 (s, 6H) ppm. **$^{13}C\{^1H\}$-NMR (91 MHz, $CDCl_3$):** δ = 161.4, 131.6 (2x), 129.9 (2x), 127.1, 125.9, 79.3, 67.9, 28.5 (2x) ppm. **IR (ATR, neat):** $\tilde{\nu}$ = 2969 (m), 2624(w), 2897 (w), 1642 (s), 1591 (m), 1479 (m), 1459 (m), 1397 (m), 1357 (m), 1315 (m), 1296 (m), 1177 (m), 1070 (s), 1063 (s), 1009 (s), 964 (m), 923 (m), 835 (s), 824 (m), 726 (s),

712 (m), 676 (s) cm^{-1}. MS (APCI): m/z = 254.0 $[C_{11}H_{12}{}^{79}BrNO+H]^+$. **HR-MS (APCI):** calculated for $C_{11}H_{13}{}^{79}BrNO^+$ $[M+H]^+$: m/z = 254.01750, found: 254.01748 (Dev.: –0.02 mu; –0.10 ppm). The analytical data are in accordance with the literature.[10]

2.2.2 5-Bromo-2-(4,4-dimethyl-4,5-dihydrooxazol-2-yl)benzaldehyde (7)

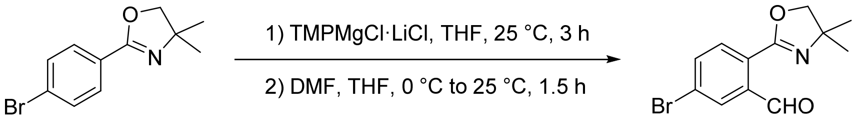

6 **7**

A modified literature procedure was used.[10] A heat gun-dried and nitrogen-flushed *Schlenk* flask, equipped with a magnetic stirring bar and a rubber septum was charged with **6** (3.30 g, 13.0 mmol, 1.00 eq) and evacuated for half an hour. The flask was flushed with nitrogen and anhydrous THF (32.5 mL, 0.4 M) was added. Addition of TMPMgCl·LiCl (26.0 mL, 26.0 mmol, 2.00 eq, 1.00 M in THF) *via* syringe within three minutes at 25 °C was followed by stirring for three hours at the same conditions. DMF (4.03 mL, 52.0 mmol, 4.00 eq) was diluted in anhydrous THF (10 mL) and added *via* syringe pump (0.5 mL/min) to the dark red solution at 0 °C. After complete addition the mixture was stirred for one and a half hours while it was allowed to warm to 25 °C. Full consumption of the starting material was followed by diluting with water (50 mL) and ethyl acetate (50 mL). The phases were separated and the aqueous layer was extracted with ethyl acetate (4 × 50 mL). The combined organic phases were dried over anhydrous Na_2SO_4, filtered and concentrated under reduced pressure on a rotary evaporator at 30 °C. Due to its sensibility, the crude product was directly used for the next step. If desired the product can be isolated by rapid flash column chromatography (SiO_2, CH:EE 10:1 v:v) to afford **7** as a yellowish oil.

$\mathbf{R_f}$ = 0.23 (SiO_2, CH:EE 10:1 v:v). **^{1}H-NMR (360 MHz, $CDCl_3$):** δ = 10.71 (s, 1H), 8.05 (d, $^4J_{HH}$ = 2.1 Hz, 1H), 7.78 (d, $^3J_{HH}$ = 8.3 Hz, 1H), 7.71 (dd, $^3J_{HH}$ = 8.3 Hz, $^4J_{HH}$ = 2.1 Hz, 1H), 4.14 (s, 2H), 1.39 (s, 6H) ppm. **^{13}C{^{1}H}-NMR (91 MHz, $CDCl_3$):** δ = 191.3, 159.6, 137.4, 135.9, 131.6, 131.0, 128.6, 125.9, 79.5, 69.0, 28.5 (2x) ppm. **IR (ATR, neat):** $\tilde{\nu}$ = 2967 (w), 2928 (w), 2894 (w), 1687 (s), 1644 (m), 1582 (m), 1563 (w), 1478 (w), 1381 (w), 1364 (w), 1351 (m), 1305 (m), 1284 (w), 1251 (w), 1212 (w), 1182 (m), 1129 (w), 1082 (w), 1046 (s), 987 (w), 963 (m), 922 (w), 881 (w), 865 (w), 836 (m), 819 (w), 712 (m), 677 (m) cm^{-1}. **MS (APCI):** m/z = 282.0 $[C_{12}H_{12}{}^{79}BrNO_2+H]^+$. **HR-MS (ESI):** calculated for $C_{12}H_{13}{}^{79}BrNO_2^+$ $[M+H]^+$: m/z = 282.01242, found: 282.01244 (Dev.: 0.02 mu; 0.07 ppm).

2.2.3 (5-Bromo-2-(4,4-dimethyl-4,5-dihydrooxazol-2-yl)phenyl)methanol (8)

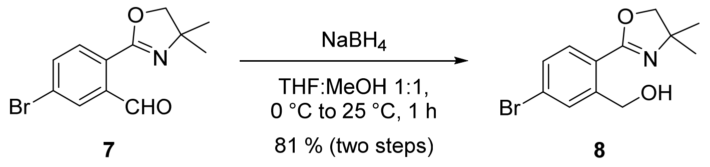

A modified literature procedure was used.[11] The crude benzaldehyde **7** (13.0 mmol, 1.00 eq) was dissolved in THF:MeOH (26 mL, 1:1 v:v, 0.5 M) and cooled to 0 °C. $NaBH_4$ (984 mg, 26.0 mmol, 2.00 eq) was added and stirring was continued for two more hours while the mixture was allowed to warm slowly to 25 °C. Saturated NH_4Cl solution (20 mL), ethyl acetate (20 mL) and water (30 mL) were added successively and the phases were separated. The aqueous layer was extracted with ethyl acetate (4 × 50 mL), the combined organic phases were dried over anhydrous Na_2SO_4, filtered and concentrated under reduced pressure. Purification by flash column chromatography (SiO_2, CH:EE 10:1 v:v) afforded **8** (2.98 g, 10.5 mmol, 81 % over two steps) as a colorless oil.

R_f = 0.17 (SiO_2, CH:EE 10:1 v:v). **^{1}H-NMR (360 MHz, $CDCl_3$):** δ = 7.71 (d, $^3J_{HH}$ = 8.2 Hz, 1H), 7.51 (d, $^4J_{HH}$ = 2.1 Hz, 1H), 7.47 (dd, $^3J_{HH}$ = 8.3 Hz, $^4J_{HH}$ = 2.1 Hz, 1H), 6.65 (t, $^3J_{HH}$ = 7.6 Hz, 1H), 4.59 (d, $^3J_{HH}$ = 7.6 Hz, 2H), 4.12 (s, 2H), 1.39 (s, 6H) ppm. **^{13}C{^{1}H}-NMR (91 MHz, $CDCl_3$):** δ = 161.6, 144.0, 133.4, 131.6, 130.8, 126.0, 125.7, 78.9, 68.2, 64.1, 28.5 (2x) ppm. **IR (ATR, neat):** $\tilde{\nu}$ = 3270 (w), 2966 (w), 2928 (w), 2895 (w), 1636 (s), 1586 (w), 1561 (w), 1462 (w), 1386 (w), 1352 (m), 1308 (m), 1284 (w), 1186 (m), 1090 (m), 1054 (s), 1032 (s), 963 (m), 875 (w), 844 (m), 826 (m), 706 (m), 679 (m) cm^{-1}. **MS (APCI):** m/z = 284.0 $[C_{12}H_{14}{}^{79}BrNO_2+H]^+$. **HR-MS (EI, 70 eV):** calculated for $C_{12}H_{14}{}^{79}BrNO_2{}^{+\cdot}$ $[M]^{+\cdot}$: m/z = 283.02024, found: 283.02011 (Dev.: –0.13 mu; –0.47 ppm).

2.2.4 2-(2-(Azidomethyl)-4-bromophenyl)-4,4-dimethyl-4,5-dihydrooxazole (9)

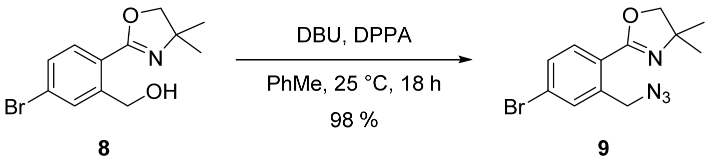

A modified literature procedure was used.[12] **8** (1.42 g, 5.00 mmol, 1.00 eq) was dissolved in PhMe (16.7 mL, 0.3 M) and DBU (970 µL, 6.50 mmol, 1.30 eq) was added *via* syringe pump (1.0 mL/min) at 25 °C. The mixture was stirred for ten minutes and DPPA (1.29 mL, 6.00 mmol, 1.20 eq) was added *via* syringe pump (0.3 mL/min). After complete addition the suspension was stirred for 18 hours at 25 °C. Saturated NH_4Cl solution (50 mL) and ethyl acetate (100 mL) were added and the phases separated. The aqueous phase was extracted with ethyl acetate (3 × 50 mL) and the combined organic phases were dried over anhydrous Na_2SO_4. After filtration the solvent was removed under reduced pressure and purification was conducted by flash column chromatography (SiO_2, CH:EE 15:1 v:v) to provide **9** (1.52 g, 4.92 mmol, 98 %) as a colorless liquid.

R_f = 0.30 (SiO_2, CH:EE 15:1 v:v). **Mp.:** around 0 °C. **^{1}H-NMR (360 MHz, $CDCl_3$):** δ = 7.75 (d, $^3J_{HH}$ = 8.4 Hz, 1H), 7.62 (d, $^4J_{HH}$ = 2.1 Hz, 1H), 7.49 (dd, $^3J_{HH}$ = 8.4 Hz, $^4J_{HH}$ = 2.0 Hz, 1H), 4.87 (s, 2H), 4.06 (s, 2H), 1.37 (s, 6H) ppm. **^{13}C{^{1}H}-NMR (91 MHz, $CDCl_3$):** δ = 160.4, 138.4, 132.3, 131.7, 131.1, 125.6, 125.6, 78.6, 68.6, 52.8, 28.5 (2x) ppm. **IR (ATR, neat):** $\tilde{\nu}$ = 2966 (w), 2929 (w), 2892 (w), 2099 (s), 1642 (m), 1588 (w), 1564 (w), 1481 (w), 1350 (w), 1338 (w), 1304 (m), 1277 (m), 1212 (w), 1184 (m), 1090 (w), 1042 (s), 967 (w), 923 (w), 872 (w), 830 (m), 741 (w), 679 (w) cm^{-1}. **MS (APCI):** m/z = 309.0 $[C_{12}H_{13}{}^{79}BrN_4O+H]^+$. **HR-MS (ESI):** calculated for $C_{12}H_{13}{}^{79}BrN_4O^+$ $[M+H]^+$: m/z = 309.03455, found: 309.03499 (Dev.: 0.44 mu; 1.43 ppm).

2.2.5 2-(Azidomethyl)-4-bromobenzaldehyde (3)

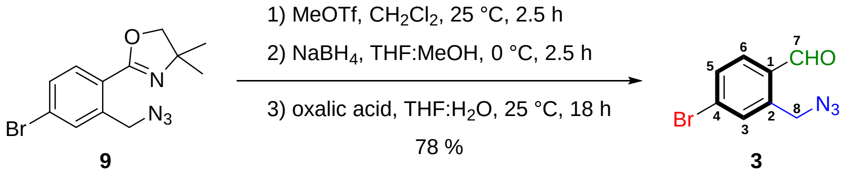

A modified literature procedure was used.[13] **9** (1.86 g, 6.00 mmol, 1.00 eq) was dissolved in CH_2Cl_2 (20.0 mL, 0.3 M) and MeOTf (1.31 mL, 12.0 mmol, 2.00 eq) was added *via* syringe in one portion at 25 °C. After stirring for two and a half hours at the same temperature the solution was cooled to 0 °C. A solution of $NaBH_4$ (454 mg, 12.0 mmol, 2.00 eq) in THF:MeOH (20.0 mL, 4:1 v:v, 0.3 M) was added *via* syringe within 20 minutes and stirring was continued at 0 °C for another two and a half hours. Saturated NH_4Cl solution (20 mL), water (20 mL) and CH_2Cl_2 (40 mL) were added and the phases separated. The aqueous layer was extracted with CH_2Cl_2 (3 × 50 mL) and the combined organic phases were dried over anhydrous Na_2SO_4. After filtration and solvent evaporation under reduced pressure, the residue was redissolved in THF:H_2O (20.0 mL, 4:1 v:v, 0.3 M). Oxalic acid dihydrate (1.51 g, 12.0 mmol, 2.00 eq) was added at 25 °C and the mixture was stirred for 18 hours at the same temperature. Saturated $NaHCO_3$ solution (20 mL), water (20 mL) and CH_2Cl_2 (40 mL) were added and the phases separated. The aqueous layer was extracted with CH_2Cl_2 (4 × 40 mL) and the combined organic phases were dried over anhydrous Na_2SO_4. Filtration and solvent evaporation under reduced pressure and purification by flash column chromatography (SiO_2, CH:EE 20:1 v:v) afforded **3** (1.12 g, 4.67 mmol, 78 %) as an off-white solid.

R_f = 0.24 (SiO_2, CH:EE 20:1 v:v). **Mp.:** 47 – 49 °C. **^{1}H-NMR (360 MHz, $CDCl_3$):** δ = 10.07 (d, $^5J_{HH}$ = 0.1 Hz 1H, C*H*O), 7.76 – 7.73 (m, 1H, H–3), 7.73 – 7.66 (m, 2H, H–5, H–6), 4.88 (s, 2H, C*H*$_2$) ppm. **^{13}C{^{1}H}-NMR (91 MHz, $CDCl_3$):** δ = 192.0 (C–7), 139.3 (C–2), 135.9 (C–6), 132.4 (C–3), 132.1 (C–1), 131.8 (C–5), 129.8 (C–4), 51.7 (C–8) ppm. **IR (ATR, neat):** $\tilde{\nu}$ = 3098 (w), 2928 (w), 2849 (w), 2751 (w), 2114 (m), 1687 (m), 1593 (m), 1556 (m), 1426 (m), 1378 (w), 1338 (m), 1292 (m), 1223 (w), 1197 (m), 1086 (m), 979 (w), 933 (m), 885 (m), 872 (m), 828 (w), 802 (s), 701 (w), 688 (m) cm^{-1}. **MS (ESI):** m/z = 262.1 $[C_8H_6{}^{79}BrN_3O+Na]^+$. **HR-MS (ESI):** calculated for $C_8H_6{}^{79}BrN_3O^+$ $[M+Na]^+$: m/z

=261.95864, found: 261.95890 (Dev.: 0.26 mu; 0.97 ppm). The analytical data are in accordance with the literature.[14]

2.3 Preparation of 4-(Azidomethyl)-2-bromobenzaldehyde (4)

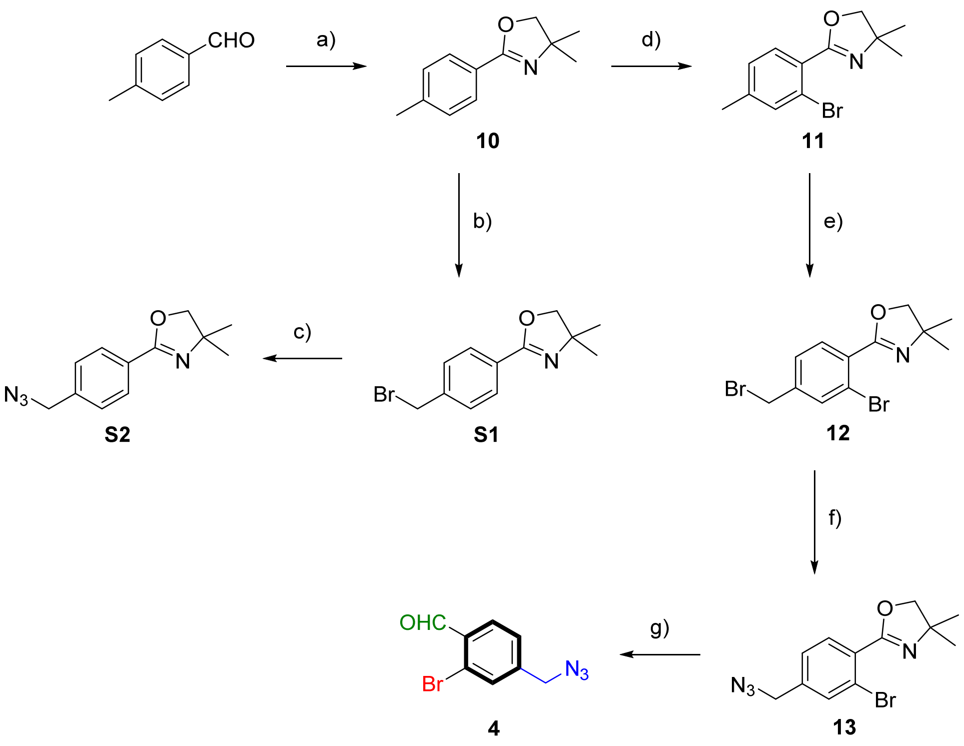

Reaction conditions: a) 1) 2-amino-2-methylpropan-1-ol, 4 Å MS, CH_2Cl_2, 25 °C, 18 h; 2) NBS, CH_2Cl_2, 25 °C, 4 h, 98 %; b) NBS, AIBN, CCl_4, 100 °C, 5 h, 78 %; c) NaN_3, DMF, 25 °C, 4 h, 90 %; d) 1) TMPMgCl·LiCl, THF, 25 °C, 4 h; 2) $(CBrCl_2)_2$, THF, 0 °C to 25 °C, 10 h, 76 %; e) NBS, AIBN, CCl_4, 100 °C, 7 h, 66 %; f) NaN_3, DMF, 25 °C, 4 h, 99 %; g) 1) MeOTf, CH_2Cl_2, 25 °C, 2.5 h; 2) $NaBH_4$, THF:MeOH 4:1 v:v, 0 °C, 2.5 h; 3) oxalic acid, THF:H_2O 4:1 v:v, 25 °C, 20 h, 85 %. Overall yield: 41 % (5 steps).

2.3.1 4,4-Dimethyl-2-(*p*-tolyl)-4,5-dihydrooxazole (10)

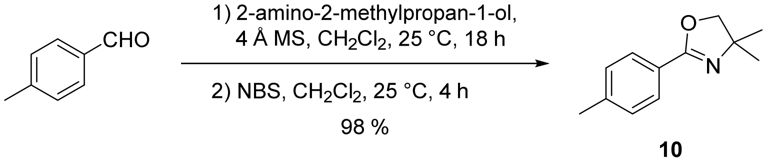

A modified literature procedure was used.[8] *p*-Tolualdehyde (8.41 g, 70.0 mmol, 1.00 eq) and 2-amino-2-methylpropan-1-ol (12.5 g, 140 mmol, 2.00 eq) were dissolved in CH_2Cl_2 (175 mL, 0.4 M) and 4 Å MS (25.0 g) was added. After 18 hours of slowly stirring at 25 °C NBS (24.9 g, 140.0 mmol, 2.00 eq) was added in one portion and stirring was continued for another four hours at 25 °C. All solids were filtered off and the organic phase was washed with saturated $NaHCO_3$ solution (3 × 100 mL). The combined aqueous phases were extracted with CH_2Cl_2 (2 × 100 mL). All organic phases were washed with saturated NaS_2O_3 solution (100 mL) and the aqueous phase was extracted with CH_2Cl_2 (50 mL).

The combined organic phases were dried over anhydrous $MgSO_4$, filtered and the solvent was removed under reduced pressure. Purification by flash column chromatography (SiO_2, CH:EE 10:1 to 5:1 v:v) afforded **10** (13.0 g, 68.7 mmol, 98 %) as a pale brown solid with a characteristic nougat odor.

R*f* = 0.16 (SiO_2, CH:EE 10:1 v:v). **Mp.:** 42 – 44°C (Lit.[15]: 45 °C). **^{1}H-NMR (360 MHz, $CDCl_3$):** δ = 7.81 (d, $^3J_{HH}$ = 7.7 Hz, 2H), 7.19 (d, $^3J_{HH}$ = 7.8 Hz, 2H), 4.07 (s, 2H), 2.37 (s, 3H), 1.36 (s, 6H) ppm. **^{13}C{^{1}H}-NMR (91 MHz, $CDCl_3$):** δ = 162.2, 141.6, 129.1 (2x), 128.3 (2x), 125.3, 79.1, 67.6, 28.5 (2x), 21.6 ppm. **IR (ATR, neat):** $\tilde{\nu}$ = 2970 (w), 2924 (w), 2894 (w), 1642 (m), 1510 (m), 1463 (w), 1409 (w), 1364 (w), 1353 (m), 1316 (m), 1302 (m), 1250 (w), 1193 (w), 1173 (m), 1065 (s), 1019 (m), 992 (w), 965 (m), 917 (m), 871 (w), 829 (s), 727 (s), 682 (s) cm^{-1}. **MS (APCI):** m/z = 190.1 $[M+H]^+$. The analytical data are in accordance with the literature.[16]

2.3.2 2-(4-(Bromomethyl)phenyl)-4,4-dimethyl-4,5-dihydrooxazole (S1)

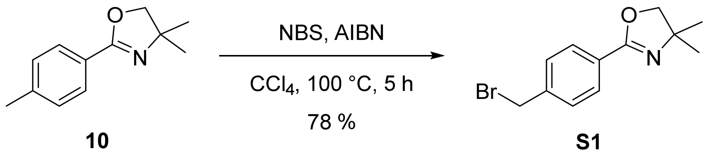

A modified literature procedure was used.[17] **10** (568 mg, 3.00 mmol, 1.00 eq) was dissolved in CCl_4 (10 mL, 0.3 M) and NBS (534 mg, 3.00 mmol, 1.00 eq) was added in one portion. AIBN (24.3 mg, 150 µmol, 0.05 eq) was added and the mixture was heated to 100 °C (oil bath temperature) for five hours. After cooling to 25 °C the mixture was directly filtered over anhydrous Na_2SO_4 and the solids were washed with CCl_4 (20 mL). The solvent was evaporated under reduced pressure and purification was conducted by flash column chromatography (SiO_2, CH:EE 10:1 v:v) to provide **S1** (631 mg, 2.35 mmol, 78 %) as a colorless solid.

R*f* = 0.11 (SiO_2, CH:EE 10:1 v:v). **Mp.:** 77 – 79 °C (Lit.[18]: 77 – 78.5 °C). **^{1}H-NMR (360 MHz, $CDCl_3$):** δ = 7.94 – 7.87 (AA'XX', 2H), 7.45 – 7.39 (AA'XX', 2H), 4.49 (s, 2H), 4.11 (s, 2H), 1.38 (s, 6H) ppm. **^{13}C{^{1}H}-NMR (91 MHz, $CDCl_3$):** δ = 161.6, 140.9, 129.1 (2x), 128.8 (2x), 128.2, 79.3, 67.8, 32.8, 28.6 (2x) ppm. **IR (ATR, neat):** $\tilde{\nu}$ = 2965 (w), 2926 (w), 2898 (w), 1644 (m), 1511 (w), 1458 (w), 1415 (m), 1357 (m), 1320 (m), 1299 (m), 1228 (w), 1194 (w), 1177 (m), 1068 (s), 1018 (m), 987 (w), 960 (m), 918 (w), 870 (w), 849 (m), 835 (m), 819 (m), 747 (w), 688 (s) cm^{-1}. **MS (APCI):** m/z = 267.9 $[C_{12}H_{14}{}^{79}BrNO+H]^+$. **HR-MS (EI, 70 eV):** calculated for $C_{12}H_{14}{}^{79}BrNO^{+\cdot}$ $[M]^{+\cdot}$: m/z = 267.02533, found: 267.02514 (Dev.: –0.19 mu; –0.70 ppm). The analytical data are in accordance with the literature.[18]

2.3.3 2-(4-(Azidomethyl)phenyl)-4,4-dimethyl-4,5-dihydrooxazole (S2)

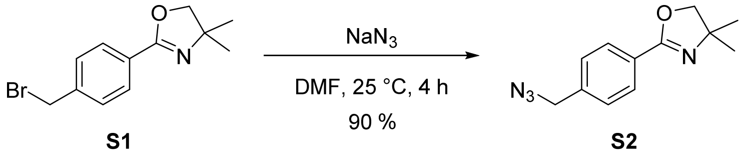

A modified literature procedure was used.[14] **S1** (992 mg, 3.70 mmol, 1.00 eq) was dissolved in DMF (12.3 mL, 0.3 M) and NaN_3 (481 mg, 7.40 mmol, 2.00 eq) was added in one portion. After stirring for four hours at 25 °C water (20 mL) and CH_2Cl_2 (50 mL) were added and the phases separated. The aqueous phase was extracted with CH_2Cl_2 (6 × 50 mL) and the combined organic phases were dried over anhydrous Na_2SO_4, filtered and the solvent was removed under reduced pressure. Purification by flash column chromatography (SiO_2, CH:EE 10:1 to 5:1 v:v) provided **S2** (771 mg, 3.35 mmol, 90 %) as a colorless liquid.

R_f = 0.09 (SiO_2, CH:EE 10:1 v:v). **R_f** = 0.23 (SiO_2, CH:EE 5:1 v:v). **^{1}H-NMR (360 MHz, $CDCl_3$):** δ = 7.98 – 7.92 (AA'XX', 2H), 7.39 – 7.32 (AA'XX', 2H), 4.38 (s, 2H), 4.11 (s, 2H), 1.38 (s, 6H) ppm. **$^{13}C\{^1H\}$-NMR (91 MHz, $CDCl_3$):** δ = 161.3, 138.2, 128.5 (2x), 127.8, 127.7 (2x), 78.9, 67.4, 54.1, 28.2 (2x) ppm. **IR (ATR, neat):** $\tilde{\nu}$ = 2967 (w), 2928 (w), 2893 (w), 2094 (s), 1647 (s), 1513 (w), 1462 (w), 1417 (m), 1352 (m), 1316 (m), 1297 (m), 1247 (m), 1179 (m), 1064 (s), 1019 (m), 991 (w), 965 (m), 920 (w), 844 (m), 802 (m), 730 (m), 693 (m) cm^{-1}. **MS (APCI):** m/z = 231.0 $[C_{12}H_{14}N_4O+H]^+$. **HR-MS (EI, 70 eV):** calculated for $C_{12}H_{14}N_4O^{+\cdot}$ $[M]^{+\cdot}$: m/z = 230.11621, found: 230.11607 (Dev.: –0.14 mu; –0.62 ppm).

2.3.4 2-(2-Bromo-4-methylphenyl)-4,4-dimethyl-4,5-dihydrooxazole (11)

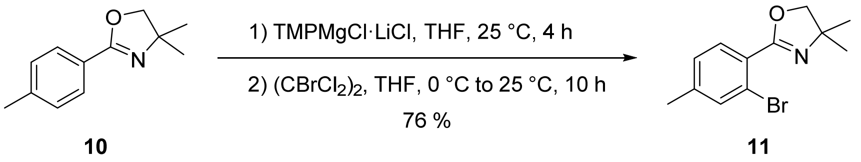

A modified literature procedure was used.[10] A heat gun-dried and nitrogen-flushed *Schlenk* flask, equipped with a magnetic stirring bar and a rubber septum was charged with **11** (1.51 g, 8.00 mmol, 1.00 eq) and evacuated for half an hour. The flask was flushed with nitrogen and anhydrous THF (20.0 mL, 0.4 M) was added. Addition of TMPMgCl·LiCl (24.0 mL, 24.0 mmol, 3.00 eq, 1.00 M in THF) *via* syringe within two minutes at 25 °C was followed by stirring for four hours at the same conditions. 1,2-Dibromotetrachloroethane (7.82 g, 24.0 mmol, 3.00 eq) was diluted in anhydrous THF (12 mL) and added *via* syringe pump (0.5 mL/min) to the dark red solution at 0 °C. After complete addition the mixture was allowed to warm to 25 °C and stirred for a total of ten hours. Saturated NH_4Cl solution (20 mL), water (50 mL) and CH_2Cl_2 (50 mL) were added and the phases separated. The aqueous phase was extracted with CH_2Cl_2 (3 × 50 mL) and the combined organic phases were dried over

anhydrous Na_2SO_4. Filtration, solvent evaporation under reduced pressure and purification by flash column chromatography (SiO_2, CH:EE 10:1 v:v) afforded **11** (1.62 g, 6.04 mmol, 76 %) as a slightly yellow liquid.

R$_f$ = 0.15 (SiO_2, CH:EE 10:1 v:v). **^{1}H-NMR (360 MHz, CDCl$_3$):** δ = 7.54 (d, $^3J_{HH}$ = 7.8 Hz, 1H), 7.44 (d, $^4J_{HH}$ = 0.6 Hz, 1H), 7.12 (dd, $^3J_{HH}$ = 7.9 Hz, $^4J_{HH}$ = 0.8 Hz, 1H), 4.11 (s, 2H), 2.34 (s, 3H), 1.40 (s, 6H) ppm. **^{13}C{^{1}H}-NMR (91 MHz, CDCl$_3$):** δ = 161.5, 141.9, 133.9, 130.8, 127.6, 127.0, 121.3, 79.0, 67.7, 28.0, 20.8 ppm. **IR (ATR, neat):** $\tilde{\nu}$ = 2965 (w), 2926 (w), 2889 (w), 1651 (m), 1604 (w), 1493 (w), 1460 (w), 1390 (w), 1363 (w), 1349 (m), 1308 (m), 1264 (w), 1248 (w), 1188 (w), 1084 (s), 1027 (s), 963 (m), 920 (w), 877 (w), 827 (m), 812 (m), 736 (m), 682 (w), 671 (m) cm^{-1}. **MS (APCI):** m/z = 267.9 [$C_{12}H_{14}{}^{79}BrNO+H$]$^+$. **HR-MS (EI, 70 eV):** calculated for $C_{12}H_{14}{}^{79}BrNO^{+\cdot}$ [M]$^{+\cdot}$: m/z = 267.02533, found: 267.02509 (Dev.: –0.24 mu; –0.89 ppm). The analytical data are in accordance with the literature.[19]

2.3.5 2-(2-Bromo-4-(bromomethyl)phenyl)-4,4-dimethyl-4,5-dihydrooxazole (12)

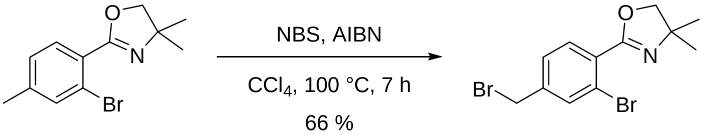

11 **12**

A modified literature procedure was used.[17] **11** (536 mg, 2.00 mmol, 1.00 eq) was dissolved in CCl_4 (6.67 mL, 0.3 M) and NBS (356 mg, 2.00 mmol, 1.00 eq) was added in one portion. AIBN (16.4 mg, 100 µmol, 0.05 eq) was added and the mixture was heated to 100 °C for seven hours. After cooling to 25 °C the mixture was directly filtered over anhydrous Na_2SO_4 and the solids were washed with CCl_4 (30 mL). The solvent was evaporated under reduced pressure and purification was conducted by flash column chromatography (SiO_2, CH:EE 10:1 v:v) to provide **12** (457 mg, 1.32 mmol, 66 %) as a colorless liquid. Upon standing the product slowly solidified to form a colorless solid.

R$_f$ = 0.11 (SiO_2, CH:EE 10:1 v:v). **Mp.:** 70 – 72 °C. **^{1}H-NMR (360 MHz, CDCl$_3$):** δ = 7.65 (d, $^4J_{HH}$ = 1.8 Hz, 1H), 7.63 (d, $^3J_{HH}$ = 7.9 Hz, 1H), 7.34 (dd, $^3J_{HH}$ = 7.9 Hz, $^4J_{HH}$ = 1.8 Hz, 1H), 4.41 (s, 2H), 4.13 (s, 2H), 1.40 (s, 6H) ppm. **^{13}C{^{1}H}-NMR (91 MHz, CDCl$_3$):** δ = 161.3, 141.5, 134.2, 131.7, 130.2, 127.8, 122.1, 79.5, 68.3, 31.3, 28.4 ppm. **IR (ATR, neat):** $\tilde{\nu}$ = 2969 (w), 2930 (w), 2892 (w), 1652 (m), 1491 (w), 1459 (w), 1397 (w), 1383 (w), 1363 (w), 1346 (m), 1308 (m), 1270 (w), 1216 (m), 1194 (m), 1170 (w), 1083 (s), 1028 (s), 991 (w), 968 (m), 939 (w), 912 (m), 884 (m), 847 (m), 834 (m), 816 (m), 694 (s), 673 (m) cm^{-1}. **MS (APCI):** m/z = 345.9 [$C_{12}H_{13}{}^{79}Br_2NO+H$]$^+$. **HR-MS (EI, 70 eV):** calculated for $C_{12}H_{13}{}^{79}Br_2NO^{+\cdot}$ [M]$^{+\cdot}$: m/z = 344.93584, found: 344.93583 (Dev.: –0.01 mu; –0.03 ppm).

2.3.6 2-(4-(Azidomethyl)-2-bromophenyl)-4,4-dimethyl-4,5-dihydrooxazole (13)

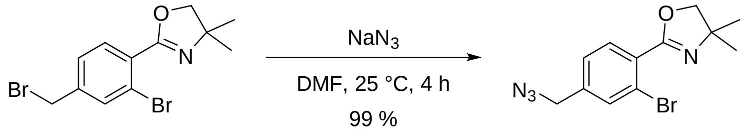

12 **13**

A modified literature procedure was used.[14] **12** (521 mg, 1.50 mmol, 1.00 eq) was dissolved in DMF (5.00 mL, 0.3 M) and NaN_3 (195 mg, 3.00 mmol, 2.00 eq) was added in one portion. After stirring for four hours at 25 °C water (20 mL) and CH_2Cl_2 (50 mL) were added and the phases separated. The aqueous phase was extracted with CH_2Cl_2 (6 × 50 mL) and the combined organic phases were dried over anhydrous Na_2SO_4, filtered and the solvent was removed under reduced pressure. Purification by flash column chromatography (SiO_2, CH:EE 10:1 to 5:1 v:v) provided **13** (457 mg, 1.48 mmol, 99 %) as a colorless liquid.

R$_f$ = 0.10 (SiO_2, CH:EE 10:1 v:v). **R$_f$** = 0.24 (SiO_2, CH:EE 5:1 v:v). **^{1}H-NMR (360 MHz, $CDCl_3$):** δ = 7.67 (d, $^3J_{HH}$ = 7.9 Hz, 1H), 7.59 (d, $^4J_{HH}$ = 1.7 Hz, 1H), 7.28 (dd, $^3J_{HH}$ = 7.9 Hz, $^4J_{HH}$ = 1.7 Hz, 1H), 4.36 (s, 2H), 4.14 (s, 2H), 1.41 (s, 6H) ppm. **^{13}C{^{1}H}-NMR (91 MHz, $CDCl_3$):** δ = 161.4, 139.4, 133.1, 131.8, 130.2, 126.6, 122.3, 79.6, 68.3, 53.7, 28.4 ppm. **IR (ATR, neat):** $\tilde{\nu}$ = 2966 (w), 2928 (w), 2891 (w), 2095 (s), 1652 (m), 1604 (w), 1492 (w), 1461 (w), 1397 (m), 1350 (m), 1338 (m), 1308 (m), 1280 (m), 1247 (m), 1189 (m), 1086 (s), 1028 (s), 961 (m), 920 (m), 888 (m), 826 (m), 813 (m), 738 (w), 686 (m) cm^{-1}. **MS (APCI):** m/z = 309.0 [$C_{12}H_{13}{}^{79}BrN_4O$+H]$^+$. **HR-MS (EI, 70 eV):** calculated for $C_{12}H_{13}{}^{79}BrN_4O^{+\cdot}$ [M]$^{+\cdot}$: m/z = 308.02672, found: 308.02707 (Dev.: 0.35 mu; 1.12 ppm).

2.3.7 4-(Azidomethyl)-2-bromobenzaldehyde (4)

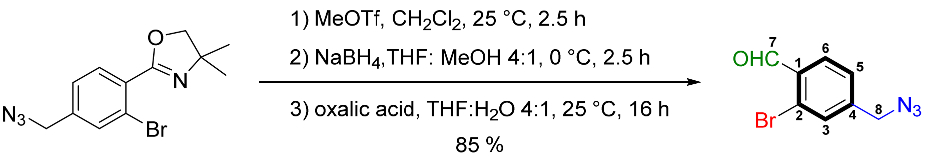

13 **4**

A modified literature procedure was used.[13] **13** (928 mg, 3.00 mmol, 1.00 eq) was dissolved in CH_2Cl_2 (10.0 mL, 0.3 M) and MeOTf (656 µL, 6.00 mmol, 2.00 eq) was added *via* syringe in one portion at 25 °C. After stirring for two and a half hours at the same temperature the solution was cooled to 0 °C. A solution of $NaBH_4$ (227 mg, 6.00 mmol, 2.00 eq) in THF:MeOH (10.0 mL, 4:1 v:v, 0.3 M) was added *via* syringe within five minutes and stirring was continued at 0 °C for another two and a half hours. Saturated NH_4Cl solution (10 mL), water (20 mL) and CH_2Cl_2 (20 mL) were added and the phases separated. The aqueous layer was extracted with CH_2Cl_2 (4 × 20 mL) and the combined organic phases were dried over anhydrous Na_2SO_4. After filtration and solvent evaporation under reduced pressure, the residue was redissolved in THF:H_2O (10.0 mL, 4:1 v:v, 0.3 M). Oxalic acid dihydrate (756 mg, 6.00 mmol, 2.00 eq) was added at 25 °C and the mixture was stirred for 16 hours at the same

temperature. Saturated $NaHCO_3$ solution (10 mL), water (20 mL) and CH_2Cl_2 (40 mL) were added and the phases separated. The aqueous layer was extracted with CH_2Cl_2 (4 × 20 mL) and the combined organic phases were dried over anhydrous Na_2SO_4, filtered and the solvent was evaporated under reduced pressure. Purification by flash column chromatography (SiO_2, CH:EE 5:1 v:v) afforded **4** (614 mg, 2.56 mmol, 85 %) as a colorless oil which solidified very slowly upon standing at 25 °C.

R_f = 0.54 (SiO_2, CH:EE 5:1 v:v). **Mp.:** 26 – 28 °C. **^{1}H-NMR (360 MHz, $CDCl_3$):** δ = 10.34 (d, $^4J_{HH}$ = 0.8 Hz, 1H, C*H*O), 7.92 (d, $^3J_{HH}$ = 8.0 Hz, 1H, H–6), 7.63 (dd, $^4J_{HH}$ = 1.6 Hz, $^5J_{HH}$ = 0.8 Hz, 1H, H–3), 7.38 (ddd, $^3J_{HH}$ = 7.9 Hz, $^4J_{HH}$ = 1.6 Hz, $^5J_{HH}$ = 0.8 Hz, 1H, H–5), 4.43 (s, 2H, CH_2) ppm. **^{13}C{^{1}H}-NMR (91 MHz, $CDCl_3$):** δ = 191.0 (C–7), 143.2 (C–4), 132.9 (C–1), 132.7 (C–3), 130.0 (C–6), 127.2 (C–2), 126.9 (C–5), 53.3 (C–8) ppm. **IR (ATR, neat):** $\tilde{\nu}$ = 3084 (w), 2920 (w), 2859 (w), 2758 (w), 2227 (w), 2116 (s), 1684 (s), 1599 (s), 1558 (m), 1483 (w), 1427 (m), 1403 (m), 1387 (m), 1339 (s), 1275 (s), 1207 (s), 1140 (m), 1038 (m), 966 (m), 957 (m), 881 (m), 872 (m), 821 (s), 797 (s), 733 (w), 681 (m) cm^{-1}. **MS (EI, 70 eV):** m/z = 238.8 $[C_8H_6{}^{79}BrN_3O]^{+\cdot}$. **HR-MS (EI, 70 eV):** calculated for $C_8H_6{}^{79}BrN_3O^{+\cdot}$ $[M]^{+\cdot}$: m/z = 238.96888, found: 238.96872 (Dev.: –0.16 mu; –0.65 ppm). The analytical data are in accordance with the literature.[20]

2.4 Preparation of Highly Functionalized Fluorenes (5), (23), (25) & (28)

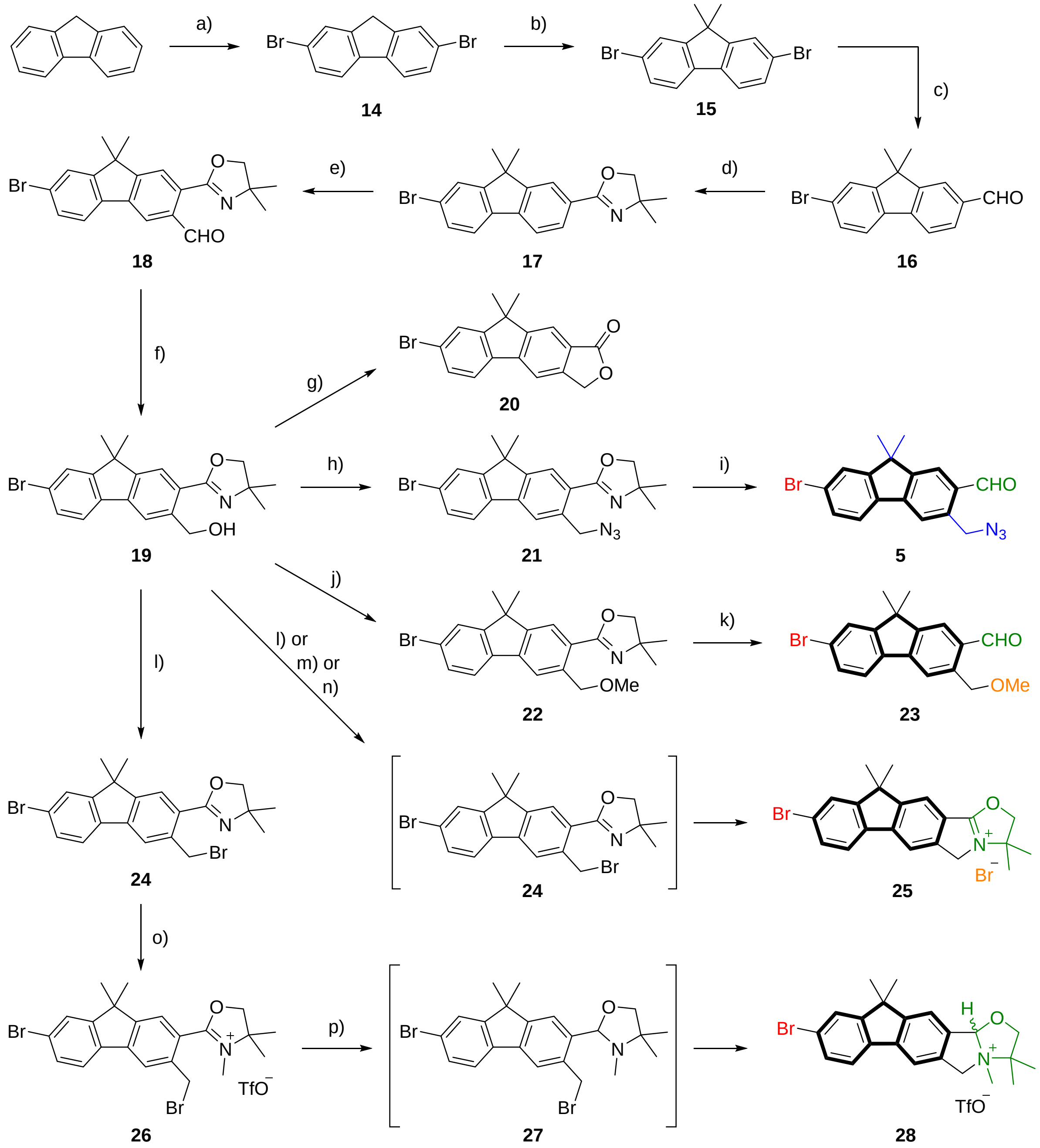

Reaction conditions: a) Br_2, Fe-powder, $CHCl_3$, 0 °C, 4 h, 99 %; b) KOH, KI, MeI, DMSO, 25 °C, 18 h, 92 %; c) 1) *n*BuLi, THF, –78 °C, 1 h; 2) DMF, THF, –78 °C to 25 °C, 10 h, 90 %; d) 1) 2-amino-2-methylpropan-1-ol, 4 Å MS, CH_2Cl_2, 25 °C, 18 h; 2) NBS, CH_2Cl_2, 25 °C, 3 h, 92 %; e) 1) TMPMgCl·LiCl, THF, 25 °C, 4 h; 2) DMF, THF, –15 °C to 25 °C, 2 h; f) $NaBH_4$, THF:MeOH 1:1 v:v, 0 °C, 1.5 h, 79 % (2 steps); g) HCl (4 N), 120 °C, 6 h, 91 %; h) 1) DPPA, DBU, PhMe, 25 °C, 15 h; 2) NaN_3, PhMe, 60 °C, 4 h, 87 %; i) 1) MeOTf, CH_2Cl_2, 25 °C, 2.5 h; 2) $NaBH_4$, THF:MeOH 4:1 v:v, 0 °C, 2.5 h; 3) oxalic acid, THF:H_2O 4:1 v:v, 25 °C, 20 h, 86 % – overall yield to **5**: 45 % (8 steps); j) 1) NaH, THF, 0 °C, 30 min; 2) MeI, THF, 0 °C to 25 °C, 2 h, 99 %; k) 1) MeOTf, CH_2Cl_2, 25 °C, 3 h; 2) $NaBH_4$, THF:MeOH 4:1 v:v, 0 °C, 3 h; 3) oxalic acid, THF:H_2O 4:1 v:v, 25 °C, 24 h, 75 % – overall yield to **23**: 44 % (8 steps); l) CBr_4, PPh_3, CH_2Cl_2, 0 °C to 25 °C, 2 h, 95 %; m) NBS, PPh_3, CH_2Cl_2, 0 °C

to 25 °C, 2 h, 91 %; n) PBr_3, CH_2Cl_2, 0 °C to 25 °C, 2 h, 92 %; o) MeOTf, CH_2Cl_2, 25 °C, 3 h; p) $NaBH_4$, THF:MeOH 4:1 v:v, 0 °C, 3 h, 56 % from **19**.

2.4.1 2,7-Dibromo-9*H*-fluorene (14)

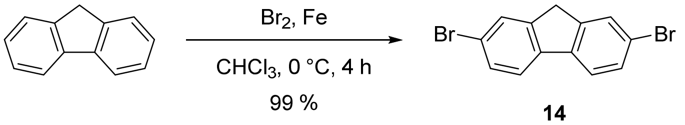

14

A modified literature procedure was used.[21] Fluorene (8.31 g, 50.0 mmol, 1.00 eq) was dissolved in $CHCl_3$ (83 mL, 0.6 M) and Fe-powder (279 mg, 5.00 mmol, 0.10 eq) was added. The solution was cooled to 0 °C in a water/ice bath. Br_2 (5.38 mL, 105 mmol, 2.10 eq) in $CHCl_3$ (42 mL) was added through a dropping funnel over one hour in the dark to the vigorously stirred mixture. After complete addition the mixture was stirred for an additional three hours at 0 °C. Saturated $Na_2S_2O_5$ solution (100 mL) was slowly added at the same temperature and stirring was continued for 30 minutes. $CHCl_3$ (100 mL) was added, the phases were separated and the aqueous layer was extracted with $CHCl_3$ (2 × 100 mL). The combined organic layers were dried over anhydrous Na_2SO_4, filtered and concentrated under reduced pressure on a rotary evaporator. **14** (16.1 g, 49.7 mmol, 99 %) was isolated as a colorless solid. If desired the product can be recrystallized from $CHCl_3$.

$\mathbf{R_f}$ = 0.61 (SiO_2, CH). **Mp.:** 164 – 166 °C (Lit.[22]: 163 – 164 °C). **^{1}H-NMR (360 MHz, $CDCl_3$):** δ = 7.64 (d, $^4J_{HH}$ = 1.6 Hz, 2H), 7.57 (d, $^3J_{HH}$ = 8.1 Hz, 2H), 7.49 (dd, $^3J_{HH}$ = 8.2 Hz, $^4J_{HH}$ = 1.8 Hz, 2H), 3.83 (s, 2H) ppm. **^{13}C{^{1}H}-NMR (91 MHz, $CDCl_3$):** δ = 144.9 (2x), 139.8 (2x), 130.2 (2x), 128.4 (2x), 121.3 (2x), 121.1 (2x), 36.7 ppm. **IR (ATR, neat):** $\tilde{\nu}$ = 3049 (w), 2919 (w), 1881 (w), 1760 (w), 1568 (w), 1453 (w), 1391 (m), 1159 (w), 1054 (m), 1005 (w), 952 (w), 931 (w), 807 (s), 685 (m), 662 (m) cm^{-1}. **MS (EI, 70 eV):** m/z = 321.8 $[C_{13}H_8{}^{79}Br_2]^{+\cdot}$. The analytical data are in accordance with the literature.[23]

2.4.2 2,7-Dibromo-9,9-dimethyl-9*H*-fluorene (15)

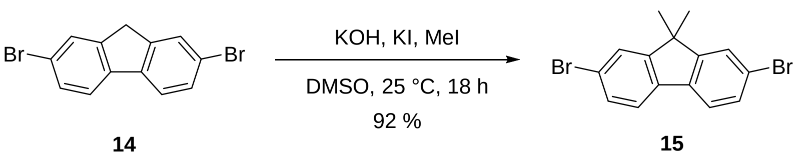

14 **15**

A modified literature procedure was used.[24] **14** (16.2 g, 50.0 mmol, 1.00 eq) was suspended in DMSO (83 mL, 0.6 M) and KI (830 mg, 5.00 mmol, 0.10 eq) was added. To the water bath-cooled and vigorously stirred mixture were added KOH pellets (11.2 g, 200 mmol, 4.00 eq). The reaction was stirred for one hour, while the solution turned intensive red. MeI (7.78 mL, 125 mmol, 2.50 eq) was added *via* syringe pump (0.15 mL/min) through a rubber septum and stirring was continued at 25 °C for 18 hours.

Excess of MeI was quenched by addition of NEt_3 (13.9 mL, 100 mmol, 2.00 eq). The mixture was stirred for 30 minutes, poured into water (500 mL) and extracted with CH_2Cl_2 (4 × 100 mL). The combined organic layers were dried over anhydrous Na_2SO_4, filtered and concentrated under reduced pressure on a rotary evaporator. Purification by flash column chromatography (SiO_2, CH) afforded **15** (16.2 g, 46.0 mmol, 92 %) as a colorless solid. If desired the product can be recrystallized from cyclohexane.

R_f = 0.68 (SiO_2, CH). **Mp.:** 177 – 179 °C (Lit.[22]: 172 – 173 °C). **^{1}H-NMR (360 MHz, $CDCl_3$):** δ = 7.55 (d, $^4J_{HH}$ = 1.8 Hz, 2H), 7.54 (d, $^3J_{HH}$ = 8.1 Hz, 2H), 7.46 (dd, $^3J_{HH}$ = 8.1 Hz, $^4J_{HH}$ = 1.8 Hz, 2H), 1.47 (s, 6H) ppm. **^{13}C{^{1}H}-NMR (91 MHz, $CDCl_3$):** δ = 155.4 (2x), 137.3 (2x), 130.5 (2x), 126.3 (2x), 121.6 (4x), 47.4, 27.0 (2x) ppm. **IR (ATR, neat):** $\tilde{\nu}$ = 2962 (m), 2920 (w), 2856 (w), 1861 (w), 1727 (w), 1597 (w), 1577 (w), 1447 (m), 1397 (m), 1259 (m), 1083 (m), 1058 (m), 1001 (m), 865 (m), 824 (m), 791 (s), 729 (m), 667 (m) cm^{-1}. **MS (EI, 70 eV):** m/z = 349.9 $[C_{15}H_{12}{}^{79}Br_2]^{+\cdot}$. The analytical data are in accordance with the literature.[25]

2.4.3 7-Bromo-9,9-dimethyl-9*H*-fluorene-2-carbaldehyde (16)

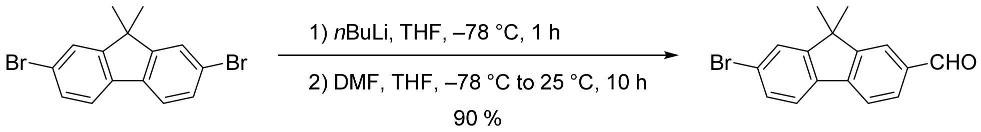

15 **16**

A modified literature procedure was used.[24] A heat gun-dried and nitrogen-flushed *Schlenk* flask was charged with **15** (14.1 g, 40.0 mmol, 1.00 eq) and anhydrous THF (200 mL, 0.2 M) was added. The solution was cooled to –78 °C and *n*BuLi (16.8 mL, 42.0 mmol, 1.05 eq, 2.5 M in hexane) was added *via* syringe pump (0.2 mL/min) through a rubber septum to the vigorously stirred mixture. The intensive red solution was stirred for one hour at –78 °C and anhydrous DMF (5.85 g, 6.19 mL, 80.0 mmol, 2.00 eq) in anhydrous THF (8 mL) was added *via* syringe pump (0.4 mL/min) through a rubber septum. Stirring was continued for 10 hours while the mixture was allowed to warm slowly to 25 °C. HCl (100 mL, 1.0 M) was poured into the reaction in small portions and the mixture was stirred rapidly for 30 minutes. The phases were separated and the aqueous layer was extracted with ethyl acetate (3 × 100 mL). The combined organic layers were dried over anhydrous Na_2SO_4, filtered and concentrated under reduced pressure on a rotary evaporator. Purification by flash column chromatography (SiO_2, CH:EE 20:1 v:v) afforded **16** (10.9 g, 36.0 mmol, 90 %) as a colorless solid.

R_f = 0.26 (SiO_2, CH:EE 20:1 v:v). **Mp.:** 147 – 149 °C. **^{1}H-NMR (360 MHz, $CDCl_3$):** δ = 10.06 (s, 1H), 7.96 (d, $^4J_{HH}$ = 1.5 Hz, 1H), 7.87 (dd, $^3J_{HH}$ = 7.8 Hz, $^4J_{HH}$ = 1.3 Hz, 1H), 7.82 (d, $^3J_{HH}$ = 7.8 Hz, 1H), 7.65 (d, $^3J_{HH}$ = 8.1 Hz, 1H), 7.61 (d, $^4J_{HH}$ = 1.6 Hz, 1H), 7.51 (dd, $^3J_{HH}$ = 8.1 Hz, $^4J_{HH}$ = 1.7 Hz, 1H), 1.52 (s, 6H) ppm. **^{13}C{^{1}H}-NMR (91 MHz, $CDCl_3$):** δ = 192.2, 157.0, 154.1, 144.6, 136.8, 135.9, 130.8, 130.7, 126.6, 123.2, 122.7, 120.5, 47.4, 26.9 (2x) ppm. **IR (ATR, neat):** $\tilde{\nu}$ = 2963 (w), 2924 (w), 2814

(w), 2783 (w), 2708 (w), 1695 (s), 1683 (s), 1605 (m), 1405 (m), 1247 (m), 1175 (s), 1060 (m), 883 (m), 810 (s), 795 (s), 755 (s), 733 (s), 658 (m) cm^{-1}. **MS (APCI):** m/z = 301.1 $[C_{16}H_{13}{}^{79}BrO+H]^+$. The analytical data are in accordance with the literature.[22]

2.4.4 2-(7-Bromo-9,9-dimethyl-9*H*-fluoren-2-yl)-4,4-dimethyl-4,5-dihydrooxazole (17)

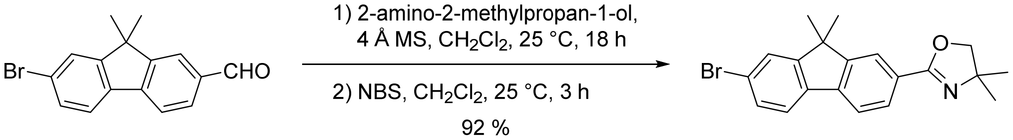

16 **17**

A modified literature procedure was used.[8] **16** (3.01 g, 10.0 mmol, 1.00 eq) and 2-amino-2-methylpropan-1-ol (1.78 g, 1.91 mL, 20.0 mmol, 2.00 eq) were dissolved in CH_2Cl_2 (40 mL, 0.25 M) and 4 Å MS (5.00 g) was added. The mixture was slowly stirred for 18 hours at 25 °C whereupon NBS (3.56 g, 20.0 mmol, 2.00 eq) was added in one portion and stirring was continued for another three hours at the same temperature. All solids were filtered off and the organic phase was washed with saturated $NaHCO_3$ solution (2 × 50 mL). The combined aqueous phases were extracted with CH_2Cl_2 (2 × 50 mL). All organic phases were combined and washed with saturated $Na_2S_2O_3$ solution (50 mL) and the aqueous phase was extracted with CH_2Cl_2 (20 mL). The combined organic phases were dried over anhydrous $MgSO_4$, filtered and concentrated under reduced pressure on a rotary evaporator. Purification by flash column chromatography (SiO_2, CH:EE 15:1 v:v) afforded **17** (3.41 g, 9.21 mmol, 92 %) as a colorless foam.

R_f = 0.17 (SiO_2, CH:EE 15:1 v:v). **Mp.:** 62 – 64 °C. **^{1}H-NMR (360 MHz, $CDCl_3$):** δ = 8.02 (d, $^4J_{HH}$ = 1.4 Hz, 1H), 7.92 (dd, $^3J_{HH}$ = 7.9 Hz, $^4J_{HH}$ = 1.5 Hz, 1H), 7.69 (d, $^3J_{HH}$ = 7.9 Hz, 1H), 7.58 (d, $^3J_{HH}$ = 8.0 Hz, 1H), 7.56 (d, $^4J_{HH}$ = 1.8 Hz, 1H), 7.46 (dd, $^3J_{HH}$ = 8.0 Hz, $^4J_{HH}$ = 1.9 Hz, 1H), 4.12 (s, 2H), 1.48 (s, 6H), 1.41 (s, 6H) ppm. **^{13}C{^{1}H}-NMR (91 MHz, $CDCl_3$):** δ = 162.3, 156.4, 153.3, 141.2, 137.4, 130.4, 127.8, 127.2, 126.4, 122.7, 122.1, 122.0, 119.9, 79.2, 67.7, 47.4, 28.6 (2x), 26.9 (2x) ppm. **IR (ATR, neat):** $\tilde{\nu}$ = 2962 (w), 2924 (w), 2359 (w), 1641 (s), 1452 (m), 1403 (m), 1355 (m), 1308 (s), 1262 (s), 1202 (s), 1086 (m), 1060 (s), 968 (m), 815 (s), 774 (m), 738 (s), 716 (s) cm^{-1}. **MS (APCI):** m/z = 370.2 $[C_{20}H_{20}{}^{79}BrNO+H]^+$. **HR-MS (EI, 70 eV):** calculated for $C_{20}H_{20}{}^{79}BrNO^{+\cdot}$ $[M]^{+\cdot}$: m/z = 369.07228, found: 369.07227 (Dev.: –0.01 mu; –0.02 ppm). The analytical data are in accordance with the literature.[26]

2.4.5 7-Bromo-2-(4,4-dimethyl-4,5-dihydrooxazol-2-yl)-9,9-dimethyl-9*H*-fluorene-3-carbaldehyde (18)

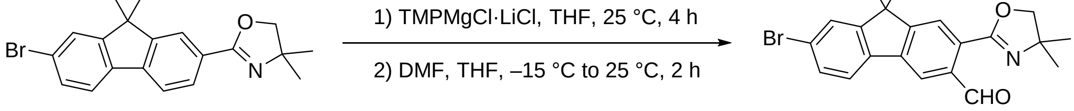

17 **18**

A literature procedure was used.[26] A heat gun-dried and nitrogen-flushed *Schlenk* flask, equipped with a magnetic stirring bar and a rubber septum, was charged with **17** (3.70 g, 10.0 mmol, 1.00 eq) and evacuated for half an hour. The flask was flushed with nitrogen and anhydrous THF (25.0 mL, 0.4 M) was added. Addition of TMPMgCl·LiCl (25.0 mL, 30.0 mmol, 3.00 eq, 1.20 M) *via* syringe through the rubber septum within five minutes at 25 °C was followed by stirring for four hours under the same conditions. DMF (3.10 mL, 40.0 mmol, 4.00 eq) was diluted in anhydrous THF (10 mL) and added *via* syringe pump (0.3 mL/min) through the rubber septum to the dark red solution at –15 °C. After complete addition the brown mixture was stirred for another two hours while it was allowed to warm to 25 °C. Full consumption of the starting material was followed by diluting with water (50 mL) and ethyl acetate (80 mL). The phases were separated and the aqueous layer was extracted with ethyl acetate (4 × 80 mL). The combined organic layers were dried over anhydrous Na_2SO_4, filtered and concentrated under reduced pressure on a rotary evaporator (30 °C water bath temperature). The product was used without further purification for the next reaction step. If desired the aldehyde **18** can be isolated as a colorless solid by washing the crude product with small amounts of ice cooled cyclohexane.

R_f = 0.33 (SiO_2, CH:EE 6:1 v:v). **Mp.:** 178 – 180 °C decomp. **^{1}H-NMR (360 MHz, $CDCl_3$):** δ = 10.75 (s, 1H), 8.26 (s, 1H), 7.93 (s, 1H), 7.67 (d, $^3J_{HH}$ = 8.1 Hz, 1H), 7.58 (s, 1H), 7.51 (d, $^3J_{HH}$ = 8.0 Hz, 1H), 4.19 (s, 2H), 1.51 (s, 6H), 1.44 (s, 6H) ppm. **^{13}C{^{1}H}-NMR (91 MHz, $CDCl_3$):** δ = 192.5, 160.7, 158.1, 156.1, 141.2, 136.5, 136.1, 130.8, 129.5, 126.5, 124.4, 122.9, 122.6, 119.7, 79.5, 68.9, 47.8, 28.6 (2x), 26.8 (2x) ppm. **IR (ATR, neat):** $\tilde{\nu}$ = 2966 (w), 2926 (w), 2359 (w), 2324 (w), 1682 (s), 1643 (m), 1613 (m), 1463 (m), 1409 (m), 1358 (m), 1302 (m), 1268 (m), 1200 (m), 1146 (m), 1086 (m), 1062 (s), 1038 (s), 973 (s), 899 (s), 810 (s), 743 (s) cm^{-1}. **MS (APCI):** m/z = 398.1 $[C_{21}H_{20}{}^{79}BrNO_2+H]^+$. **HR-MS (EI, 70 eV):** calculated for $C_{21}H_{20}{}^{79}BrNO_2^{+\cdot}$ $[M]^{+\cdot}$: m/z = 397.06719, found: 397.06645 (Dev.: –0.74 mu; –1.87 ppm). The analytical data are in accordance with the literature.[26]

2.4.6 (7-Bromo-2-(4,4-dimethyl-4,5-dihydrooxazol-2-yl)-9,9-dimethyl-9*H*-fluoren-3-yl)methanol (19)

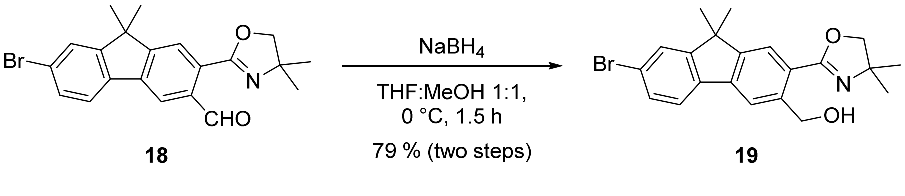

18 **19**

A modified literature procedure was used.[11] The crude aldehyde **18** (10.0 mmol, 1.00 eq) was dissolved in THF:MeOH (25.0 mL, 1:1 v:v, 0.4 M) and cooled to 0 °C. $NaBH_4$ (757 mg, 20.0 mmol, 2.00 eq) was added at once and stirring was continued for 90 minutes while the temperature was maintained. Water (25 mL) was added dropwise at 0 °C to stop the reaction, CH_2Cl_2 (50 mL) was added and the phases were separated. The aqueous layer was extracted with CH_2Cl_2 (4 × 50 mL) and the combined organic layers were dried over anhydrous Na_2SO_4, filtered and concentrated under reduced

pressure. Purification by flash column chromatography (SiO_2, CH:EE 10:1 to 6:1 v:v) afforded **19** (3.16 g, 7.89 mmol, 79 % over two steps) as a colorless solid. If necessary, the product can be washed with cyclohexane to remove yellow impurities. $CDCl_3$, used for NMR analysis, must be free of acids, due to decomposition of **19** in acidic media. Therefore, $CDCl_3$ was flushed over neutral alumina.

R*f* = 0.06 (SiO_2, CH:EE 10:1 v:v). **R*f*** = 0.22 (SiO_2, CH:EE 6:1 v:v). **Mp.:** 179 – 181 °C. **^{1}H-NMR (360 MHz, $CDCl_3$):** δ = 7.91 (s, 1H), 7.68 (s, 1H), 7.61 (d, $^3J_{HH}$ = 8.0 Hz, 1H), 7.57 (d, $^4J_{HH}$ = 1.8 Hz, 1H), 7.49 (dd, $^3J_{HH}$ = 8.0 Hz, $^4J_{HH}$ = 1.8 Hz, 1H), 6.77 (br. s, 1H), 4.74 (s, 2H), 4.17 (s, 2H), 1.48 (s, 6H), 1.43 (s, 6H) ppm. **^{13}C{^{1}H}-NMR (91 MHz, $CDCl_3$):** δ = 162.4, 156.7, 152.5, 141.9, 141.4, 137.2, 130.6, 126.4, 126.0, 124.4, 122.4, 122.2 (2x), 78.9, 68.2, 64.9, 47.3, 28.6 (2x), 26.9 (2x) ppm. **IR (ATR, neat):** $\tilde{\nu}$ = 3184 (br), 3050 (w), 2962 (w), 2947 (w), 2926 (w), 2864 (w), 2360 (w), 1635 (s), 1567 (w), 1459 (m), 1366 (m), 1307 (m), 1298 (m), 1204 (s), 1089 (m), 1064 (s), 1050 (s), 1019 (s), 974 (m), 950 (s), 899 (m), 885 (m), 830 (s), 819 (s), 740 (s), 724 (s), 654 (s) cm^{-1}. **MS (APCI):** m/z = 400.2 [$C_{21}H_{22}{}^{79}BrNO_2$+H]$^+$. **HR-MS (EI, 70 eV):** calculated for $C_{21}H_{22}{}^{79}BrNO_2^{+\cdot}$ [M]$^{+\cdot}$: m/z = 399.08284, found: 399.08234 (Dev.: –0.50 mu; –1.25 ppm). The analytical data are in accordance with the literature.[26]

2.4.7 7-Bromo-9,9-dimethyl-3,9-dihydro-1*H*-fluoreno[2,3-*c*]furan-1-one (20)

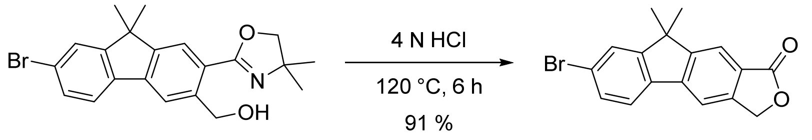

19 **20**

A modified literature procedure was used.[27] **19** (160 mg, 400 µmol, 1.00 eq) was suspended in 4 N HCl (2.00 mL, 0.4 M) and heated to 120 °C (oil bath temperature). After stirring for six hours at this temperature the mixture was allowed to cool to 25 °C and saturated $NaHCO_3$ solution (10 mL) and CH_2Cl_2 (10 mL) were added. The phases were separated and the aqueous phase was extracted with CH_2Cl_2 (3 × 10 mL). The combined organic phases were washed with saturated $NaHCO_3$ solution (10 mL), dried over anhydrous Na_2SO_4, filtered and concentrated under reduced pressure. Purification by flash column chromatography (SiO_2, CH:EE 6:1 v:v) afforded **20** (120 mg, 365 µmol, 91 %) as a colorless solid.

R*f* = 0.22 (SiO_2, CH:EE 6:1 v:v). **Mp.:** 215 – 217 °C. **^{1}H-NMR (360 MHz, $CDCl_3$):** δ = 7.94 (d, $^5J_{HH}$ = 0.9 Hz, 1H), 7.75 (d, $^5J_{HH}$ = 0.8 Hz, 1H), 7.65 (d, $^3J_{HH}$ = 8.1 Hz, 1H), 7.62 (d, $^4J_{HH}$ = 1.8 Hz, 1H), 7.52 (dd, $^3J_{HH}$ = 8.1 Hz, $^4J_{HH}$ = 1.8 Hz, 1H), 5.37 (s, 2H), 1.51 (s, 6H) ppm. **^{13}C{^{1}H}-NMR (91 MHz, $CDCl_3$):** δ = 171.4, 156.9, 154.9, 146.7, 144.9, 136.3, 130.8, 126.8, 125.0, 123.5, 122.5, 120.1, 113.4, 69.8, 47.1, 27.1 (2x) ppm. **IR (ATR, neat):** $\tilde{\nu}$ = 2910 (w), 2869 (w), 2360 (w), 1755 (s), 1622 (m), 1452 (m), 1345 (s), 1252 (m), 1184 (s), 1081 (m), 1055 (m), 1034 (s), 1006 (s), 953 (w), 884 (m), 870 (m), 822 (s), 777

(s), 762 (s) cm^{-1}. **MS (APCI):** m/z = 329.1 [$C_{17}H_{13}{}^{79}BrO_2$+H]$^+$. **HR-MS (EI, 70 eV):** calculated for $C_{17}H_{13}{}^{79}BrO_2{}^{+\cdot}$ [M]$^{+\cdot}$: m/z = 328.00934, found: 328.00958 (Dev.: 0.24 mu; 0.72 ppm). The analytical data are in accordance with the literature.[26]

2.4.8 2-(3-(Azidomethyl)-7-bromo-9,9-dimethyl-9*H*-fluoren-2-yl)-4,4-dimethyl-4,5-dihydrooxazole (21)

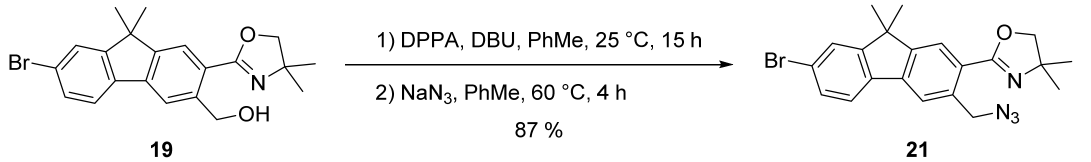

A modified literature procedure was used.[12] **19** (1.00 g, 2.50 mmol, 1.00 eq) was dissolved in PhMe (12.5 mL, 0.2 M) and DBU (485 µL, 3.25 mmol, 1.30 eq) was added *via* syringe pump (0.5 mL/min) at 25 °C. The mixture was stirred for ten minutes, whereupon DPPA (645 µL, 3.00 mmol, 1.20 eq) was added *via* syringe pump (0.3 mL/min). After complete addition the suspension was stirred for 15 hours at 25 °C. Due to incomplete consumption of the intermediately formed phosphate, NaN_3 (163 mg, 2.50 mmol, 1.00 eq) was added and the mixture was heated to 60 °C (oil bath temperature) for four hours. Saturated NH_4Cl solution (10 mL) and CH_2Cl_2 (30 mL) were added, the phases separated and the aqueous phase was extracted with CH_2Cl_2 (4 × 40 mL). The combined organic phases were dried over anhydrous Na_2SO_4, filtered and the solvent was removed under reduced pressure. Purification by flash column chromatography (SiO_2, CH:EE 15:1 v:v) provided **21** (921 mg, 2.17 mmol, 87 %) as a colorless oil. The oil solidified upon standing at 25 °C to form a colorless solid.

R_f = 0.34 (SiO_2, CH:EE 15:1 v:v). **Mp.:** 122 – 124 °C. **^{1}H-NMR (360 MHz, CDCl$_3$):** δ = 7.94 (s, 1H), 7.75 (s, 1H), 7.62 (d, $^3J_{HH}$ = 8.1 Hz, 1H), 7.57 (d, $^4J_{HH}$ = 1.8 Hz, 1H), 7.49 (dd, $^3J_{HH}$ = 8.1 Hz, $^4J_{HH}$ 1.8 Hz, 1H), 4.95 (s, 2H), 4.12 (s, 2H), 1.49 (s, 6H), 1.42 (s, 6H) ppm. **^{13}C{^{1}H}-NMR (91 MHz, CDCl$_3$):** δ = 161.5, 156.6, 152.8, 141.0, 137.1, 135.8, 130.5, 126.4, 126.0, 124.6, 122.4, 122.2, 121.3, 78.7, 68.5, 53.6, 47.4, 28.5 (2x), 26.9 (2x) ppm. **IR (ATR, neat):** $\tilde{\nu}$ = 2964 (w), 2924 (w), 2359 (w), 2103 (s), 1637 (m), 1458 (m), 1409 (m), 1360 (m), 1327 (m), 1301 (s), 1261 (s), 1200 (m), 1087 (m), 1061 (m), 1038 (s), 974 (m), 951 (m), 908 (m), 872 (m), 812 (s), 767 (m), 742 (s) cm^{-1}. **MS (APCI):** m/z = 425.2 [$C_{21}H_{21}{}^{79}BrN_4O$+H]$^+$. **HR-MS (APCI):** calculated for $C_{21}H_{22}{}^{79}BrN_4O^+$ [M+H]$^+$: m/z = 425.09715, found: 425.09753 (Dev.: 0.38 mu; 0.90 ppm). The analytical data are in accordance with the literature.[26]

2.4.9 3-(Azidomethyl)-7-bromo-9,9-dimethyl-9*H*-fluorene-2-carbaldehyde (5)

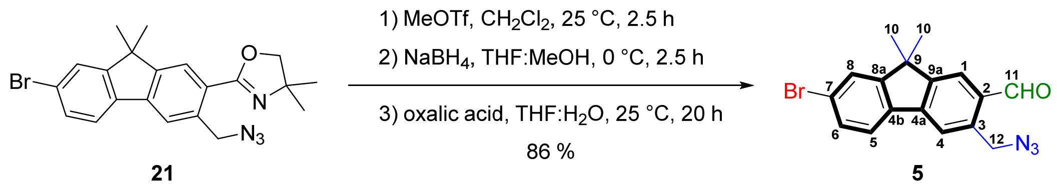

A modified literature procedure was used.[13] **21** (319 mg, 750 µmol, 1.00 eq) was dissolved in CH_2Cl_2 (2.50 mL, 0.3 M) and MeOTf (164 µL, 1.50 mmol, 2.00 eq) was added *via* syringe in one portion at 25 °C. After stirring for two and a half hours at the same temperature the solution was cooled to 0 °C. A solution of $NaBH_4$ (56.8 mg, 1.50 mmol, 2.00 eq) in THF:MeOH (2.5 mL, 4:1 v:v, 0.3 M) was added *via* syringe within five minutes and stirring was continued at 0 °C for another two and a half hours. Saturated NH_4Cl solution (5 mL), water (5 mL) and CH_2Cl_2 (10 mL) were added and the phases were separated. The aqueous layer was extracted with CH_2Cl_2 (4 × 20 mL) and the combined organic phases were dried over anhydrous Na_2SO_4. After filtration and solvent evaporation under reduced pressure, the residue was redissolved in THF:H_2O (2.5 mL, 4:1 v:v, 0.3 M). Oxalic acid dihydrate (189 mg, 1.50 mmol, 2.00 eq) was added at 25 °C and the mixture was stirred for 20 hours at the same temperature. Saturated $NaHCO_3$ solution (10 mL), water (10 mL) and CH_2Cl_2 (20 mL) were added and the phases separated. The aqueous layer was extracted with CH_2Cl_2 (4 × 20 mL), the combined organic phases were dried over anhydrous Na_2SO_4, filtered and the solvent was removed under reduced pressure. Purification by flash column chromatography (SiO_2, CH:EE 20:1 v:v) afforded **5** (230 mg, 646 µmol, 86 %) as a colorless oil. Upon standing at 25 °C the oil solidified very slowly to form a colorless solid. Single crystals suitable for X-ray analysis were grown by slow diffusion of cyclohexane into a CH_2Cl_2 solution.

R_f = 0.27 (SiO_2, CH:EE 20:1 v:v). **Mp.:** 103 – 105 °C. **^{1}H-NMR (360 MHz, $CDCl_3$):** δ = 10.20 (s, 1H, C*H*O), 7.90 (s, 1H, H–1), 7.84 (s, 1H, H–4), 7.69 (d, $^3J_{HH}$ = 8.1 Hz, 1H, H–5), 7.62 (d, $^4J_{HH}$ = 1.7 Hz, 1H, H–8), 7.53 (dd, $^3J_{HH}$ = 8.1 Hz, $^4J_{HH}$ = 1.8 Hz, 1H, H–6), 4.95 (s, 2H, C*H*$_2$), 1.53 (s, 6H, 2xC*H*$_3$) ppm. **^{13}C{^{1}H}-NMR (91 MHz, $CDCl_3$):** δ = 192.3 (C–11), 157.1 (C–8a), 153.3 (C–9a), 144.0 (C–4a), 137.5 (C–3), 136.5 (C–4b), 132.7 (C–2), 130.9 (C–6), 128.4 (C–1), 126.7 (C–8), 123.5 (C–7), 122.9 (C–5), 121.2 (C–4), 52.5 (C–12), 47.4 (C–9), 26.8 (2x, C–10) ppm. **IR (ATR, neat):** $\tilde{\nu}$ = 2965 (w), 2929 (w), 2860 (w), 2735 (w), 2359 (w), 2103 (s), 1682 (s), 1616 (m), 1599 (m) 1558 (s), 1452 (m), 1425 (m), 1361 (m), 1294 (m), 1254 (m), 1178 (s), 1151 (s), 1060 (m), 965 (m), 887 (m), 877 (m), 828 (s), 796 (s), 774 (m), 746 (s), 737 (s), 674 (m) cm^{-1}. **MS (EI, 70 eV):** m/z = 354.9 [$C_{17}H_{14}{}^{79}Br$]$^{+\cdot}$. **HR-MS (EI, 70 eV):** calculated for $C_{17}H_{14}{}^{79}BrN_3O^{+\cdot}$ [M]$^{+\cdot}$: m/z = 355.03148, found: 355.03133 (Dev.: –0.15 mu; –0.41 ppm). The analytical data are in accordance with the literature.[26]

2.4.10 2-(7-Bromo-3-(methoxymethyl)-9,9-dimethyl-9*H*-fluoren-2-yl)-4,4-dimethyl-4,5-dihydrooxazole (22)

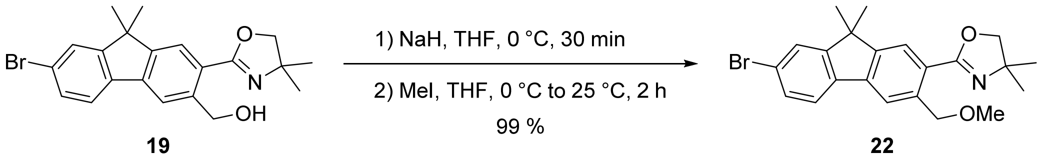

A slightly modified literature procedure was used.[28] A heat gun-dried and nitrogen-flushed *Schlenk* tube, equipped with a magnetic stirring bar and a rubber septum, was charged with **19** (200 mg, 500 µmol, 1.00 eq). The tube was flushed with nitrogen, anhydrous THF (5.00 mL, 0.1 M) was added and the colorless solution was cooled to 0 °C. NaH (40.0 mg, 1.00 mmol, 2.00 eq, 60 % in mineral oil) was added at once and the mixture was stirred at 0 °C for 30 minutes. MeI (68.5 µL, 156 mg, 1.10 mmol, 2.20 eq) was dropwise added *via* syringe at 0 °C and the mixture was stirred for two hours while it was allowed to warm to 25 °C. Saturated NH_4Cl solution (10 mL) and CH_2Cl_2 (10 mL) were added and the phases were separated. The aqueous phase was extracted with CH_2Cl_2 (4 × 10 mL) and the combined organic phases were dried over anhydrous Na_2SO_4, filtered and the solvent was removed under reduced pressure. Purification by flash column chromatography (SiO_2, CH:EE 5:1 v:v) provided **22** (206 mg, 497 mmol, 99 %) as a colorless oil, which solidified rapidly upon standing at 25 °C.

R_f = 0.34 (SiO_2, CH:EE 5:1 v:v). **Mp.:** 151 – 153 °C. **^{1}H-NMR (601 MHz, $CDCl_3$):** δ = 7.90 (s, 1H), 7.84 (s, 1H), 7.63 (d, $^3J_{HH}$ = 8.1 Hz, 1H), 7.56 (d, $^4J_{HH}$ = 1.7 Hz, 1H), 7.47 (dd, $^3J_{HH}$ = 8.1 Hz, $^4J_{HH}$ = 1.8 Hz, 1H), 4.88 (s, 2H), 4.10 (s, 2H), 3.50 (s, 3H), 1.48 (s, 6H), 1.41 (s, 6H) ppm. **^{13}C{^{1}H}-NMR (151 MHz, $CDCl_3$):** δ = 162.1, 156.6, 151.8, 140.7, 138.8, 137.6, 130.4, 126.4, 125.5, 124.1, 122.2, 122.0, 119.4, 78.7, 73.0, 68.3, 58.8, 47.3, 28.6 (2x), 27.0 (2x) ppm. **IR (ATR, neat):** $\tilde{\nu}$ = 2960 (w), 2921 (w), 2896 (w), 2804 (w), 1640 (m), 1601 (w), 1566 (w), 1483 (w), 1458 (w), 1430 (w), 1411 (w), 1384 (w), 1355 (m), 1302 (w), 1285 (w), 1262 (m), 1191 (m), 1161 (m), 1110 (s), 1084 (m), 1060 (m), 1035 (s), 971 (m), 945 (m), 921 (m), 869 (m), 901 (m), 882 (m), 820 (m), 809 (s), 765 (m), 742 (m), 672 (w) cm^{-1}. **MS (ESI):** m/z = 413.9 [$C_{22}H_{24}{}^{79}BrNO_2$+H]$^+$. **HR-MS (ESI):** calculated for $C_{22}H_{25}{}^{79}BrNO_2{}^+$ [M+H]$^+$: m/z = 414.10632, found: 414.10617 (Dev.: –0.15 mu; –0.35 ppm); calculated for $C_{22}H_{24}{}^{79}BrNNaO_2{}^+$ [M+Na]$^+$: m/z = 436.08826, found: 436.08811 (Dev.: –0.15 mu; –0.34 ppm).

2.4.11 7-Bromo-3-(methoxymethyl)-9,9-dimethyl-9*H*-fluorene-2-carbaldehyde (23)

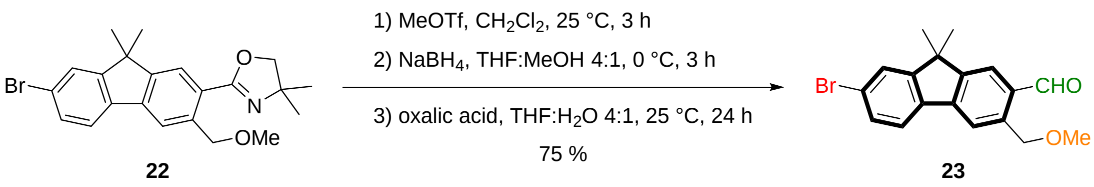

A modified literature procedure was used.[13] **22** (82.9 mg, 200 µmol, 1.00 eq) was dissolved in CH_2Cl_2 (1.00 mL, 0.2 M) and MeOTf (43.8 µL, 400 µmol, 2.00 eq) was added *via* syringe in one portion at

25 °C. After stirring for three hours at the same temperature the solution was cooled to 0 °C. A solution of $NaBH_4$ (15.1 mg, 400 µmol, 2.00 eq) in THF:MeOH (1.0 mL, 4:1 v:v, 0.4 M) was added *via* syringe within five minutes and stirring was continued at 0 °C for another three hours. Saturated NH_4Cl solution (5 mL), water (5 mL) and CH_2Cl_2 (10 mL) were added and the phases were separated. The aqueous layer was extracted with CH_2Cl_2 (4 × 10 mL) and the combined organic phases were dried over anhydrous Na_2SO_4. After filtration and solvent evaporation under reduced pressure, the residue was redissolved in THF:H_2O (1.0 mL, 4:1 v:v, 0.2 M). Oxalic acid dihydrate (50.4 mg, 400 µmol, 2.00 eq) was added at 25 °C and the mixture was stirred for 24 hours under the same conditions. Saturated $NaHCO_3$ solution (5 mL), water (5 mL) and CH_2Cl_2 (10 mL) were added and the phases were separated. The aqueous layer was extracted with CH_2Cl_2 (4 × 10 mL), the combined organic phases were dried over anhydrous Na_2SO_4, filtered and the solvent was removed under reduced pressure. Purification by flash column chromatography (SiO_2, CH:EE 10:1 v:v) afforded **23** (51.6 mg, 150 µmol, 75 %) as a colorless solid.

R_f = 0.18 (SiO_2, CH:EE 10:1 v:v). **Mp.:** 62 – 64 °C. **^{1}H-NMR (600 MHz, $CDCl_3$):** δ = 10.26 (s, 1H), 7.92 (s, 1H), 7.91 (s, 1H), 7.68 (d, $^3J_{HH}$ = 8.1 Hz, 1H), 7.61 (d, $^4J_{HH}$ = 1.6 Hz, 1H), 7.51 (dd, $^3J_{HH}$ = 8.1 Hz, $^4J_{HH}$ = 1.7 Hz, 1H), 4.93 (s, 2H), 3.54 (s, 3H), 1.51 (s, 6H) ppm. **^{13}C{^{1}H}-NMR (151 MHz, $CDCl_3$):** δ = 192.2, 157.2, 152.6, 143.8, 141.0, 136.9, 132.8, 130.7, 126.6, 126.2, 123.2, 122.8, 120.0, 72.2, 58.9, 47.4, 26.9 (2x) ppm. **IR (ATR, neat):** $\tilde{\nu}$ = 2956 (w), 2923 (w), 2864 (w), 2820 (w), 2721 (w), 1682 (s), 1614 (m), 1598 (m), 1559 (s), 1471 (m), 1444 (m), 1405 (m), 1384 (m), 1361 (w), 1334 (m), 1302 (w), 1282 (w), 1245 (m), 1222 (w), 1178 (s), 1156 (m), 1134 (m), 1104 (s), 1079 (m), 1057 (m), 980 (s), 907 (m), 886 (m), 838 (m), 825 (s), 798 (s), 772 (m), 729 (s), 673 (m) cm^{-1}. **MS (ESI):** m/z = 413.9 $[C_{22}H_{24}{}^{79}BrNO_2+H]^+$. **HR-MS (ESI):** calculated for $C_{18}H_{17}{}^{79}BrNaO_2{}^+$ $[M+Na]^+$: m/z = 367.03041, found: 367.03027 (Dev.: –0.14 mu; –0.39 ppm); calculated for $C_{36}H_{34}{}^{79}Br_2NaO_4{}^+$ $[2M+Na]^+$: m/z = 711.07160, found: 711.07146 (Dev.: –0.14 mu; –0.20 ppm).

2.4.12 9-Bromo-3,3,11,11-tetramethyl-2,3,5,11-tetrahydroindeno[1,2-f]oxazolo[2,3-*a*]isoindol-4-ium bromide (25)

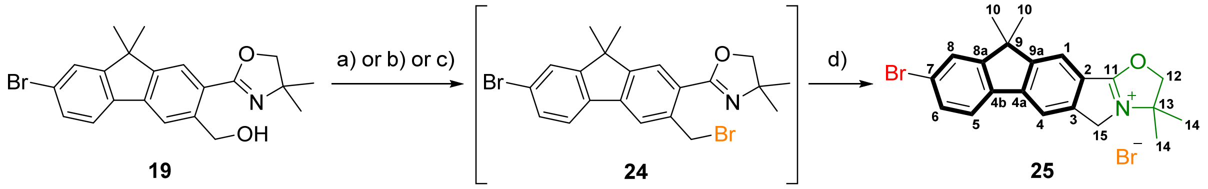

Reaction conditions: a) CBr_4, PPh_3, CH_2Cl_2, 0 °C to 25 °C, 2 h, 95 %; b) NBS, PPh_3, CH_2Cl_2, 0 °C to 25 °C, 2 h, 91 %; c) PBr_3, CH_2Cl_2, 0 °C to 25 °C, 2 h, 92 %; d) spontaneous during/upon workup.

For a): A slightly modified literature procedure was used.[29] A heat gun-dried and nitrogen-flushed *Schlenk* tube, equipped with a magnetic stirring bar and a rubber septum, was charged with **19** (80.1 mg,

200 µmol, 1.00 eq). The tube was flushed with nitrogen, anhydrous CH_2Cl_2 (2.00 mL, 0.1 M) was added and the colorless solution was cooled to 0 °C. PPh_3 (78.7 mg, 300 µmol, 1.50 eq) and CBr_4 (99.5 mg, 300 µmol, 1.50 eq) were added subsequently and the mixture was stirred for two hours while it was allowed to warm to 25 °C – the initial dark yellow color disappeared in the course of the reaction. After full conversion the reaction mixture was directly flushed through a plug of neutral alumina using CH_2Cl_2. The solvent was removed under reduced pressure to afford the cyclized iminium bromide **25** (88.3 mg, 191 µmol, 95 %) as a colorless foam.

For b): A slightly modified literature procedure was used.[30] A heat gun-dried and nitrogen-flushed *Schlenk* tube, equipped with a magnetic stirring bar and a rubber septum, was charged with **19** (80.1 mg, 200 µmol, 1.00 eq). The tube was flushed with nitrogen, anhydrous CH_2Cl_2 (2.00 mL, 0.1 M) was added and the colorless solution was cooled to 0 °C. PPh_3 (78.7 mg, 300 µmol, 1.50 eq) and NBS (53.4 mg, 300 µmol, 1.50 eq) were added subsequently and the mixture was stirred for two hours while it was allowed to warm to 25 °C – the mixture stayed slightly yellow in the course of the reaction. After full conversion the reaction mixture was directly flushed through a plug of neutral alumina using CH_2Cl_2. The solvent was removed under reduced pressure to afford the cyclized iminium bromide **25** (84.5 mg, 182 µmol, 91 %) as a colorless foam.

For c): A slightly modified literature procedure was used.[31] A heat gun-dried and nitrogen-flushed *Schlenk* tube, equipped with a magnetic stirring bar and a rubber septum, was charged with **19** (80.1 mg, 200 µmol, 1.00 eq). The tube was flushed with nitrogen, anhydrous CH_2Cl_2 (2.00 mL, 0.1 M) was added and the colorless solution was cooled to 0 °C. PBr_3 (28.5 mL, 81.2 mg, 300 µmol, 1.50 eq) was added dropwise and the mixture was stirred for two hours while it was allowed to warm to 25 °C – the mixture stayed colorless in the course of the reaction. After full conversion the reaction mixture was directly flushed through a plug of neutral alumina using CH_2Cl_2. The solvent was removed under reduced pressure to afford the cyclized iminium bromide **25** (85.2 mg, 184 µmol, 92 %) as a colorless foam.

24: R*f* = 0.44 (SiO_2, CH:EE 6:1 v:v). **HR-MS (ESI):** calculated for $C_{21}H_{22}{}^{79}Br_2NO^+$ $[M+H]^+$: m/z = 462.00626, found: 462.00581 (Dev.: –0.46 mu; –0.99 ppm); calculated for $C_{21}H_{21}{}^{79}Br_2NNaO^+$ $[M+Na]^+$: m/z = 483.98821, found: 483.98779 (Dev.: –0.42 mu; –0.87 ppm); calculated for $C_{21}H_{21}{}^{79}Br_2KNO^+$ $[M+K]^+$: m/z = 499.96215, found: 499.96202 (Dev.: –0.13 mu; –0.26 ppm); calculated for $C_{42}H_{43}{}^{79}Br_4N_2O_2^+$ $[2M+H]^+$: m/z = 923.00525, found: 923.00458 (Dev.: –0.67 mu; –0.73 ppm); calculated for $C_{42}H_{42}{}^{79}Br_4N_2NaO_2^+$ $[2M+Na]^+$: m/z = 944.98720, found: 944.98614 (Dev.: –1.06 mu; –1.12 ppm); calculated for $C_{42}H_{42}{}^{79}Br_4KN_2O_2^+$ $[2M+K]^+$: m/z = 960.96114, found: 960.96237 (Dev.: 1.24 mu; 1.29 ppm).

25: R$_f$ = 0.18 (SiO_2, CH:EE 6:1 v:v). **Mp.:** 108 – 110 °C. **^{1}H-NMR (601 MHz, $CDCl_3$):** δ = 7.85 (s, 1H, H–1), 7.69 (s, 1H, H–4), 7.61 (d, $^3J_{HH}$ = 8.1 Hz, 1H, H–5), 7.59 (d, $^4J_{HH}$ = 1.7 Hz, 1H, H–8), 7.49 (dd, $^3J_{HH}$ = 8.1 Hz, $^4J_{HH}$ = 1.8 Hz, 1H, H–6), 4.60 (s, 2H, H–15), 4.16 (s, 2H, H–12), 1.69 (s, 6H, H–14), 1.50 (s, 6H, H–10) ppm. **^{13}C{^{1}H}-NMR (151 MHz, $CDCl_3$):** δ = 169.2 (C=N$^+$), 156.6 (C–8a), 153.8 (C–9a), 142.0 (C–4a), 140.6 (C–3), 137.2 (C–4b), 133.2 (C–2), 130.5 (C–6), 126.6 (C–8), 122.4 (C–7), 122.0 (C–5), 117.8 (C–1), 114.0 (C–4), 56.7 (C–13), 50.0 (C–15), 47.1 (C–9), 41.7 (C–12), 27.2 (C–10), 25.4 (C–14) ppm. **IR (ATR, neat):** $\tilde{\nu}$ = 2960 (w), 2922 (w), 2860 (w), 1669 (s), 1622 (m), 1486 (w), 1436 (m), 1388 (s), 1362 (m), 1314 (w), 1247 (s), 1200 (s), 1182 (m), 1142 (m), 1088 (m), 1065 (m), 976 (w), 951 (w), 875 (m), 822 (s), 780 (s), 765 (m), 737 (m), 703 (m), 660 (m) cm^{-1}. **MS (ESI):** m/z = 382.0 [$C_{21}H_{21}{}^{79}BrNO$]$^+$. **HR-MS (ESI):** calculated for $C_{21}H_{21}{}^{79}BrNO^+$ [M–Br$^-$]$^+$: m/z = 382.08010, found: 382.07985 (Dev.: –0.25 mu; –0.65 ppm).

2.4.13 9-Bromo-3,3,4,11,11-pentamethyl-2,3,4,5,11,12b-hexahydroindeno[1,2-*f*]oxazolo[2,3-*a*]isoindol-4-ium trifluoromethanesulfonate (28)

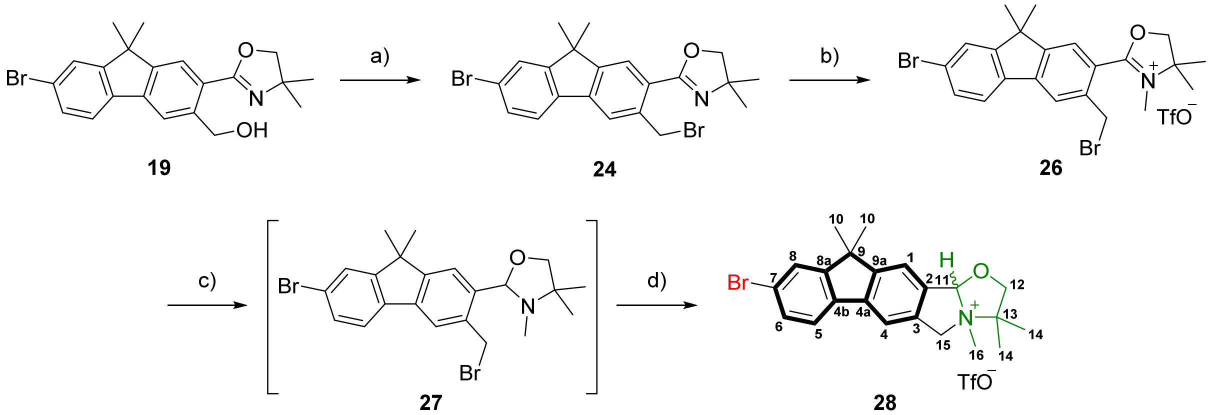

Reaction conditions: a) CBr_4, PPh_3, CH_2Cl_2, 0 °C to 25 °C, 2 h, rapid filtration over neutral alumina b) MeOTf, CH_2Cl_2, 25 °C, 3 h; c) $NaBH_4$, THF:MeOH 4:1 v:v, 0 °C, 3 h; d) during reaction, 56 % from **19**.

Slightly modified literature procedures were used.[13, 29] A heat gun-dried and nitrogen-flushed *Schlenk* tube, equipped with a magnetic stirring bar and a rubber septum, was charged with **19** (160 mg, 400 µmol, 1.00 eq). The tube was flushed with nitrogen, anhydrous CH_2Cl_2 (2.00 mL, 0.1 M) was added and the colorless solution was cooled to 0 °C. PPh_3 (157 mg, 600 µmol, 1.50 eq) and CBr_4 (199 mg, 600 µmol, 1.50 eq) were added subsequently and the mixture was stirred for two hours while it was allowed to warm to 25 °C – the initial dark yellow color disappeared in the course of the reaction. After full conversion the reaction mixture was rapidly flushed through a plug of neutral alumina using CH_2Cl_2. The solvent was removed under reduced pressure on a rotary evaporator (30 °C water bath temperature) to obtain primarily the benzyl bromide **24** as a colorless foam.

The crude product was dissolved in CH_2Cl_2 (2.00 mL, 0.2 M) and MeOTf (87.5 µL, 800 µmol, 2.00 eq) was added *via* syringe in one portion at 25 °C. After stirring for three hours at the same temperature the

solution was cooled to 0 °C. A solution of $NaBH_4$ (30.3 mg, 800 µmol, 2.00 eq) in THF:MeOH (1.0 mL, 4:1 v:v, 0.4 M) was added *via* syringe within five minutes and stirring was continued at 0 °C for another three hours. Saturated NH_4Cl solution (5 mL), water (5 mL) and CH_2Cl_2 (10 mL) were added and the phases were separated. The aqueous layer was extracted with CH_2Cl_2 (4 × 10 mL) and the combined organic phases were dried over anhydrous Na_2SO_4, filtered and the solvent was removed under reduced pressure. Due to unexpected cyclization a further acidic hydrolysis using oxalic acid becomes impossible. Purification by flash column chromatography (SiO_2, CH_2Cl_2:MeOH 15:1 v:v) afforded the ammonium triflate **28** (87.7 mg, 223 µmol, 56 % from **19**) as a colorless foam.

R_f = 0.14 (SiO_2, CH_2Cl_2:MeOH 15:1 v:v). **Mp.:** 205 – 222 °C (very long decomp.). **^{1}H-NMR (601 MHz, MeOD–d_4):** δ = 7.86 (s, 1H, H–4), 7.77 (d, $^3J_{HH}$ = 8.1 Hz, 1H, H–5), 7.73 (s, 1H, H–1), 7.72 (d, $^4J_{HH}$ = 1.7 Hz, 1H, H–8), 7.54 (dd, $^3J_{HH}$ = 8.1 Hz, $^4J_{HH}$ = 1.6 Hz, 1H, H–6), 6.56 (s, 1H, H–11), 5.43 (d, $^2J_{HH}$ = 14.9 Hz, 1H, H–15), 4.73 (d, $^2J_{HH}$ = 14.9 Hz, 1H, H–15), 4.35 (d, $^2J_{HH}$ = 9.8 Hz, 1H, H–12), 4.31 (d, $^2J_{HH}$ = 9.8 Hz, 1H, H–12), 3.26 (s, 3H, H–16), 1.77 (s, 3H, H–14), 1.69 (s, 3H, H–14), 1.51 (s, 3H, H–10), 1.51 (s, 3H, H–10) ppm. **^{13}C{^{1}H}-NMR (151 MHz, MeOD–d_4):** δ = 157.7 (C–8a), 156.7 (C–9a), 143.5 (C–4a), 137.9 (C–4b), 135.7 (C–3), 133.2 (C–2), 131.7 (C–6), 127.6 (C–8), 123.6 (C–7), 123.4 (C–5), 121.8 (q, *C*F_3, $^1J_{CF}$ = 318.4 Hz), 120.8 (C–1), 116.5 (C–4), 107.5 (C–11), 79.1 (C–12), 75.6 (C–13), 65.8 (C–15), 48.4 (C–9), 44.2 (C–16), 27.1 (C–10), 27.0 (C–10), 23.2 (C–14), 18.8 (C–14) ppm. **^{19}F{^{1}H}-NMR (565 MHz, MeOD-d_4):** δ = –80.09 (s) ppm. **IR (ATR, neat):** $\tilde{\nu}$ = 2965 (w), 2927 (w), 1623 (w), 1601 (w), 1475 (w), 1457 (w), 1436 (w), 1405 (w), 1388 (w), 1360 (w), 1251 (s), 1224 (s), 1190 (m), 1154 (s), 1086 (m), 1050 (m), 1028 (s), 968 (m), 918 (m), 878 (m), 820 (m), 755 (m), 676 (w) cm^{-1}. **MS (ESI):** m/z = 397.8 $[C_{22}H_{25}{}^{79}BrNO]^+$. **HR-MS (ESI):** calculated for $C_{22}H_{25}{}^{79}BrNO^+$ $[M–OTf^-]^+$: m/z = 398.11140, found: 398.11122 (Dev.: –0.18 mu; –0.45 ppm).

2.4.14 Attempted Preparation of (7-bromo-2-(4,4-dimethyl-4,5-dihydrooxazol-2-yl)-9,9-dimethyl-9H-fluoren-3-yl)methyl methanesulfonate (S3)

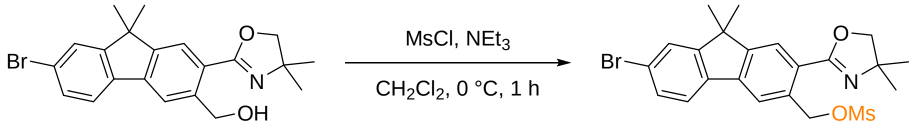

19 **S3**

A slightly modified literature procedure was used.[32] A heat gun-dried and nitrogen-flushed *Schlenk* tube, equipped with a magnetic stirring bar and a rubber septum, was charged with **19** (400 mg, 1.00 mmol, 1.00 eq). The tube was flushed with nitrogen, anhydrous CH_2Cl_2 (5.00 mL, 0.2 M) was added and the colorless solution was cooled to 0 °C. NEt_3 (152 mg, 209 µL, 1.50 mmol, 1.50 eq) and MsCl (160 mg, 108 µL, 1.40 mmol, 1.40 eq) were added successively and the mixture was stirred at 0 °C for one hour. Despite complete conversion of **19**, only traces of **S3** were observed.

2.5 Preparation of Sterically Hindered Alkynes

2.5.1 Preparation of (3*r*,5*r*,7*r*)-1-ethynyladamantane (31)

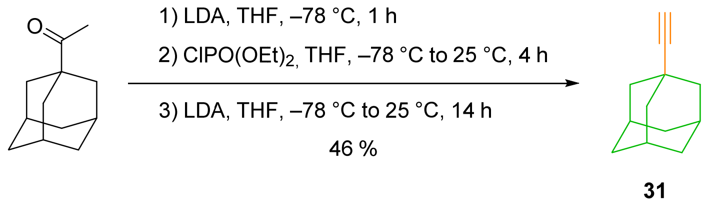

A modified literature procedure was used.[33] Initially a fresh LDA solution was prepared. Therefore, a heat gun-dried and nitrogen-flushed *Schlenk* flask, equipped with a magnetic stirring bar and a rubber septum, was charged with anhydrous THF (16.7 mL) and *N*,*N*-diisopropylamine (1.69 mL, 12.0 mmol, 1.20 eq) was added. The solution was cooled to –78 °C and *n*BuLi (4.40 mL, 11.0 mmol, 1.10 eq, 2.50 M in hexane) was added in one portion, whereupon the solution turned slightly yellow. After stirring for 30 minutes at the same temperature 1-((3*r*,5*r*,7*r*)-adamantan-1-yl)ethan-1-one (1.78 g, 10.0 mmol, 1.00 eq), dissolved in anhydrous THF (12.5 mL), was added dropwise *via* syringe pump (0.5 mL/min) to the LDA solution. After complete addition the solution was stirred at –78 °C for an additional one hour and then diethyl chlorophosphate (1.59 mL, 11.0 mmol, 1.10 eq) was added *via* syringe pump (0.5 mL/min), whereupon the mixture was stirred for one more hour under the same conditions. The cooling bath was removed and the mixture was stirred for three more hours while it was allowed to warm to 25 °C. In a separately heat gun-dried and nitrogen-flushed *Schlenk* flask, equipped with a magnetic stirring bar and a rubber septum, was prepared a second LDA solution. Therefore, *N*,*N*-diisopropylamine (2.96 mL, 21.0 mmol, 2.10 eq) was added to anhydrous THF (25.0 mL) and the solution was cooled to –78 °C. *n*BuLi (8.00 mL, 20.0 mmol, 2.00 eq, 2.50 M in hexane) was added in one portion and the slightly yellow solution was stirred for 30 minutes at the same temperature. The phosphate containing solution was added *via* syringe pump (1.0 mL/min) to the second LDA solution at –78 °C and stirred under these conditions for two hours. Then the mixture was allowed to warm slowly to 25 °C and stirred for 12 hours in all. Saturated NH_4Cl solution (40 mL) and water (20 mL) were added and the phases separated. The aqueous layer was extracted with ethyl acetate (3 × 40 mL) and the combined organic layers were dried over anhydrous Na_2SO_4, filtered and the solvent was evaporated under reduced pressure. Purification by flash column chromatography (SiO_2, CH) afforded **31** (735 mg, 4.59 mmol, 46 %) as a colorless solid.

R*f* = 0.74 (SiO_2, CH, staining with $KMnO_4$). **Mp.:** 79 – 81 °C (Lit.[34]: 83 °C). **^{1}H-NMR (360 MHz, $CDCl_3$):** δ = 2.10 (s, 1H), 1.95 (br. s, 3H), 1.88 (d, $^3J_{HH}$ = 2.8 Hz, 6H), 1.68 (t, $^3J_{HH}$ = 3.1 Hz, 6H) ppm. **^{13}C{^{1}H}-NMR (91 MHz, $CDCl_3$):** δ = 93.2, 66.7, 42.9 (3x), 36.4 (3x), 29.5, 28.0 (3x) ppm. **IR (ATR, neat):** $\tilde{\nu}$ = 3304 (m), 2900 (s), 2849 (s), 2657 (w), 2104 (w), 1450 (s), 1356 (w), 1343 (w), 1314 (w),

1227 (m), 1182 (w), 1132 (w), 1098 (m), 1023 (w), 975 (w), 932 (w), 811 (m), 772 (w), 699 (w) cm^{-1}. **MS (EI, 70 eV):** m/z = 160.1 $[M]^{+\cdot}$. The analytical data are in accordance with the literature.[33]

2.5.2 Preparation of 1,3-di-*tert*-butyl-5-ethynylbenzene (32)

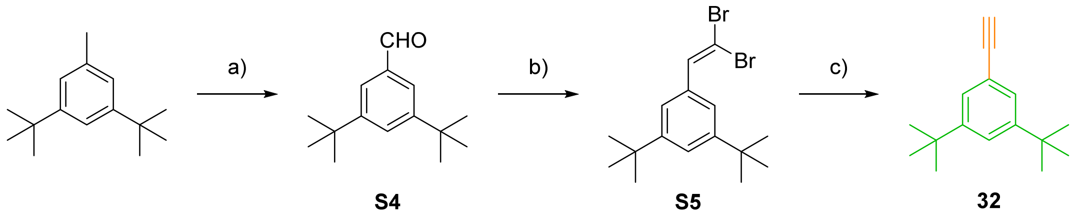

Reaction conditions: a) 1) NBS, AIBN, PhH, 90 °C, 16 h; 2) $(CH_2)_6N_4$, EtOH:H_2O 1:1 v:v, 90 °C 4 h; (3) conc. HCl, 90 °C, 0.5 h, 80 %; b) PPh_3, Zn, CBr_4, CH_2Cl_2, 0 °C to 25 °C, 3 h, 96 %; c) *n*BuLi, THF, –78 °C to 25 °C, 5 h, 89 %.

2.5.2.1 3,5-Di-*tert*-butylbenzaldehyde (S4)

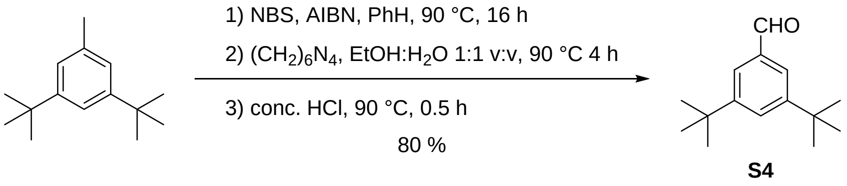

A modified literature procedure was used.[35] 1,3-Di-*tert*-butyl-5-methylbenzene (3.07 g, 15.0 mmol, 1.00 eq) was dissolved in benzene (15.0 mL, 1.0 M) and NBS (1.50 g, 22.5 mmol, 1.50 eq) and AIBN (123 mg, 750 µmol, 0.05 eq) were added. The mixture was heated to 100 °C (oil bath temperature) and stirred for 16 hours at this temperature. After cooling to 25 °C, the mixture was filtered through Celite® and washed with ethyl acetate (~50 mL). The solution was concentrated under reduced pressure to afford a brown oil, which was used without further purification. The crude product was dissolved in EtOH:H_2O (15 mL 1:1 v:v) and hexamethylenetetramine (6.31 g, 45.0 mmol, 3.00 eq) was added in one portion. Heating for four hours at 100 °C (oil bath temperature) was followed by the addition of concentrated HCl and heating was continued for another 30 minutes. Ethanol was removed under reduced pressure by rotary evaporation and water (35 mL) and ethyl acetate (50 mL) were added. The phases were separated and the aqueous phase was extracted with ethyl acetate (3 × 50 mL). The combined organic phases were dried over anhydrous Na_2SO_4, filtered and concentrated under reduced pressure. Purification by flash column chromatography (SiO_2, CH:EE 50:1 v:v) afforded **S4** (2.61 g, 12.0 mmol, 80 %) as a colorless oil, which slowly solidified upon standing at 25 °C.

$\mathbf{R_f}$ = 0.09 (SiO_2, CH). $\mathbf{R_f}$ = 0.26 (SiO_2, CH:EE 50:1 v:v). **Mp.:** 83 – 85 °C (Lit.[36]: 84.2 – 84.3 °C). **^{1}H-NMR (360 MHz, $CDCl_3$):** δ = 10.01 (s, 1H), 7.76 – 7.69 (m, 3H), 1.37 (s, 18H) ppm. **^{13}C{^{1}H}-NMR (91 MHz, $CDCl_3$):** δ = 193.4, 152.0 (2x), 136.3, 129.0, 124.3 (2x), 35.1 (2x), 31.5 (6x) ppm. **IR (ATR, neat):** $\tilde{\nu}$ = 2960 (m), 2905 (w), 2868 (w), 2817 (w), 2734 (w), 1686 (s), 1591 (m), 1474 (m), 1463 (m),

1388 (m), 1363 (m), 1325 (w), 1305 (w), 1278 (w), 1250 (m), 1189 (s), 1127 (w), 1011 (w), 948 (w), 900 (w), 882 (m), 818 (m), 768 (w), 706 (s), 699 (s) cm^{-1}. **MS (EI, 70 eV):** m/z = 218.1 $[M]^{+\cdot}$. The analytical data are in accordance with the literature.[37]

2.5.2.2 1,3-Di-*tert*-butyl-5-(2,2-dibromovinyl)benzene (S5)

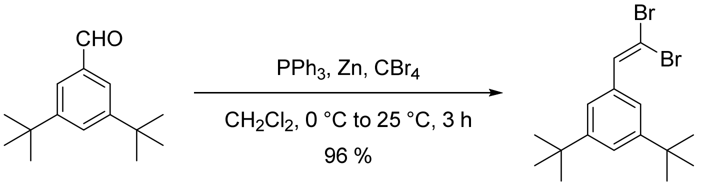

S4 **S5**

A modified literature procedure was used.[38] A heat gun-dried and nitrogen-flushed *Schlenk* flask, equipped with a magnetic stirring bar and a rubber septum, was charged with PPh_3 (4.72 g, 18.0 mmol, 2.00 eq) and anhydrous CH_2Cl_2 (45 mL) was added. Zinc dust (particle < 10 µm) was added in one portion and the suspension was cooled to 0 °C. CBr_4 (5.97 g, 18.0 mmol, 2.00 eq) was added in one portion whereupon the suspension turned dirty green. The mixture was allowed to warm slowly to ambient temperature and stirred for 24 hours, while the color turned deep red. **S4** (1.97 g, 9.00 mmol, 1.00 eq) was dissolved in anhydrous CH_2Cl_2 (15 mL) and added *via* syringe pump (1.0 mL/min) to the ice cooled (0 °C) mixture. Stirring was continued for three more hours while the mixture was allowed to warm to 25 °C. Filtration through a short plug of Celite® and washing with CH_2Cl_2 (150 mL) was followed by removing of the solvent under reduced pressure. Purification by flash column chromatography (SiO_2, CH) afforded **S5** (3.23 g, 8.63 mmol, 96 %) as a colorless oil which slowly solidified upon standing at 25 °C.

$\mathbf{R_f}$ = 0.78 (SiO_2, CH). **Mp.:** 41 – 44 °C. **^{1}H-NMR (360 MHz, $CDCl_3$):** δ = 7.52 (br. s, 1H), 7.43 – 7.37 (m, 3H), 1.34 (s, 18H) ppm. **^{13}C{^{1}H}-NMR (91 MHz, $CDCl_3$):** δ = 150.9 (2x), 137.9, 134.5, 122.9 (2x), 122.8, 88.7, 35.0 (2x), 31.5 (6x) ppm. **IR (ATR, neat):** $\tilde{\nu}$ = 3308 (w), 2961 (s), 2903 (s), 2853 (m), 2733 (w), 2105 (w), 1687 (s), 1593 (m), 1475 (m), 1451 (m), 1389 (m), 1363 (s), 1305 (w), 1278 (w), 1249 (m), 1189 (s), 1127 (w), 1098 (w), 1011 (w), 949 (w), 900 (m), 882 (s), 827 (m), 817 (m), 799 (m), 767 (w), 705 (s) cm^{-1}. **MS (EI, 70 eV):** m/z = 371.9 $[C_{16}H_{22}{}^{79}Br_2]^{+\cdot}$. **HR-MS (EI, 70 eV):** calculated for $C_{16}H_{22}{}^{79}Br_2{}^{+\cdot}$ $[M]^{+\cdot}$: m/z = 372.00883, found: 372.00842 (Dev.: 0.41 mu; 1.10 ppm). The analytical data are in accordance with the literature.[38]

2.5.2.3 1,3-Di-*tert*-butyl-5-ethynylbenzene (32)

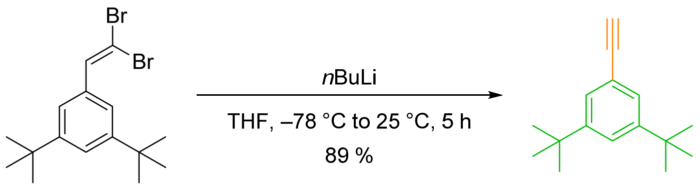

A modified literature procedure was used.[38] A heat gun-dried and nitrogen-flushed *Schlenk* flask, equipped with a magnetic stirring bar and a rubber septum, was charged with **S5** (1.87 g, 5.00 mmol, 1.00 eq) and dissolved in anhydrous THF (12.5 mL, 0.4 M). The solution was cooled to –78 °C and *n*BuLi (6.00 mL, 15.0 mmol, 3.00 eq, 2.50 M in hexane) was added *via* syringe pump (1.0 mL/min). Stirring was continued for one hour at –78 °C, then the cooling bath was removed while it was allowed to warm slowly to 25 °C and stirred for four hours in all. Cooling to 0 °C was followed by the dropwise addition of saturated NH_4Cl solution (10 mL). Water (10 mL) and CH_2Cl_2 (10 mL) were added and the phases separated. The aqueous phase was extracted with CH_2Cl_2 (3 × 20 mL), the combined organic phases dried over anhydrous Na_2SO_4, filtered and concentrated under reduced pressure. Purification by flash column chromatography (SiO_2, CH) provided **32** (949 mg, 4.43 mmol, 89 %) as a colorless solid.

R_f = 0.66 (SiO_2, CH). **Mp.:** 83 – 85 °C (Lit.[38]: 88 – 89 °C). **^{1}H-NMR (360 MHz, $CDCl_3$):** δ = 7.45 – 7.38 (m, 1H), 7.38 – 7.32 (m, 2H), 3.03 (s, 1H), 1.31 (s, 18H) ppm. **^{13}C{^{1}H}-NMR (91 MHz, $CDCl_3$):** δ = 151.0 (2x), 126.5 (2x), 123.4, 121.1, 85.0, 75.9, 34.9 (2x), 31.4 (6x) ppm. **IR (ATR, neat):** $\tilde{\nu}$ = 3272 (m), 2951 (s), 2903 (m), 2868 (m), 1776 (w), 1591 (m), 1477 (m), 1460 (m), 1422 (m), 1392 (w), 1364 (s), 1316 (w), 1248 (m), 1192 (m), 1137 (w), 1025 (w), 935 (w), 914 (w), 900 (m), 883 (s), 710 (s), 673 (s) cm^{-1}. **MS (EI, 70 eV):** m/z = 214.1 $[M]^{+\cdot}$. **HR-MS (EI, 70 eV):** calculated for $C_{16}H_{22}^{+\cdot}$ $[M]^{+\cdot}$: m/z = 214.17215, found: 214.17143 (Dev.: 0.72 mu; 3.36 ppm). The analytical data are in accordance with the literature.[38]

2.6 Copper-Catalyzed Azide-Alkyne Cycloadditions (CuAAC)

2.6.1 General Procedure for the CuAACs (GP1)

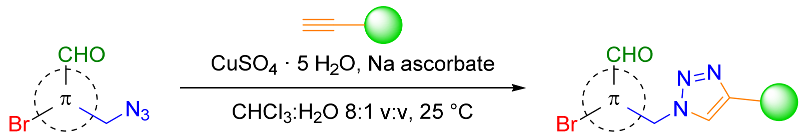

A modified literature procedure was used.[39] In a screw-cap vial azide (100 µmol, 1.00 eq) and terminal alkyne (105 µmol, 1.05 eq) were dissolved in $CHCl_3$ (1.00 mL, 0.1 M). $CuSO_4 \cdot 5\ H_2O$ (2.4 mg, 10 µmol, 0.10 eq) and sodium ascorbate (9.9 mg, 50 µmol, 0.50 eq) were suspended separately in water (125 µL) and added to the reaction vial. The heterogeneous mixture was stirred vigorously at 25 °C for the indicated time and directly purified by flash column chromatography.

2.6.2 4-Bromo-2-((4-octyl-1*H*-1,2,3-triazol-1-yl)methyl)benzaldehyde (33)

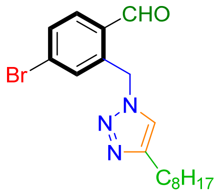

33

According to GP1, the reaction of **3** (24.0 mg, 100 µmol, 1.00 eq) and 1-decyne (19.0 µL, 105 µmol, 1.05 eq) provided after 24 hours reaction time and subsequent flash column chromatography (SiO_2, CH:EE 3:1 v:v) **32** (35.5 mg, 93.8 µmol, 94 %) as a colorless solid.

R_f = 0.33 (SiO_2, CH:EE 3:1 v:v). **Mp.:** 82 – 84 °C. **^{1}H-NMR (360 MHz, $CDCl_3$):** δ = 10.08 (d, $^4J_{HH}$ = 0.6 Hz, 1H), 7.72 – 7.67 (m, 2H), 7.39 (s, 1H), 7.22 – 7.16 (m, 1H), 5.94 (s, 2H), 2.71 (t, $^3J_{HH}$ = 7.6 Hz, 2H), 1.66 (p, $^3J_{HH}$ = 7.9 Hz, 2H), 1.39 – 1.20 (m, 10H), 0.86 (t, $^3J_{HH}$ = 7.2 Hz, 3H) ppm. **^{13}C{^{1}H}-NMR (91 MHz, $CDCl_3$):** δ = 192.6, 149.1, 138.6, 136.7, 132.9, 132.3, 131.8, 130.1, 121.7, 50.2, 32.0, 29.5, 29.4, 29.4, 29.3, 25.8, 22.8, 14.2 ppm. **IR (ATR, neat):** $\tilde{\nu}$ = 3056 (w), 2950 (w), 2921 (m), 2850 (m), 2782 (w), 1686 (s), 1583 (s), 1562 (m), 1466 (w), 1435 (m), 1334 (m), 1288 (w), 1210 (m), 1196 (m), 1174 (m), 1141 (m), 1097 (m), 1079 (m), 1055 (m), 1033 (w), 866 (s), 834 (m), 803 (s), 721 (m), 670 (w) cm^{-1}. **MS (APCI):** m/z = 378.1 $[C_{18}H_{24}{}^{79}BrN_3O+H]^+$. **HR-MS (EI, 70 eV):** calculated for $C_{18}H_{24}{}^{79}BrN_3O^{+\cdot}$ $[M]^{+\cdot}$: m/z = 377.10973, found: 377.10924 (Dev.: –0.49 mu; –1.29 ppm); calculated for $C_{18}H_{24}{}^{81}BrN_3O^{+\cdot}$ $[M]^{+\cdot}$: m/z = 379.10768, found: 379.10780 (Dev.: 0.12 mu; 0.32 ppm).

2.6.3 2-((4-((1*s*,3*s*)-Adamantan-1-yl)-1*H*-1,2,3-triazol-1-yl)methyl)-4-bromobenzaldehyde (34)

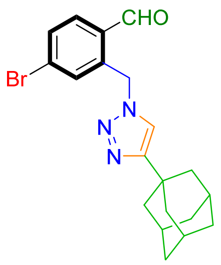

34

According to GP1, the reaction of **3** (24.0 mg, 100 µmol, 1.00 eq) and **31** (16.8 mg, 105 µmol, 1.05 eq) provided after three days reaction time and subsequent flash column chromatography (SiO_2, CH:EE 4:1 v:v) **34** (38.9 mg, 97.2 µmol, 97 %) as a colorless solid.

R*f* = 0.23 (SiO_2, CH:EE 4:1 v:v). **Mp.:** 131 – 133 °C. **^{1}H-NMR (360 MHz, $CDCl_3$):** δ = 10.08 (s, 1H), 7.69 (d, $^4J_{HH}$ = 1.6 Hz, 2H), 7.34 (s, 1H), 7.24 – 7.19 (m, 1H), 5.93 (s, 2H), 2.08 – 2.02 (m, 3H), 1.96 (d, $^3J_{HH}$ = 2.9 Hz, 6H), 1.82 – 1.71 (m, 6H) ppm. **^{13}C{^{1}H}-NMR (91 MHz, $CDCl_3$):** δ = 192.6, 158.5, 138.6, 136.6, 133.1, 132.3, 131.8, 130.0, 119.5, 50.1, 42.6 (3x), 36.8 (3x), 32.8, 28.5 (3x) ppm. **IR (ATR, neat):** $\tilde{\nu}$ = 2906 (m), 2848 (m), 1694 (s), 1587 (m), 1558 (m), 1479 (w), 1456 (m), 1413 (w), 1385 (w), 1340 (m), 1309 (m), 1290 (m), 1212 (m), 1186 (m), 1170 (m), 1146 (m), 1077 (m), 1047 (m), 1001 (m), 976 (w), 888 (s), 852 (w), 804 (s), 754 (m), 723 (m), 701 (m), 683 (m), 661 (m) cm^{-1}. **MS (APCI):** m/z = 400.1 [$C_{20}H_{22}{}^{79}BrN_3O$+H]$^+$. **HR-MS (ESI):** calculated for $C_{20}H_{23}{}^{79}BrN_3O^+$ [M+H]$^+$: m/z = 400.10190, found: 400.10175 (Dev.: –0.15 mu; –0.37 ppm).

2.6.4 4-Bromo-2-((4-phenyl-1*H*-1,2,3-triazol-1-yl)methyl)benzaldehyde (35)

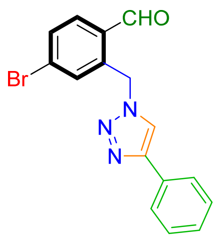

35

According to GP1, the reaction of **3** (24.0 mg, 100 µmol, 1.00 eq) and phenylacetylene (11.5 µL, 105 µmol, 1.05 eq) provided after 24 hours reaction time and subsequent flash column chromatography (SiO_2, CH:EE 3:1 v:v) **35** (33.8 mg, 98.7 µmol, 99 %) as a colorless solid.

R*f* = 0.27 (SiO_2, CH:EE 3:1 v:v). **Mp.:** 157 – 159 °C. **^{1}H-NMR (360 MHz, $CDCl_3$):** δ = 10.08 (d, $^4J_{HH}$ = 0.6 Hz, 1H), 7.92 (s, 1H), 7.87 – 7.79 (m, 2H), 7.72 – 7.70 (m, 2H), 7.44 – 7.38 (m, 2H), 7.37 – 7.35 (m, 1H), 7.35 – 7.30 (m, 1H), 6.02 (s, 2H) ppm. **^{13}C{^{1}H}-NMR (91 MHz, $CDCl_3$):** δ = 192.7, 148.1,

138.0, 136.9, 133.3, 132.5, 131.8, 130.5, 130.1, 129.0 (2x), 128.4, 125.8 (2x), 120.7, 50.4 ppm. **IR (ATR, neat):** $\tilde{\nu}$ = 3082 (w), 2876 (w), 2777 (w), 1686 (s), 1582 (m), 1563 (m), 1466 (m), 1433 (m), 1400 (w), 1349 (w), 1288 (w), 1214 (m), 1202 (m), 1172 (m), 1099 (w), 1084 (m), 1047 (m), 1029 (w), 978 (m), 915 (w), 865 (m), 833 (m), 804 (m), 781 (m), 765 (s), 714 (m), 692 (s), 671 (m), 657 (m) cm^{-1}. **MS (APCI):** m/z = 342.0 $[C_{16}H_{12}{}^{79}BrN_3O+H]^+$. **HR-MS (EI, 70 eV):** calculated for $C_{16}H_{12}{}^{79}BrN_3O^{+\cdot}$ $[M]^{+\cdot}$: m/z = 341.01583, found: 341.01570 (Dev.: –0.13 mu; –0.37 ppm).

2.6.5 4-Bromo-2-((4-(3,5-di-*tert*-butylphenyl)-1*H*-1,2,3-triazol-1-yl)methyl)benzaldehyde (36)

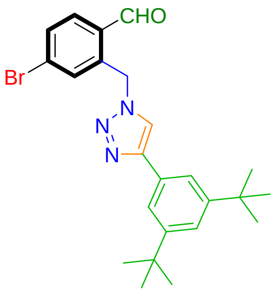

36

According to GP1, the reaction of **3** (24.0 mg, 100 µmol, 1.00 eq) and **32** (22.5 mg, 105 µmol, 1.05 eq) provided after three days reaction time and subsequent flash column chromatography (SiO_2, CH:EE 4:1 v:v) **36** (43.6 mg, 96.0 µmol, 96 %) as a colorless solid.

R_f = 0.31 (SiO_2, CH:EE 4:1 v:v). **Mp.:** 132 – 134 °C. **^{1}H-NMR (360 MHz, $CDCl_3$):** δ = 10.10 (s, 1H), 7.91 (s, 1H), 7.71 (d, $^5J_{HH}$ = 1.0 Hz, 2H), 7.69 (d, $^4J_{HH}$ = 1.8 Hz, 2H), 7.43 (t, $^4J_{HH}$ = 1.8 Hz, 1H), 7.31 (s, 1H), 6.06 (s, 2H), 1.37 (s, 18H) ppm. **^{13}C{^{1}H}-NMR (91 MHz, $CDCl_3$):** δ = 192.7, 151.5 (2x), 149.1, 138.3, 136.8, 133.0, 132.4, 131.8, 130.2, 129.6, 122.7, 120.6 (2x), 120.3, 50.4, 35.1 (2x), 31.6 (6x) ppm. **IR (ATR, neat):** $\tilde{\nu}$ = 2962 (m), 2903 (m), 2847 (w), 1698 (s), 1590 (m), 1562 (s), 1447 (m), 1419 (m), 1393 (m), 1361 (m), 1343 (m), 1287 (m), 1249 (m), 1225 (m), 1201 (m), 1183 (m), 1097 (m), 1080 (m), 1064 (m), 1051 (s), 1001 (w), 968 (m), 902 (s), 879 (s), 848 (m), 826 (s), 816 (s), 785 (s), 768 (s), 727 (s), 718 (s), 709 (s), 685 (m), 673 (m), 660 (m) cm^{-1}. **MS (APCI):** m/z = 454.0 $[C_{24}H_{28}{}^{79}BrN_3O+H]^+$. **HR-MS (ESI):** calculated for $C_{24}H_{29}{}^{79}BrN_3O^+$ $[M+H]^+$: m/z = 454.14885, found: 454.14826 (Dev.: –0.59 mu; –1.30 ppm).

2.6.6 2-Bromo-4-((4-octyl-1*H*-1,2,3-triazol-1-yl)methyl)benzaldehyde (37)

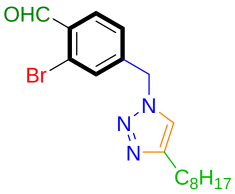

37

According to GP1, the reaction of **4** (24.0 mg, 100 µmol, 1.00 eq) and 1-decyne (19.0 µL, 105 µmol, 1.05 eq) provided after 24 hours reaction time and subsequent flash column chromatography (SiO_2, CH:EE 3:1 v:v) **37** (34.7 mg, 91.7 µmol, 92 %) as a colorless solid.

R$_f$ = 0.14 (SiO_2, CH:EE 3:1 v:v). **Mp.:** 75 – 77 °C. **^{1}H-NMR (360 MHz, $CDCl_3$):** δ = 10.30 (d, $^4J_{HH}$ = 0.8 Hz, 1H), 7.87 (d, $^3J_{HH}$ = 8.0 Hz, 1H), 7.50 – 7.44 (m, 1H), 7.30 – 7.21 (m, 2H), 5.53 (s, 2H), 2.70 (t, $^3J_{HH}$ = 7.7 Hz, 2H), 1.64 (p, $^3J_{HH}$ = 7.9 Hz, 2H), 1.36 – 1.19 (m, 10H), 0.84 (t, $^3J_{HH}$ = 6.8 Hz, 3H) ppm. **^{13}C{^{1}H}-NMR (91 MHz, $CDCl_3$):** δ = 191.1, 149.6, 142.9, 133.5, 132.8, 130.5, 127.6, 127.1, 120.9, 52.8, 31.9, 29.4, 29.4, 29.3, 29.3, 25.8, 22.7, 14.2 ppm. **IR (ATR, neat):** $\tilde{\nu}$ = 3061 (w), 2951 (w), 2918 (m), 2848 (m), 1687 (s), 1600 (m), 1558 (m), 1466 (m), 1432 (m), 1408 (m), 1389 (m), 1349 (m), 1291 (w), 1260 (m), 1202 (s), 1137 (m), 1079 (w), 1052 (m), 1036 (m), 977 (w), 889 (w), 866 (m), 826 (m), 805 (s), 781 (s), 765 (m), 721 (m), 692 (m), 674 (m) cm^{-1}. **MS (APCI):** m/z = 378.1 [$C_{18}H_{24}{}^{79}BrN_3O$+H]$^+$. **HR-MS (EI, 70 eV):** calculated for $C_{18}H_{24}{}^{79}BrN_3O^{+\cdot}$ [M]$^{+\cdot}$: m/z = 377.10973, found: 377.10955 (Dev.: –0.18 mu; –0.47 ppm).

2.6.7 4-((4-((1*s*,3*s*)-Adamantan-1-yl)-1*H*-1,2,3-triazol-1-yl)methyl)-2-bromobenzaldehyde (38)

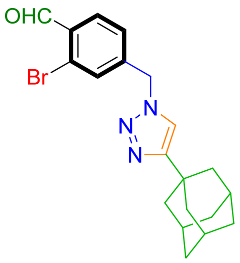

38

According to GP1, the reaction of **4** (24.0 mg, 100 µmol, 1.00 eq) and **31** (16.8 mg, 105 µmol, 1.05 eq) provided after 24 hours reaction time and subsequent flash column chromatography (SiO_2, CH:EE 3:1 v:v) **38** (38.7 mg, 96.7 µmol, 97 %) as a colorless solid.

R$_f$ = 0.24 (SiO_2, CH:EE 3:1 v:v). **Mp.:** 99 – 101 °C. **^{1}H-NMR (360 MHz, $CDCl_3$):** δ = 10.31 (s, 1H), 7.88 (d, $^3J_{HH}$ = 8.0 Hz, 1H), 7.51 (d, $^4J_{HH}$ = 1.7 Hz, 1H), 7.26 (dd, $^3J_{HH}$ = 8.2 Hz, $^4J_{HH}$ = 1.6 Hz, 1H), 7.21 (s, 1H), 5.52 (s, 2H), 2.08 – 2.00 (m, 3H), 1.95 (d, $^3J_{HH}$ = 2.9 Hz, 6H), 1.81 – 1.70 (m, 6H) ppm. **^{13}C{1H}-NMR (91 MHz, $CDCl_3$):** δ = 191.2, 159.1, 142.9, 133.5, 133.0, 130.6, 127.6, 127.3, 118.7, 52.8, 42.6 (3x), 36.7 (3x), 32.8, 28.5 (3x) ppm. **IR (ATR, neat):** $\tilde{\nu}$ = 2900 (s), 2847 (m), 1689 (s), 1599 (m), 1562 (w), 1539 (w), 1450 (w), 1406 (w), 1387 (m), 1343 (w), 1303 (w), 1286 (w), 1266 (m), 1205 (s), 1140 (w), 1101 (w), 1073 (w), 1042 (s), 1002 (m), 975 (w), 925 (w), 898 (w), 845 (m), 811 (w), 788 (s), 771 (m), 755 (s), 724 (s), 676 (m) cm^{-1}. **MS (APCI):** m/z = 400.0 [$C_{20}H_{22}{}^{79}BrN_3O$+H]$^+$. **HR-MS**

(ESI): calculated for $C_{20}H_{23}{}^{79}BrN_3O^+$ $[M+H]^+$: m/z = 400.10190, found: 400.10166 (Dev.: –0.24 mu; –0.59 ppm).

2.6.8 2-Bromo-4-((4-phenyl-1*H*-1,2,3-triazol-1-yl)methyl)benzaldehyde (39)

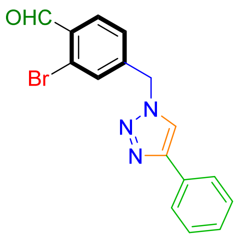

39

According to GP1, the reaction of **4** (24.0 mg, 100 µmol, 1.00 eq) and phenylacetylene (11.5 µL, 105 µmol, 1.05 eq) provided after 24 hours reaction time and subsequent flash column chromatography (SiO_2, CH:EE 3:1 v:v) **39** (33.1 mg, 96.7 µmol, 97 %) as a colorless solid.

R*f* = 0.13 (SiO_2, CH:EE 3:1 v:v). **Mp.:** 141 – 143 °C. **^{1}H-NMR (360 MHz, $CDCl_3$):** δ = 10.32 (d, $^4J_{HH}$ = 0.8 Hz, 1H), 7.90 (d, $^3J_{HH}$ = 8.0 Hz, 1H), 7.86 – 7.77 (m, 2H), 7.77 (s, 1H), 7.55 (d, $^4J_{HH}$ = 1.6 Hz, 1H), 7.44 – 7.39 (m, 2H), 7.37 – 7.30 (m, 2H), 5.61 (s, 2H) ppm. **^{13}C{^{1}H}-NMR (91 MHz, $CDCl_3$):** δ = 191.1, 148.8, 142.5, 133.7, 133.0, 130.6, 130.2, 129.0 (2x), 128.6, 127.7, 127.2, 125.8 (2x), 119.8, 53.0 ppm. **IR (ATR, neat):** $\tilde{\nu}$ = 3084 (w), 2858 (w), 1687 (m), 1584 (m), 1563 (w), 1465 (m), 1426 (w), 1403 (m), 1383 (w), 1344 (w), 1224 (m), 1201 (m), 1179 (m), 1148 (w), 1084 (m), 1047 (m), 1039 (m), 978 (m), 914 (w), 865 (w), 845 (w), 825 (m), 805 (m), 782 (m), 763 (s), 710 (m), 692 (s), 677 (m), 654 (m) cm^{-1}. **MS (APCI):** m/z = 342.0 $[C_{16}H_{12}{}^{79}BrN_3O+H]^+$. **HR-MS (EI, 70 eV):** calculated for $C_{16}H_{12}{}^{79}BrN_3O^{+\cdot}$ $[M]^{+\cdot}$: m/z = 341.01583, found: 341.01565 (Dev.: –0.18 mu; –0.51 ppm).

2.6.9 2-Bromo-4-((4-(3,5-di-*tert*-butylphenyl)-1*H*-1,2,3-triazol-1-yl)methyl)benzaldehyde (40)

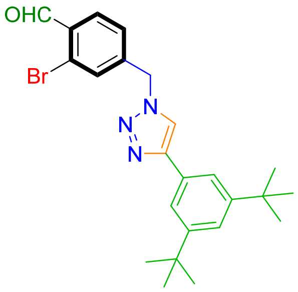

40

According to GP1, the reaction of **4** (24.0 mg, 100 µmol, 1.00 eq) and **32** (22.5 mg, 105 µmol, 1.05 eq) provided after 24 hours reaction time and subsequent flash column chromatography (SiO_2, CH:EE 3:1 v:v) **40** (42.9 mg, 94.4 µmol, 94 %) as a colorless solid.

R$_f$ = 0.34 (SiO_2, CH:EE 3:1 v:v). **Mp.:** 190 – 192 °C. **^{1}H-NMR (360 MHz, $CDCl_3$):** δ = 10.36 (s, 1H), 7.94 (d, $^3J_{HH}$ = 8.0 Hz, 1H), 7.82 (s, 1H), 7.70 (d, $^4J_{HH}$ = 1.8 Hz, 2H), 7.58 (d, $^4J_{HH}$ = 1.7 Hz, 1H), 7.46 (t, $^4J_{HH}$ = 1.9 Hz, 1H), 7.35 (ddd, $^3J_{HH}$ = 7.8 Hz, $^4J_{HH}$ = 1.6 Hz, $^5J_{HH}$ = 0.8 Hz, 1H), 5.66 (s, 2H), 1.39 (s, 18H) ppm. **^{13}C{1H}-NMR (91 MHz, $CDCl_3$):** δ = 191.2, 151.6 (2x), 149.7, 142.7, 133.6, 132.9, 130.6, 129.4, 127.7, 127.1, 122.9, 120.3 (2x), 119.8, 53.0, 35.1 (2x), 31.5 (6x) ppm. **IR (ATR, neat):** $\tilde{\nu}$ = 3104 (w), 3067 (w), 2961 (m), 2865 (w), 1699 (s), 1599 (m), 1546 (w), 1474 (w), 1463 (w), 1444 (w), 1420 (w), 1405 (w), 1386 (m), 1363 (m), 1279 (w), 1264 (m), 1248 (m), 1224 (m), 1199 (m), 1139 (w), 1099 (w), 1054 (m), 1042 (m), 1008 (w), 882 (m), 839 (m), 781 (s), 745 (m), 714 (s), 677 (m) cm^{-1}. **MS (APCI):** m/z = 454.1 [$C_{24}H_{28}{}^{79}BrN_3O+H]^+$. **HR-MS (ESI):** calculated for $C_{24}H_{29}{}^{79}BrN_3O^+$ $[M+H]^+$: m/z = 454.14885, found: 454.14914 (Dev.: 0.28 mu; 0.63 ppm).

2.6.10 7-Bromo-9,9-dimethyl-3-((4-octyl-1*H*-1,2,3-triazol-1-yl)methyl)-9*H*-fluorene-2-carbaldehyde (41)

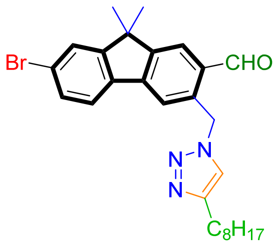

41

According to GP1, the reaction of **5** (35.6 mg, 100 µmol, 1.00 eq) and 1-decyne (19.0 µL, 105 µmol, 1.05 eq) provided after 24 hours reaction time and subsequent flash column chromatography (SiO_2, CH:EE 3:1 v:v) **41** (45.2 mg, 91.4 µmol, 91 %) as a yellowish oil.

R$_f$ = 0.22 (SiO_2, CH:EE 3:1 v:v). **^{1}H-NMR (360 MHz, $CDCl_3$):** δ = 10.18 (s, 1H), 7.86 (s, 1H), 7.59 (d, $^4J_{HH}$ = 1.7 Hz, 1H), 7.54 (d, $^3J_{HH}$ = 8.1 Hz, 1H), 7.48 (dd, $^3J_{HH}$ = 8.1 Hz, $^4J_{HH}$ = 1.7 Hz, 1H), 7.46 – 7.44 (m, 2H), 6.04 (s, 2H), 2.71 (t, $^3J_{HH}$ = 7.6 Hz, 2H), 1.66 (p, $^3J_{HH}$ = 7.4 Hz, 2H), 1.51 (s, 6H), 1.39 – 1.18 (m, 10H), 0.85 (t, $^3J_{HH}$ = 6.8 Hz, 3H) ppm. **^{13}C{^{1}H}-NMR (91 MHz, $CDCl_3$):** δ = 192.7, 156.6, 153.2, 148.5, 143.9, 136.4, 135.8, 131.9, 130.5, 129.3, 126.2, 123.3, 122.6, 121.5, 121.4, 50.6, 47.0, 31.6, 29.2, 29.1, 29.0, 29.0, 26.4 (2x), 25.5, 22.4, 13.9 ppm. **IR (ATR, neat):** $\tilde{\nu}$ = 2923 (m), 2853 (m), 1684 (s), 1614 (m), 1600 (m), 1561 (m), 1457 (m), 1405 (m), 1362 (w), 1251 (m), 1217 (w), 1179 (s), 1047 (m), 966 (m), 907 (w), 876 (w), 821 (m), 800 (m), 771 (m), 732 (s), 672 (w) cm^{-1}. **MS (APCI):** m/z = 494.1 [$C_{27}H_{32}{}^{79}BrN_3O+H]^+$. **HR-MS (EI, 70 eV):** calculated for $C_{27}H_{32}{}^{79}BrN_3O^{+\cdot}$ $[M]^{+\cdot}$: m/z = 493.17233, found: 493.17229 (Dev.: –0.04 mu; –0.07 ppm). The analytical data are in accordance with the literature.[26]

2.6.11 3-((4-((3*r*,5*r*,7*r*)-Adamantan-1-yl)-1*H*-1,2,3-triazol-1-yl)methyl)-7-bromo-9,9-dimethyl-9*H*-fluorene-2-carbaldehyde (42)

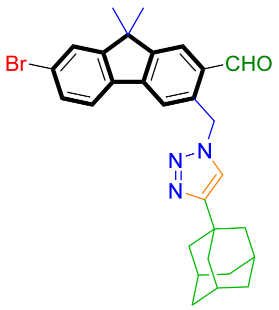

42

According to GP1, **5** (107 mg, 300 µmol, 1.00 eq) and **31** (50.5 mg, 315 µmol, 1.05 eq) were dissolved in $CHCl_3$ (3.00 mL, 0.1 M). $CuSO_4 \cdot 5\ H_2O$ (7.2 mg, 30 µmol, 0.10 eq) and sodium ascorbate (29.7 mg, 150 µmol, 0.50 eq) were suspended separately in water (375 µL) and added to the reaction vial. The heterogeneous mixture was stirred vigorously at 25 °C for three days and directly purified by flash column chromatography (SiO_2, CH:EE 3:1 v:v) **42** (153 mg, 296 µmol, 99 %) as a colorless oil, which solidified upon standing at 25 °C.

R_f = 0.33 (SiO_2, CH:EE 3:1 v:v). **Mp.:** 123 – 125 °C. **^{1}H-NMR (360 MHz, $CDCl_3$):** δ = 10.18 (s, 1H), 7.86 (s, 1H), 7.59 (s, 1H), 7.53 (d, $^3J_{HH}$ = 8.0 Hz, 1H), 7.49 (d, $^3J_{HH}$ = 7.9 Hz, 1H), 7.45 (s, 1H), 7.39 (s, 1H), 6.03 (s, 2H), 2.04 (s, 3H), 1.97 (s, 6H), 1.75 (s, 6H), 1.50 (s, 6H) ppm. **^{13}C{^{1}H}-NMR (91 MHz, $CDCl_3$):** δ = 193.1, 158.4, 157.0, 153.5, 144.2, 136.8, 136.2, 132.3, 130.9, 129.5, 126.6, 123.6, 122.9, 122.1, 119.4, 50.8, 47.4, 42.6 (3x), 36.8 (3x), 32.8, 28.5 (3x), 26.7 (2x) ppm. **IR (ATR, neat):** $\tilde{\nu}$ = 2903 (m), 2847 (m), 1677 (s), 1615 (m), 1563 (m), 1448 (m), 1427 (m), 1408 (w), 1361 (w), 1344 (w), 1312 (w), 1253 (m), 1213 (w), 1182 (m), 1167 (m), 1128 (w), 1101 (w), 1081 (w), 1063 (m), 1049 (m), 1001 (w), 967 (m), 905 (m), 880 (w), 827 (s), 806 (m), 789 (m), 767 (m), 726 (s), 672 (m) cm^{-1}. **MS (APCI):** m/z = 516.1 [$C_{29}H_{30}{}^{79}BrN_3O$+H]$^+$. **HR-MS (APCI):** calculated for $C_{29}H_{31}{}^{79}BrN_3O^+$ [M+H]$^+$: m/z = 516.16450, found: 516.16445 (Dev.: –0.05 mu; –0.10 ppm).

2.6.12 7-Bromo-9,9-dimethyl-3-((4-phenyl-1*H*-1,2,3-triazol-1-yl)methyl)-9*H*-fluorene-2-carbaldehyde (43)

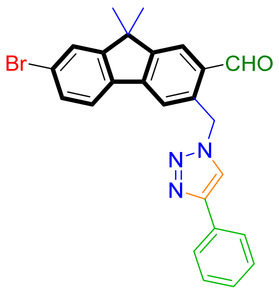

43

According to GP1, the reaction of **5** (35.6 mg, 100 µmol, 1.00 eq) and phenylacetylene (11.5 µL, 105 µmol, 1.05 eq) provided after 48 hours reaction time and subsequent flash column chromatography (SiO_2, CH:EE 3:1 v:v) **43** (42.9 mg, 93.6 µmol, 94 %) as a colorless foam.

R$_f$ = 0.24 (SiO_2, CH:EE 3:1 v:v). **Mp.:** 76 – 86 °C (very long melting). **^{1}H-NMR (360 MHz, $CDCl_3$):** δ = 10.19 (s, 1H), 7.98 (s, 1H), 7.87 (s, 1H), 7.88 – 7.79 (m, 2H), 7.61 – 7.59 (m, 2H), 7.57 (d, $^3J_{HH}$ = 8.2 Hz, 1H), 7.48 (dd, $^3J_{HH}$ = 8.2 Hz, $^4J_{HH}$ = 1.7 Hz, 1H), 7.43 – 7.37 (m, 2H), 7.34 – 7.28 (m, 1H), 6.13 (s, 2H), 1.51 (s, 6H) ppm. **^{13}C{^{1}H}-NMR (91 MHz, $CDCl_3$):** δ = 192.9, 156.6, 153.4, 147.7, 144.0, 135.8, 135.7, 132.0, 130.6, 130.3, 129.6, 128.6 (2x), 127.9, 126.2, 125.5 (2x), 123.4, 122.7, 122.0, 120.4, 50.8, 47.1, 26.4 (2x) ppm. **IR (ATR, neat):** $\tilde{\nu}$ = 2961 (w), 2924 (w), 2858 (w), 1683 (s), 1614 (m), 1600 (w), 1561 (m), 1463 (w), 1406 (w), 1251 (m), 1179 (m), 1076 (m), 1045 (m), 965 (m), 907 (w), 822 (m), 810 (m), 763 (s), 732 (s), 694 (s), 672 (m) cm^{-1}. **MS (APCI):** m/z = 458.0 [$C_{25}H_{20}{}^{79}BrN_3O$+H]$^+$. **HR-MS (EI, 70 eV):** calculated for $C_{25}H_{20}{}^{79}BrN_3O^{+\cdot}$ [M]$^{+\cdot}$: m/z = 457.07843, found: 457.07831 (Dev.: –0.12 mu; –0.25 ppm).

2.6.13 7-Bromo-3-((4-(3,5-di-*tert*-butylphenyl)-1*H*-1,2,3-triazol-1-yl)methyl)-9,9-dimethyl-9*H*-fluorene-2-carbaldehyde (44)

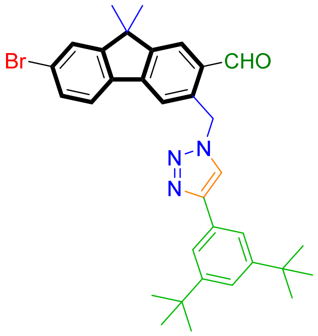

44

According to GP1, the reaction of **5** (35.6 mg, 100 µmol, 1.00 eq) and **32** (22.5 mg, 105 µmol, 1.05 eq) provided after six days reaction time and subsequent flash column chromatography (SiO_2, CH:EE 3:1 v:v) **44** (55.9 mg, 98.0 µmol, 98 %) as a colorless solid.

R$_f$ = 0.47 (SiO_2, CH:EE 3:1 v:v). **Mp.:** 210 – 212 °C. **^{1}H-NMR (360 MHz, $CDCl_3$):** δ = 10.21 (s, 1H), 7.97 (s, 1H), 7.88 (s, 1H), 7.70 (d, J = 1.8 Hz, 2H), 7.60 (d, J = 1.7 Hz, 1H), 7.57 (d, J = 8.2 Hz, 1H), 7.54 (s, 1H), 7.48 (dd, J = 8.2, 1.8 Hz, 1H), 7.42 (t, J = 1.8 Hz, 1H), 6.16 (s, 2H), 1.52 (s, 6H), 1.36 (s, 18H) ppm. **^{13}C{^{1}H}-NMR (91 MHz, $CDCl_3$):** δ = 193.3, 157.0, 153.6, 151.4 (2x), 149.0, 144.3, 136.6, 136.1, 132.2, 130.9, 129.8 (2x), 126.6, 123.7, 123.1, 122.6, 121.8, 120.6, 120.2 (2x), 51.1, 47.4, 35.1 (2x), 31.6 (6x), 26.7 (2x) ppm. **IR (ATR, neat):** $\tilde{\nu}$ = 2960 (m), 2903 (w), 2866 (w), 1686 (s), 1613 (m), 1600 (m), 1563 (m), 1462 (m), 1420 (m), 1393 (m), 1362 (s), 1281 (m), 1250 (s), 1224 (m), 1179 (s), 1091 (m), 1047 (m), 967 (m), 900 (m), 876 (s), 847 (m), 821 (s), 810 (s), 730 (s), 708 (s), 673 (m), 661

(m) cm^{-1}. **MS (APCI):** m/z = 570.1 $[C_{33}H_{36}{}^{79}BrN_3O+H]^+$. **HR-MS (APCI):** calculated for $C_{33}H_{37}{}^{79}BrN_3O^+$ $[M+H]^+$: m/z = 570.21145, found: 570.21154 (Dev.: 0.09 mu; 0.15 ppm).

2.7 Methylation Reaction of Adamantyl substituted triazole (42)

2.7.1 4-((3*r*,5*r*,7*r*)-Adamantan-1-yl)-1-((7-bromo-2-formyl-9,9-dimethyl-9*H*-fluoren-3-yl)methyl)-3-methyl-1*H*-1,2,3-triazol-3-ium tetrafluoroborate (45)

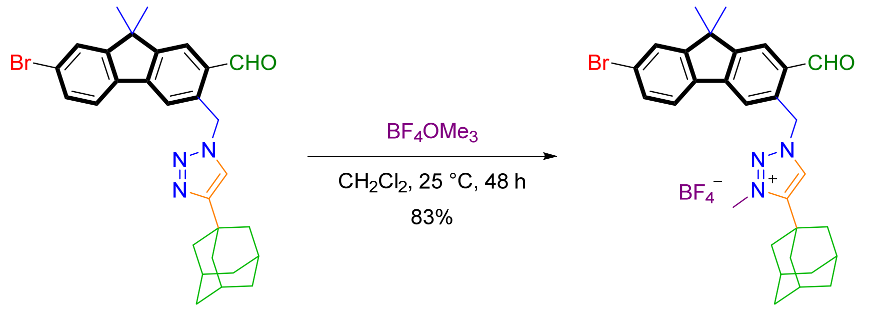

A modified literature procedure was used.[40] A heat gun-dried and nitrogen-flushed *Schlenk* flask, equipped with a magnetic stirring bar and a rubber septum, was charged with **42** (25.8 mg, 50.0 µmol, 1.00 eq) and dissolved in anhydrous CH_2Cl_2 (3.33 mL, 15 mM). Trimethyloxonium tetrafluoroborate (7.40 mg, 50.0 µmol, 1.00 eq) was added in one portion at 25 °C whereupon the mixture turned slightly yellow. After stirring for two days under these conditions, methanol (1.0 mL) was added and the mixture was stirred for 30 minutes under air. All volatile components were removed under reduced pressure and the oily yellow residue was purified by flash column chromatography (CH_2Cl_2:MeOH 15:1 v:v) to provide **45** (25.7 mg, 41.6 µmol, 83 %) as a colorless solid. If desired the product can be precipitated by dissolving in CH_2Cl_2 and addition of diethyl ether until a white precipitate is formed.

R*f* = 0.33 (SiO_2, DCM:MeOH 15:1 v:v). **Mp.:** 249 – 251 °C decomp. **^{1}H-NMR (360 MHz, $CDCl_3$):** δ = 10.10 (s, 1H), 8.22 (s, 1H), 8.20 (s, 1H), 7.92 (s, 1H), 7.86 (d, $^3J_{HH}$ = 8.1 Hz, 1H), 7.59 (d, $^4J_{HH}$ = 1.7 Hz, 1H), 7.52 (dd, $^3J_{HH}$ = 8.2 Hz, $^4J_{HH}$ = 1.8 Hz, 1H), 6.17 (s, 2H), 4.31 (s, 3H), 2.11 (s, 3H), 2.07 – 2.02 (m, 6H), 1.78 – 1.72 (m, 6H), 1.53 (s, 6H) ppm. **^{13}C{^{1}H}-NMR (91 MHz, $CDCl_3$):** δ = 193.1, 158.7, 156.8, 155.4, 151.0, 145.0, 136.0, 133.0, 131.2, 130.7, 130.0, 128.2, 126.4, 126.3, 124.0, 54.7, 47.6, 41.0, 39.5 (3x), 35.8 (3x), 33.8, 27.7 (3x), 26.6 (2x) ppm. **IR (ATR, neat):** $\tilde{\nu}$ = 2909 (m), 2850 (w), 2734 (w), 1703 (s), 1615 (w), 1600 (w), 1562 (m), 1456 (w), 1406 (w), 1380 (w), 1356 (w), 1325 (w), 1311 (w), 1284 (w), 1251 (w), 1251 (w), 1231 (w), 1180 (m), 1166 (w), 1137 (w), 1106 (m), 1057 (s), 1026 (s), 963 (m), 884 (m), 867 (w), 827 (s), 811 (m), 785 (m), 773 (m), 736 (m), 700 (w), 674 (w), 655 (w) cm^{-1}. **MS (ESI):** m/z = 530.2 $[C_{30}H_{33}{}^{79}BrN_3O]^+$. **HR-MS (ESI):** calculated for $C_{30}H_{33}{}^{79}BrN_3O^+$ $[M]^+$: m/z = 530.18015, found: 530.17960 (Dev.: –0.55 mu; –1.04 ppm).

3 Optical Properties

3.1 2-(Azidomethyl)-4-bromobenzaldehyde (3) & Derivatives

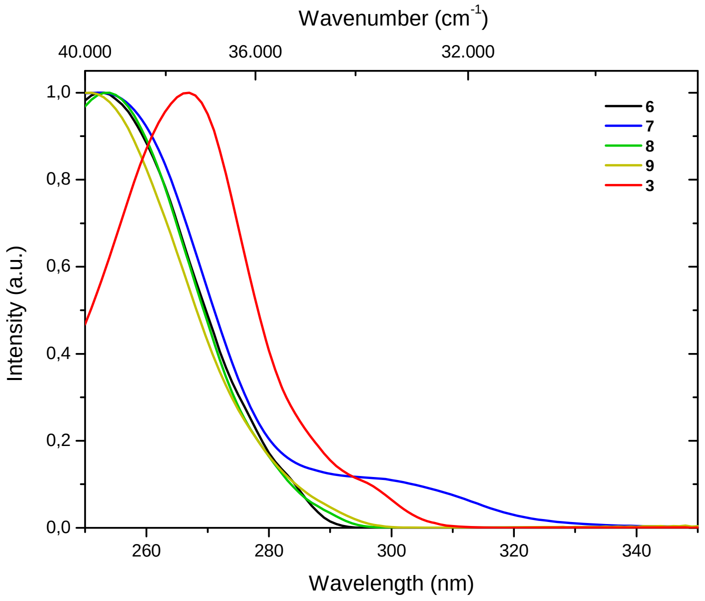

Figure S1: Normalized absorption spectra of **6** (black line), **7** (blue line), **8** (green line), **9** (yellow line) and **3** (red line) in chloroform solution ($c = 10^{-5}$ mol L^{-1}).

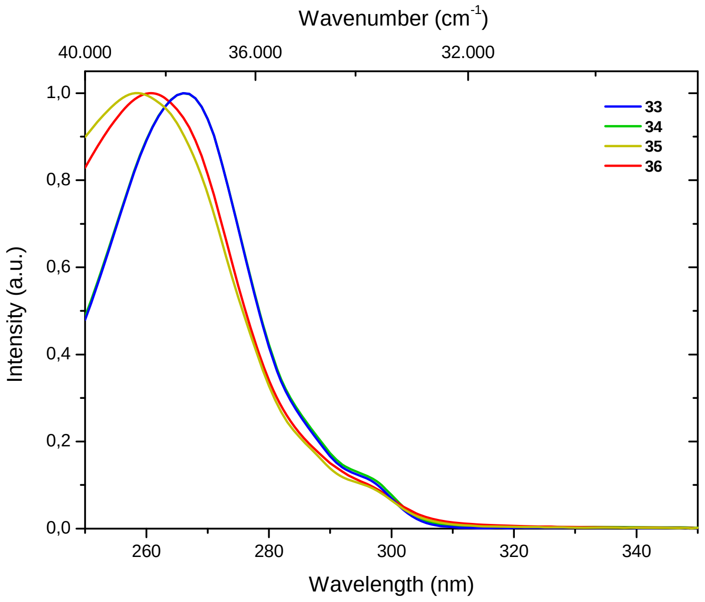

Figure S2: Normalized absorption spectra of **33** (blue line), **34** (green line), **35** (yellow line) and **36** (red line) in chloroform solution ($c = 10^{-5}$ mol L^{-1}).

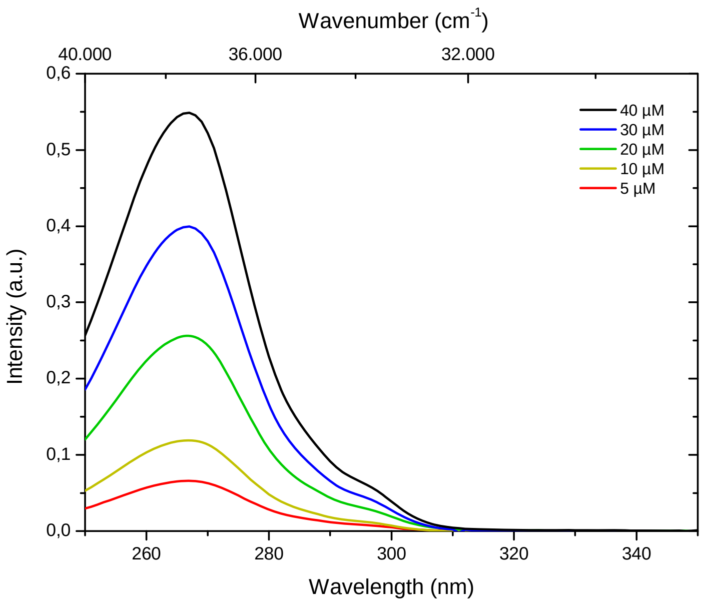

Figure S3: Absorption spectra of **3** at various concentration in chloroform solution.

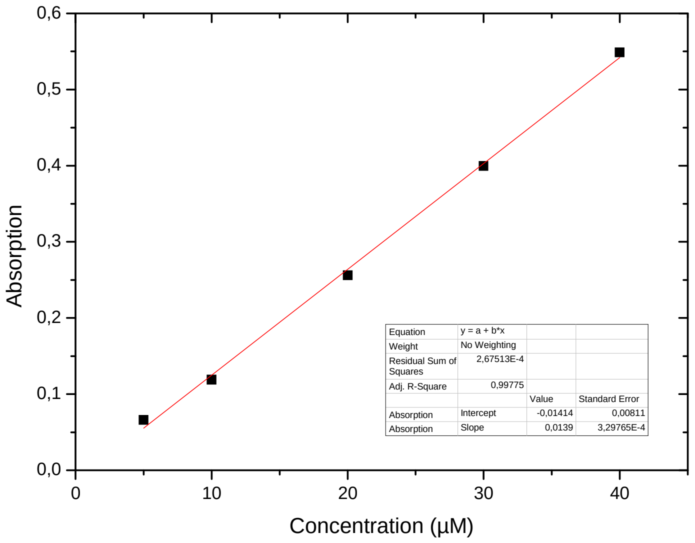

Figure S4: Linear regression for extinction coefficient determination (with respect to Figure S3): ε_{267} = 13.900 L mol^{-1} cm^{-1}.

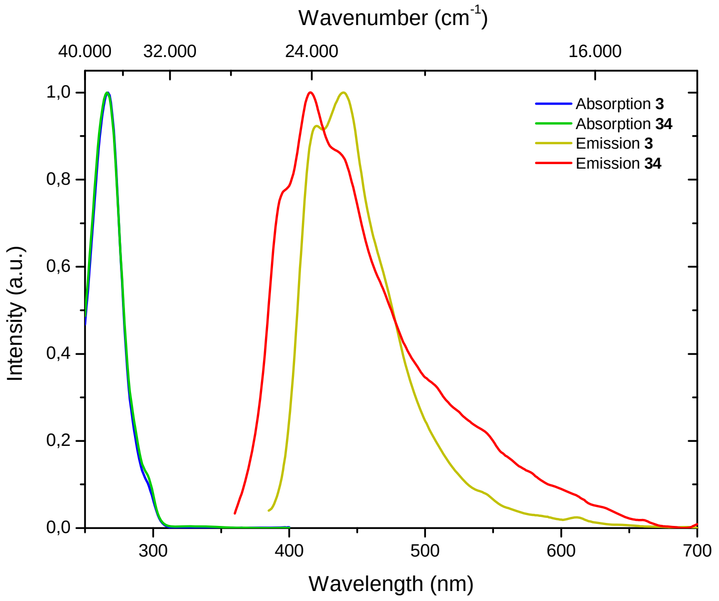

Figure S5: Normalized absorption spectra of **3** (blue line) and **34** (green line) in chloroform solution (c = 10^{-5} mol L^{-1}) after bubbling with argon for 30 minutes and normalized emission spectra of **3** (yellow line), **34** (red line) in the solid-state.

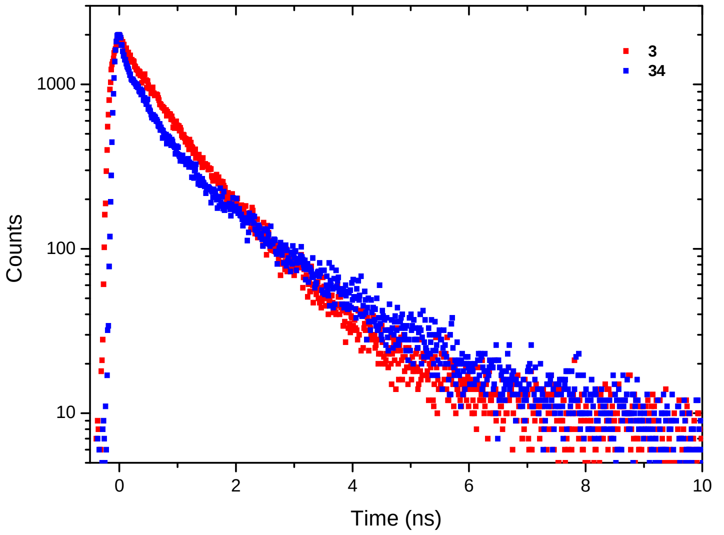

Figure S6: Fluorescence decay profile of **3** and **34** in the solid-state with excitation at 376 nm.

3: τ_1 = 0.796 ± 0.006 (84.4%) ns; τ_2 = 3.228 ± 0.115 (15.6%) ns; χ^2 = 1.081.

34: τ_1 = 0.516 ± 0.006 (50.2%) ns; τ_2 = 2.212 ± 0.022 (49.8%) ns; χ^2 = 1.069.

3.2 4-(Azidomethyl)-2-bromobenzaldehyde (4) & Derivatives

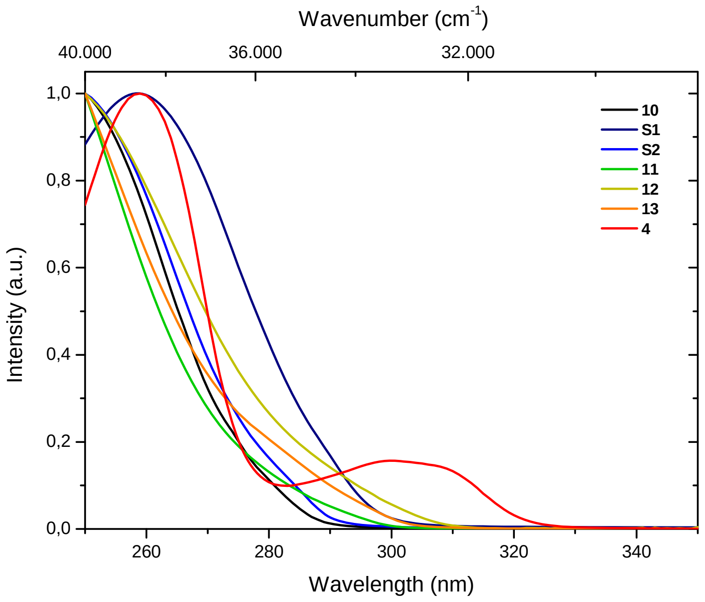

Figure S7: Normalized absorption spectra of **10** (black line), **S1** (navy line), **S2** (blue line), **11** (green line), **12** (yellow line), **13** (orange line) and **4** (red line) in chloroform solution ($c = 10^{-5}$ mol L^{-1}).

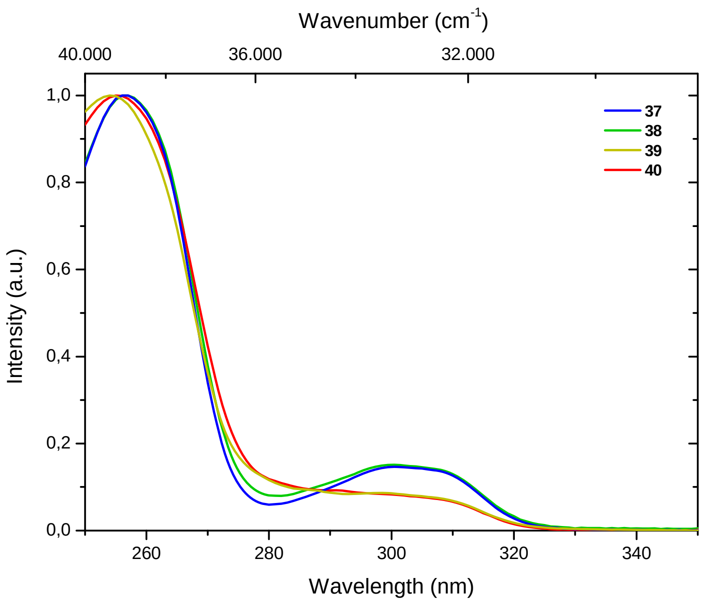

Figure S8: Normalized absorption spectra of **37** (blue line), **38** (green line), **39** (yellow line), **40** (red line) in chloroform solution ($c = 10^{-5}$ mol L^{-1}).

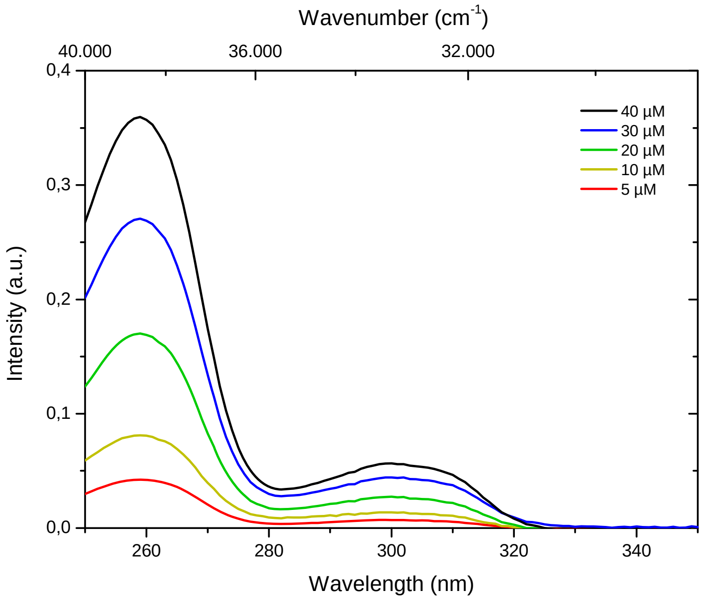

Figure S9: Absorption spectra of **4** at various concentration in chloroform solution.

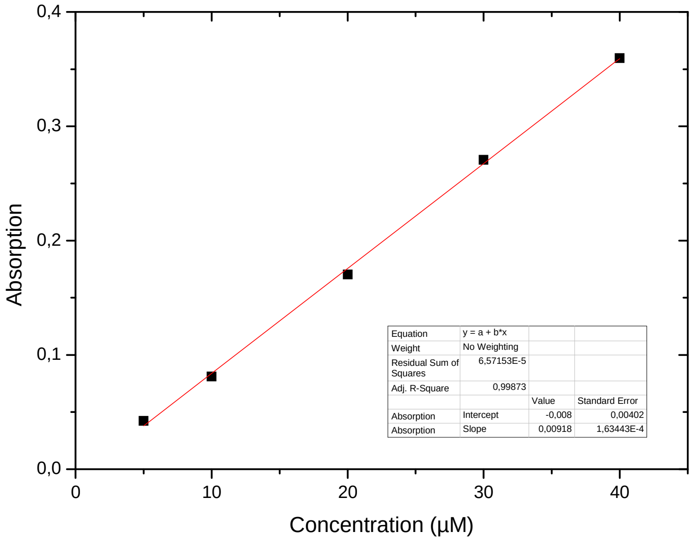

Figure S10: Linear regression for extinction coefficient determination (with respect to Figure S9): ε_{259} = 9.180 L mol^{-1} cm^{-1}.

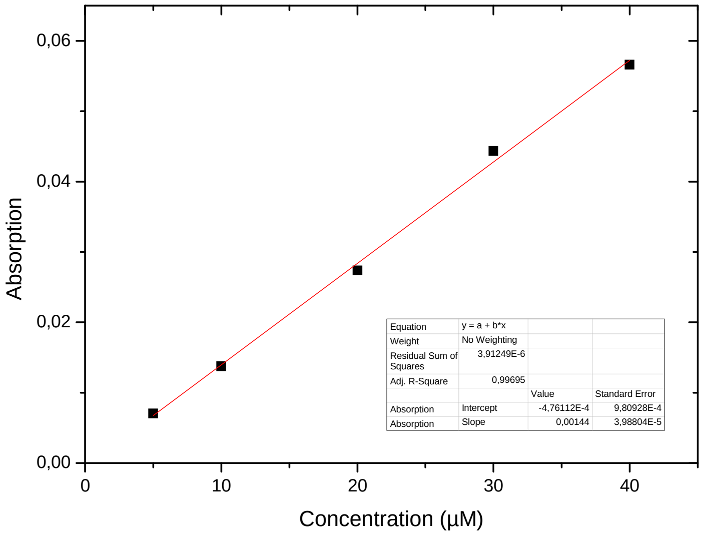

Figure S11: Linear regression for extinction coefficient determination (with respect to Figure S9): ε_{300} = 1.440 L mol^{-1} cm^{-1}.

3.3 3-(Azidomethyl)-7-bromo-9,9-dimethyl-9*H*-fluorene-2-carbaldehyde (5) & Derivatives

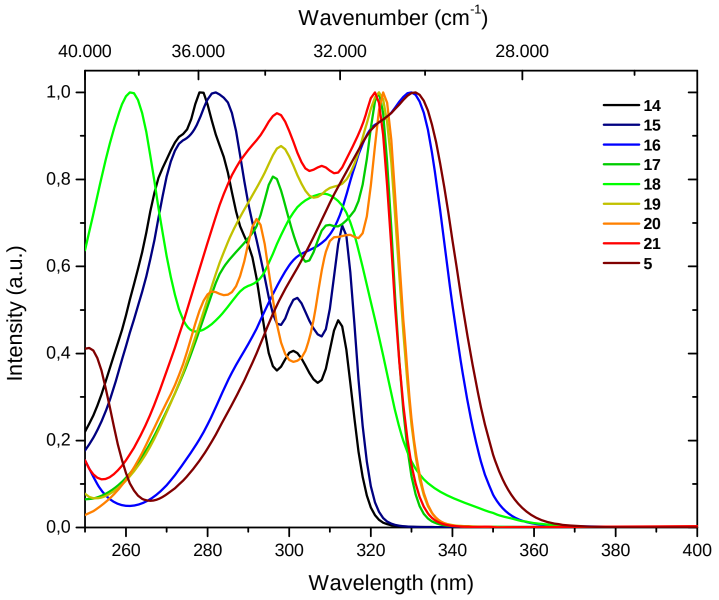

Figure S12: Normalized absorption spectra of **14** (black line), **15** (navy line), **16** (blue line), **17** (green line), **18** (light green line), **19** (yellow line), **20** (orange line), **21** (red line) and **6** (wine line) in chloroform solution (c = 10^{-5} mol L^{-1}).

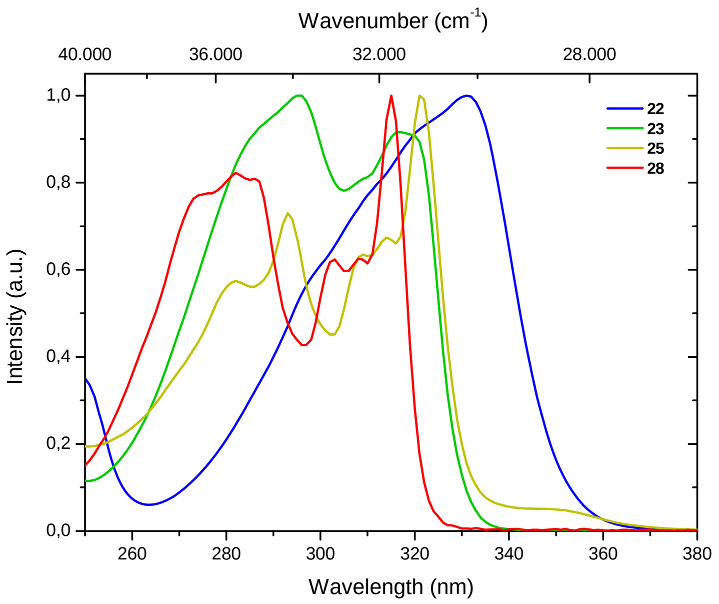

Figure S13: Normalized absorption spectra of **22** (blue line), **23** (green line), **25** (yellow line), **28** (red line) in chloroform solution ($c = 10^{-5}$ mol L^{-1}).

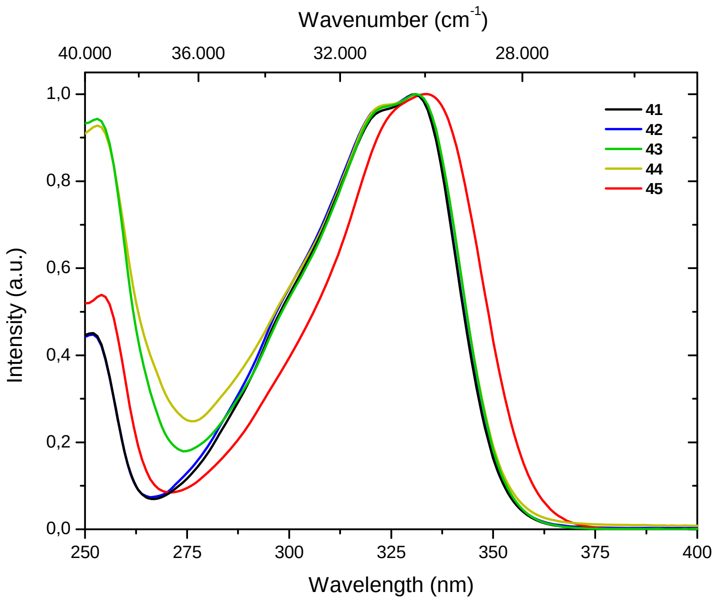

Figure S14: Normalized absorption spectra of **41** (black line), **42** (blue line), **43** (green line), **44** (yellow line), **45** (red line) in chloroform solution ($c = 10^{-5}$ mol L^{-1}).

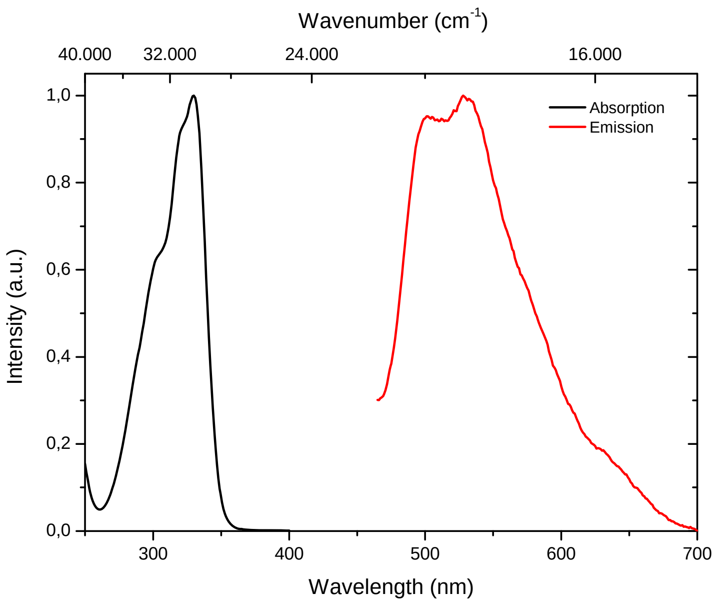

Figure S15: Normalized absorption (black line) and emission (red line) spectra of **16** in chloroform solution ($c = 10^{-5}$ mol L^{-1}) after bubbling with argon for 30 minutes. Emission was monitored by excitation at the absorption maximum wavelength of 330 nm. Emission maximum at 528 nm.

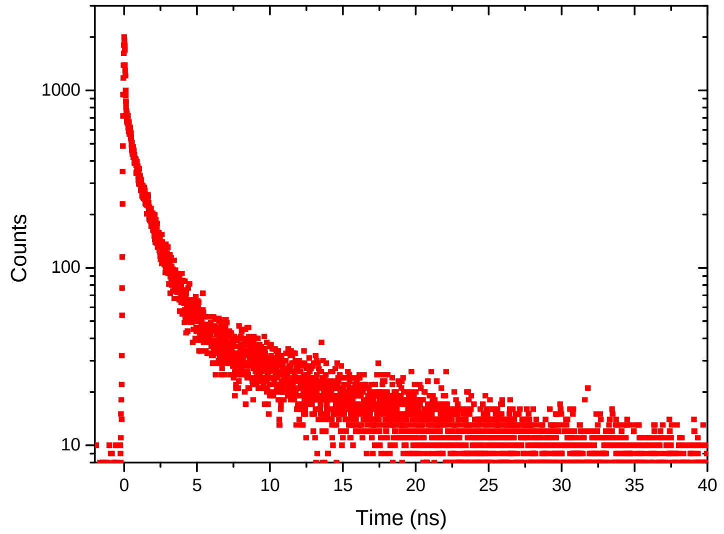

Figure S16: Fluorescence decay profile of **16** in chloroform solution ($c = 10^{-5}$ mol L^{-1}) after bubbling with argon for 30 minutes with excitation at 376 nm: $\tau_1 = 1.186 \pm 0.010$ (54.7%) ns; $\tau_2 = 11.50 \pm 0.26$ (45.3%) ns; $\chi^2 = 1.247$. Emission was detected at 530 nm.

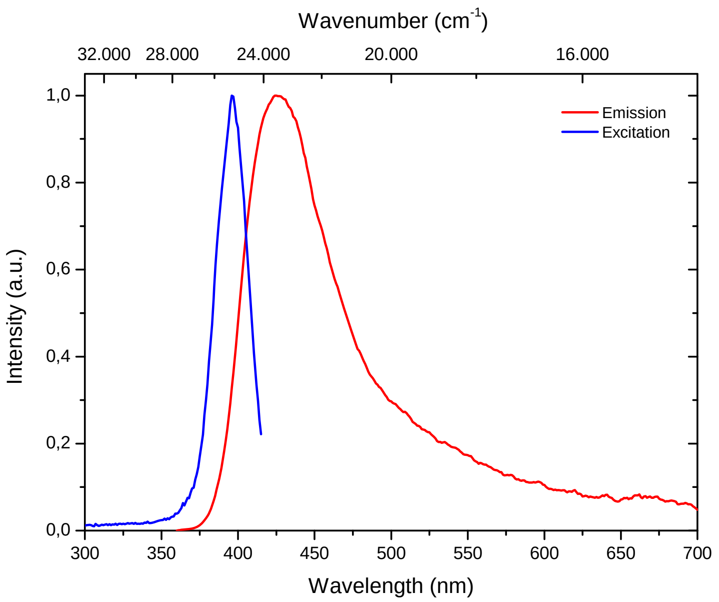

Figure S17: Normalized excitation (blue line) and emission (red line) spectra of **16** in the solid-state. Emission was monitored by excitation at the absorption wavelength of 396 nm and excitation was monitored at fluorescence maximum wavelength of 424 nm.

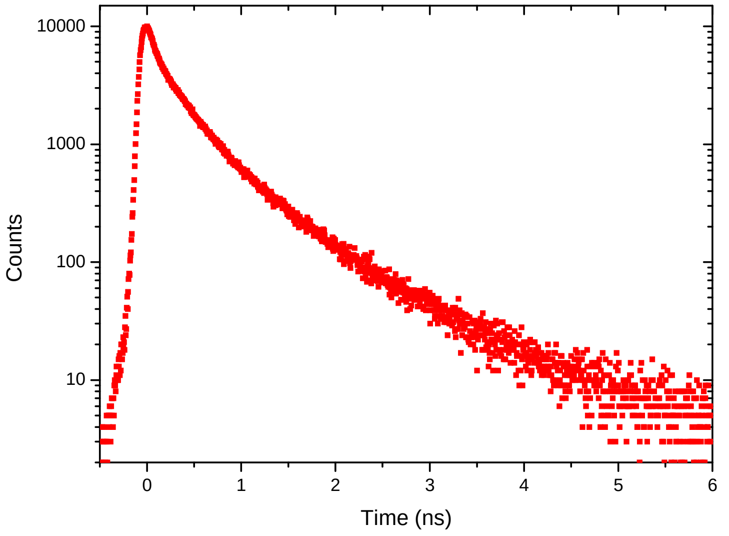

Figure S18: Fluorescence decay profile of **16** in the solid-state with excitation at 376 nm: τ_1 = 0.286 ± 0.002 (54.4%) ns; τ_2 = 0.872 ± 0.006 (45.6%) ns; χ^2 = 1.283.

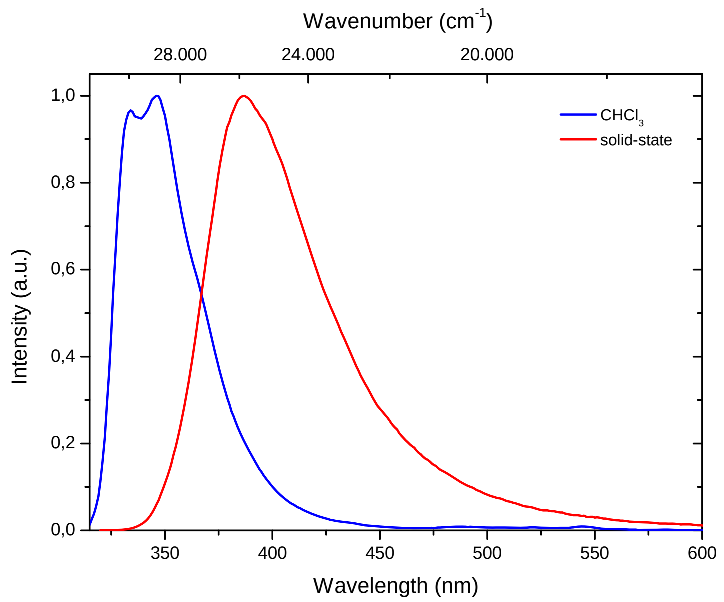

Figure S19: Normalized emission spectra of **19** in chloroform solution (blue line, $c = 10^{-5}$ mol L^{-1}) after bubbling with argon for 30 minutes and the solid-state (red line). Quantum yield in the solid-state was determined to 6.0 %.

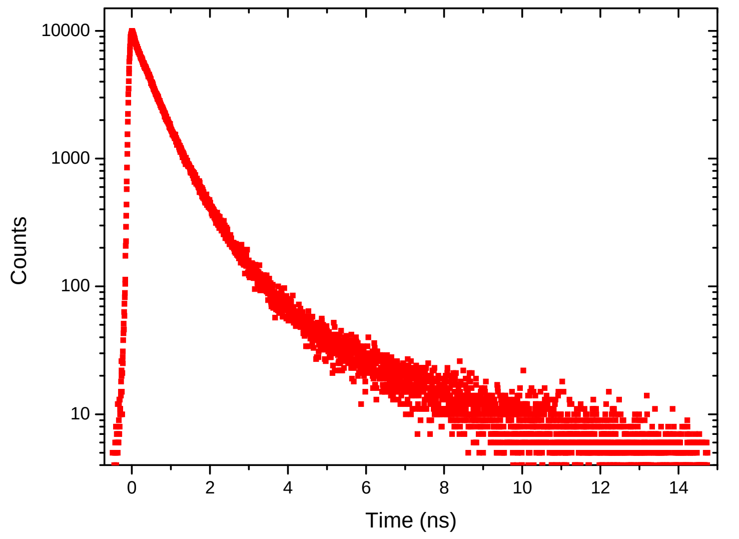

Figure S20: Fluorescence decay profile of **19** in the solid-state with excitation at 376 nm: $\tau_1 = 0.554 \pm 0.001$ (85.3%) ns; $\tau_2 = 2.043 \pm 0.020$ (14.7%) ns; $\chi^2 = 1.430$.

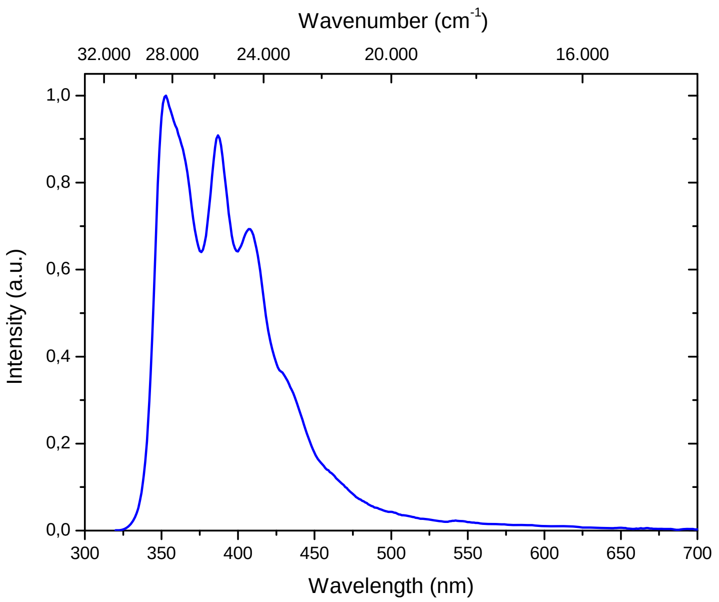

Figure S21: Normalized emission spectrum of **20** in the solid-state. Quantum yield in the solid-state was determined to 12.6 %.

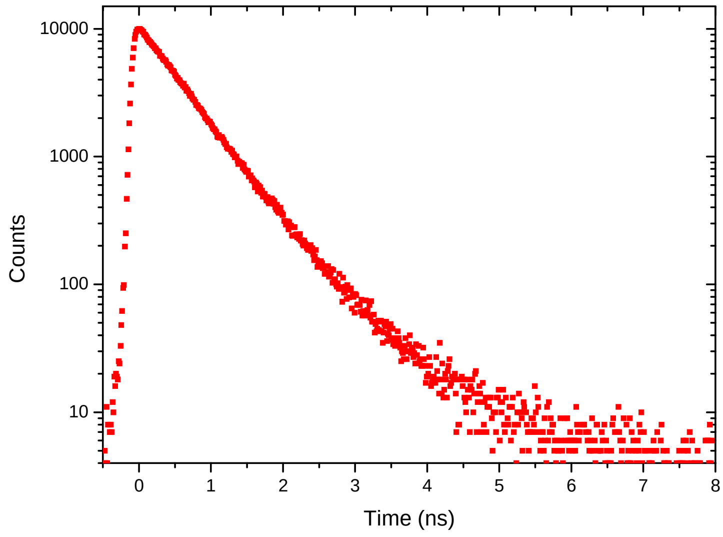

Figure S22: Fluorescence decay profile of **20** in the solid-state with excitation at 376 nm: τ = 0.587 ± 0.001 ns; χ^2 = 1.430. Emission was detected at 407 nm.

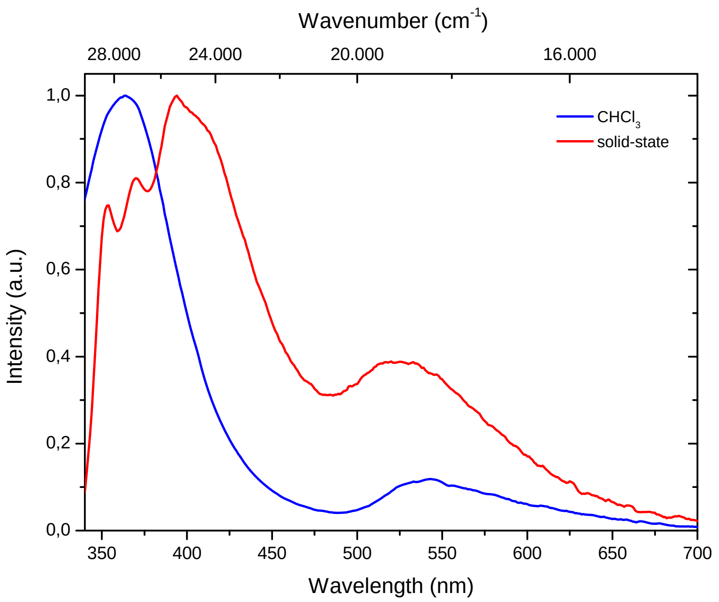

Figure S23: Normalized emission spectra of **21** in chloroform solution (blue line, $c = 10^{-5}$ mol L^{-1}) after bubbling with argon for 30 minutes and the solid-state (red line).

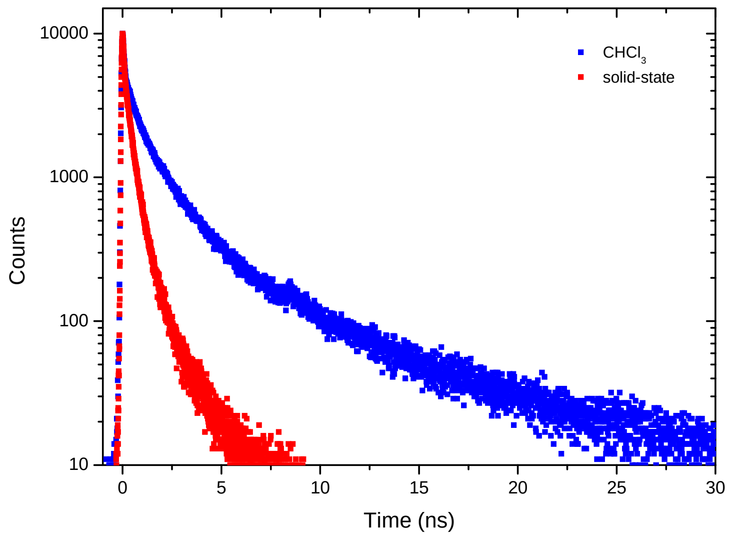

Figure S24: Fluorescence decay profile of **21** in chloroform solution (blue dots, detection at 400 nm) after bubbling with argon for 30 minutes and the solid-state (red dots, detection at 392 nm) with excitation at 376 nm. $CHCl_3$: $\tau_1 = 0.442 \pm 0.011$ (12.9%) ns; $\tau_2 = 1.697 \pm 0.020$ (54.6%) ns; $\tau_3 = 7.630 \pm 0.080$ (32.5%) ns; $\chi^2 = 1.199$. Solid-state: $\tau_1 = 0.378 \pm 0.002$ (73.5%) ns; $\tau_2 = 1.648 \pm 0.013$ (26.5%) ns; $\chi^2 = 1.274$.

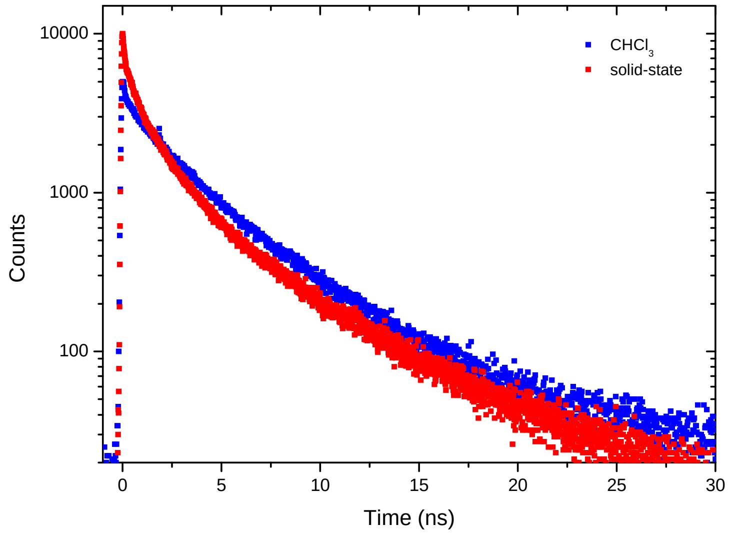

Figure S25: Fluorescence decay profile of **21** in chloroform solution (blue dots, detection at 550 nm) after bubbling with argon for 30 minutes and the solid-state (red dots, detection at 527 nm) with excitation at 376 nm.
$CHCl_3$: τ_1 = 2.137 ± 0.030 (40.5%) ns; τ_2 = 5.747 ± 0.058 (59.5%) ns; χ^2 = 1.404.
Solid-state: τ_1 = 1.503 ± 0.008 (45.2%) ns; τ_2 = 6.153 ± 0.027 (54.8%) ns; χ^2 = 1.445.

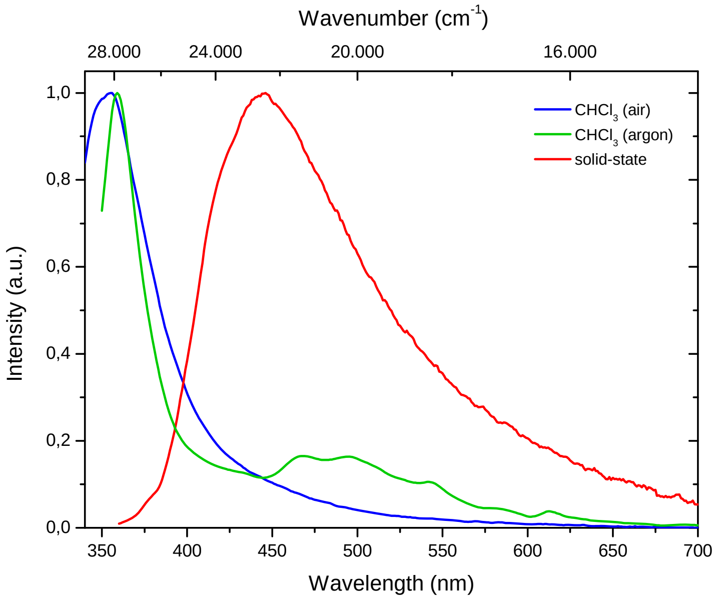

Figure S26: Normalized emission spectra of **5** in chloroform solution (blue line, $c = 10^{-5}$ mol L^{-1}) under air, in chloroform solution (green line, $c = 10^{-5}$ mol L^{-1}) after bubbling with argon for 30 minutes and the solid-state (red line).

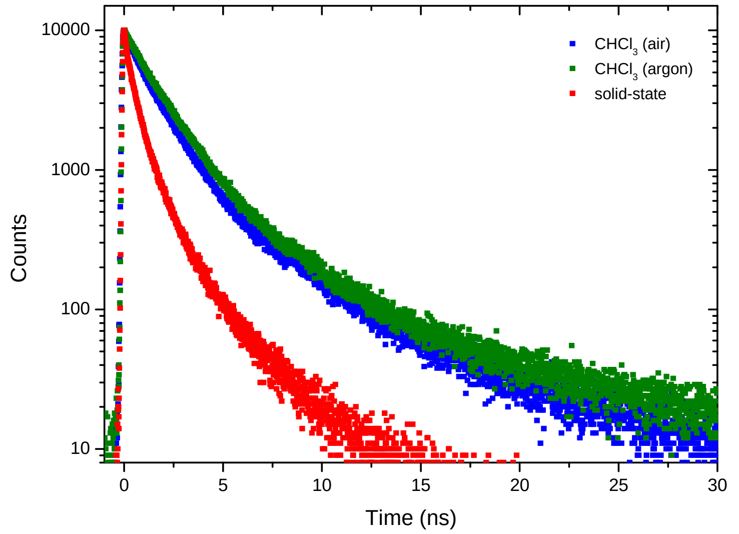

Figure S27: Fluorescence decay profile of **5** in chloroform solution under air (blue dots, detection at 450 nm), in chloroform solution after bubbling with argon for 30 minutes (green dots, detection at 450 nm) and the solid-state (red dots, detection at 446 nm) with excitation at 376 nm.

$CHCl_3$ (air): τ_1 = 1.459 ± 0.004 (72.2%) ns; τ_2 = 5.692 ± 0.030 (27.8%) ns; χ^2 = 1.353.

$CHCl_3$ (argon): τ_1 = 1.709 ± 0.004 (77.1%) ns; τ_2 = 6.288 ± 0.045 (22.9%) ns; χ^2 = 1.265.

Solid-state: τ_1 = 0.637 ± 0.003 (66.2%) ns; τ_2 = 2.601 ± 0.015 (33.8%) ns; χ^2 = 1.183.

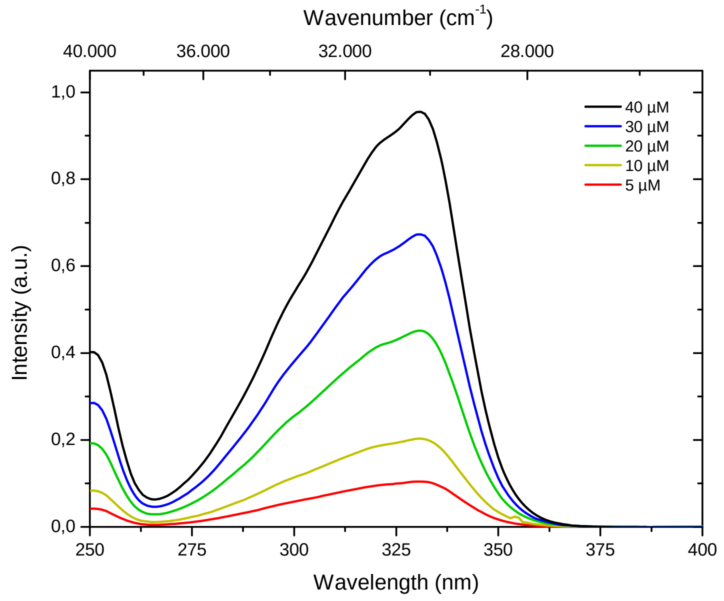

Figure S28: Absorption spectra of **5** at various concentration in chloroform solution.

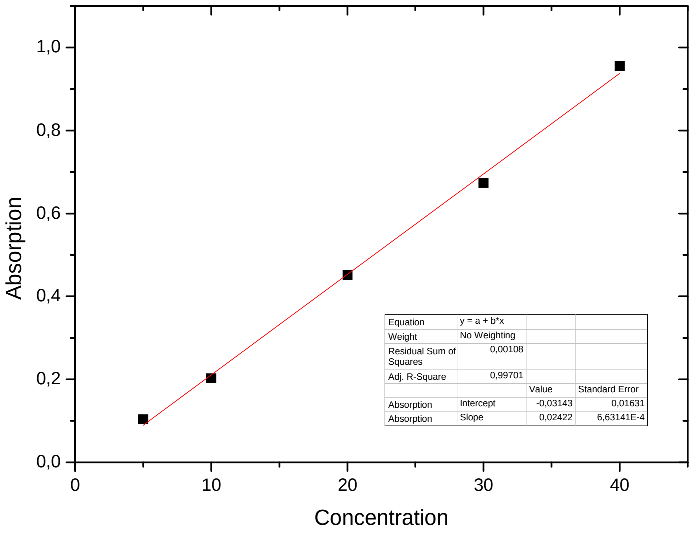

Figure S29: Linear regression for extinction coefficient determination (with respect to Figure S28): ε_{331} = 24.220 L mol^{-1} cm^{-1}.

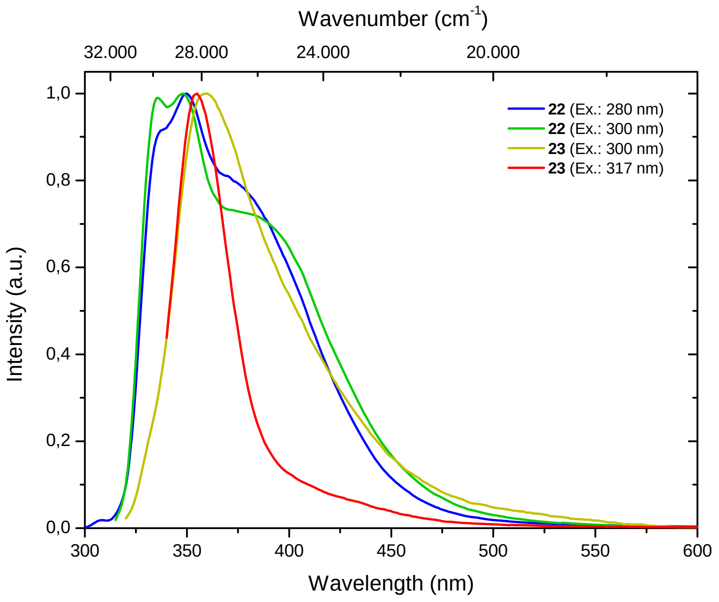

Figure S30: Normalized emission spectra of **22** in chloroform solution (blue line, c = 10^{-5} mol L^{-1}, excitation at 280 nm) and (green line, excitation at 300 nm) and of **23** in chloroform solution (yellow line, c = 10^{-5} mol L^{-1}, excitation at 300 nm) and (red line, excitation at 317 nm) after bubbling with argon for 30 minutes.

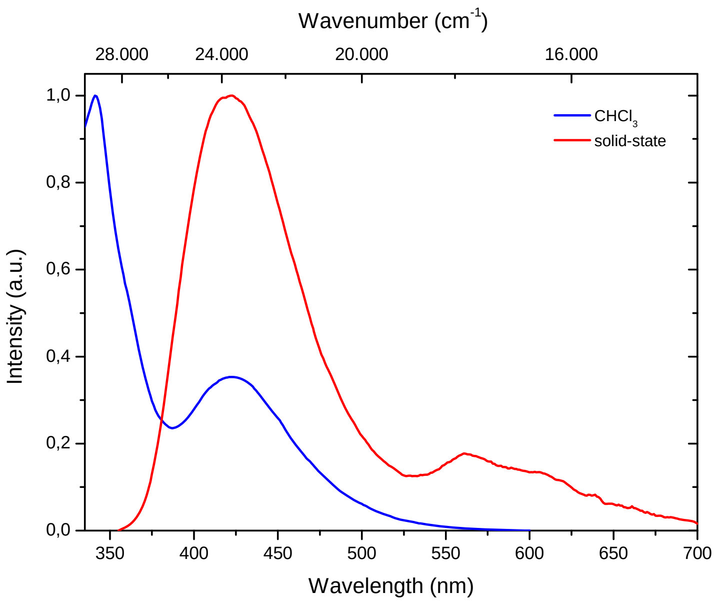

Figure S31: Normalized emission spectra of **25** in chloroform solution (blue line, $c = 10^{-5}$ mol L^{-1}) after bubbling with argon for 30 minutes and the solid-state (red line).

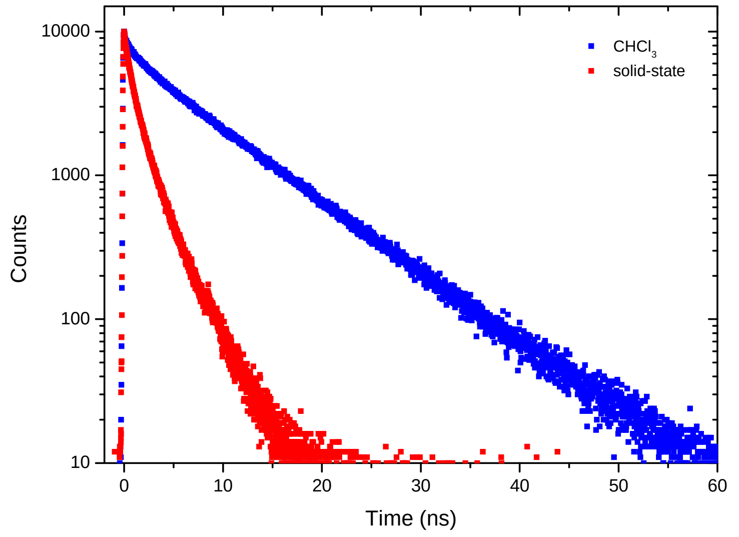

Figure S32: Fluorescence decay profile of **25** in chloroform solution (blue dots, detection at 430 nm) after bubbling with argon for 30 minutes and the solid-state (red dots, detection at 560 nm) with excitation at 376 nm.

$CHCl_3$: $\tau_1 = 1.505 \pm 0.027$ (5.5%) ns; $\tau_2 = 8.661 \pm 0.009$ (94.5%) ns; $\chi^2 = 1.135$.

Solid-state: $\tau_1 = 0.876 \pm 0.005$ (44.9%) ns; $\tau_2 = 2.826 \pm 0.010$ (55.1%) ns; $\chi^2 = 1.404$.

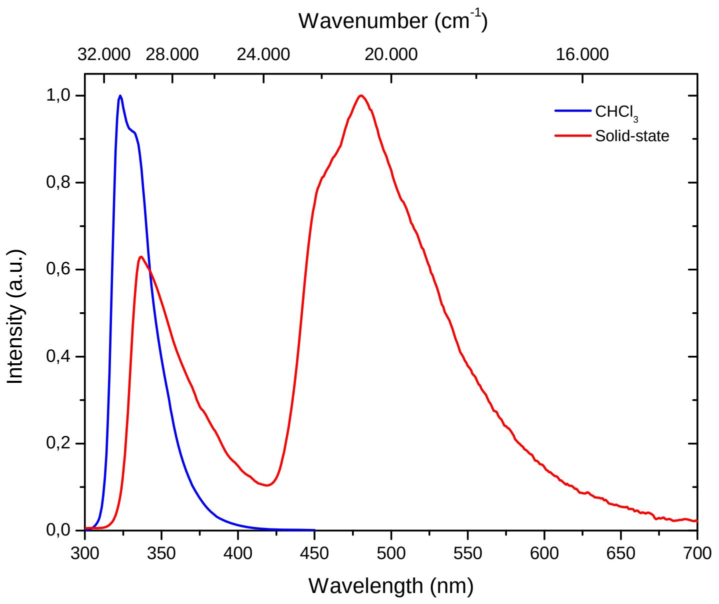

Figure S33: Normalized emission spectra of **28** in chloroform solution (blue line, $c = 10^{-5}$ mol L^{-1}) after bubbling with argon for 30 minutes and the solid-state (red line).

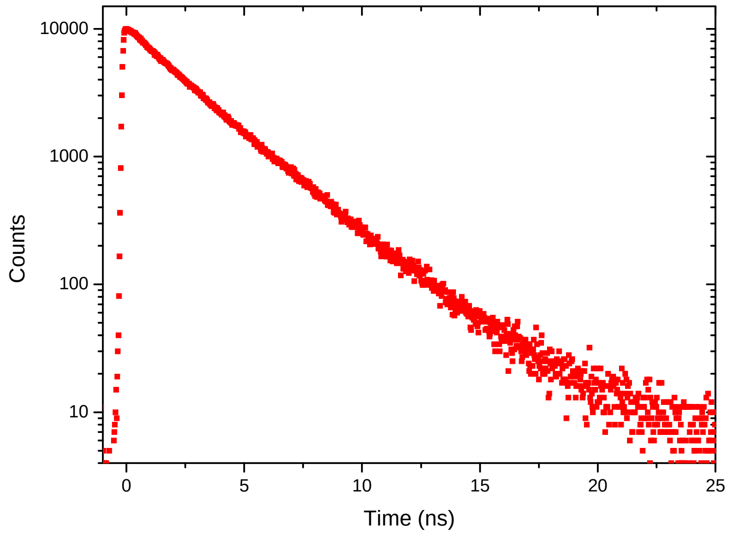

Figure S34: Fluorescence decay profile of **28** in the solid-state with excitation at 376 nm: $\tau_1 = 2.564 \pm 0.009$ ns (93.0%); $\tau_2 = 5.513 \pm 0.158$ (7.1%) ns; $\chi^2 = 1.099$. Emission was detected at 480 nm.

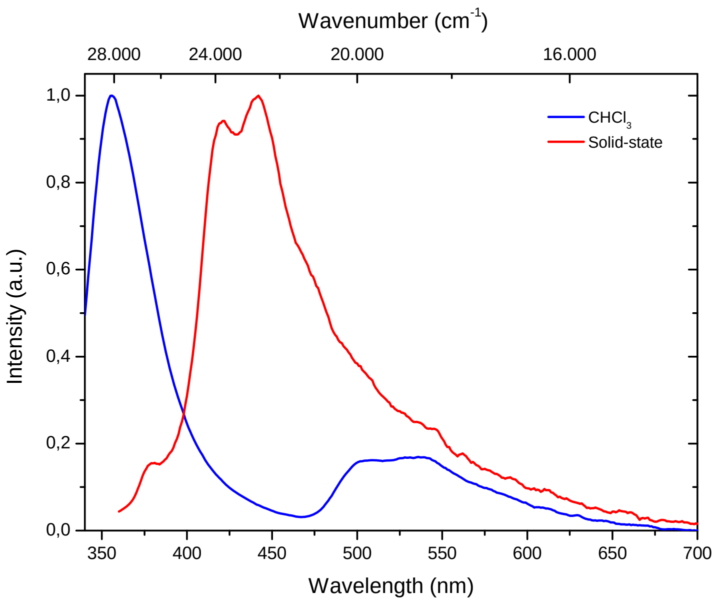

Figure S35: Normalized emission spectra of **42** in chloroform solution (blue line, $c = 10^{-5}$ mol L^{-1}) after bubbling with argon for 30 minutes and the solid-state (red line).

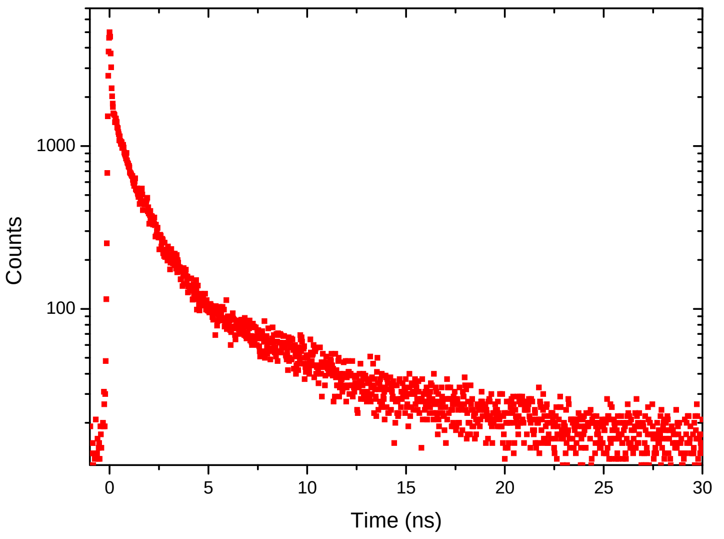

Figure S36: Fluorescence decay profile of **42** in chloroform solution ($c = 10^{-5}$ mol L^{-1}) after bubbling with argon for 30 minutes with excitation at 376 nm: $\tau_1 = 1.004 \pm 0.010$ (57.7%) ns; $\tau_2 = 6.171 \pm 0.104$ (42.3%) ns; $\chi^2 = 1.285$. Emission was detected at 540 nm.

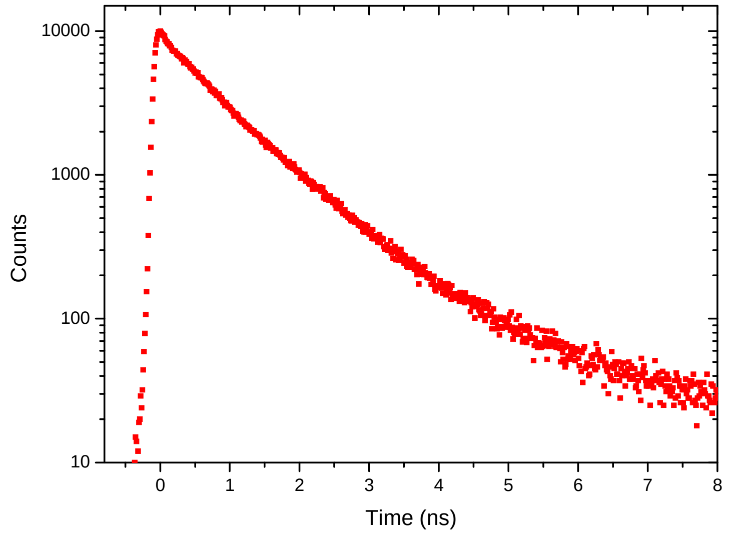

Figure S37: Fluorescence decay profile of **42** in the solid-state with excitation at 376 nm: τ_1 = 0.871 ± 0.002 ns (90.7%); τ_2 = 4.805 ± 0.049 (9.3%) ns; χ^2 = 1.457. Emission was detected at 442 nm.

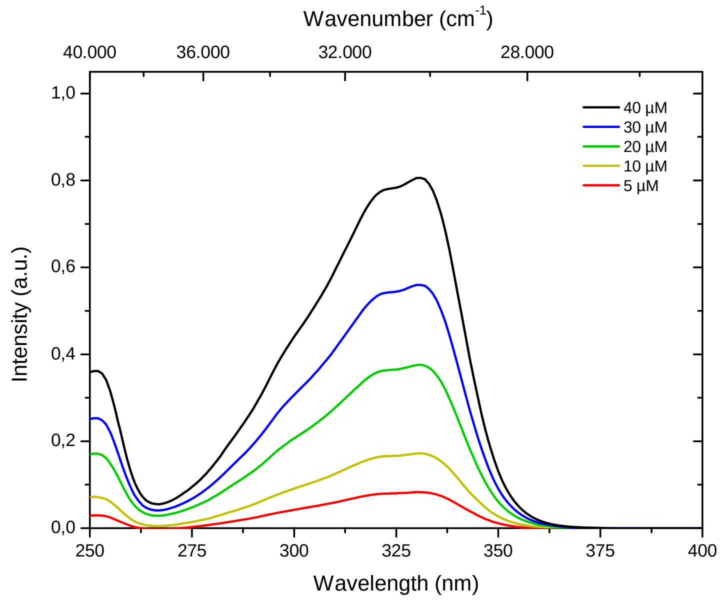

Figure S38: Absorption spectra of **42** at various concentration in chloroform solution.

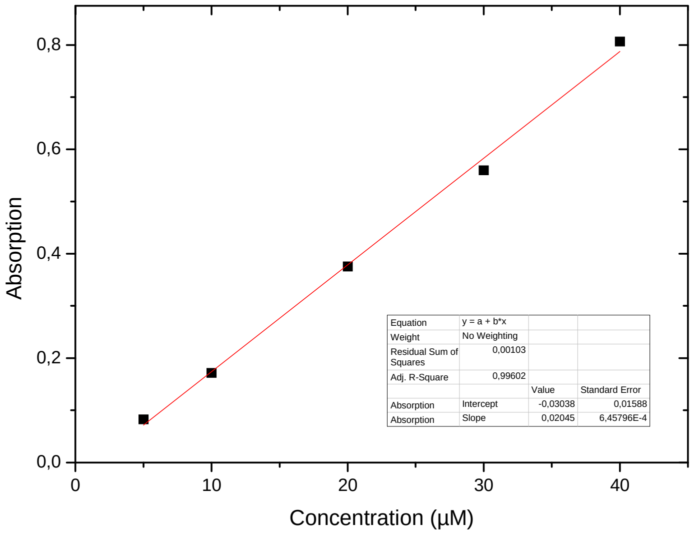

Figure S39: Linear regression for extinction coefficient determination (with respect to Figure S38): ε_{331} = 20.450 L mol^{-1} cm^{-1}.

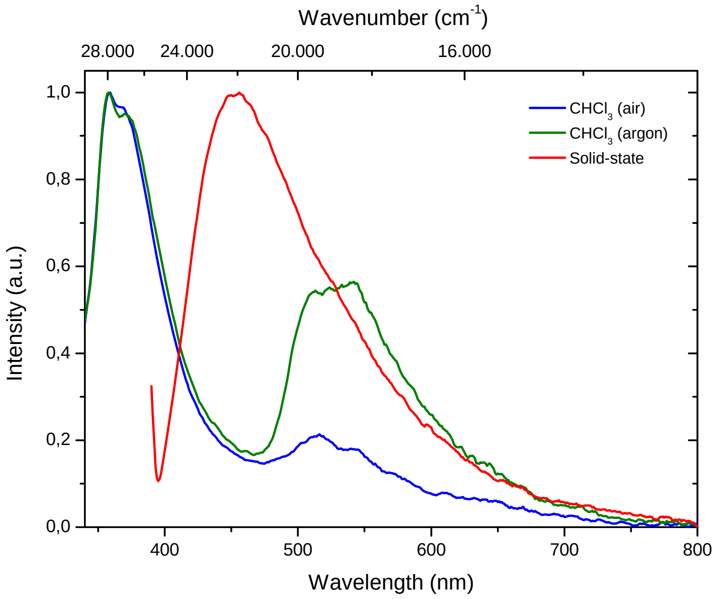

Figure S40: Normalized emission spectra of **45** in chloroform solution (blue line, c = 10^{-5} mol L^{-1}) under air, in chloroform solution (green line, c = 10^{-5} mol L^{-1}) after bubbling with argon for 30 minutes and the solid-state (red line).

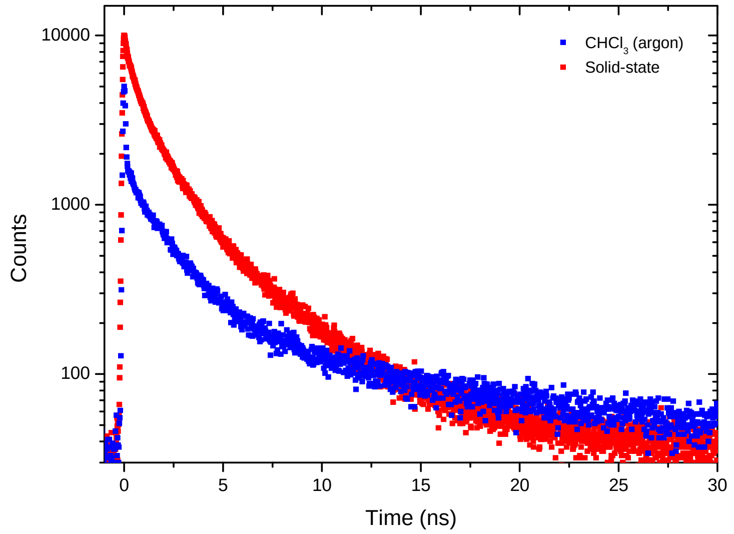

Figure S41: Fluorescence decay profile of **45** in chloroform solution (blue dots, detection at 540 nm) after bubbling with argon for 30 minutes and the solid-state (red dots, detection at 580 nm) with excitation at 376 nm.

$CHCl_3$: τ_1 = 1.824 ± 0.018 (51.3%) ns; τ_2 = 11.56 ± 0.31 (48.7%) ns; χ^2 = 1.225.

Solid-state: τ_1 = 1.076 ± 0.006 (43.9%) ns; τ_2 = 4.050 ± 0.018 (56.1%) ns; χ^2 = 1.435.

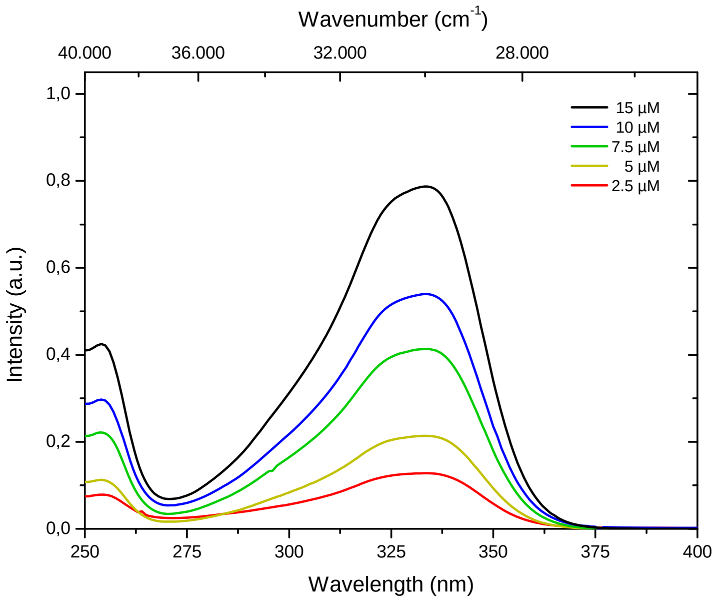

Figure S42: Absorption spectra of **45** at various concentration in chloroform solution.

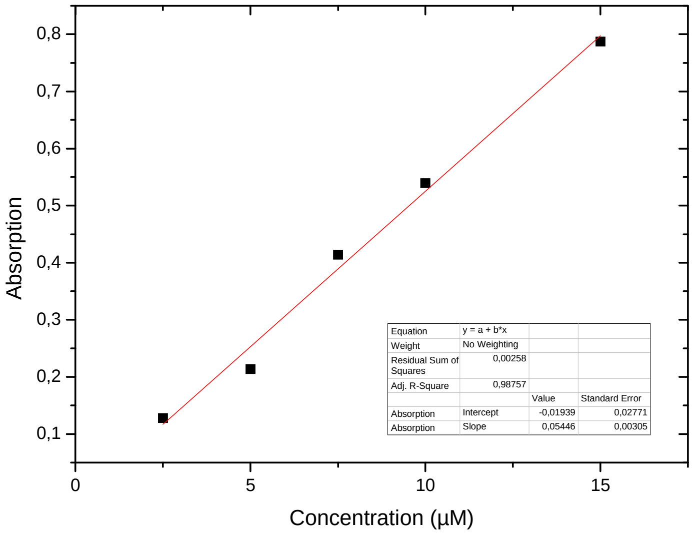

Figure S43: Linear regression for extinction coefficient determination (with respect to Figure S42): ε_{334} = 54.460 L mol^{-1} cm^{-1}.

4 Crystal Structure of 3-(Azidomethyl)-7-bromo-9,9-dimethyl-9*H*-fluorene-2-carbaldehyde (5)

4.1 Crystal Data for (5)

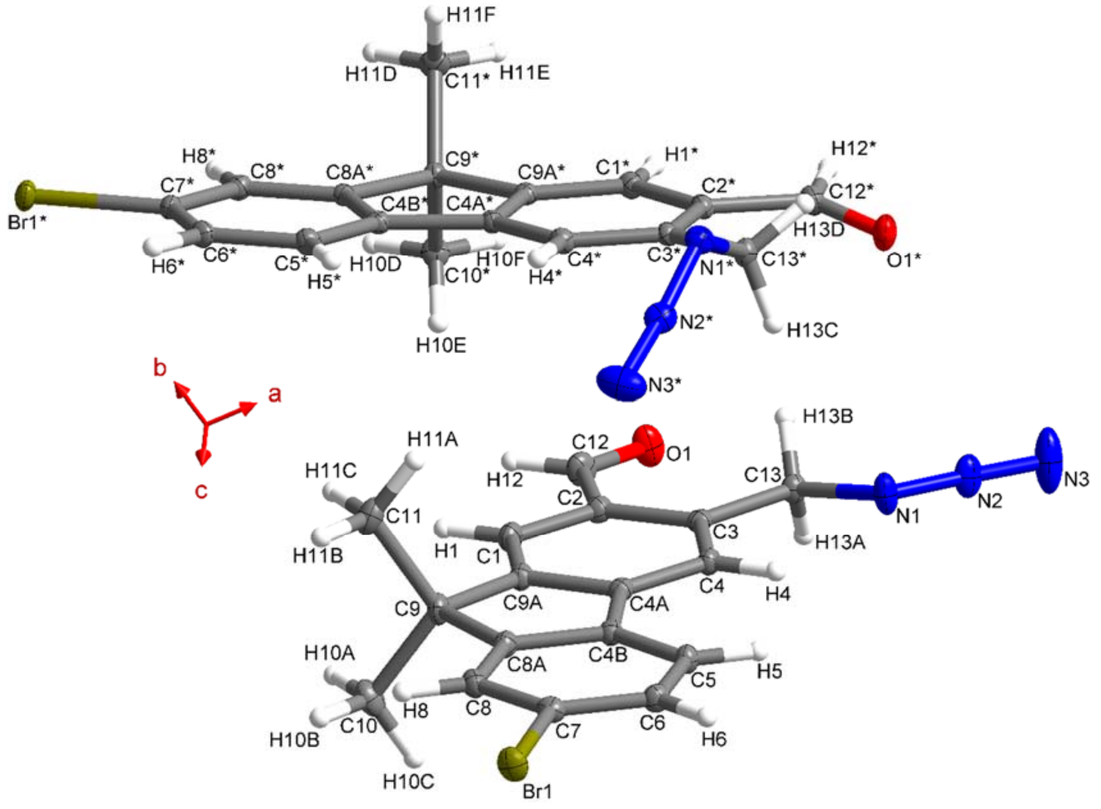

Figure S1: Asymmetric unit of **5** with atom labels.

Table S1: Crystal data and structure refinement for **5**.

Empirical formula	$C_{17}H_{14}BrN_3O$
Formula weight, g mol^{-1}	356.22
Crystal system	Triclinic
Crystal size, mm^3	0.3 × 0.2 × 0.2
Space group (number)	P–1 (2)
a/Å	8.2141(3)
b/Å	12.7921(4)
c/Å	16.1129(6)
α/°	107.8710(10)
β/°	102.8730(10)
γ/°	99.2410(10)
Volume/Å^3	1522.30(9)

Z	4
$\rho_{calc,}$ g/cm^3	1.554
μ/mm^{-1}	2.705
F(000)	720
2Θ range for data collection/°	5.18 to 61.22
Index ranges	$-11 \leq h \leq 11$<br>$-18 \leq k \leq 18$<br>$-23 \leq l \leq 23$
No. of reflections collected	53013
No. of independent reflections	9309 [R_{int} = 0.0274, R_{sigma} = 0.0219]
Data/restraints/parameters	9309/0/401
Goodness-of-fit on F^2	1.081
Final R indexes [I ≥ 2σ (I)]	R_1 = 0.0246, wR_2 = 0.0540
Final R indexes [all data]	R_1 = 0.0310, wR_2 = 0.0561
Largest diff. peak/hole/ e Å^{-3}	0.60/–0.46
Completeness to θ = 25.242°	99.9 %
CCDC number	1989134

Table S2: Fractional Atomic Coordinates [A^2] and Equivalent Isotropic Displacement Parameters [Å^2] for **5**. U_{eq} is defined as 1/3 of the trace of the orthogonalised U_{IJ} tensor.

Atom	*x*	*y*	*z*	U_{eq}
Br1	0.62959(2)	0.87935(2)	0.88875(2)	0.01738(4)
O1	0.33038(13)	0.46964(9)	0.13714(7)	0.0220(2)
N1	0.79293(15)	0.48548(11)	0.32343(8)	0.0190(2)
N2	0.87272(16)	0.42834(11)	0.27937(8)	0.0204(2)
N3	0.95945(19)	0.37986(15)	0.24644(10)	0.0381(4)
C1	0.28731(16)	0.63068(11)	0.35404(9)	0.0144(2)
H1	0.181517	0.646152	0.329310	0.017
C2	0.36981(16)	0.56857(11)	0.29586(9)	0.0132(2)
C3	0.52889(16)	0.54570(10)	0.33167(8)	0.0113(2)
C4	0.59980(16)	0.58406(10)	0.42580(8)	0.0117(2)
H4	0.705325	0.568757	0.451174	0.014
C4A	0.51590(16)	0.64483(10)	0.48263(8)	0.0115(2)
C4B	0.56563(16)	0.69502(10)	0.58212(8)	0.0115(2)
C5	0.70807(16)	0.69389(11)	0.64733(9)	0.0134(2)
H5	0.791526	0.655539	0.629495	0.016
C6	0.72612(17)	0.74983(11)	0.73898(9)	0.0136(2)

H6	0.822249	0.750274	0.784673	0.016
C7	0.60132(16)	0.80525(11)	0.76293(9)	0.0127(2)
C8	0.45795(16)	0.80749(11)	0.69884(9)	0.0138(2)
H8	0.374611	0.845824	0.716874	0.017
C8A	0.44162(16)	0.75146(11)	0.60754(9)	0.0123(2)
C9	0.29979(16)	0.74108(11)	0.52397(9)	0.0137(2)
C9A	0.36006(16)	0.66938(11)	0.44743(9)	0.0124(2)
C10	0.12230(17)	0.68180(13)	0.52496(10)	0.0200(3)
H10A	0.036244	0.669237	0.467621	0.030
H10B	0.090161	0.729581	0.576028	0.030
H10C	0.127354	0.608788	0.531852	0.030
C11	0.29608(19)	0.85823(12)	0.51709(10)	0.0197(3)
H11A	0.410171	0.895712	0.517395	0.030
H11B	0.264871	0.904650	0.569130	0.030
H11C	0.210823	0.848851	0.460379	0.030
C12	0.28224(17)	0.52849(12)	0.19717(9)	0.0178(3)
H12	0.179054	0.550673	0.178968	0.021
C13	0.61929(16)	0.48143(11)	0.26895(8)	0.0129(2)
H13A	0.551907	0.401722	0.237200	0.016
H13B	0.629690	0.515846	0.222492	0.016
Br1*	-0.22890(2)	1.00015(2)	0.14743(2)	0.01550(4)
O1*	0.89725(13)	0.61783(9)	0.17610(7)	0.0191(2)
N1*	0.88357(14)	0.89128(9)	0.40051(8)	0.0143(2)
N2*	0.81665(15)	0.88222(10)	0.46022(8)	0.0158(2)
N3*	0.76277(19)	0.88614(12)	0.51975(9)	0.0285(3)
C1*	0.51351(16)	0.69391(11)	0.08530(9)	0.0121(2)
H1*	0.499974	0.649222	0.023855	0.015
C2*	0.65942(16)	0.70333(11)	0.15443(9)	0.0117(2)
C3*	0.68417(16)	0.77256(10)	0.24610(8)	0.0111(2)
C4*	0.55942(16)	0.82955(11)	0.26659(8)	0.0112(2)
H4*	0.573964	0.876650	0.327514	0.013
C4A*	0.41258(15)	0.81752(10)	0.19750(8)	0.0100(2)
C4B*	0.26634(16)	0.86909(10)	0.20113(8)	0.0106(2)
C5*	0.23047(16)	0.94449(11)	0.27376(8)	0.0121(2)
H5*	0.306336	0.969258	0.333719	0.015
C6*	0.08156(17)	0.98299(11)	0.25707(9)	0.0129(2)
H6*	0.054806	1.034677	0.305587	0.016

C7*	-0.02773(16)	0.94503(11)	0.16862(9)	0.0120(2)
C8*	0.00514(16)	0.86908(11)	0.09497(9)	0.0121(2)
H8*	-0.071691	0.843831	0.035221	0.014
C8A*	0.15437(15)	0.83179(10)	0.11232(8)	0.0102(2)
C9*	0.22183(16)	0.75194(10)	0.04453(8)	0.0105(2)
C9A*	0.38937(15)	0.74993(10)	0.10694(8)	0.0102(2)
C10*	0.09774(16)	0.63361(11)	-0.00032(9)	0.0142(2)
H10D	-0.013154	0.638890	-0.034819	0.021
H10E	0.080477	0.603261	0.046861	0.021
H10F	0.146688	0.583159	-0.041645	0.021
C11*	0.25559(18)	0.79907(12)	-0.02848(9)	0.0156(2)
H11D	0.146586	0.804420	-0.064864	0.023
H11E	0.306879	0.748393	-0.068189	0.023
H11F	0.334881	0.874536	0.000948	0.023
C12*	0.78038(17)	0.63657(11)	0.12570(9)	0.0148(2)
H12*	0.766049	0.605275	0.062135	0.018
C13*	0.84582(16)	0.78619(11)	0.31997(8)	0.0128(2)
H13C	0.832009	0.719942	0.339569	0.015
H13D	0.944912	0.787400	0.294493	0.015

Table S3: Bond Lengths for **5**.

Atom–Atom	Length [Å]	Atom–Atom	Length [Å]
Br1–C7	1.8980(13)	Br1*–C7*	1.9013(12)
O1–C12	1.2157(17)	O1*–C12*	1.2185(16)
N1–N2	1.2291(16)	N1*–N2*	1.2349(16)
N1–C13	1.4826(16)	N1*–C13*	1.4844(16)
N2–N3	1.1321(18)	N2*–N3*	1.1337(18)
C1–H1	0.9500	C1*–H1*	0.9500
C1–C2	1.4028(18)	C1*–C2*	1.4029(17)
C1–C9A	1.3833(18)	C1*–C9A*	1.3837(17)
C2–C3	1.4200(17)	C2*–C3*	1.4188(17)
C2–C12	1.4766(18)	C2*–C12*	1.4716(17)
C3–C4	1.3922(17)	C3*–C4*	1.3905(17)
C3–C13	1.5064(18)	C3*–C13*	1.5180(17)
C4–H4	0.9500	C4*–H4*	0.9500
C4–C4A	1.3907(17)	C4*–C4A*	1.3981(17)
C4A–C4B	1.4632(17)	C4A*–C4B*	1.4641(17)

C4A–C9A	1.4050(17)	C4A*–C9A*	1.4007(17)
C4B–C5	1.3928(17)	C4B*–C5*	1.3925(17)
C4B–C8A	1.4027(17)	C4B*–C8A*	1.4053(17)
C5–H5	0.9500	C5*–H5*	0.9500
C5–C6	1.3910(18)	C5*–C6*	1.3926(17)
C6–H6	0.9500	C6*–H6*	0.9500
C6–C7	1.3943(18)	C6*–C7*	1.3922(18)
C7–C8	1.3933(18)	C7*–C8*	1.3944(18)
C8–H8	0.9500	C8*–H8*	0.9500
C8–C8A	1.3892(18)	C8*–C8A*	1.3875(17)
C8A–C9	1.5263(17)	C8A*–C9*	1.5199(17)
C9–C9A	1.5267(18)	C9*–C9A*	1.5219(17)
C9–C10	1.5374(19)	C9*–C10*	1.5353(17)
C9–C11	1.5409(19)	C9*–C11*	1.5335(17)
C10–H10A	0.9800	C10*–H10D	0.9800
C10–H10B	0.9800	C10*–H10E	0.9800
C10–H10C	0.9800	C10*–H10F	0.9800
C11–H11A	0.9800	C11*–H11D	0.9800
C11–H11B	0.9800	C11*–H11E	0.9800
C11–H11C	0.9800	C11*–H11F	0.9800
C12–H12	0.9500	C12*–H12*	0.9500
C13–H13A	0.9900	C13*–H13C	0.9900
C13–H13B	0.9900	C13*–H13D	0.9900

Table S4: Bond Angles for **5**.

Atom–Atom–Atom	Angle [°]	Atom–Atom–Atom	Angle [°]
N2–N1–C13	114.01(11)	N2*–N1*–C13*	115.47(11)
N3–N2–N1	173.21(14)	N3*–N2*–N1*	172.57(14)
C2–C1–H1	120.0	C2*–C1*–H1*	120.1
C9A–C1–H1	120.0	C9A*–C1*–H1*	120.1
C9A–C1–C2	119.98(12)	C9A*–C1*–C2*	119.76(11)
C1–C2–C3	120.73(11)	C1*–C2*–C3*	120.79(11)
C1–C2–C12	116.65(11)	C1*–C2*–C12*	115.95(11)
C3–C2–C12	122.62(12)	C3*–C2*–C12*	123.25(11)
C2–C3–C13	120.59(11)	C2*–C3*–C13*	120.55(11)
C4–C3–C2	118.72(11)	C4*–C3*–C2*	118.76(11)
C4–C3–C13	120.69(11)	C4*–C3*–C13*	120.68(11)

C3–C4–H4	120.1	C3*–C4*–H4*	120.0
C4A–C4–C3	119.89(11)	C3*–C4*–C4A*	119.97(11)
C4A–C4–H4	120.1	C4A*–C4*–H4*	120.0
C4–C4A–C4B	129.90(11)	C4*–C4A*–C4B*	130.38(11)
C4–C4A–C9A	121.56(11)	C4*–C4A*–C9A*	121.10(11)
C9A–C4A–C4B	108.52(11)	C9A*–C4A*–C4B*	108.49(10)
C5–C4B–C4A	130.36(12)	C5*–C4B*–C4A*	131.05(11)
C5–C4B–C8A	121.10(12)	C5*–C4B*–C8A*	120.85(11)
C8A–C4B–C4A	108.53(11)	C8A*–C4B*–C4A*	108.09(10)
C4B–C5–H5	120.5	C4B*–C5*–H5*	120.5
C6–C5–C4B	118.92(12)	C4B*–C5*–C6*	118.91(11)
C6–C5–H5	120.5	C6*–C5*–H5*	120.5
C5–C6–H6	120.4	C5*–C6*–H6*	120.3
C5–C6–C7	119.15(12)	C7*–C6*–C5*	119.37(11)
C7–C6–H6	120.4	C7*–C6*–H6*	120.3
C6–C7–Br1	117.83(9)	C6*–C7*–Br1*	118.55(9)
C8–C7–Br1	119.29(10)	C6*–C7*–C8*	122.69(11)
C8–C7–C6	122.88(12)	C8*–C7*–Br1*	118.77(9)
C7–C8–H8	121.3	C7*–C8*–H8*	121.3
C8A–C8–C7	117.40(12)	C8A*–C8*–C7*	117.44(11)
C8A–C8–H8	121.3	C8A*–C8*–H8*	121.3
C4B–C8A–C9	111.03(11)	C4B*–C8A*–C9*	111.33(10)
C8–C8A–C4B	120.56(12)	C8*–C8A*–C4B*	120.74(11)
C8–C8A–C9	128.41(12)	C8*–C8A*–C9*	127.93(11)
C8A–C9–C9A	100.99(10)	C8A*–C9*–C9A*	100.86(9)
C8A–C9–C10	111.47(11)	C8A*–C9*–C10*	111.25(10)
C8A–C9–C11	110.98(11)	C8A*–C9*–C11*	111.67(10)
C9A–C9–C10	112.16(11)	C9A*–C9*–C10*	111.31(10)
C9A–C9–C11	110.84(11)	C9A*–C9*–C11*	111.24(10)
C10–C9–C11	110.14(11)	C11*–C9*–C10*	110.23(10)
C1–C9A–C4A	119.11(12)	C1*–C9A*–C4A*	119.58(11)
C1–C9A–C9	129.96(11)	C1*–C9A*–C9*	129.19(11)
C4A–C9A–C9	110.91(11)	C4A*–C9A*–C9*	111.22(10)
C9–C10–H10A	109.5	C9*–C10*–H10D	109.5
C9–C10–H10B	109.5	C9*–C10*–H10E	109.5
C9–C10–H10C	109.5	C9*–C10*–H10F	109.5
H10A–C10–H10B	109.5	H10D–C10*–H10E	109.5

H10A–C10–H10C	109.5	H10D–C10*–H10F	109.5
H10B–C10–H10C	109.5	H10E–C10*–H10F	109.5
C9–C11–H11A	109.5	C9*–C11*–H11D	109.5
C9–C11–H11B	109.5	C9*–C11*–H11E	109.5
C9–C11–H11C	109.5	C9*–C11*–H11F	109.5
H11A–C11–H11B	109.5	H11D–C11*–H11E	109.5
H11A–C11–H11C	109.5	H11D–C11*–H11F	109.5
H11B–C11–H11C	109.5	H11E–C11*–H11F	109.5
O1–C12–C2	125.83(12)	O1*–C12*–C2*	125.87(12)
O1–C12–H12	117.1	O1*–C12*–H12*	117.1
C2–C12–H12	117.1	C2*–C12*–H12*	117.1
N1–C13–C3	108.94(10)	N1*–C13*–C3*	112.68(10)
N1–C13–H13A	109.9	N1*–C13*–H13C	109.1
N1–C13–H13B	109.9	N1*–C13*–H13D	109.1
C3–C13–H13A	109.9	C3*–C13*–H13C	109.1
C3–C13–H13B	109.9	C3*–C13*–H13D	109.1
H13A–C13–H13B	108.3	H13C–C13*–H13D	107.8

Table S5: Torsion Angles for **5**.

Atom–Atom–Atom–Atom	Torsion Angle [°]	Atom–Atom–Atom–Atom	Torsion Angle [°]
Br1–C7–C8–C8A	-179.52(9)	Br1*–C7*–C8*–C8A*	178.96(9)
N2–N1–C13–C3	174.96(12)	N2*–N1*–C13*–C3*	89.13(14)
C1–C2–C3–C4	1.34(19)	C1*–C2*–C3*–C4*	1.11(18)
C1–C2–C3–C13	-178.51(12)	C1*–C2*–C3*–C13*	-177.50(11)
C1–C2–C12–O1	-176.60(14)	C1*–C2*–C12*–O1*	-166.75(13)
C2–C1–C9A–C4A	-0.49(19)	C2*–C1*–C9A*–C4A*	1.37(18)
C2–C1–C9A–C9	177.71(13)	C2*–C1*–C9A*–C9*	-179.65(12)
C2–C3–C4–C4A	-0.87(18)	C2*–C3*–C4*–C4A*	0.36(18)
C2–C3–C13–N1	171.26(11)	C2*–C3*–C13*–N1*	157.56(11)
C3–C2–C12–O1	2.9(2)	C3*–C2*–C12*–O1*	12.4(2)
C3–C4–C4A–C4B	-178.81(12)	C3*–C4*–C4A*–C4B*	-178.88(12)
C3–C4–C4A–C9A	-0.27(19)	C3*–C4*–C4A*–C9A*	-0.96(18)
C4–C3–C13–N1	-8.58(16)	C4*–C3*–C13*–N1*	-21.03(16)
C4–C4A–C4B–C5	-1.5(2)	C4*–C4A*–C4B*–C5*	-0.1(2)
C4–C4A–C4B–C8A	177.87(13)	C4*–C4A*–C4B*–C8A*	178.41(13)
C4–C4A–C9A–C1	0.96(19)	C4*–C4A*–C9A*–C1*	0.09(18)
C4–C4A–C9A–C9	-177.56(11)	C4*–C4A*–C9A*–C9*	-179.06(11)

C4A–C4B–C5–C6	179.44(13)	C4A*–C4B*–C5*–C6*	178.13(12)
C4A–C4B–C8A–C8	-179.69(11)	C4A*–C4B*–C8A*–C8*	-178.84(11)
C4A–C4B–C8A–C9	0.06(14)	C4A*–C4B*–C8A*–C9*	0.27(14)
C4B–C4A–C9A–C1	179.78(12)	C4B*–C4A*–C9A*–C1*	178.42(11)
C4B–C4A–C9A–C9	1.26(14)	C4B*–C4A*–C9A*–C9*	-0.73(14)
C4B–C5–C6–C7	0.05(19)	C4B*–C5*–C6*–C7*	0.23(19)
C4B–C8A–C9–C9A	0.64(14)	C4B*–C8A*–C9*–C9A*	-0.65(13)
C4B–C8A–C9–C10	119.93(12)	C4B*–C8A*–C9*–C10*	117.48(11)
C4B–C8A–C9–C11	-116.91(12)	C4B*–C8A*–C9*–C11*	-118.90(12)
C5–C4B–C8A–C8	-0.21(19)	C5*–C4B*–C8A*–C8*	-0.14(19)
C5–C4B–C8A–C9	179.54(11)	C5*–C4B*–C8A*–C9*	178.97(11)
C5–C6–C7–Br1	179.42(10)	C5*–C6*–C7*–Br1*	-179.32(9)
C5–C6–C7–C8	-0.1(2)	C5*–C6*–C7*–C8*	0.13(19)
C6–C7–C8–C8A	-0.03(19)	C6*–C7*–C8*–C8A*	-0.49(19)
C7–C8–C8A–C4B	0.17(19)	C7*–C8*–C8A*–C4B*	0.49(18)
C7–C8–C8A–C9	-179.53(12)	C7*–C8*–C8A*–C9*	-178.46(12)
C8–C8A–C9–C9A	-179.64(13)	C8*–C8A*–C9*–C9A*	178.38(12)
C8–C8A–C9–C10	-60.35(18)	C8*–C8A*–C9*–C10*	-63.49(16)
C8–C8A–C9–C11	62.81(17)	C8*–C8A*–C9*–C11*	60.14(16)
C8A–C4B–C5–C6	0.09(19)	C8A*–C4B*–C5*–C6*	-0.23(19)
C8A–C9–C9A–C1	-179.47(13)	C8A*–C9*–C9A*–C1*	-178.21(12)
C8A–C9–C9A–C4A	-1.15(14)	C8A*–C9*–C9A*–C4A*	0.84(13)
C9A–C1–C2–C3	-0.65(19)	C9A*–C1*–C2*–C3*	-1.99(19)
C9A–C1–C2–C12	178.89(12)	C9A*–C1*–C2*–C12*	177.21(11)
C9A–C4A–C4B–C5	179.76(13)	C9A*–C4A*–C4B*–C5*	-178.23(13)
C9A–C4A–C4B–C8A	-0.82(14)	C9A*–C4A*–C4B*–C8A*	0.29(14)
C10–C9–C9A–C1	61.74(18)	C10*–C9*–C9A*–C1*	63.70(16)
C10–C9–C9A–C4A	-119.94(12)	C10*–C9*–C9A*–C4A*	-117.25(11)
C11–C9–C9A–C1	-61.81(18)	C11*–C9*–C9A*–C1*	-59.66(17)
C11–C9–C9A–C4A	116.50(12)	C11*–C9*–C9A*–C4A*	119.39(11)
C12–C2–C3–C4	-178.18(12)	C12*–C2*–C3*–C4*	-178.02(12)
C12–C2–C3–C13	1.97(19)	C12*–C2*–C3*–C13*	3.37(19)
C13–C3–C4–C4A	178.97(11)	C13*–C3*–C4*–C4A*	178.97(11)

4.2 Crystal Packing Views of (5)

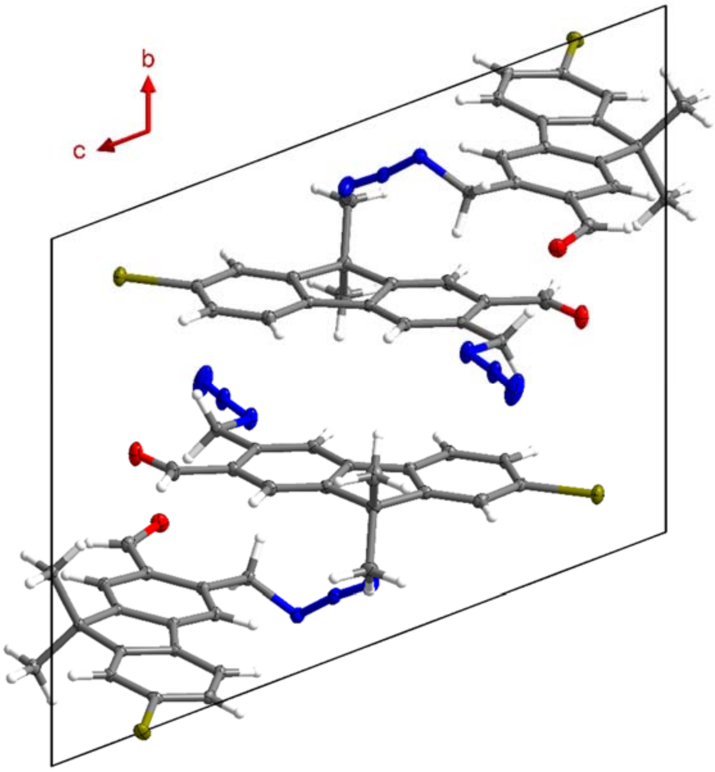

Figure S2: Unit cell packing view of a **5** crystal along the *a*-axis.

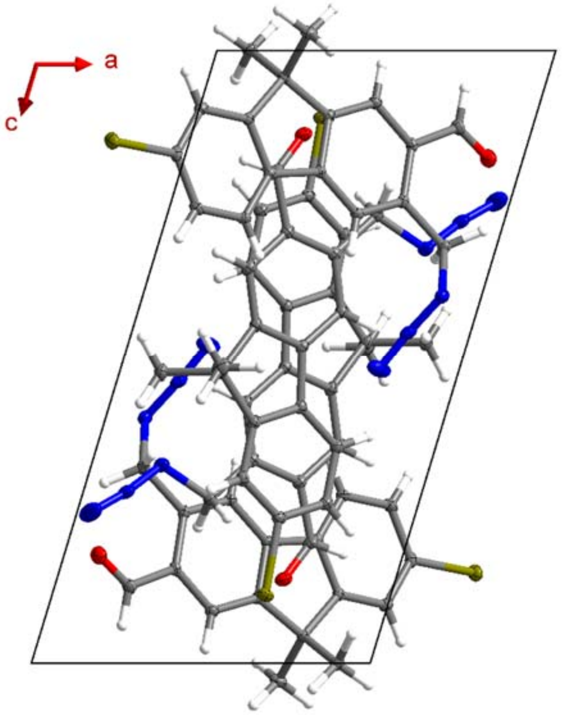

Figure S3: Unit cell packing view of a **5** crystal along the *b*-axis.

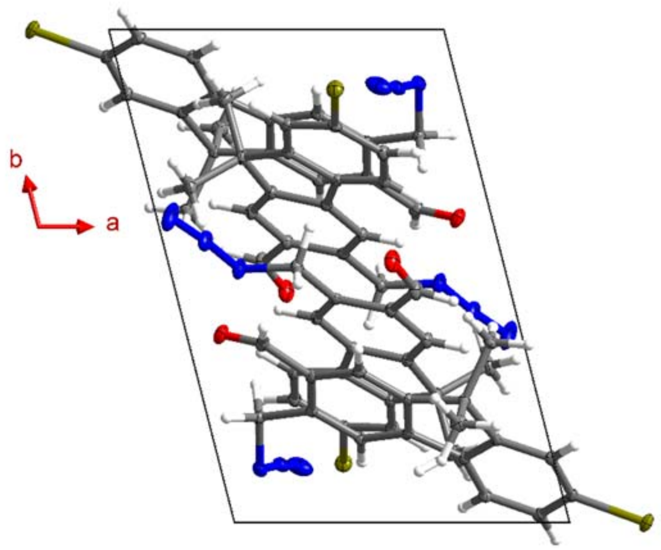

Figure S4: Unit cell packing view of a **5** crystal along the *c*-axis.

5 References

1. Armarego, W. L. F.; Chai, C. L. L. *Purification of Laboratory Chemicals*, 6th ed.; Elsevier/BH: Oxford, 2009.
2. Sheldrick, G. M. *Acta Cryst. A* **2008**, *64*, 112–122. doi:10.1107/S0108767307043930
3. Dolomanov, O. V.; Bourhis, L. J.; Gildea, R. J.; Howard, J. A. K.; Puschmann, H. *J. Appl. Cryst.* **2009**, *42*, 339–341. doi:10.1107/S0021889808042726
4. Krasovskiy, A.; Knochel, P. *Angew. Chem. Int. Ed.* **2004**, *43*, 3333–3336. doi:10.1002/anie.200454084
5. Bodroux, F. *C. R. Chim.* **1902**, *135*, 1350–1351.
6. Krasovskiy, A.; Krasovskaya, V.; Knochel, P. *Angew. Chem. Int. Ed.* **2006**, *45*, 2958–2961. doi:10.1002/anie.200504024
7. Hammett, L. P.; Walden, G. H.; Edmonds, S. M. *J. Am. Chem. Soc.* **1934**, *56*, 1092–1094. doi:10.1021/ja01320a025
8. Schwekendiek, K.; Glorius, F. *Synthesis* **2006**, *2006*, 2996–3002. doi:10.1055/s-2006-950198
9. A. I. Meyers; Davis L. Temple; Dan Haidukewych; and Edward D. Mihelich; Meyers, A. I.; Temple, D. L.; Haidukewych, D.; Mihelich, E. D. *J. Org. Chem.* **1974**, *39*, 2787–2793. doi:10.1021/jo00932a024
10. Batista, J. H. C.; dos Santos, F. M.; Bozzini, L. A.; Vessecchi, R.; Oliveira, A. R. M.; Clososki, G. C. *Eur. J. Org. Chem.* **2015**, *2015*, 967–977. doi:10.1002/ejoc.201403255
11. Cheng, P.; Clive, D. L. J. *J. Org. Chem.* **2012**, *77*, 3348–3364. doi:10.1021/jo3001657
12. Fischer, D.; Tomeba, H.; Pahadi, N. K.; Patil, N. T.; Yamamoto, Y. *Angew. Chem. Int. Ed.* **2007**, *46*, 4764–4766. doi:10.1002/anie.200701392
13. Meyers, A.I.; Willemsen, J. J. *Tetrahedron* **1998**, *54*, 10493–10511. doi:10.1016/S0040-4020(98)00501-8
14. John, J.; Thomas, J.; Parekh, N.; Dehaen, W. *Eur. J. Org. Chem.* **2015**, *2015*, 4922–4930. doi:10.1002/ejoc.201500459
15. Sharma, R.; Vadivel, S. K.; Duclos, R. I.; Makriyannis, A. *Tetrahedron Lett.* **2009**, *50*, 5780–5782. doi:10.1016/j.tetlet.2009.07.079
16. Ackermann, L.; Barfüsser, S.; Kornhaass, C.; Kapdi, A. R. *Org. Lett.* **2011**, *13*, 3082–3085. doi:10.1021/ol200986x
17. Sureshbabu, R.; Saravanan, V.; Dhayalan, V.; Mohanakrishnan, A. K. *Eur. J. Org. Chem.* **2011**, 922–935. doi:10.1002/ejoc.201001309
18. Summers, G. J.; Maseko, R. B.; Beebeejaun, B. M. P.; Summers, C. A. *J. Polym. Sci. A Polym. Chem.* **2011**, *49*, 2601–2614. doi:10.1002/pola.24692
19. Tang, X.; Zhang, D.; Jie, S.; Sun, W.-H.; Chen, J. *J. Organomet. Chem.* **2005**, *690*, 3918–3928. doi:10.1016/j.jorganchem.2005.05.026

20. Choi-Sledeski, Y. M.; Liang, G.; Nieduzak, T. R.; Poli, G. B.; Shum, P. W.-K.; Stoklosa, G. T.; Zhao, Z. Indolyl-piperidinyl benzylamines as beta-tryptase inhibitors. WO 2011079102, Jun 30,2011.
21. Yang, J.; Jiang, C.; Zhang, Y.; Yang, R.; Yang, W.; Hou, Q.; Cao, Y. *Macromolecules* **2004**, *37*, 1211–1218. doi:10.1021/ma035743u
22. Denmark, S. E.; Cullen, L. R. *J. Org. Chem.* **2015**, *80*, 11818–11848. doi:10.1021/acs.joc.5b01759
23. Chakraborty, C.; Bera, M. K.; Rana, U.; Malik, S. *Chem. Commun.* **2015**, *51*, 13123–13126. doi:10.1039/c5cc04275e
24. Singh, V.; Wang, S.; Kool, E. T. *J. Am. Chem. Soc.* **2013**, *135*, 6184–6191. doi:10.1021/ja4004393
25. Hosseinzadeh, R.; Mohadjerani, M.; Pooryousef, M. *Luminescence* **2015**, *30*, 549–555. doi:10.1002/bio.2776
26. Göbel, D.; Clamor, N.; Nachtsheim, B. J. *Org. Biomol. Chem.* **2018**, *16*, 4071–4075. doi:10.1039/c8ob01072b
27. Meyers, A. I.; Avila, W. B. *J. Org. Chem.* **1981**, *46*, 3881–3886. doi:10.1021/jo00332a024
28. Bos, M. E.; Wulff, W. D.; Miller, R. A.; Chamberlin, S.; Brandvold, T. A. *J. Am. Chem. Soc.* **1991**, *113*, 9293–9319. doi:10.1021/ja00024a040
29. Lingel, A.; Sendzik, M.; Huang, Y.; Shultz, M. D.; Cantwell, J.; Dillon, M. P.; Fu, X.; Fuller, J.; Gabriel, T.; Gu, J.; Jiang, X.; Li, L.; Liang, F.; McKenna, M.; Qi, W.; Rao, W.; Sheng, X.; Shu, W.; Sutton, J.; Taft, B.; Wang, L.; Zeng, J.; Zhang, H.; Zhang, M.; Zhao, K.; Lindvall, M.; Bussiere, D. E. *J. Med. Chem.* **2017**, *60*, 415–427. doi:10.1021/acs.jmedchem.6b01473
30. Bedford, R. B.; Brenner, P. B.; Carter, E.; Gallagher, T.; Murphy, D. M.; Pye, D. R. *Organometallics* **2014**, *33*, 5940–5943. doi:10.1021/om500847j
31. Moon, D. J.; Al-Amin, M.; Lewis, R. S.; Arnold, K. M.; Yap, G. P. A.; Sims-Mourtada, J.; Chain, W. J. *Eur. J. Org. Chem.* **2018**, 3348–3351. doi:10.1002/ejoc.201800707
32. Reddy, B. R. P.; Chowdhury, S.; Auffrant, A.; Gosmini, C. *Adv. Synth. Catal.* **2018**, *360*, 3026–3029. doi:10.1002/adsc.201800468
33. Stulgies, B.; Prinz, P.; Magull, J.; Rauch, K.; Meindl, K.; Rühl, S.; Meijere, A. de. *Chem. Eur. J.* **2004**, *11*, 308–320. doi:10.1002/chem.200400723
34. Stetter, H.; Goebel, P. *Chem. Ber.* **1962**, *95*, 1039–1042. doi:10.1002/cber.19620950435
35. Newman, S. G.; Lautens, M. *J. Am. Chem. Soc.* **2010**, *132*, 11416–11417. doi:10.1021/ja1052335
36. Beets, M. G. J.; Meerburg, W.; van Essen, H. *Recl. Trav. Chim. Pays-Bas* **1959**, *78*, 570–585. doi:10.1002/recl.19590780804
37. Karamé, I.; Jahjah, M.; Messaoudi, A.; Tommasino, M.L.; Lemaire, M. *Tetrahedron: Asymmetry* **2004**, *15*, 1569–1581. doi:10.1016/j.tetasy.2004.03.023
38. Philp, D.; Gramlich, V.; Seiler, P.; Diederich, F. *J. Chem. Soc., Perkin Trans. 2* **1995**, *32*, 875–886. doi:10.1039/P29950000875

39. Rostovtsev, V. V.; Green, L. G.; Fokin, V. V.; Sharpless, K. B. *Angew. Chem. Int. Ed.* **2002**, *41*, 2596–2599. doi:10.1002/1521-3773(20020715)41:14<2596:AID-ANIE2596>3.0.CO;2-4
40. Schmid, J.; Frey, W.; Peters, R. *Organometallics* **2017**, *36*, 4313–4324. doi:10.1021/acs.organomet.7b00729

6 NMR-Spectra

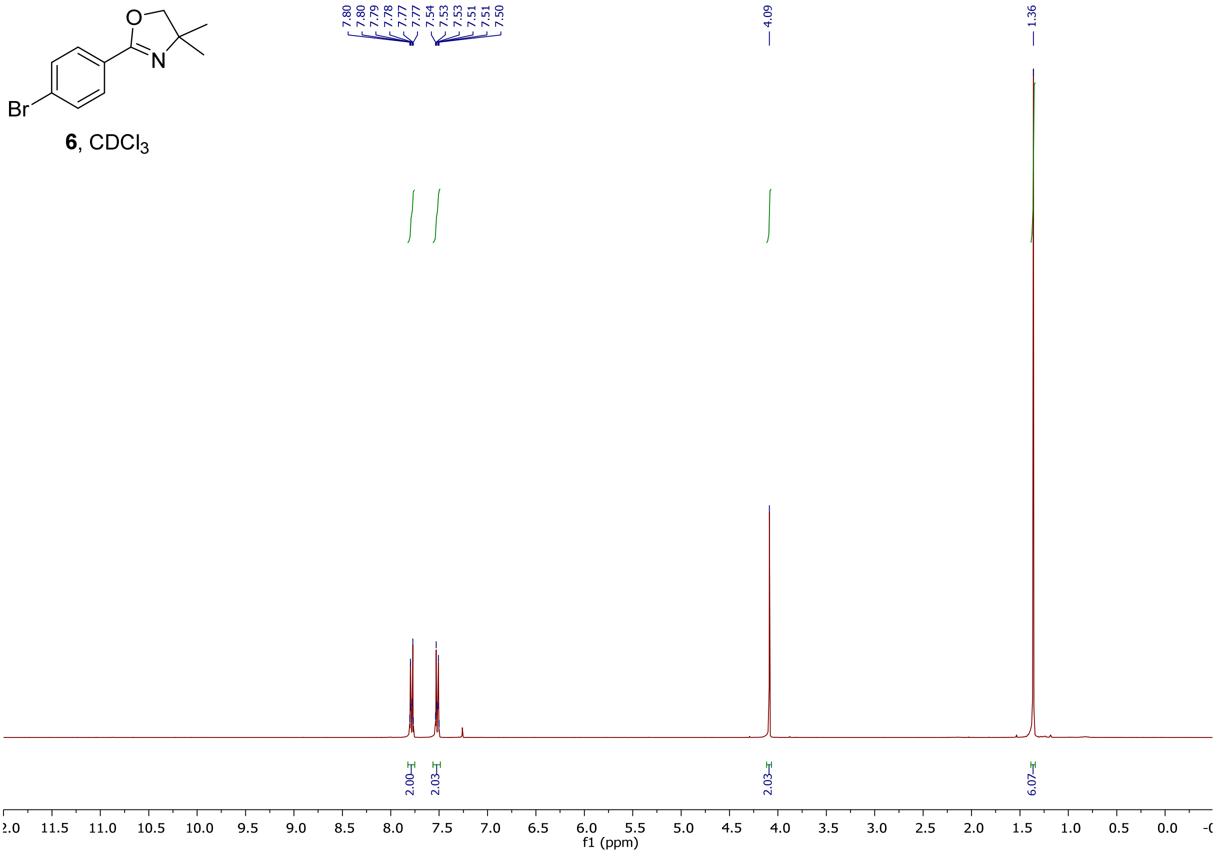

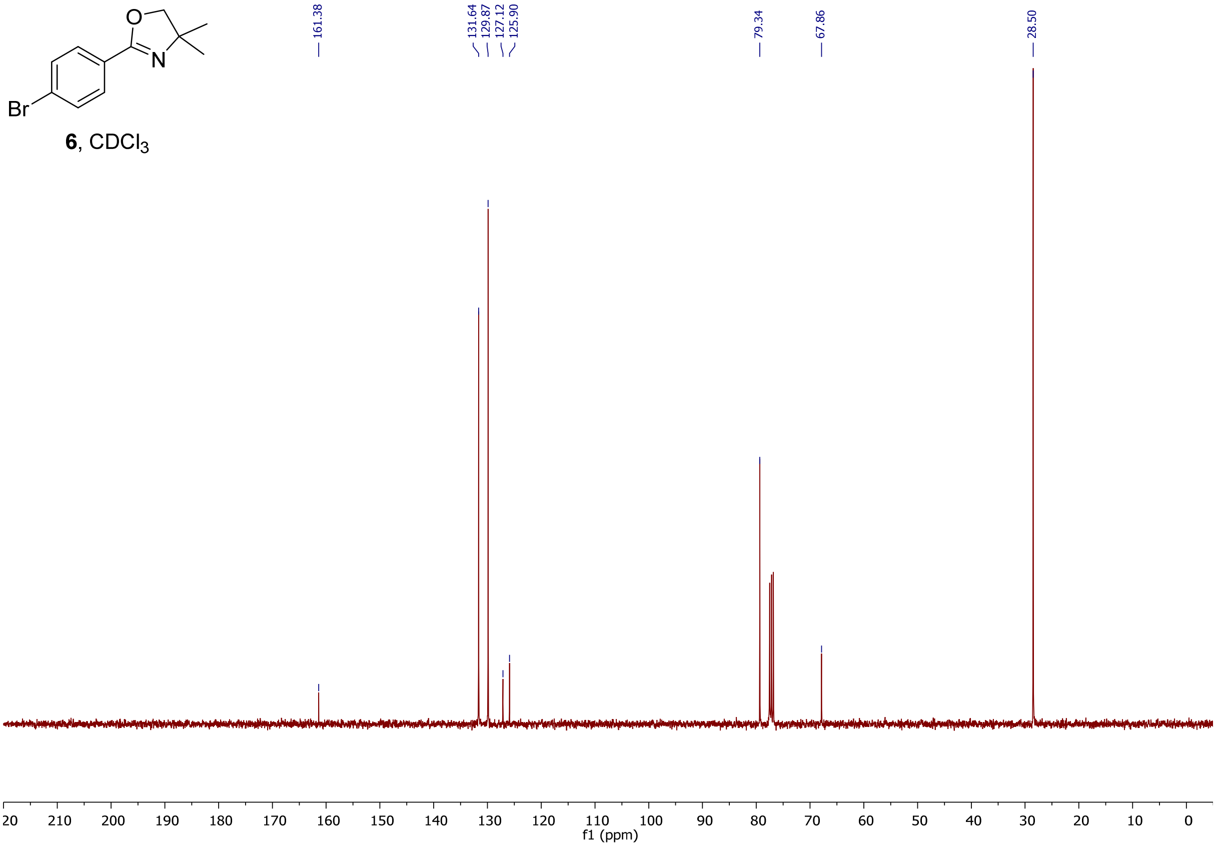

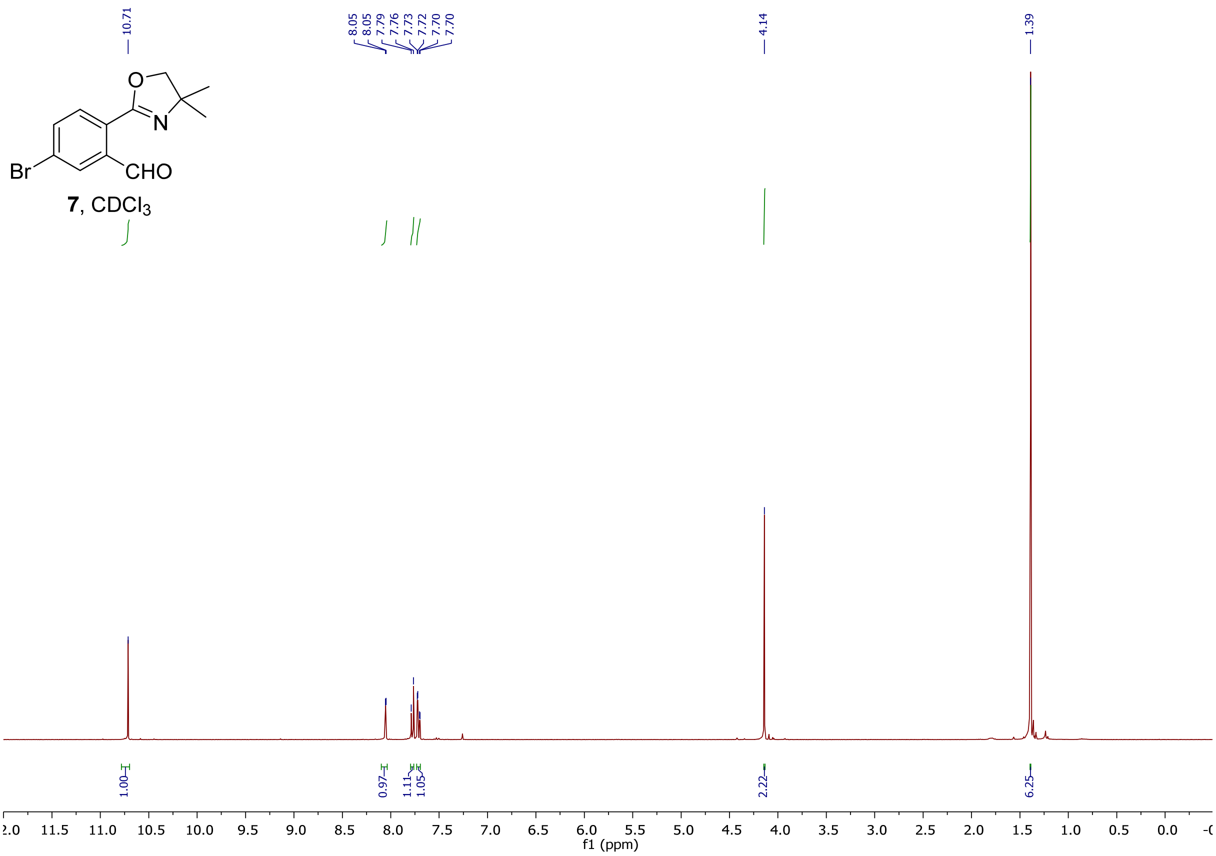

7, $CDCl_3$

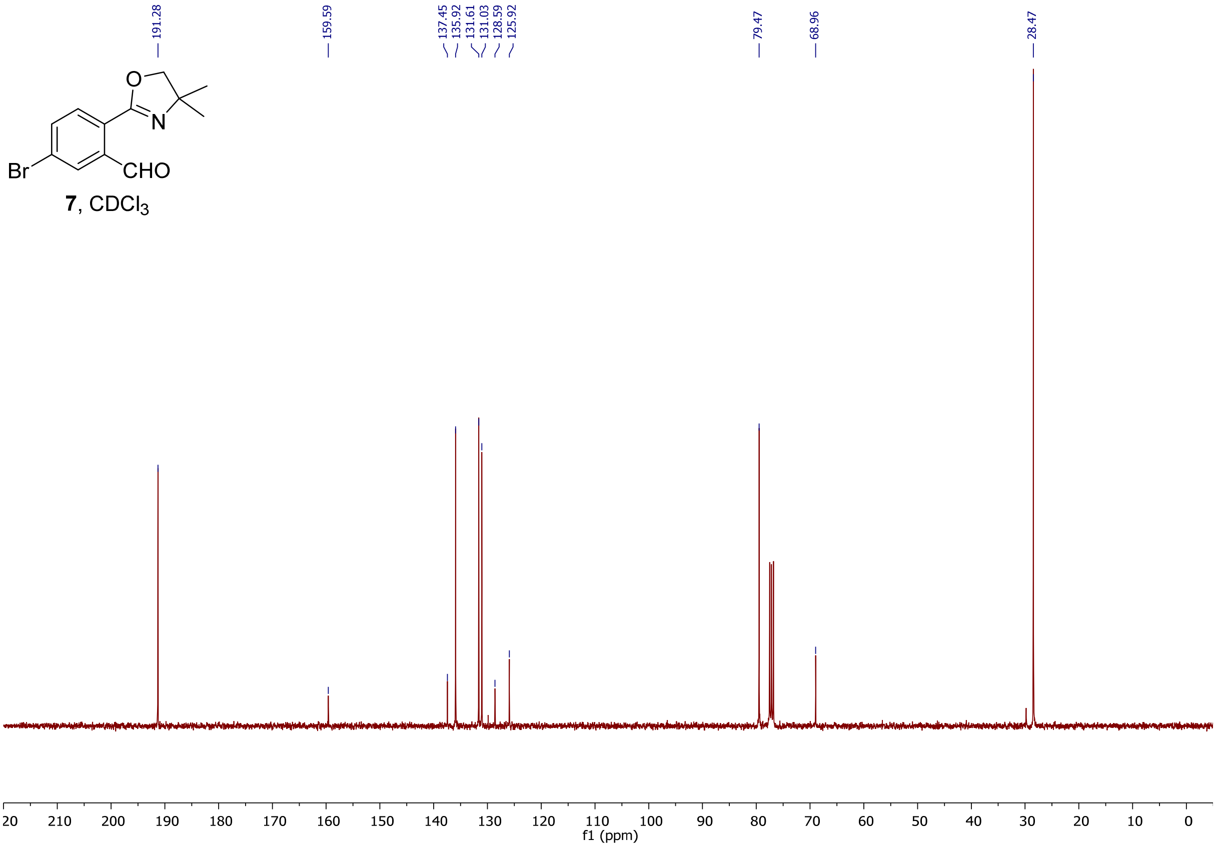

7, $CDCl_3$

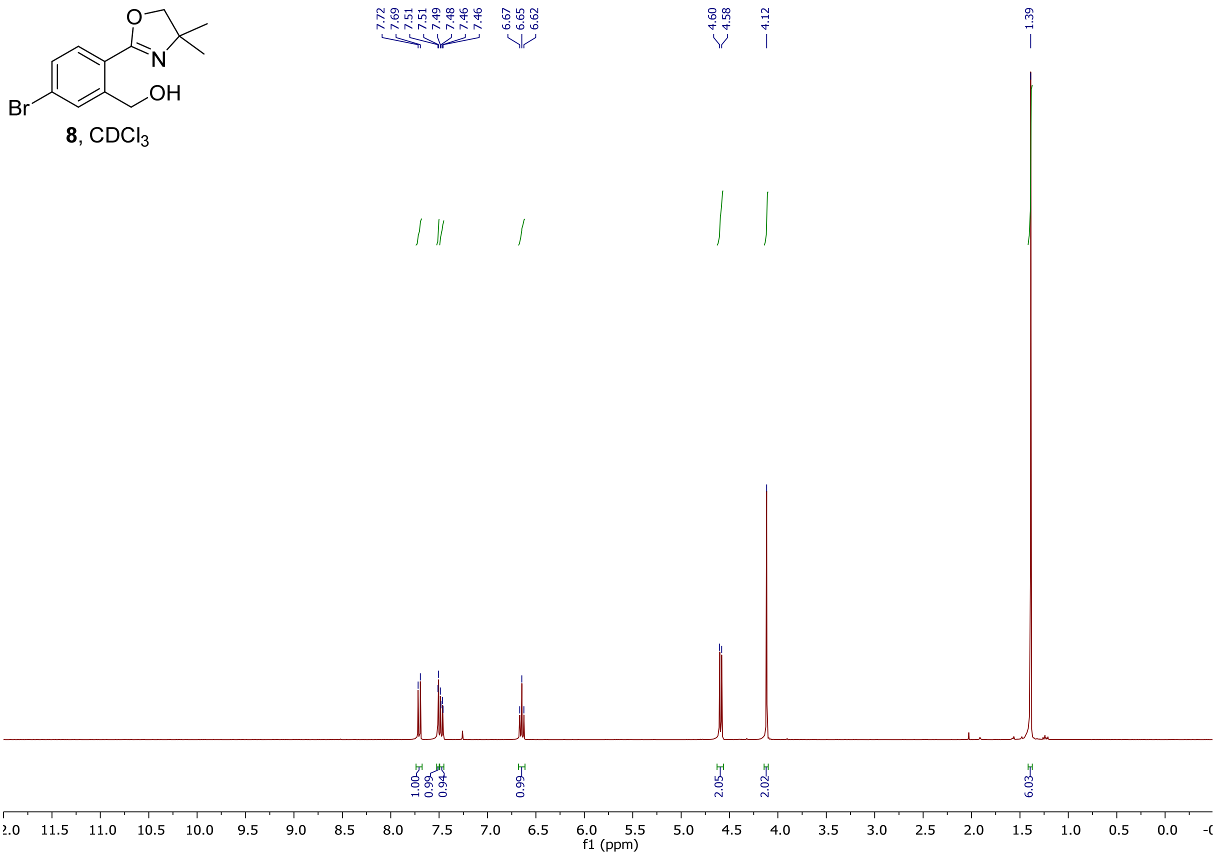
8, CDCl3
7.72
7.69
7.51
7.51
7.49
7.48
7.46
7.46
6.67
6.65
6.62
4.60
4.58
4.12
1.39
1.00
0.99
0.94
0.99
2.05
2.02
6.03
f1 (ppm)

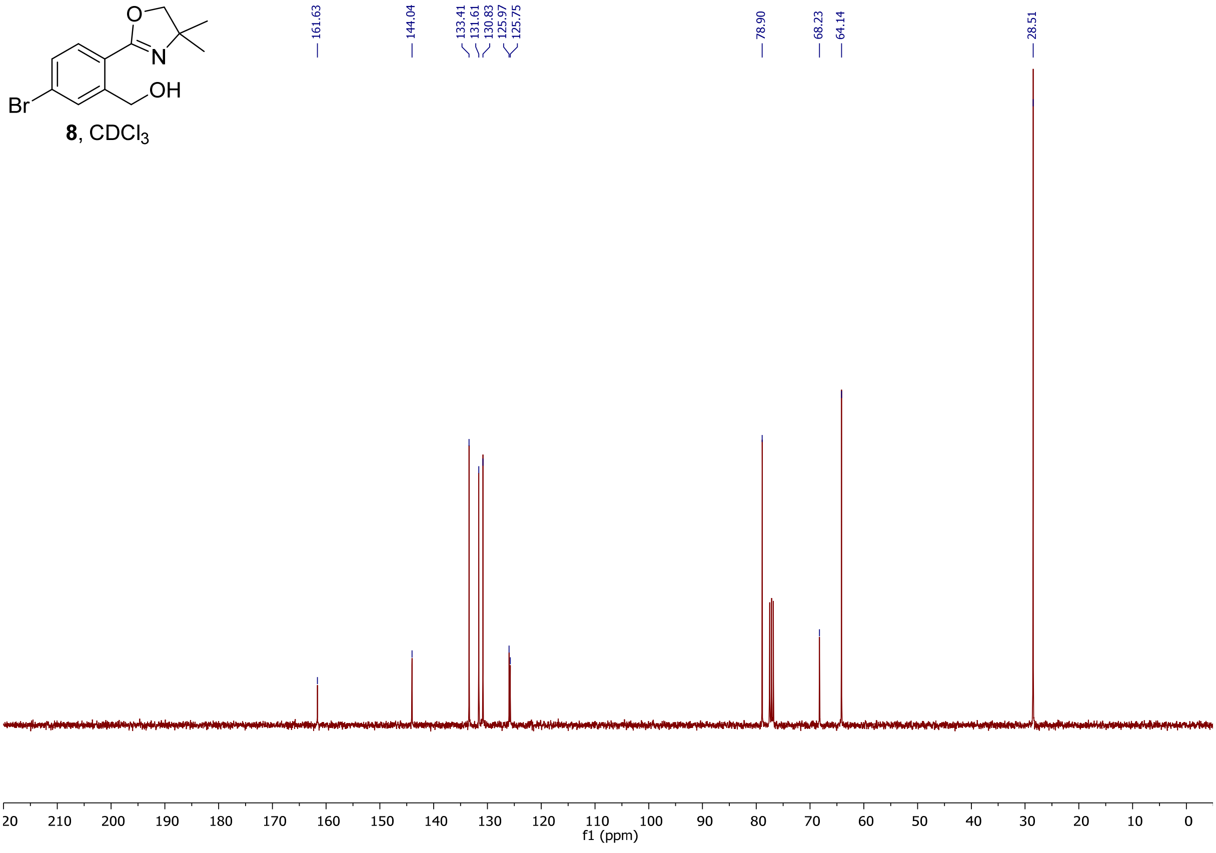
8, CDCl3
161.63
144.04
133.41
131.61
130.83
125.97
125.75
78.90
68.23
64.14
28.51
f1 (ppm)

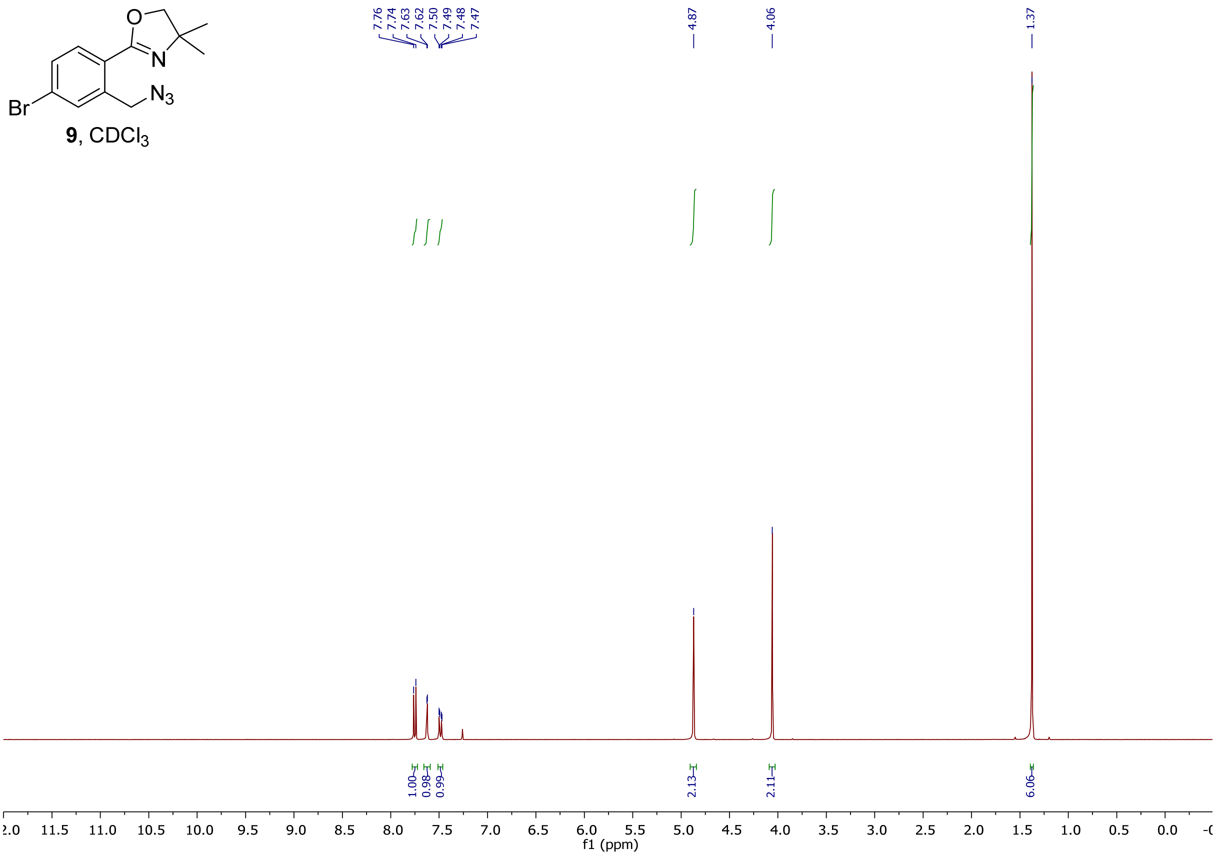
9, CDCl3
7.76
7.74
7.63
7.62
7.50
7.49
7.48
7.47
4.87
4.06
1.37
1.00
0.98
0.99
2.13
2.11
6.06
f1 (ppm)

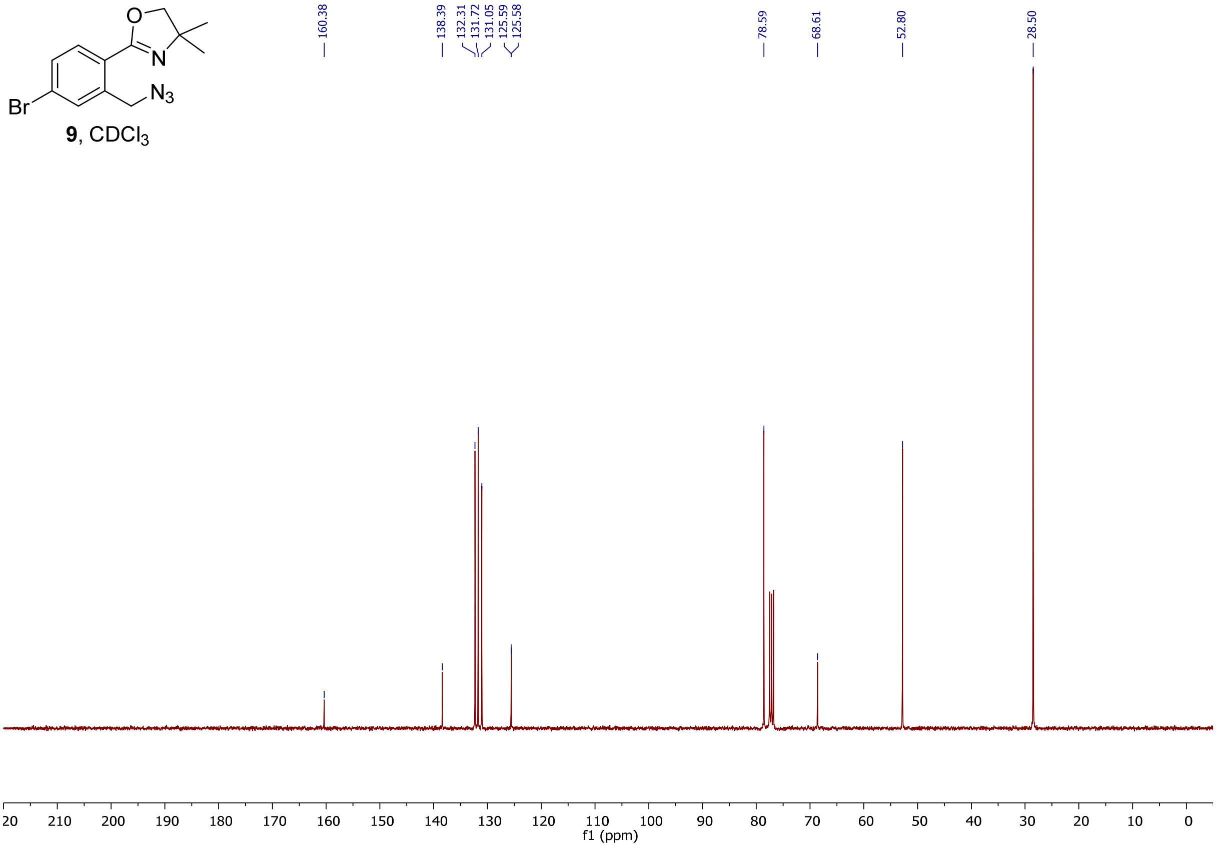
9, CDCl3
160.38
138.39
132.31
131.72
131.05
125.59
125.58
78.59
68.61
52.80
28.50
f1 (ppm)

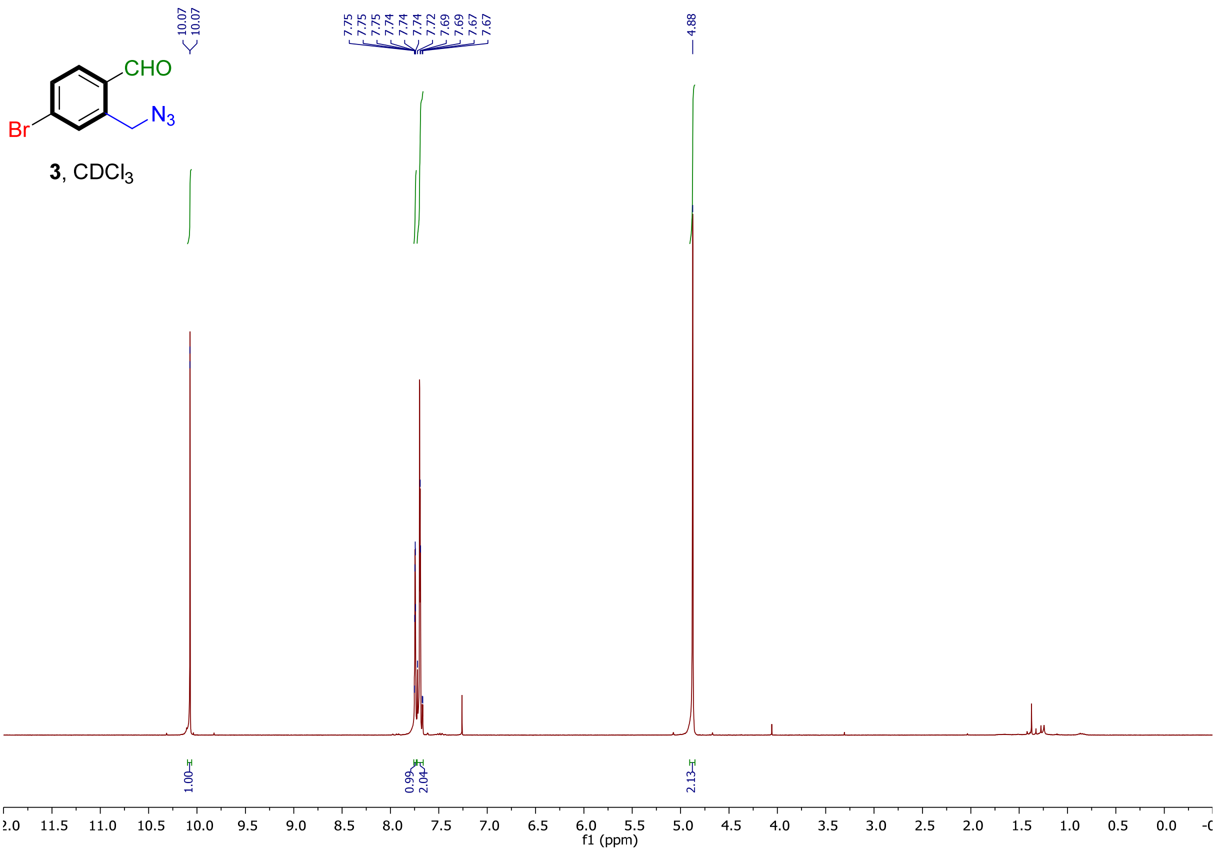
10.07
10.07
7.75
7.75
7.75
7.74
7.74
7.74
7.72
7.69
7.69
7.67
7.67
4.88
CHO
N_3
Br
3, $CDCl_3$
1.00
0.99
2.04
2.13
f1 (ppm)

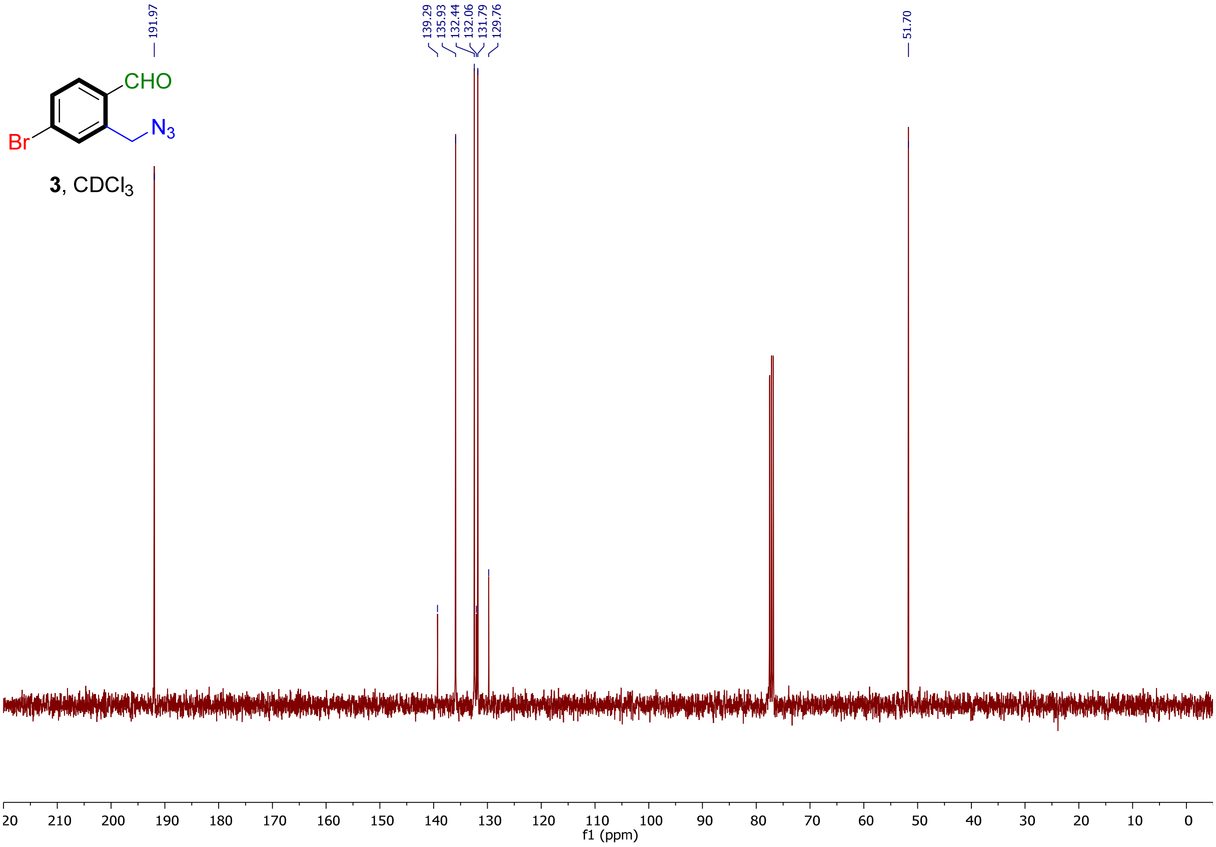
191.97
139.29
135.93
132.44
132.06
131.79
129.76
51.70
CHO
N_3
Br
3, $CDCl_3$
f1 (ppm)

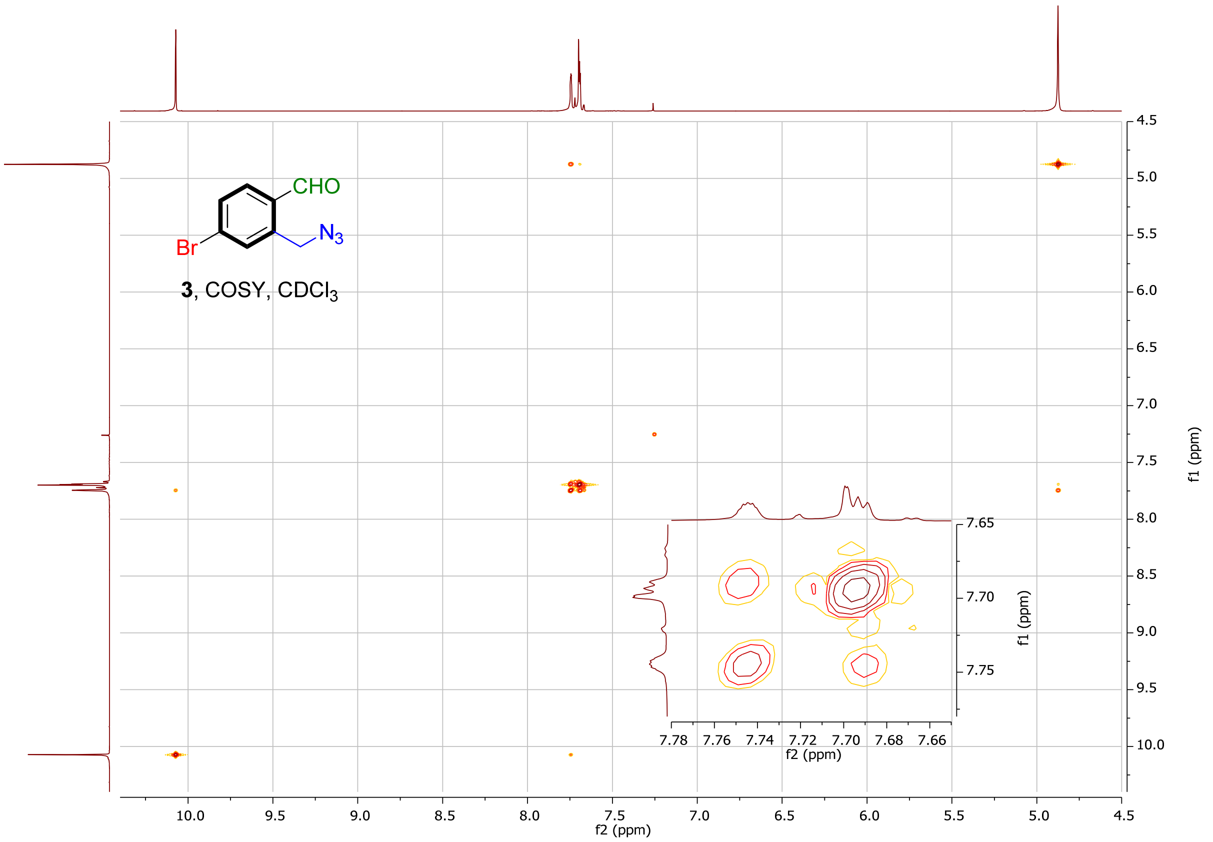
CHO
N3
Br
3, COSY, CDCl3
f2 (ppm)
f1 (ppm)

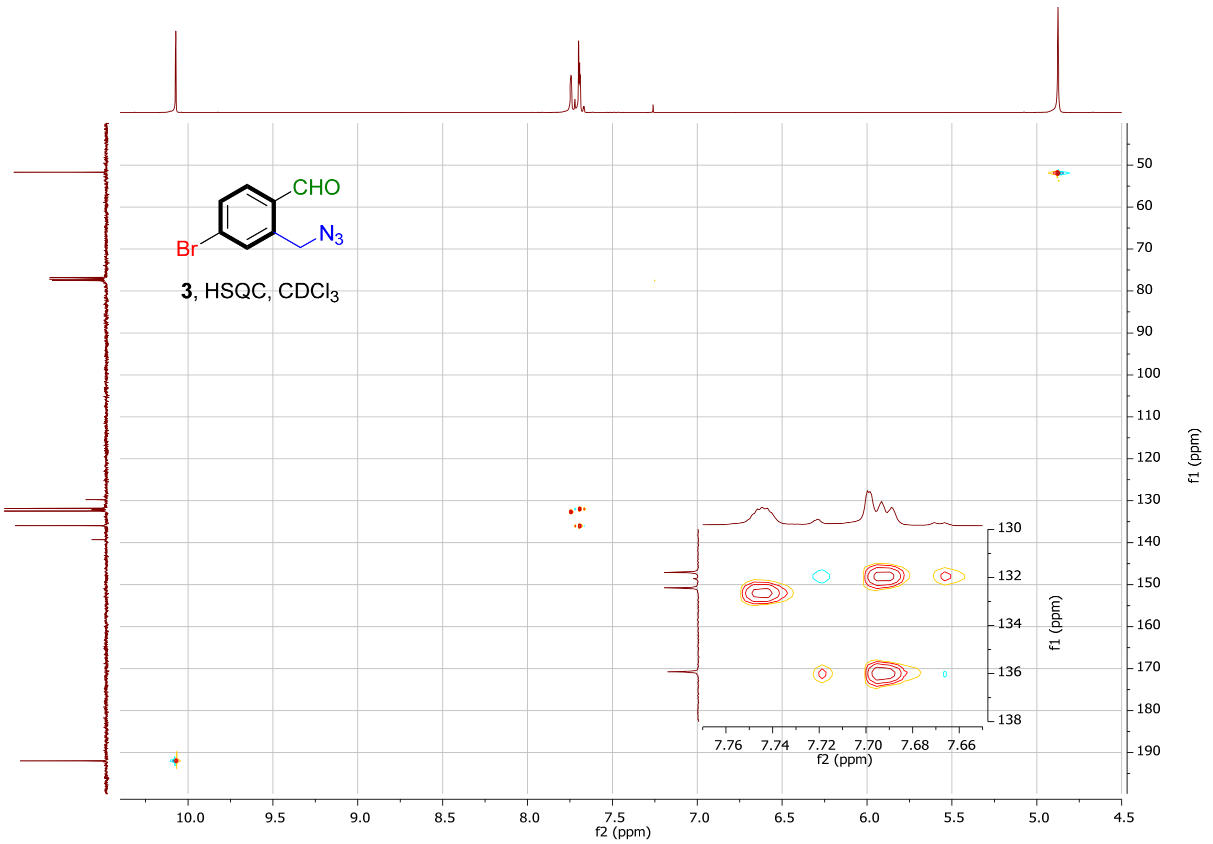
CHO
N3
Br
3, HSQC, CDCl3
f2 (ppm)
f1 (ppm)

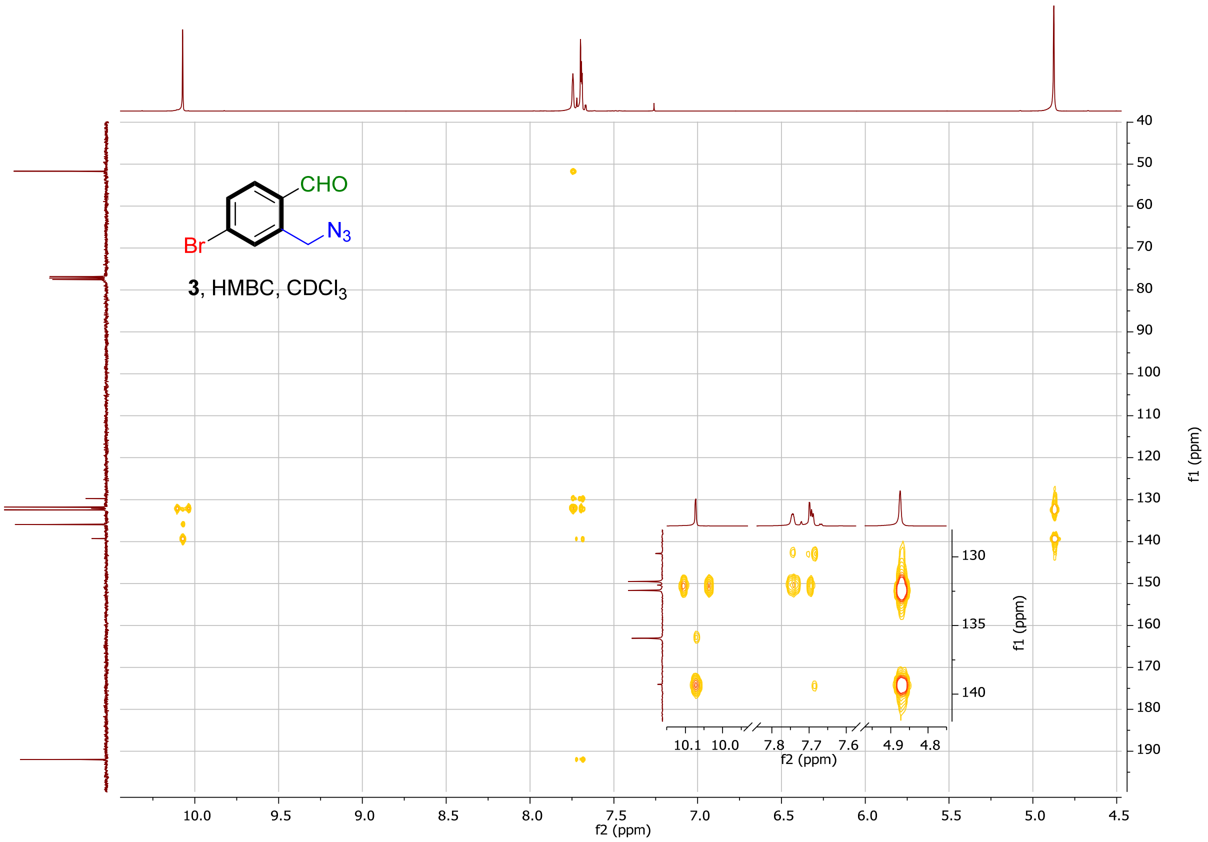
CHO
N3
Br
3, HMBC, CDCl3
f2 (ppm)
f1 (ppm)

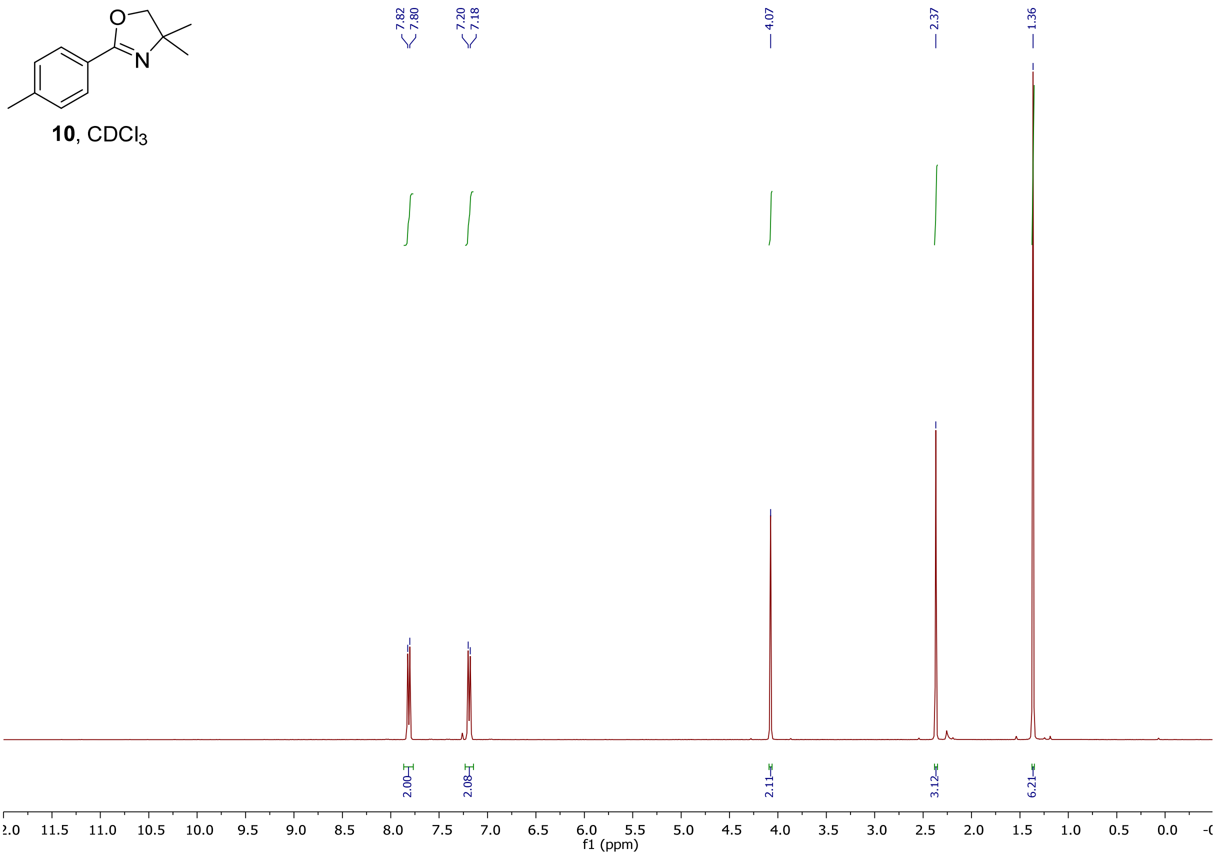
O
N
10, CDCl3
7.82
7.80
7.20
7.18
4.07
2.37
1.36
2.00
2.08
2.11
3.12
6.21
f1 (ppm)

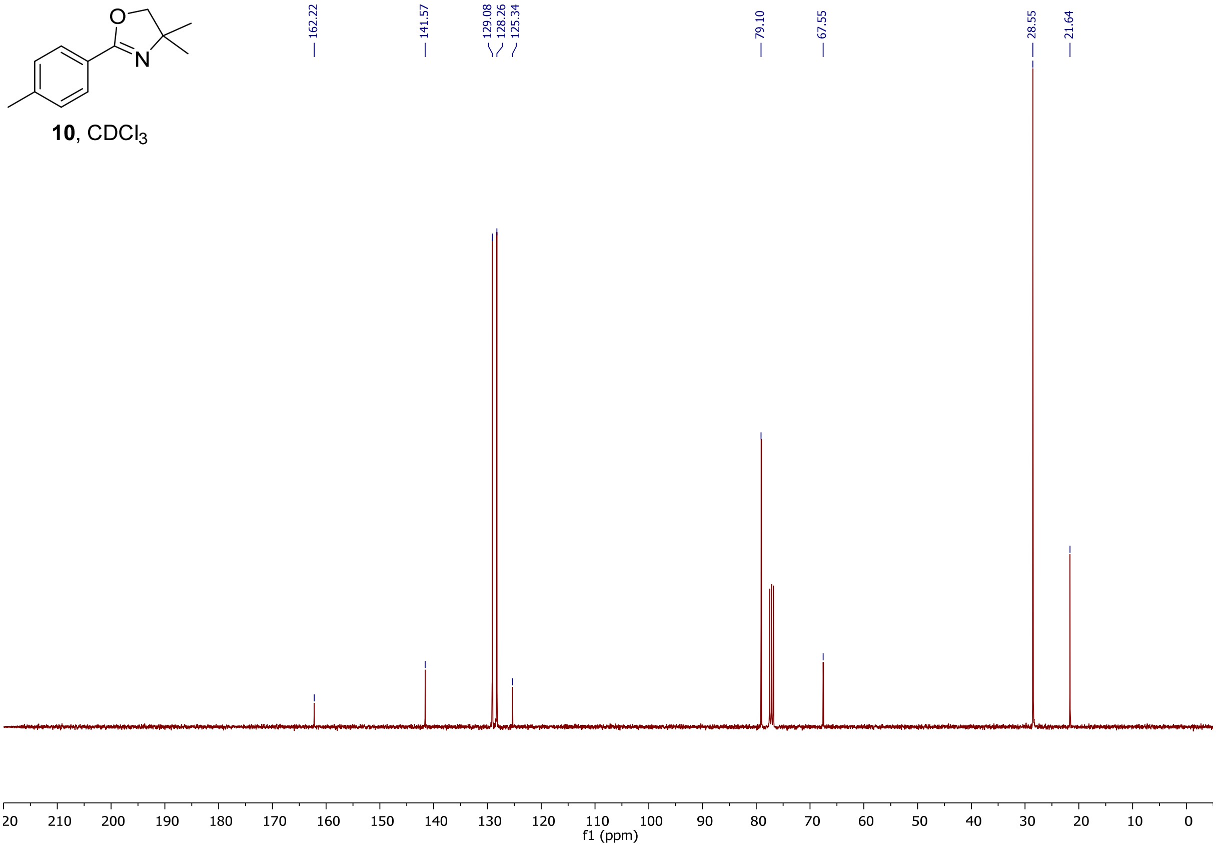
O
N
10, CDCl3
162.22
141.57
129.08
128.26
125.34
79.10
67.55
28.55
21.64
f1 (ppm)

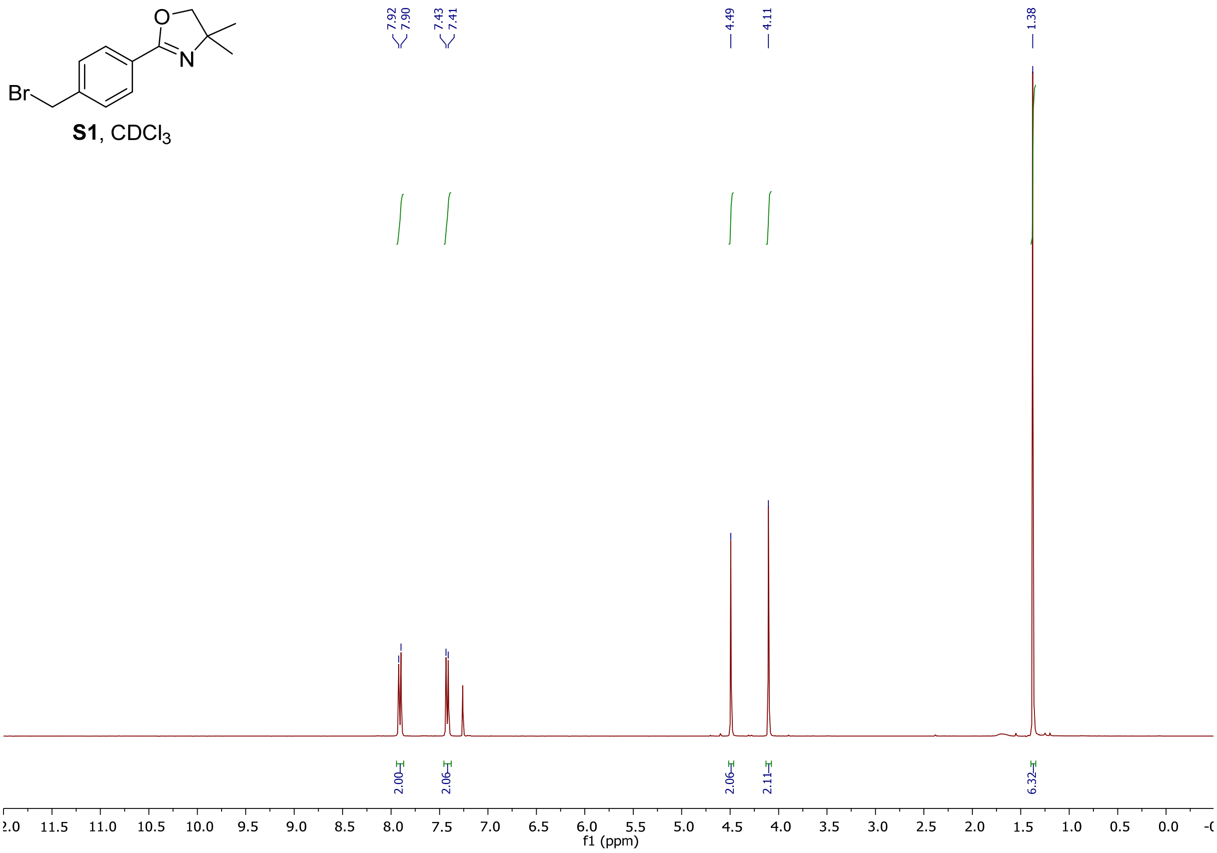

Br
O
N
S1, CDCl3
7.92
7.90
7.43
7.41
4.49
4.11
1.38
2.00
2.06
2.06
2.11
6.32
f1 (ppm)

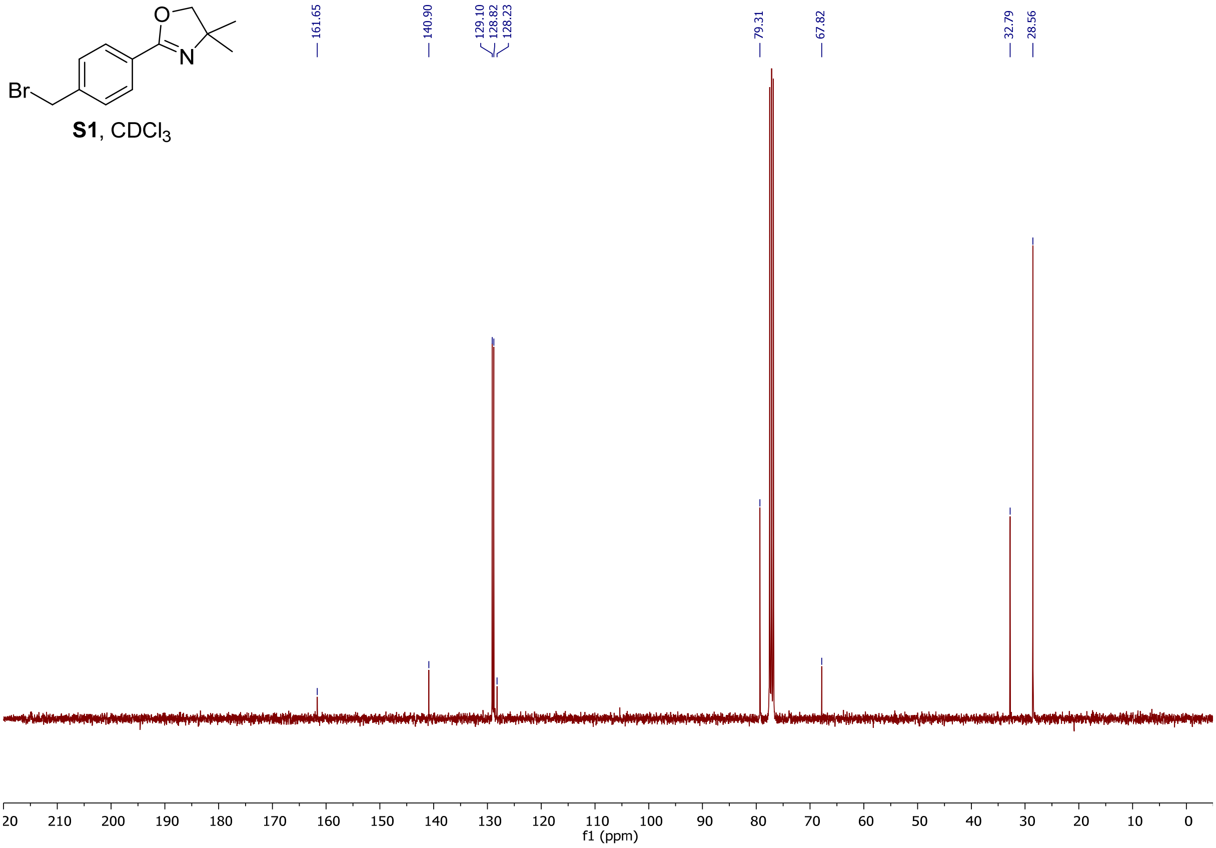

Br
O
N
S1, CDCl3
161.65
140.90
129.10
128.82
128.23
79.31
67.82
32.79
28.56
f1 (ppm)

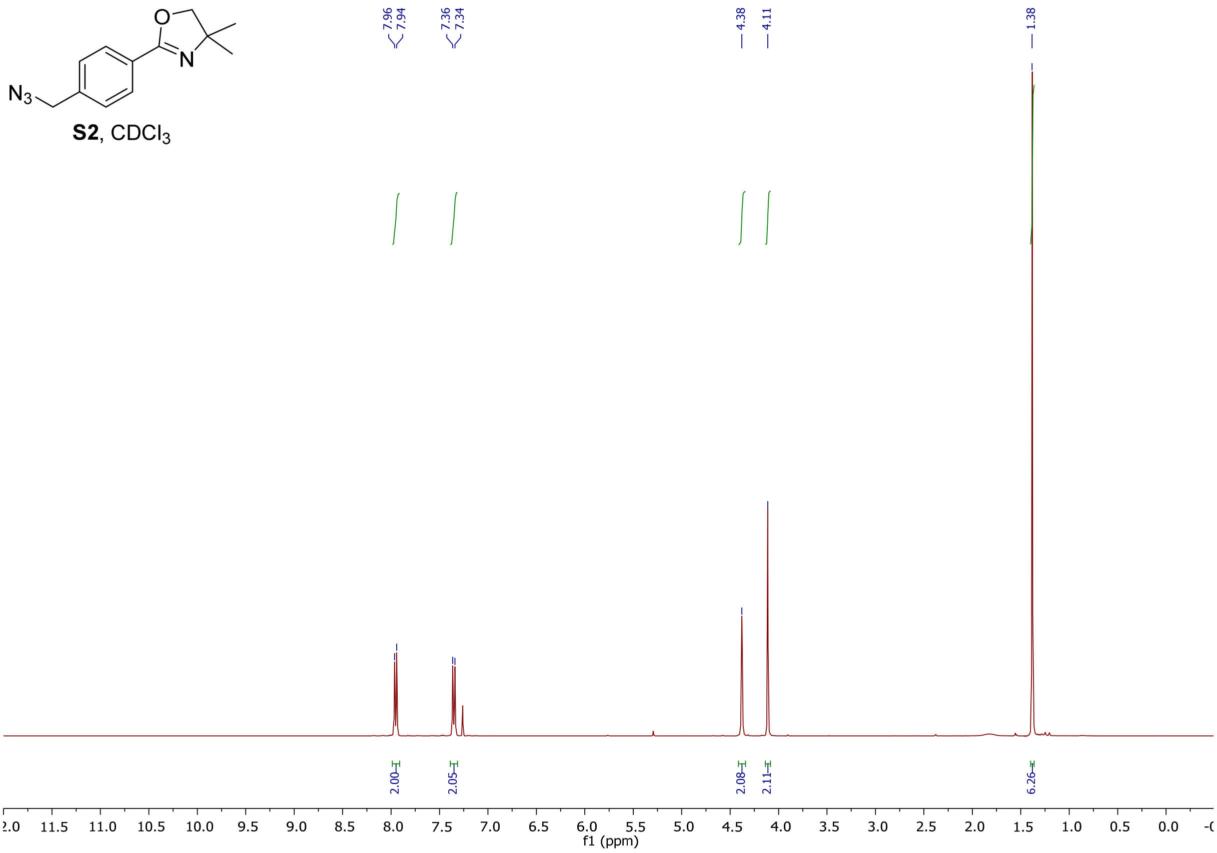

N3
S2, CDCl3
7.96
7.94
7.36
7.34
4.38
4.11
1.38
2.00
2.05
2.08
2.11
6.26
f1 (ppm)

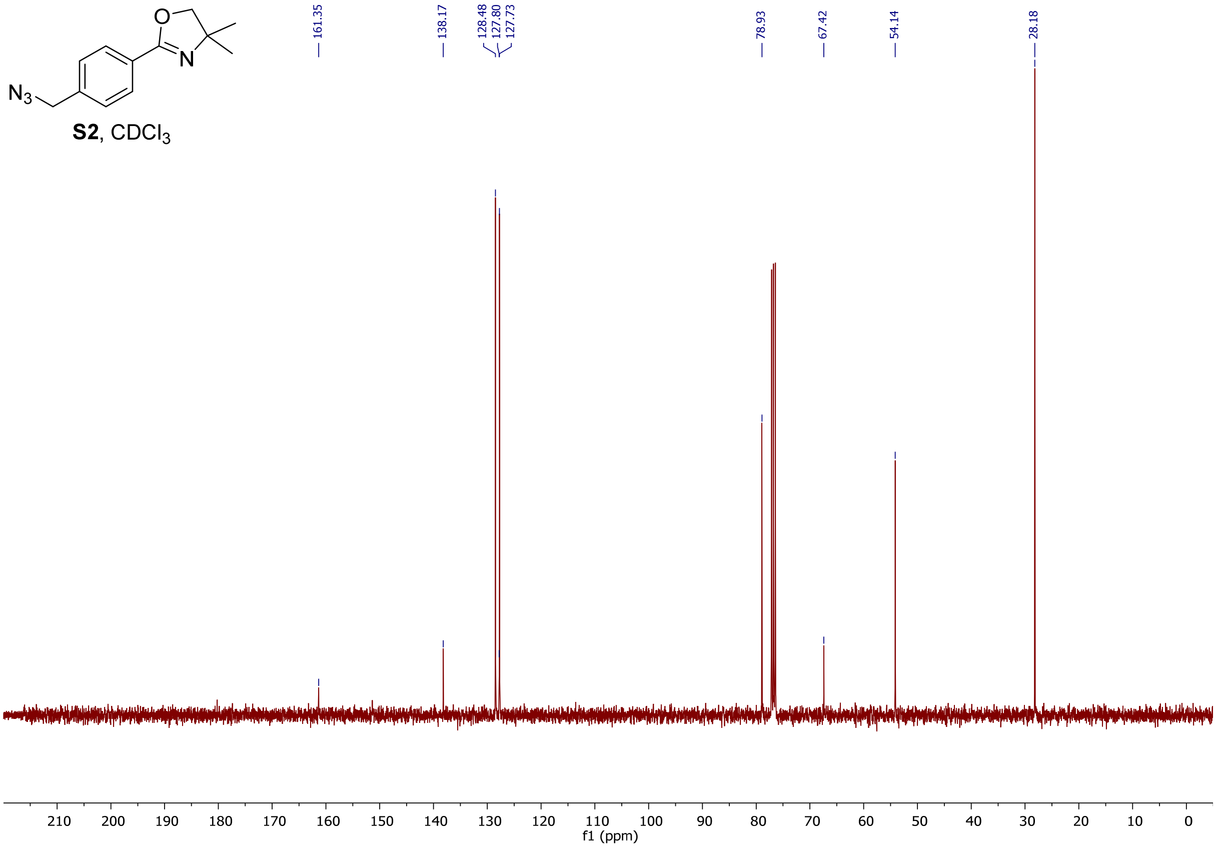

N3
S2, CDCl3
161.35
138.17
128.48
127.80
127.73
78.93
67.42
54.14
28.18
f1 (ppm)

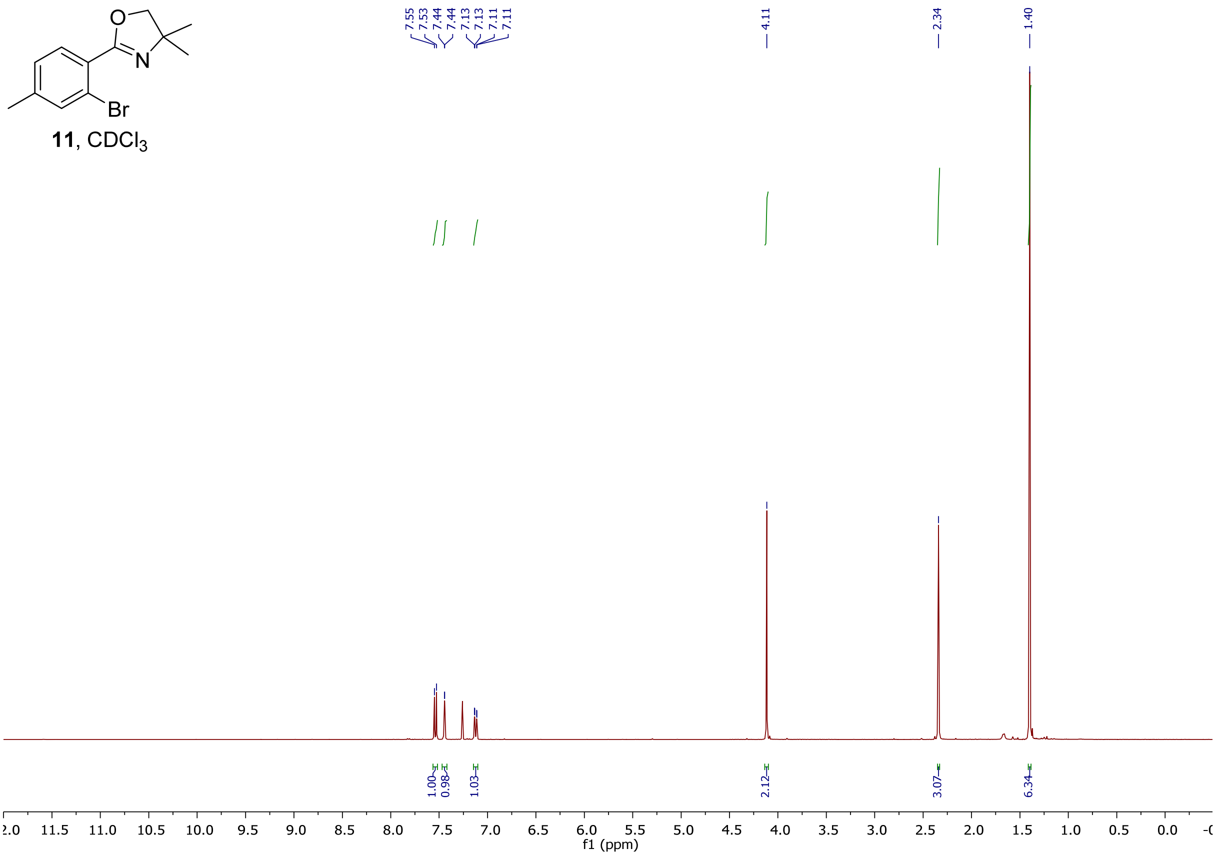
O
N
Br
11, CDCl3
7.55
7.53
7.44
7.44
7.13
7.13
7.11
7.11
4.11
2.34
1.40
1.00
0.98
1.03
2.12
3.07
6.34
f1 (ppm)

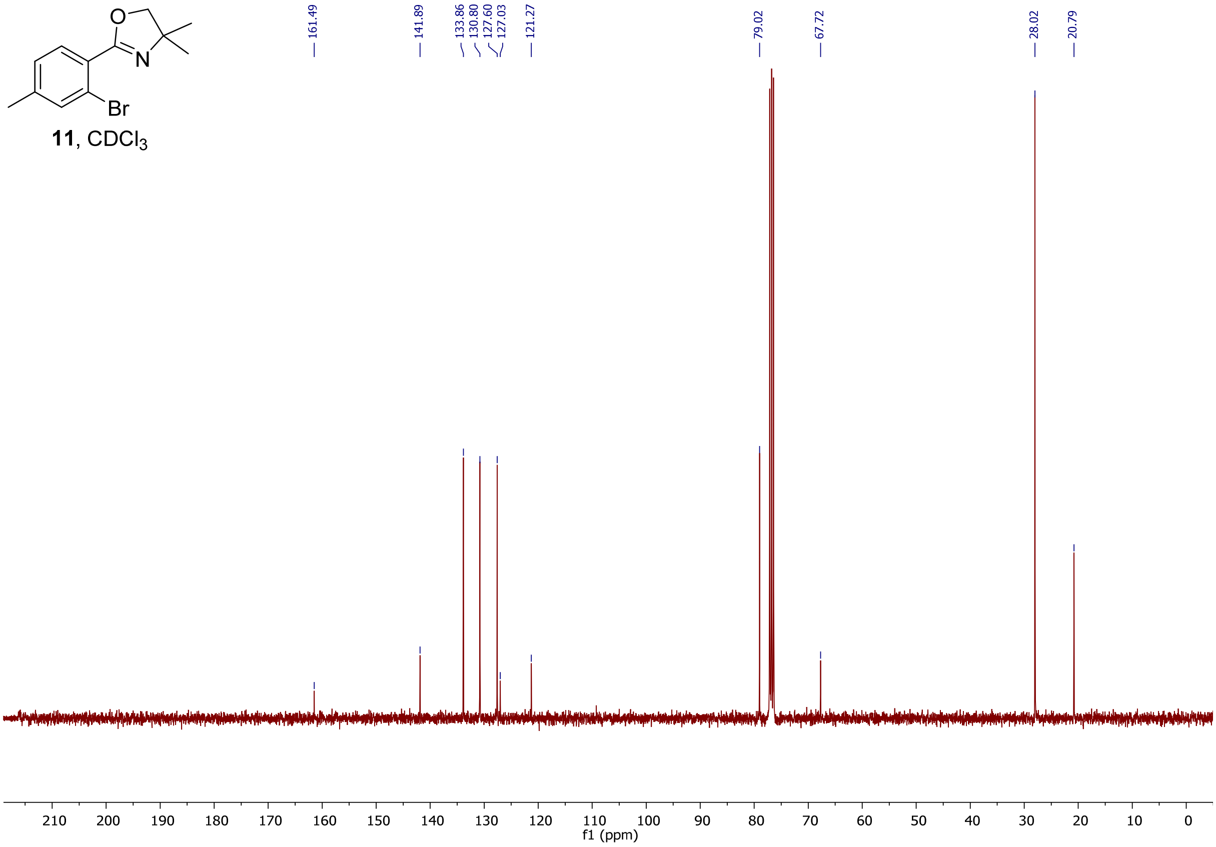
O
N
Br
11, CDCl3
161.49
141.89
133.86
130.80
127.60
127.03
121.27
79.02
67.72
28.02
20.79
f1 (ppm)

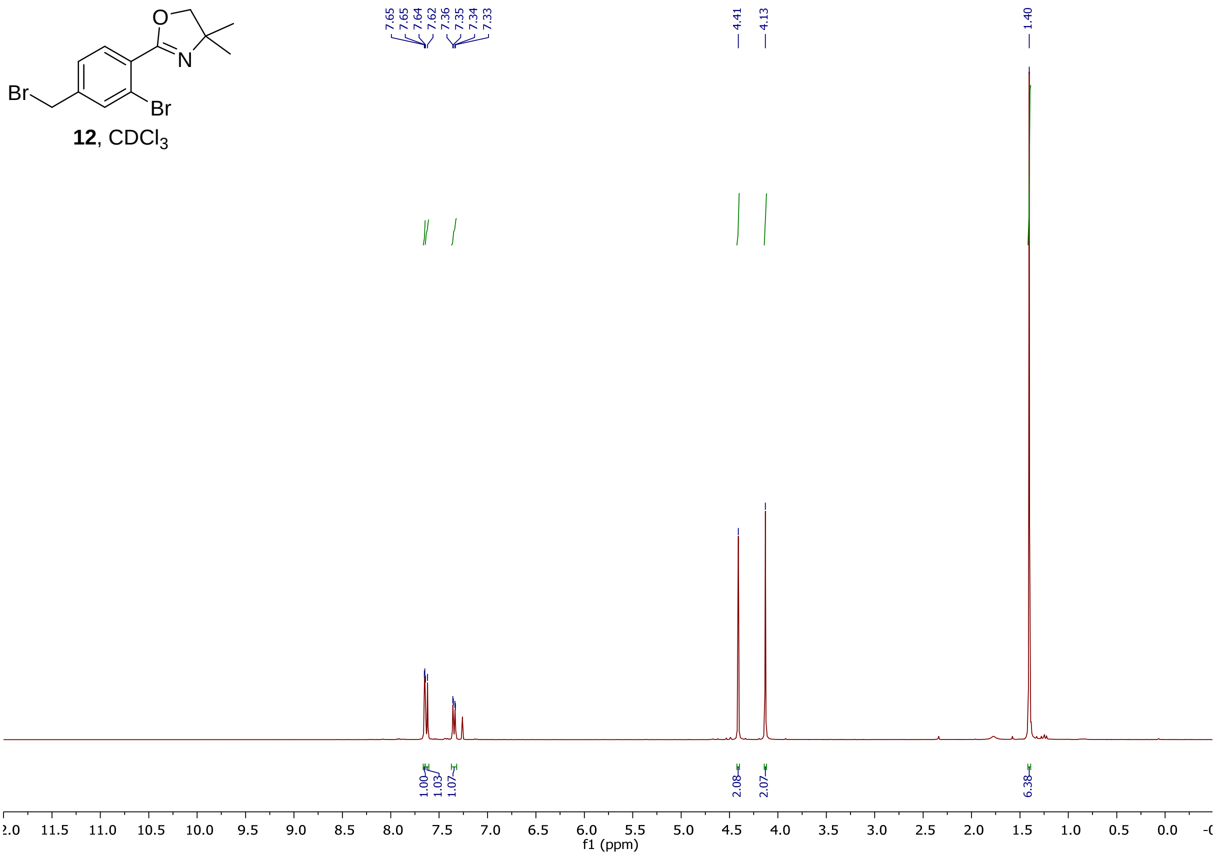

12, $CDCl_3$

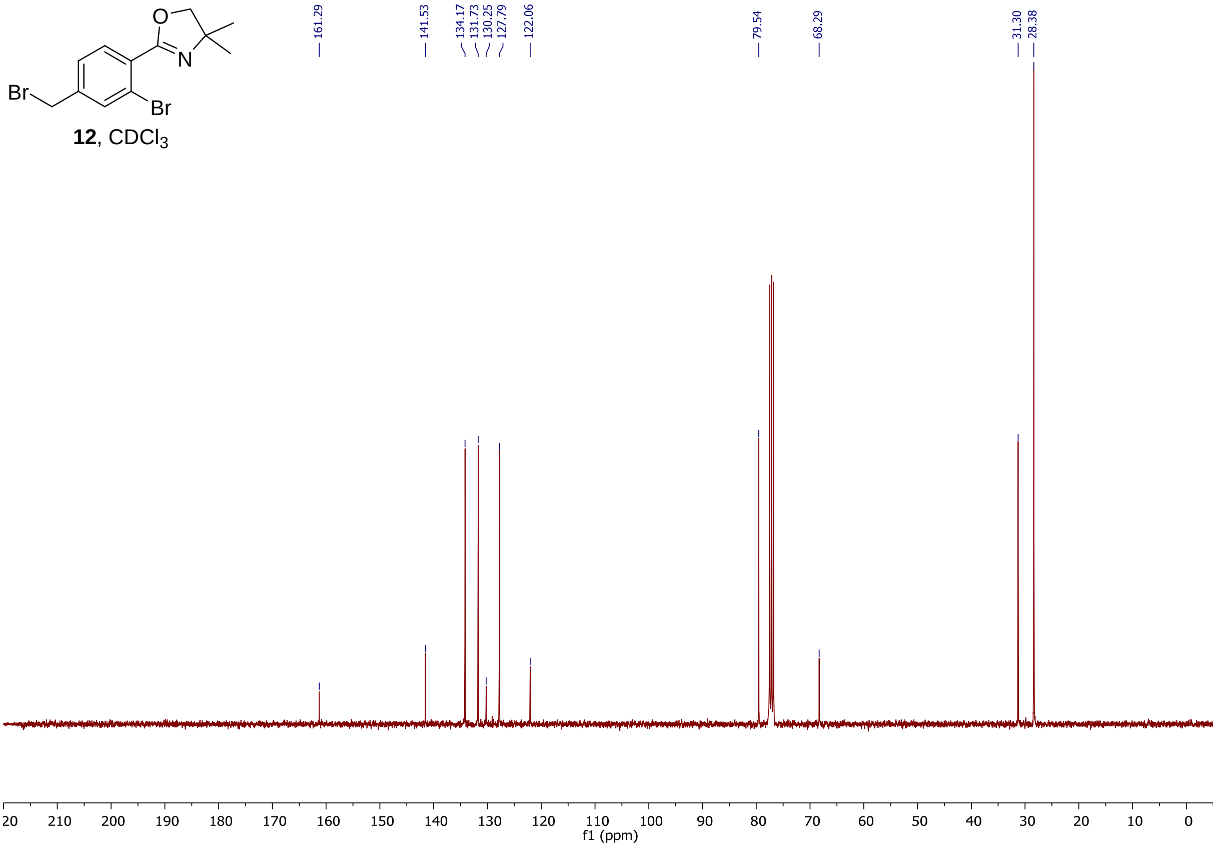

12, $CDCl_3$

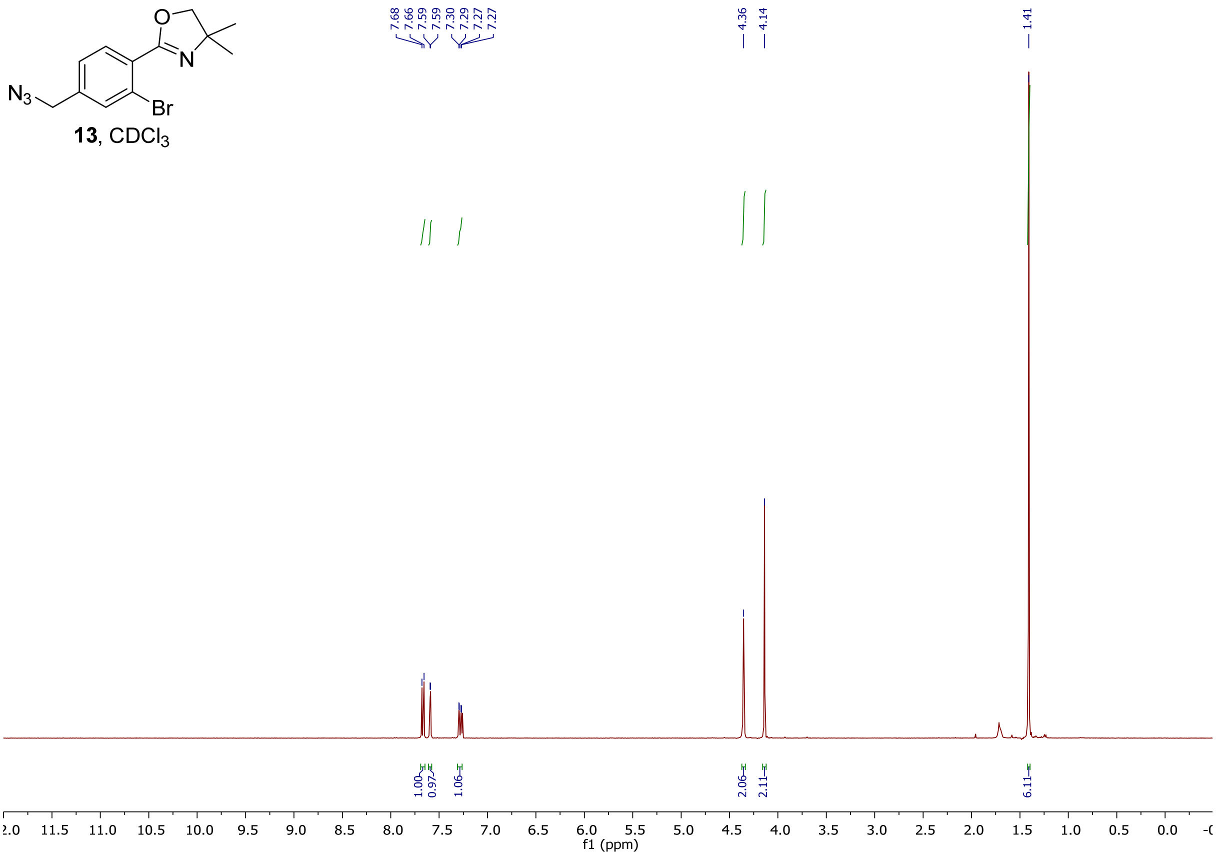

13, $CDCl_3$

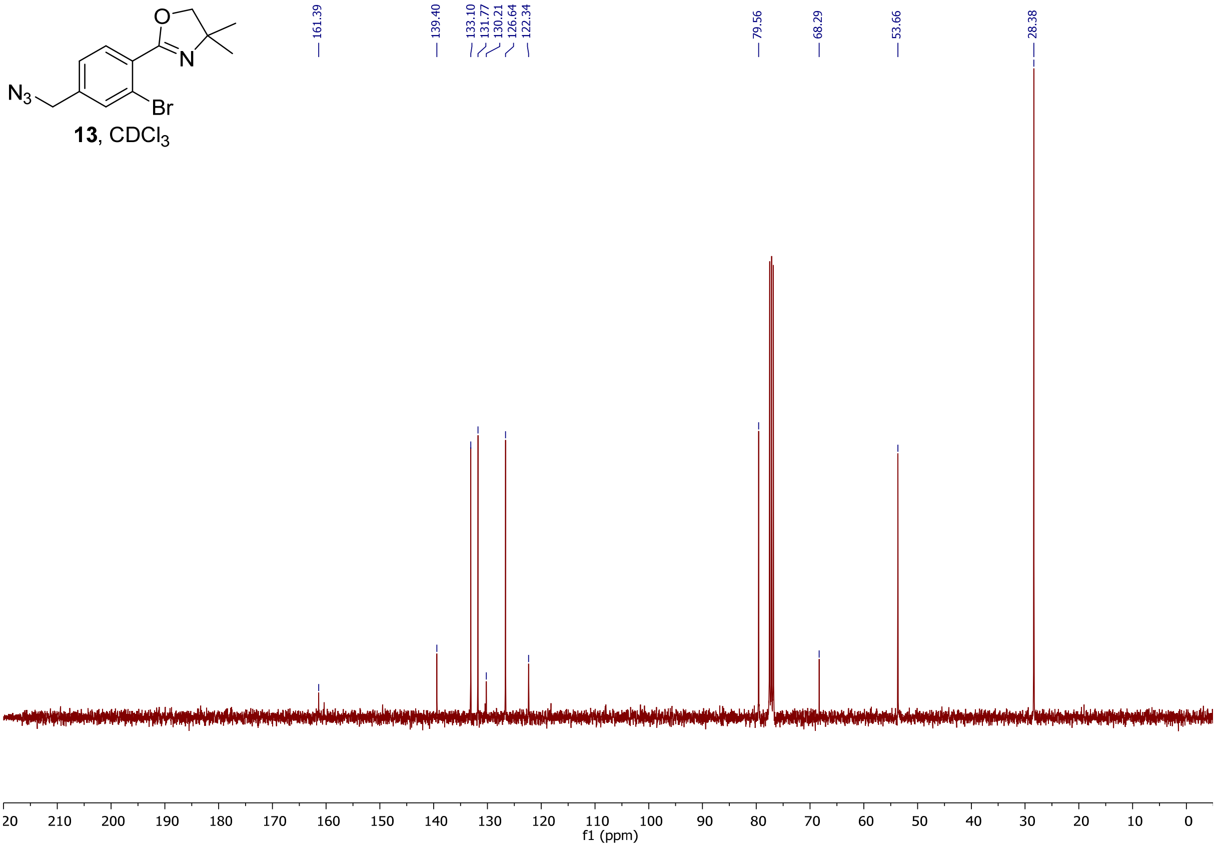

13, $CDCl_3$

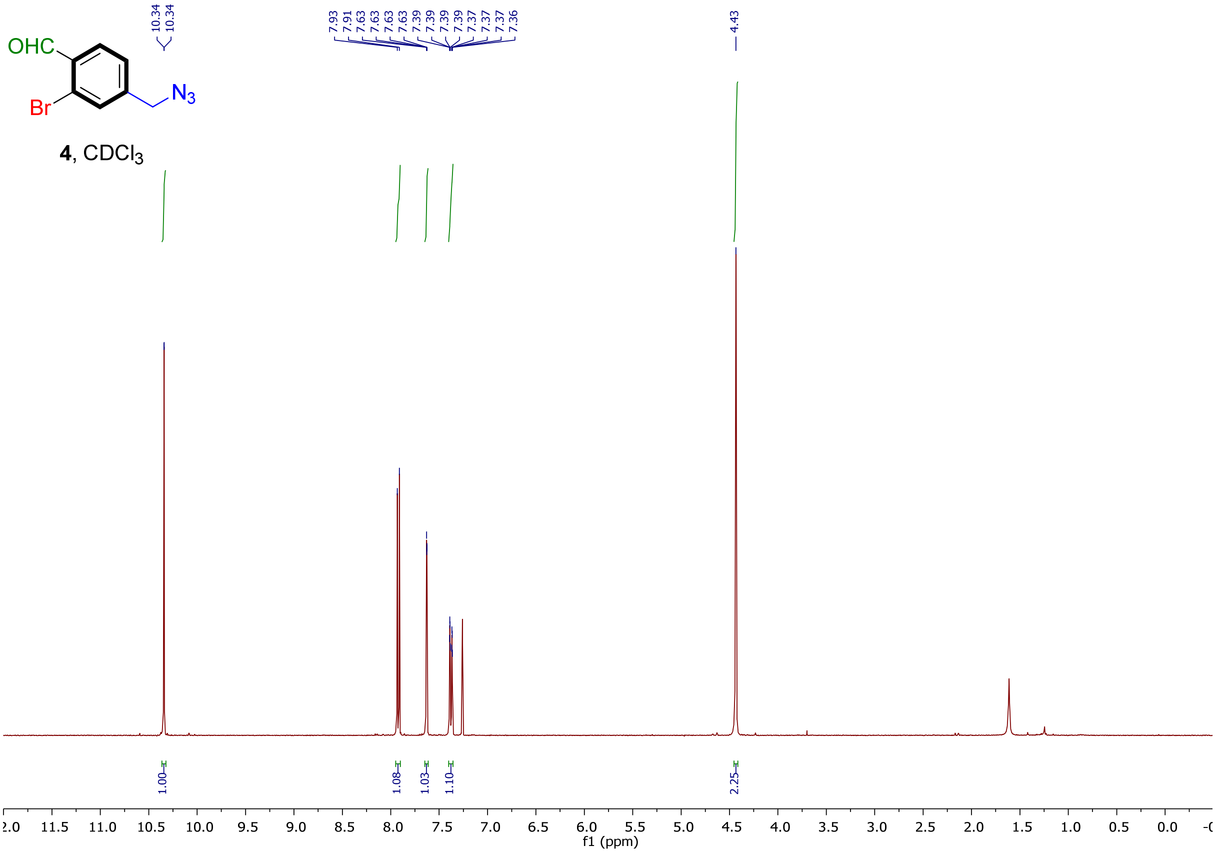
OHC
Br
N3
4, CDCl3
10.34
10.34
7.93
7.91
7.63
7.63
7.63
7.63
7.39
7.39
7.39
7.39
7.37
7.37
7.37
7.36
4.43
1.00
1.08
1.03
1.10
2.25
f1 (ppm)

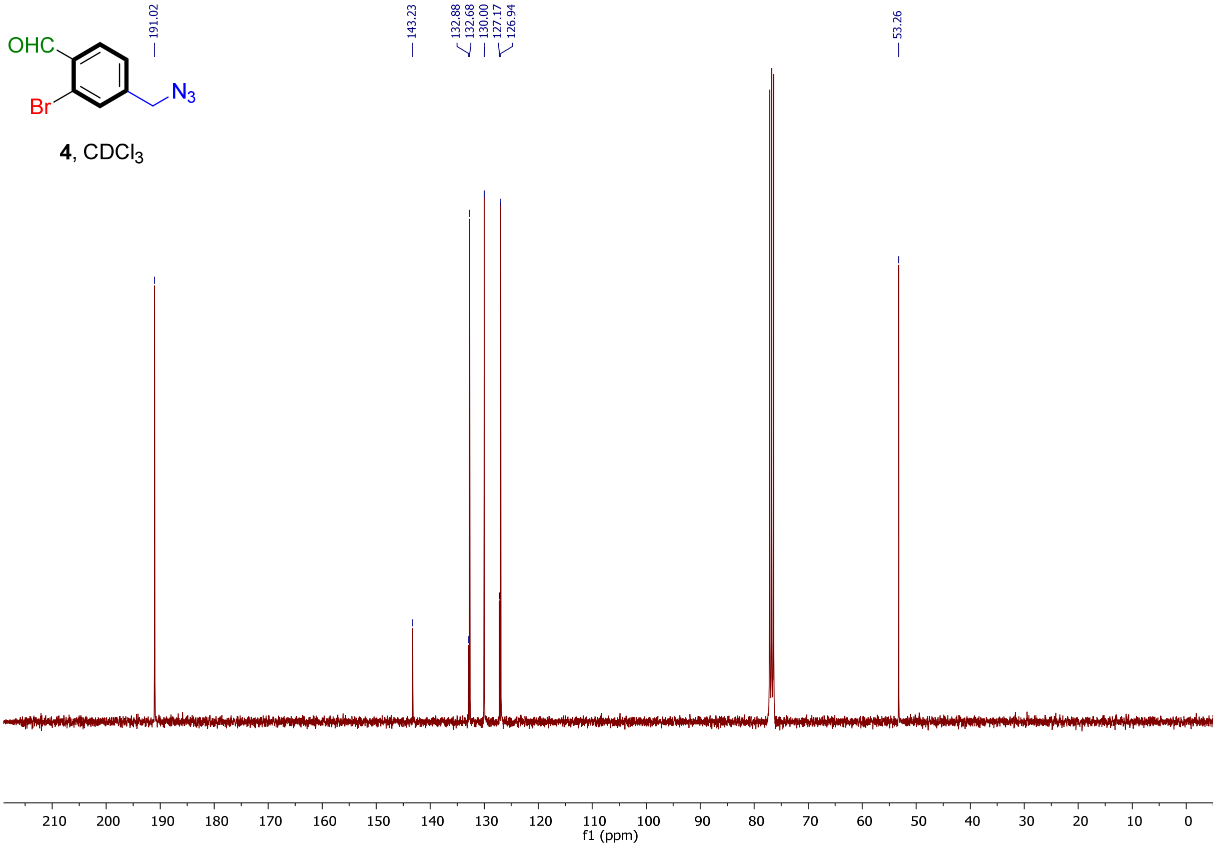
OHC
Br
N3
4, CDCl3
191.02
143.23
132.88
132.68
130.00
127.17
126.94
53.26
f1 (ppm)

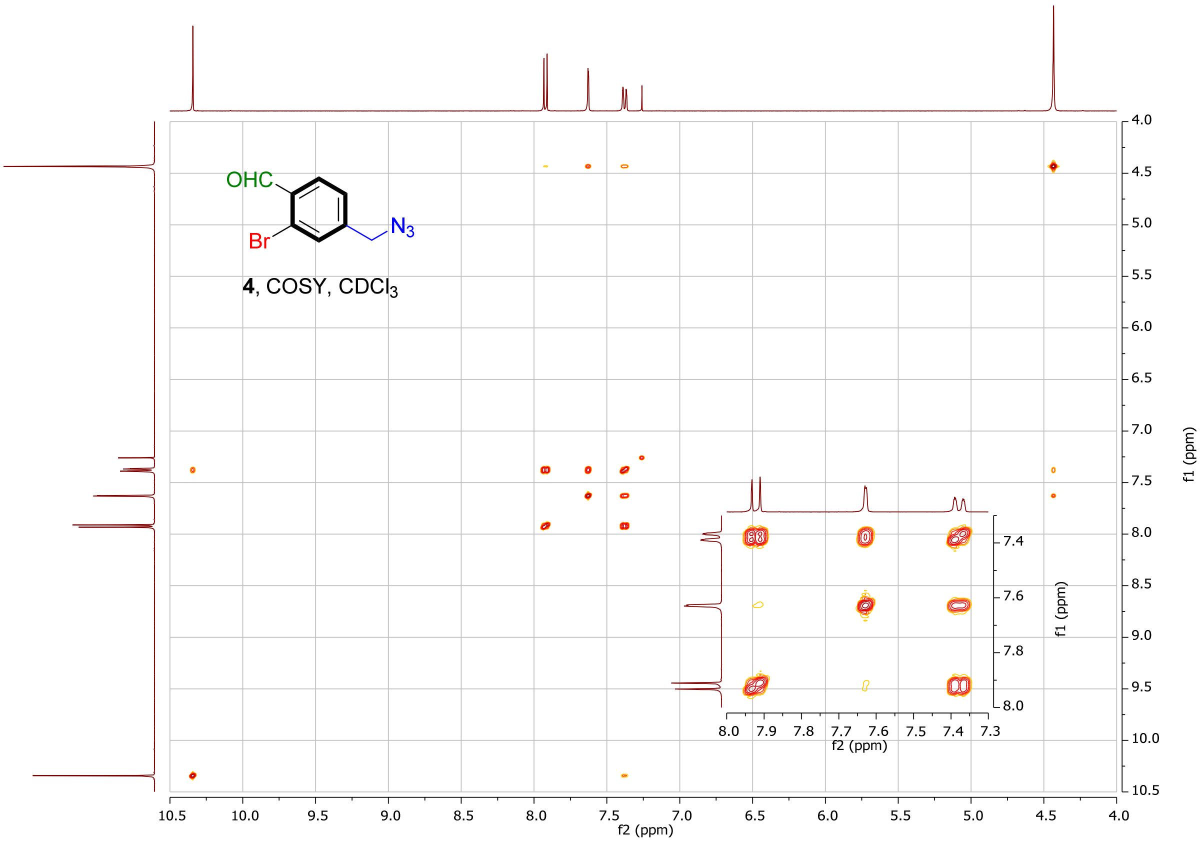
OHC
Br
N3
4, COSY, CDCl3
f2 (ppm)
f1 (ppm)

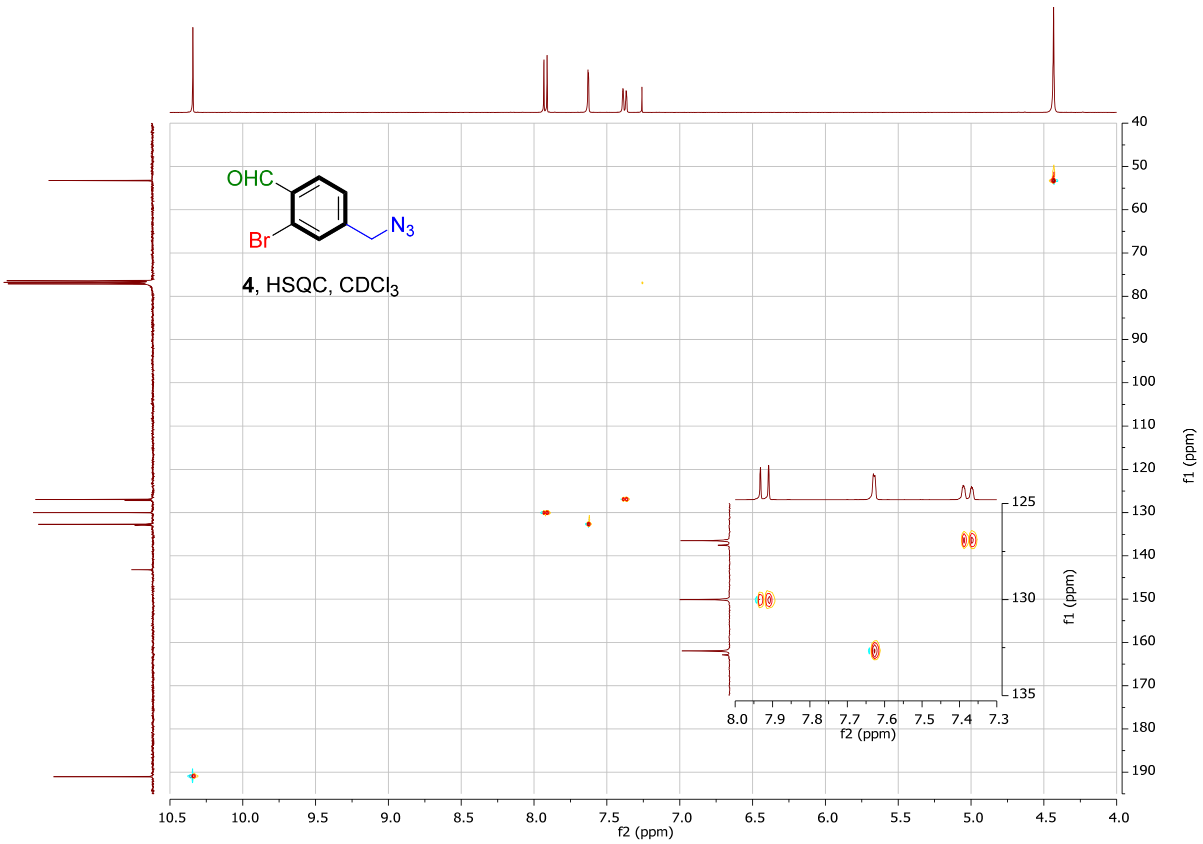
OHC
Br
N3
4, HSQC, CDCl3
f2 (ppm)
f1 (ppm)

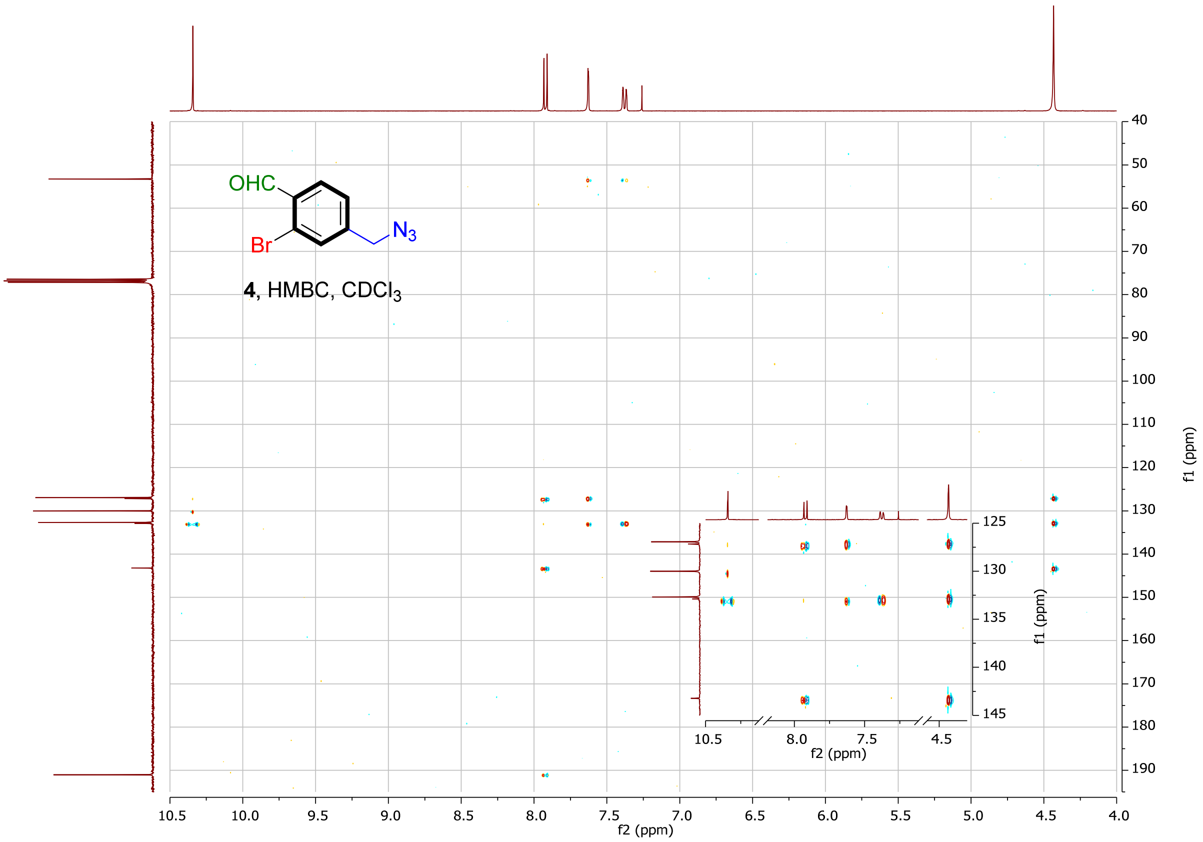
OHC
Br
N3
4, HMBC, CDCl3
f1 (ppm)
f2 (ppm)

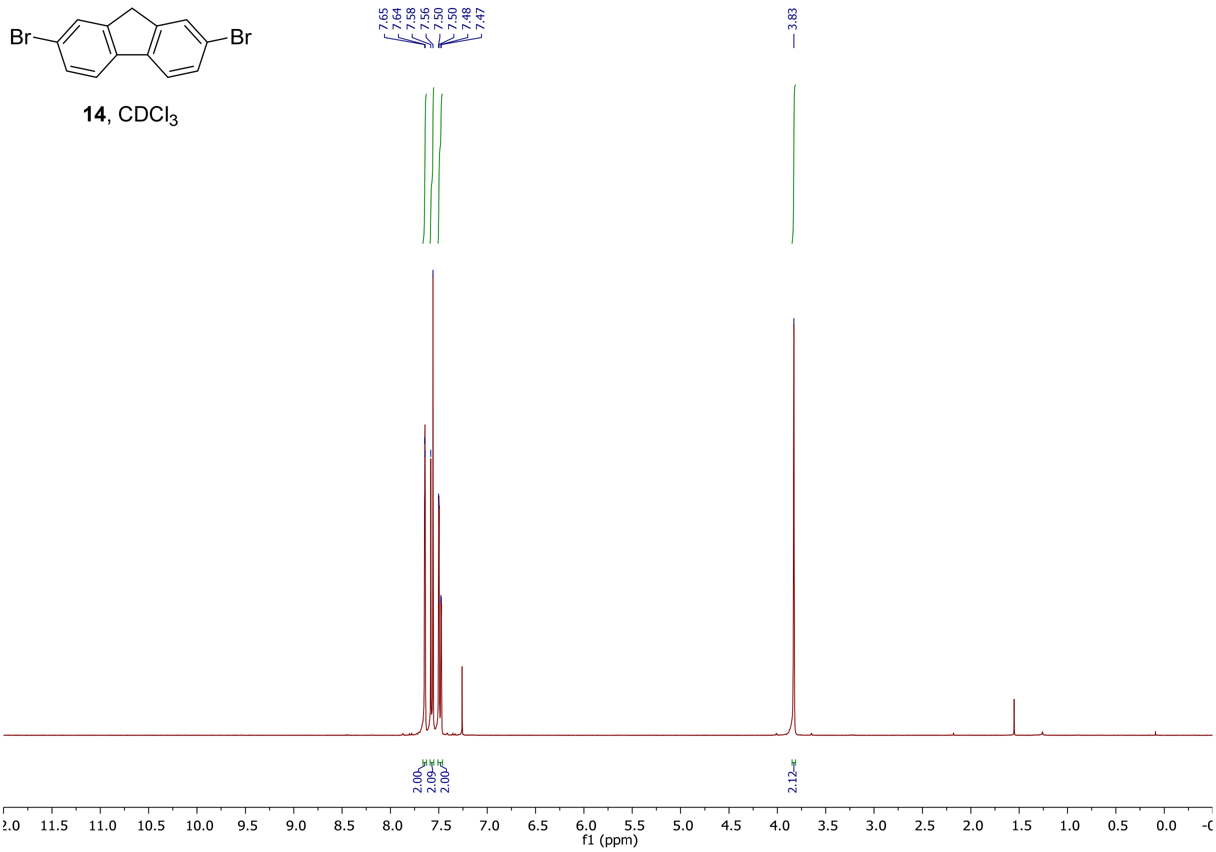

Br
Br
14, $CDCl_3$
7.65
7.64
7.58
7.56
7.50
7.50
7.48
7.47
3.83
2.00
2.09
2.00
2.12
f1 (ppm)

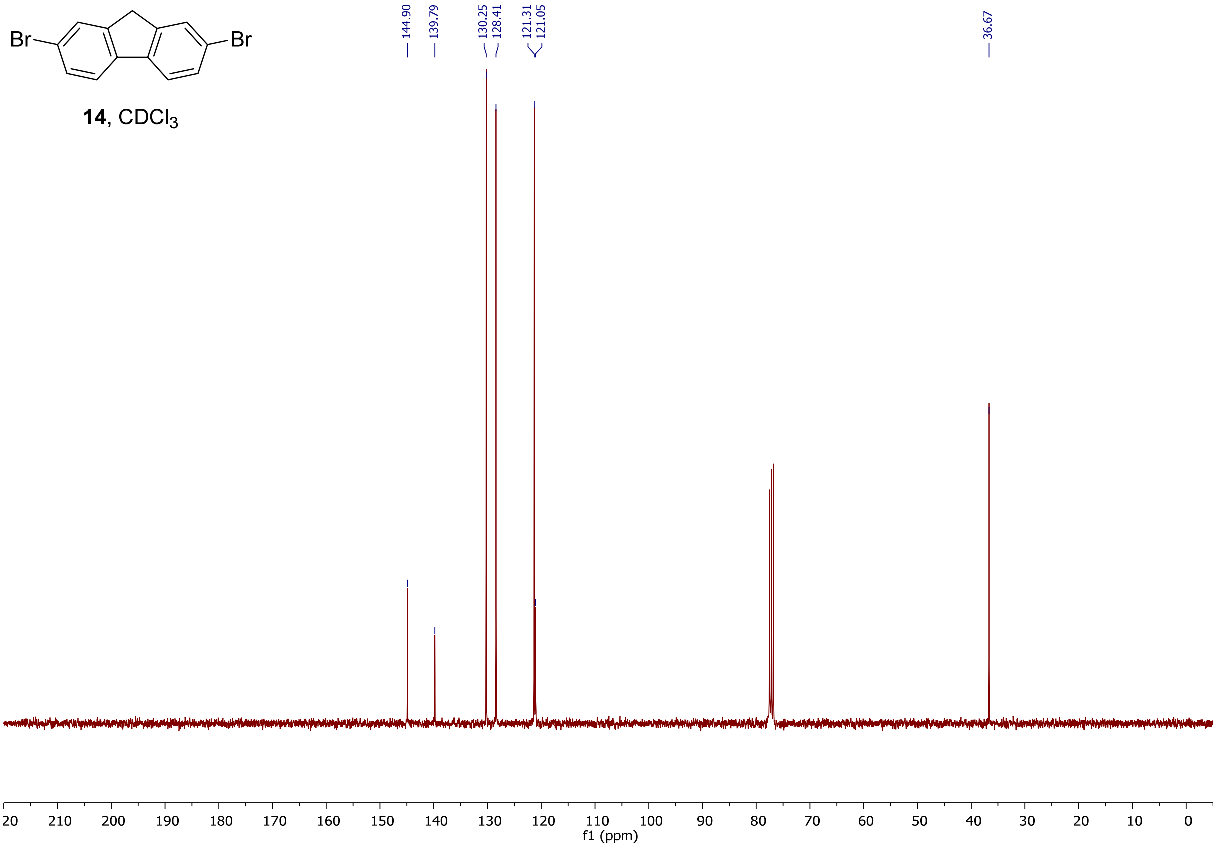

Br
Br
14, $CDCl_3$
144.90
139.79
130.25
128.41
121.31
121.05
36.67
f1 (ppm)

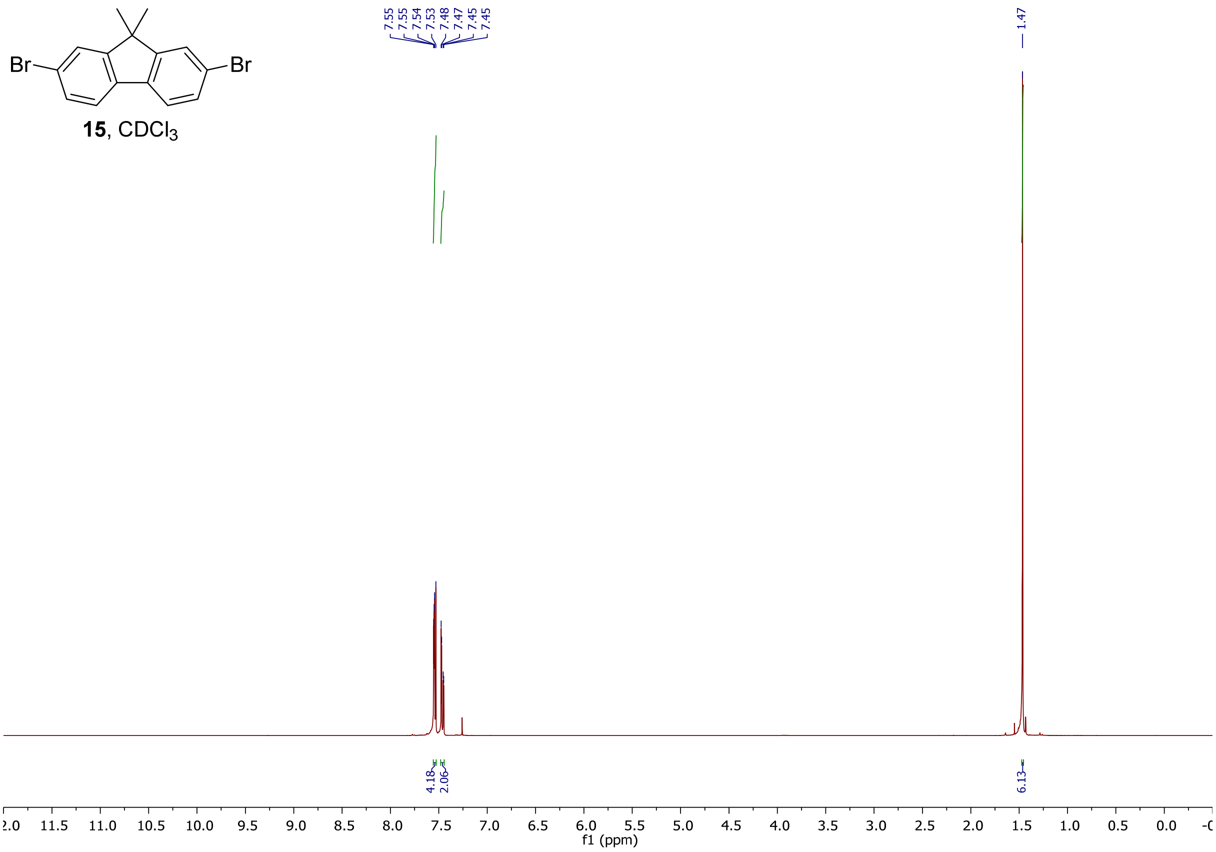
Br
Br
15, CDCl3
7.55
7.55
7.54
7.53
7.48
7.47
7.45
7.45
1.47
4.18
2.06
6.13
f1 (ppm)

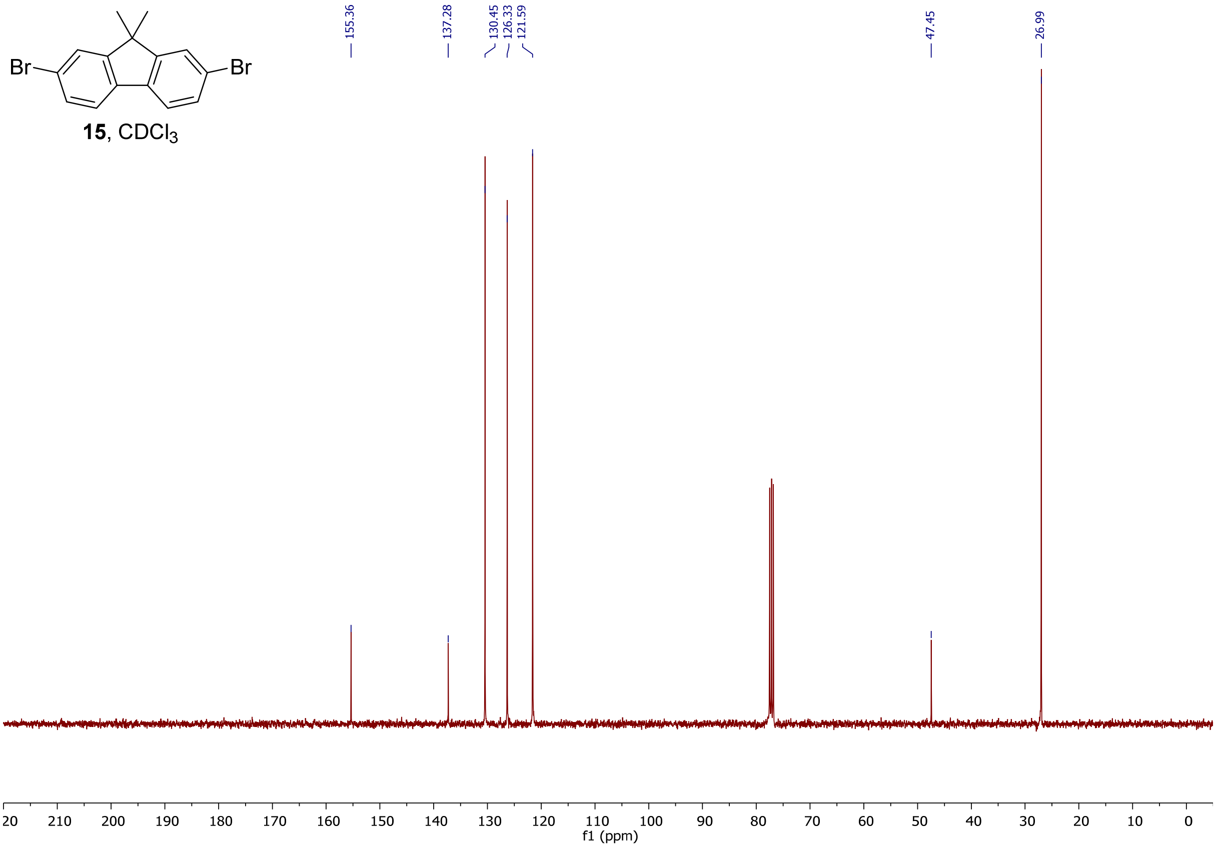
Br
Br
15, CDCl3
155.36
137.28
130.45
126.33
121.59
47.45
26.99
f1 (ppm)

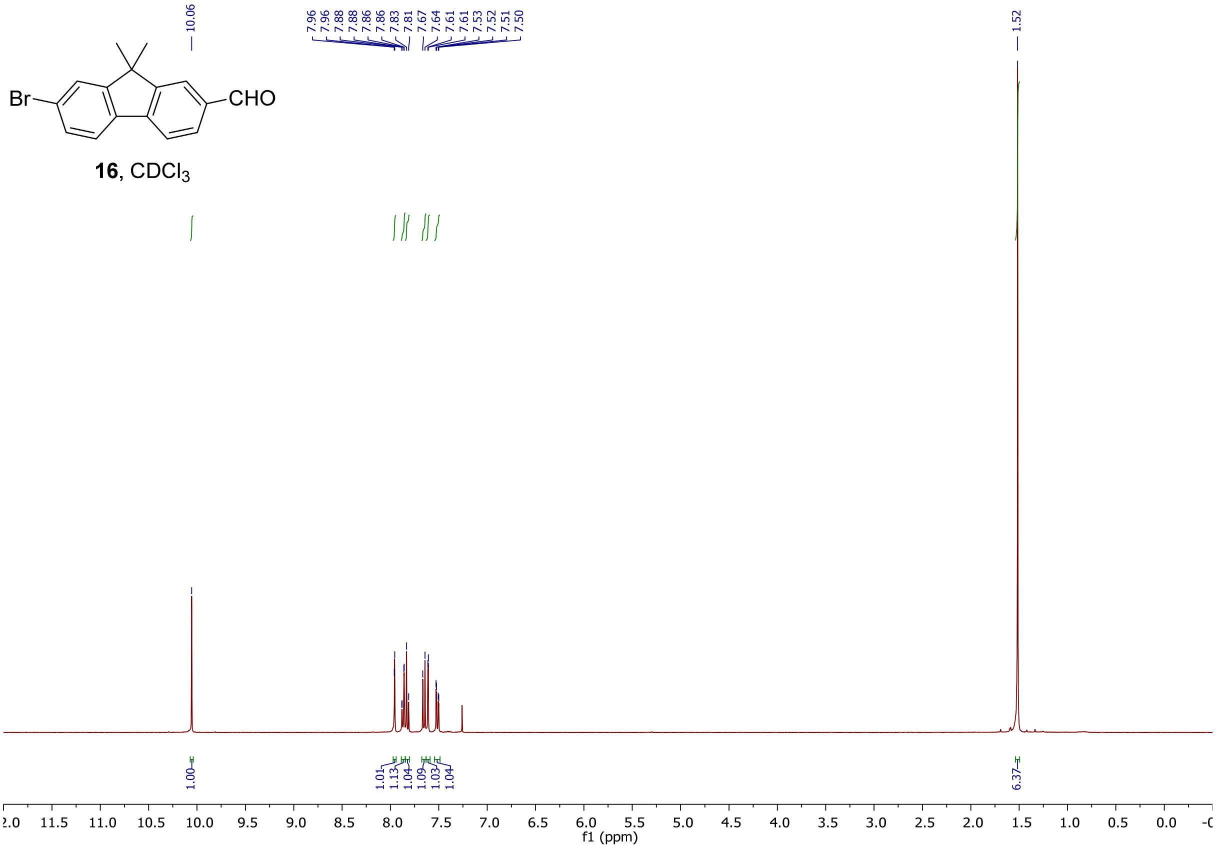
10.06
7.96 7.96 7.88 7.88 7.86 7.83 7.81 7.67 7.64 7.61 7.61 7.53 7.52 7.51 7.50
1.52
Br
CHO
16, CDCl3
1.00
1.01 1.13 1.04 1.09 1.03 1.04
6.37
f1 (ppm)

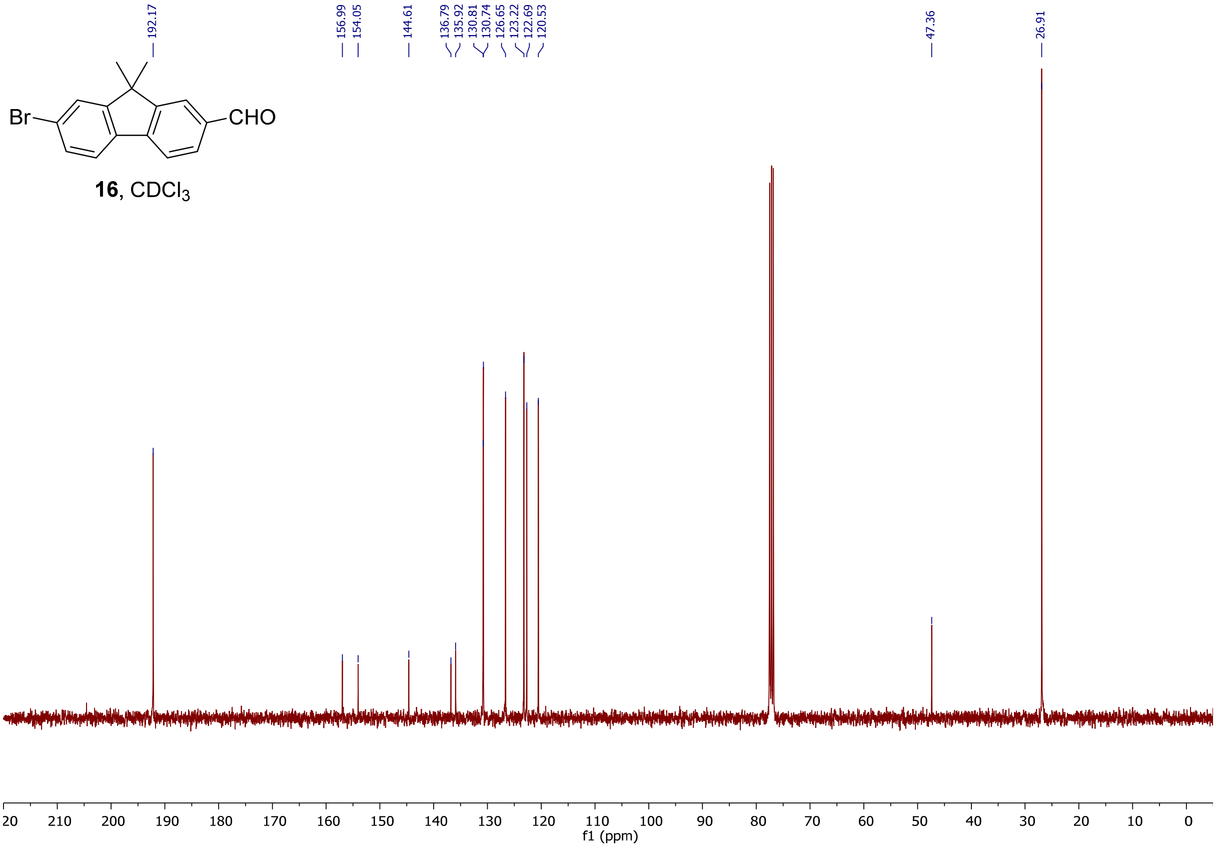
192.17
156.99 154.05
144.61
136.79 135.92 130.81 130.74 126.65 123.22 122.69 120.53
47.36
26.91
Br
CHO
16, CDCl3
f1 (ppm)

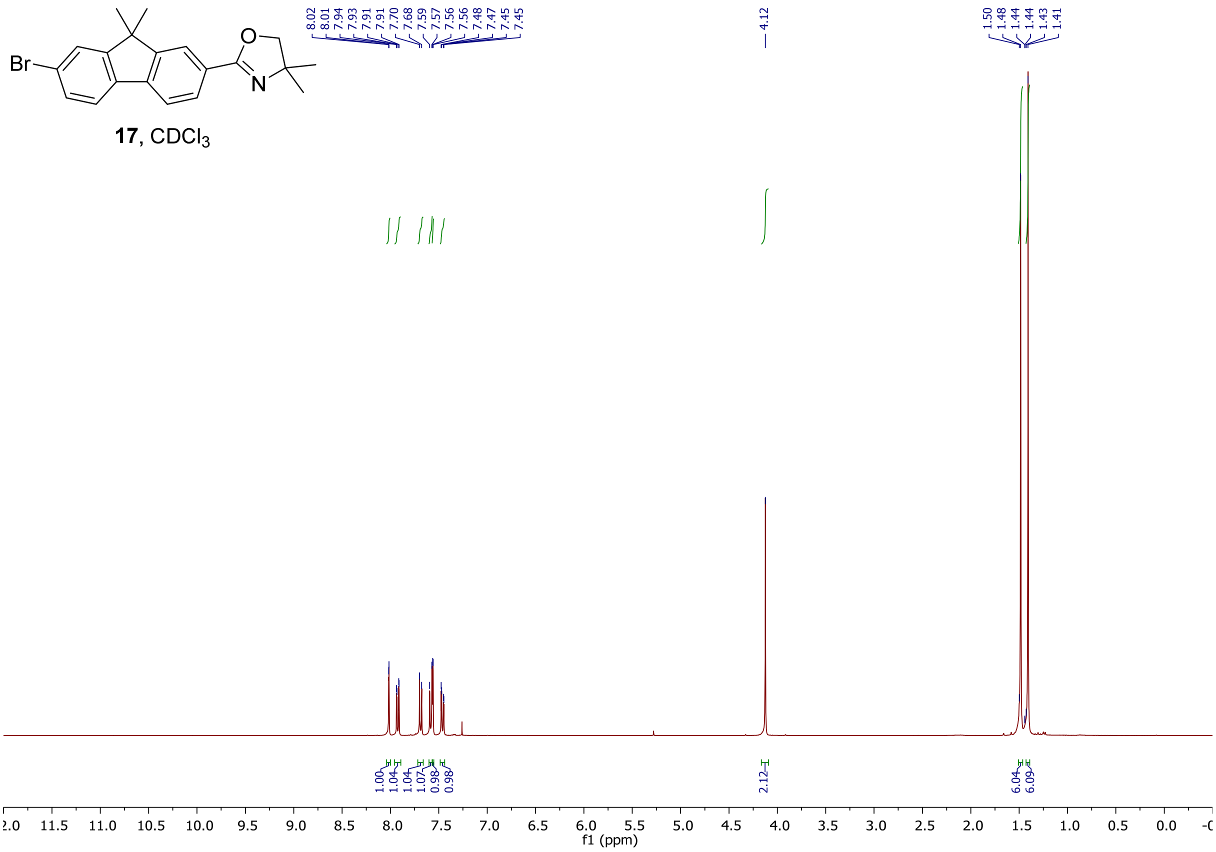
Br
O
N
17, $CDCl_3$
8.02
8.01
7.94
7.93
7.91
7.91
7.70
7.68
7.59
7.57
7.56
7.56
7.48
7.47
7.45
7.45
4.12
1.50
1.48
1.44
1.44
1.43
1.41
1.00
1.04
1.04
1.07
0.98
0.98
2.12
6.04
6.09
f1 (ppm)

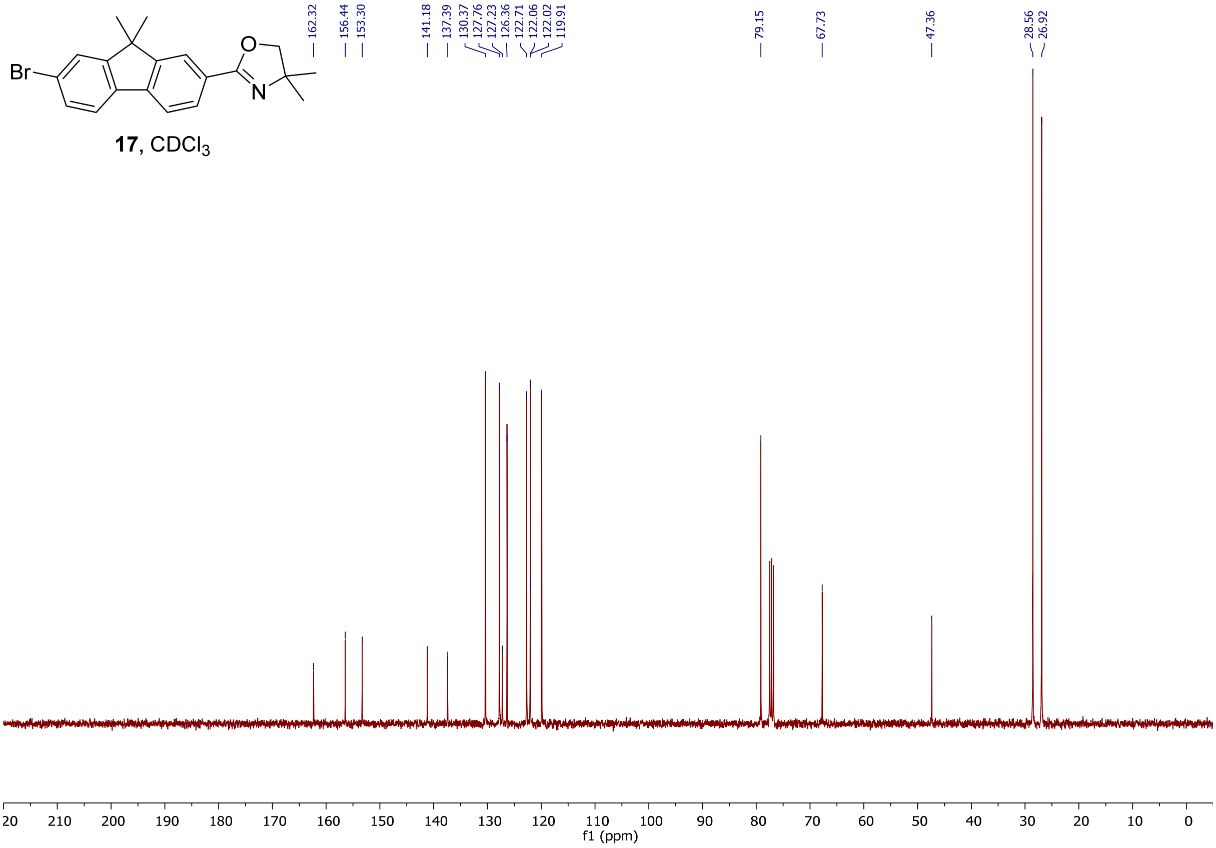
Br
O
N
17, $CDCl_3$
162.32
156.44
153.30
141.18
137.39
130.37
127.76
127.23
126.36
122.71
122.06
122.02
119.91
79.15
67.73
47.36
28.56
26.92
f1 (ppm)

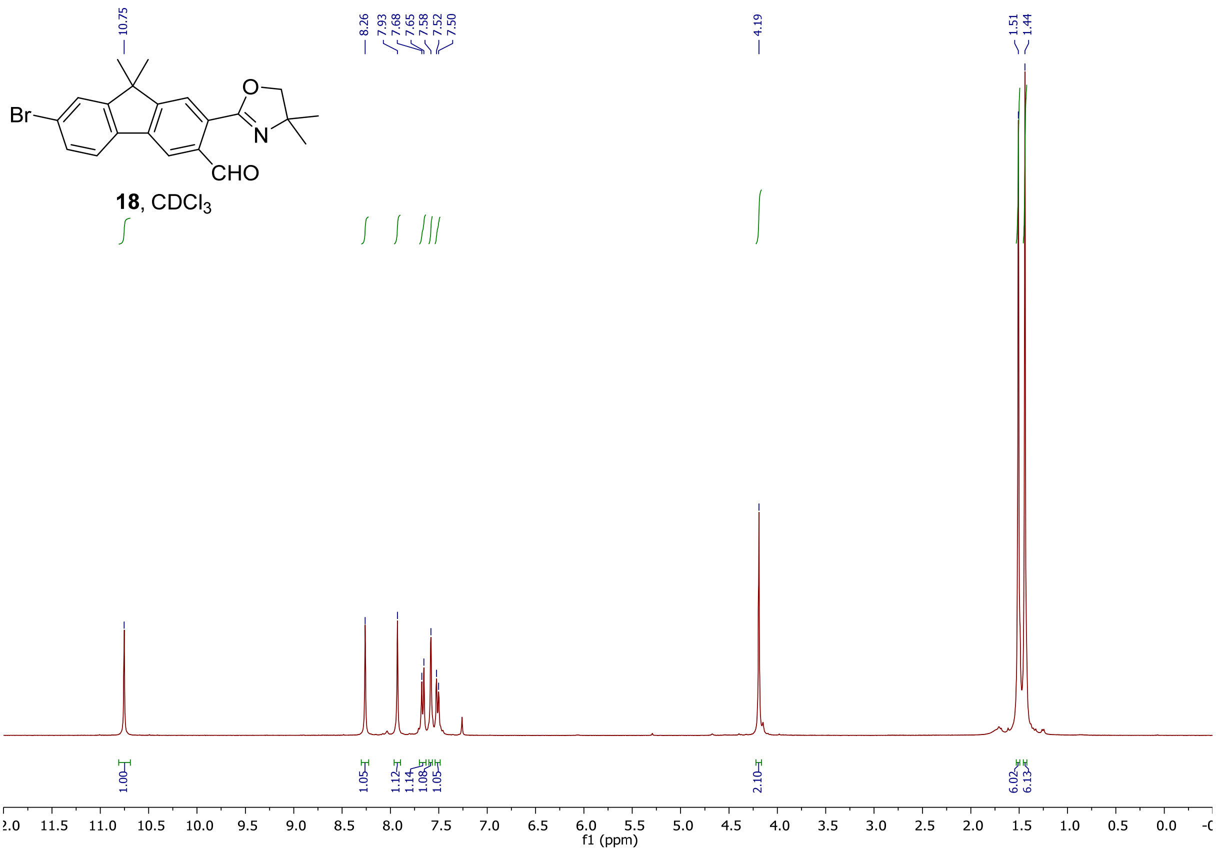
10.75
8.26
7.93
7.68
7.65
7.58
7.52
7.50
4.19
1.51
1.44
Br
O
N
CHO
18, CDCl3
1.00
1.05
1.12
1.14
1.08
1.05
2.10
6.02
6.13
f1 (ppm)

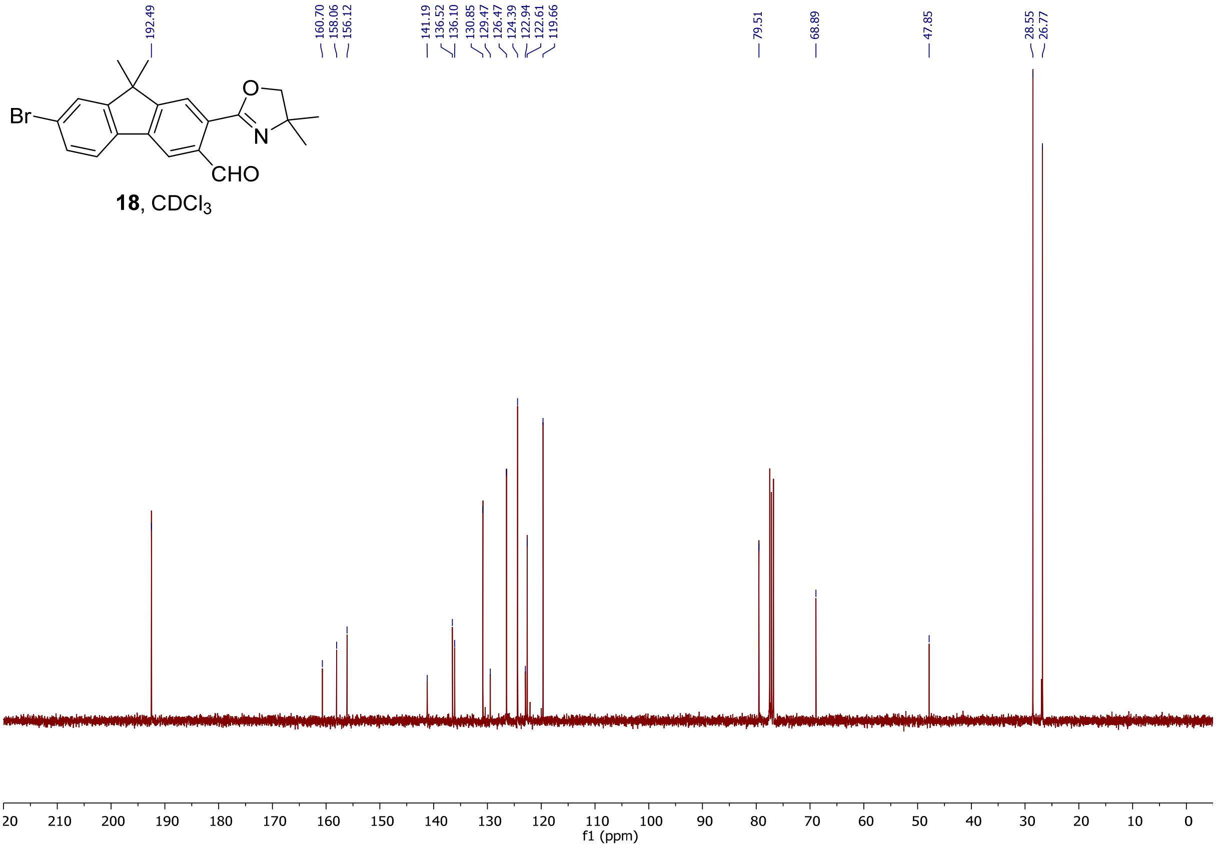
192.49
160.70
158.06
156.12
141.19
136.52
136.10
130.85
129.47
126.47
124.39
122.94
122.61
119.66
79.51
68.89
47.85
28.55
26.77
Br
O
N
CHO
18, CDCl3
f1 (ppm)

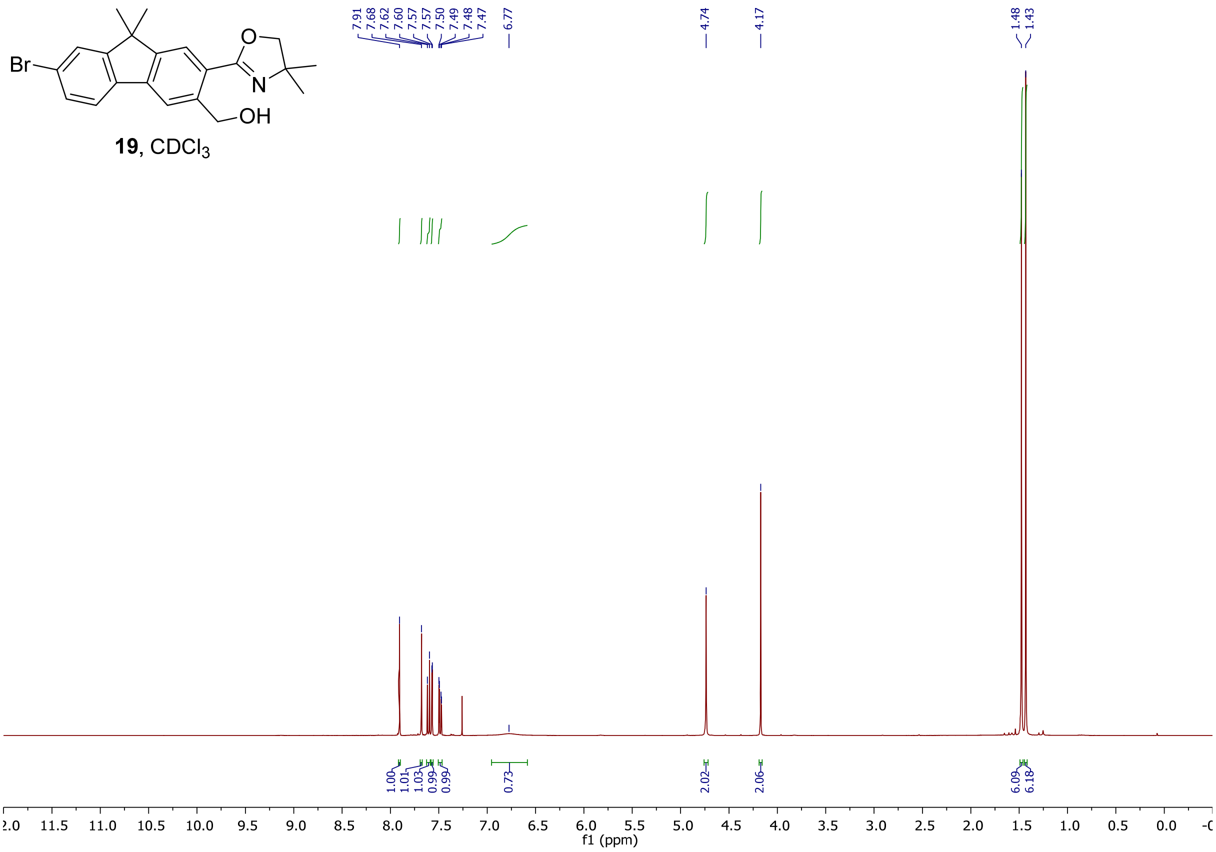

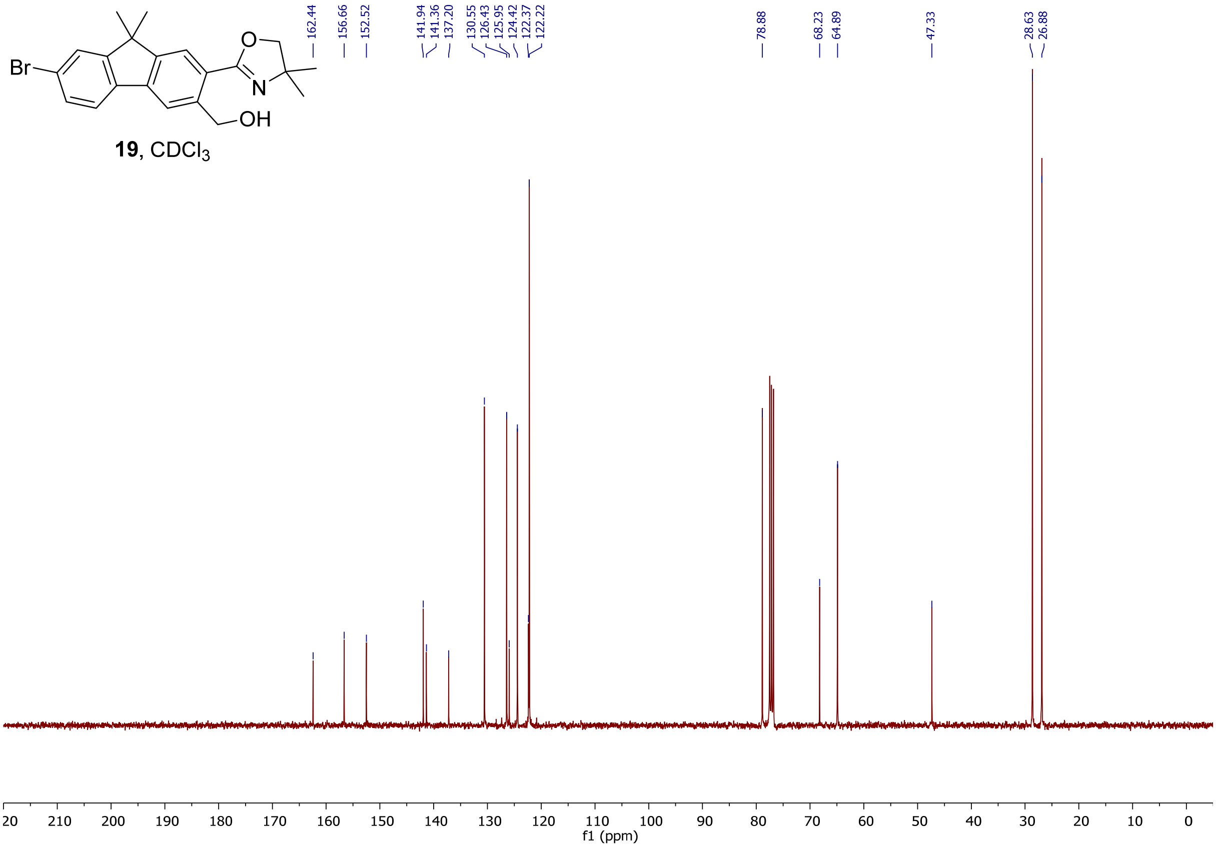

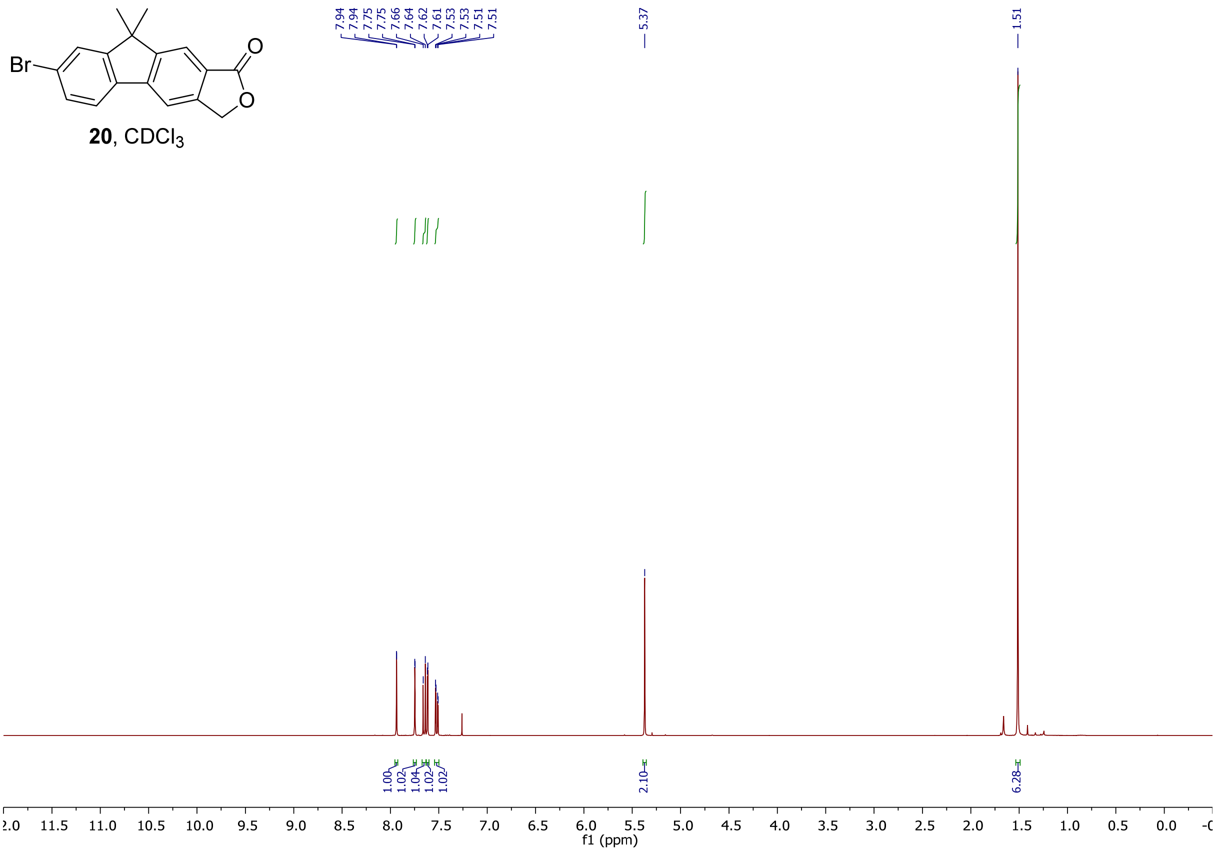
20, CDCl3
7.94
7.94
7.75
7.75
7.66
7.64
7.62
7.61
7.53
7.53
7.51
7.51
5.37
1.51
1.00
1.02
1.04
1.02
1.02
2.10
6.28
f1 (ppm)

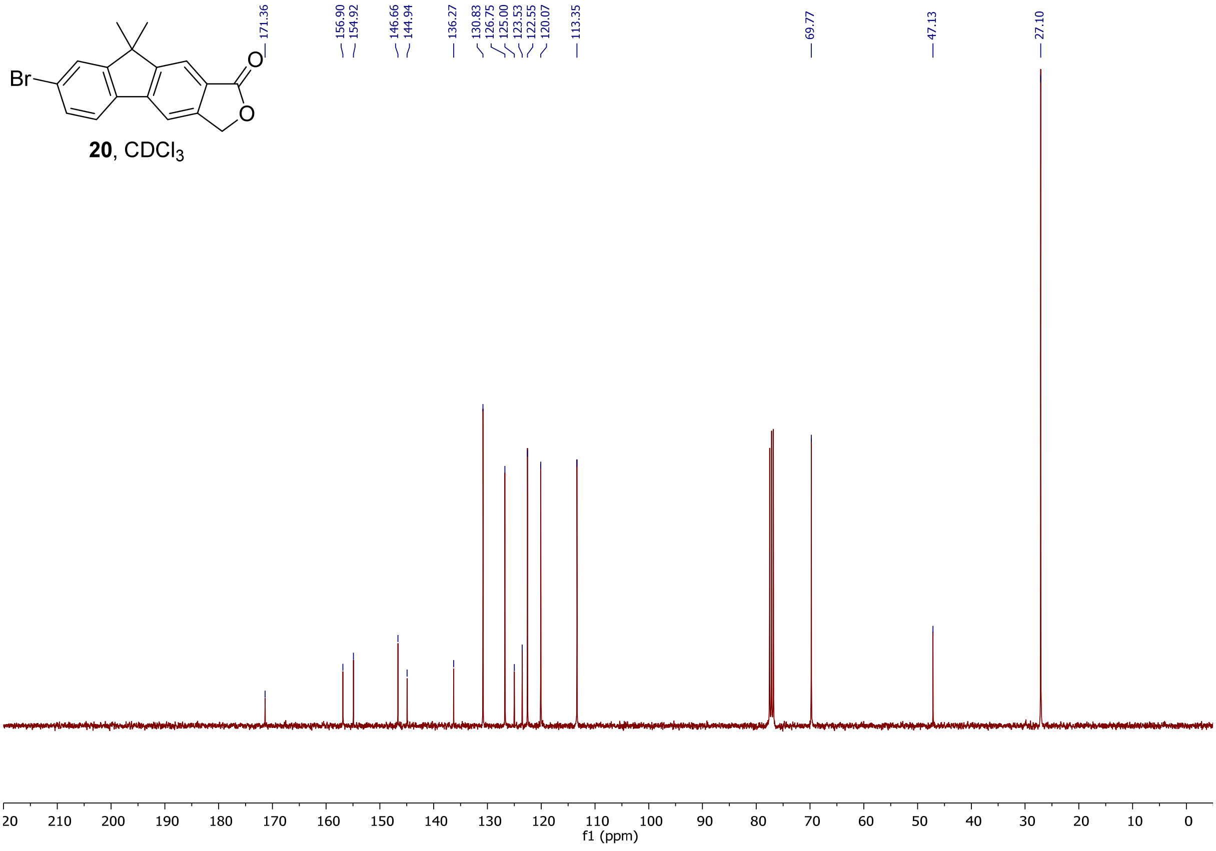
20, CDCl3
171.36
156.90
154.92
146.66
144.94
136.27
130.83
126.75
125.00
123.53
122.55
120.07
113.35
69.77
47.13
27.10
f1 (ppm)

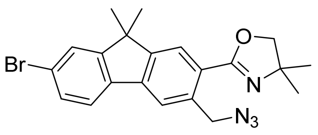

21, $CDCl_3$

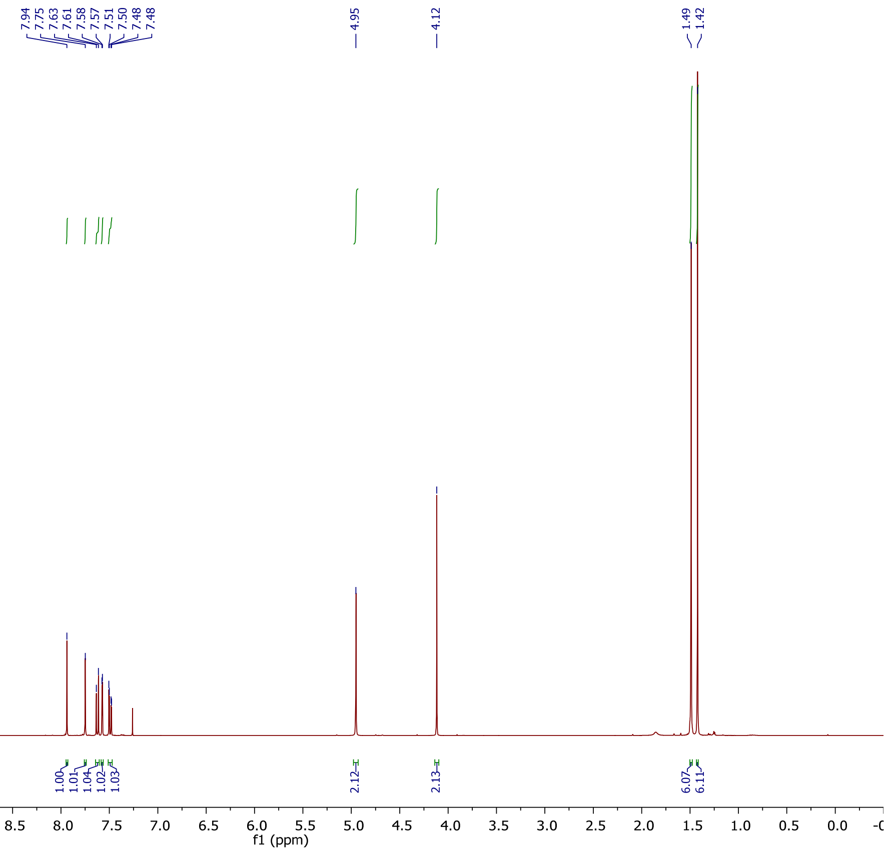

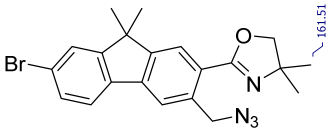

21, $CDCl_3$

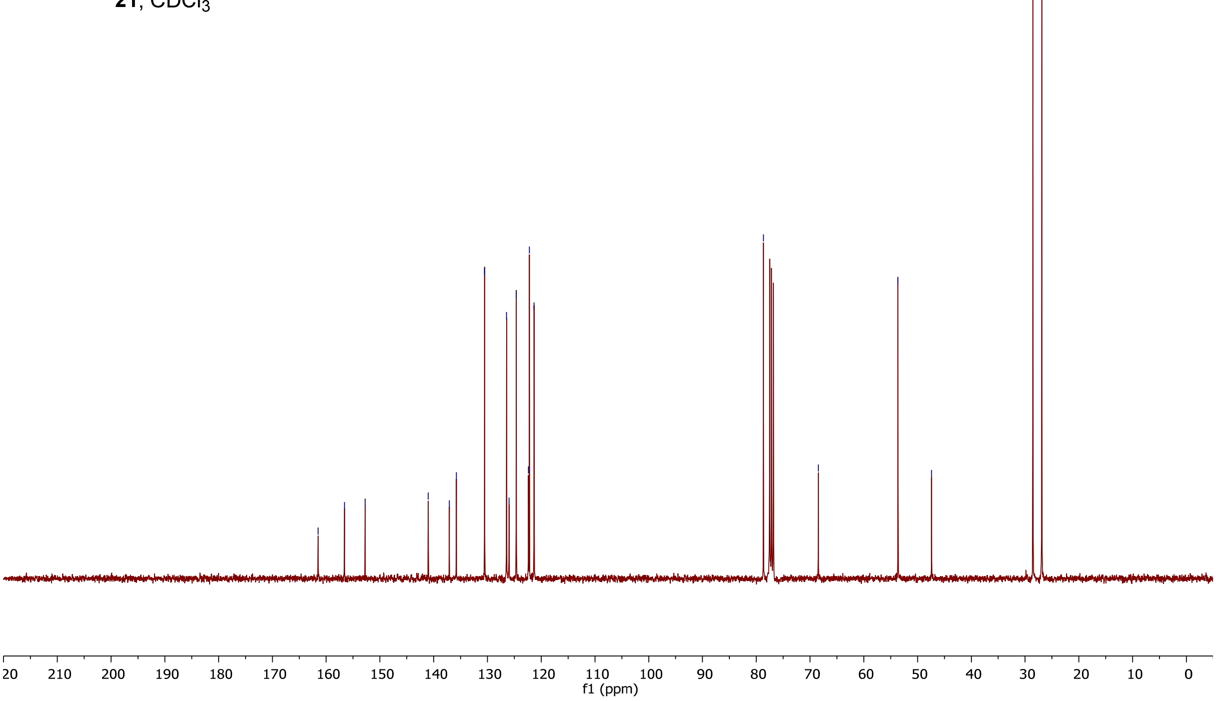

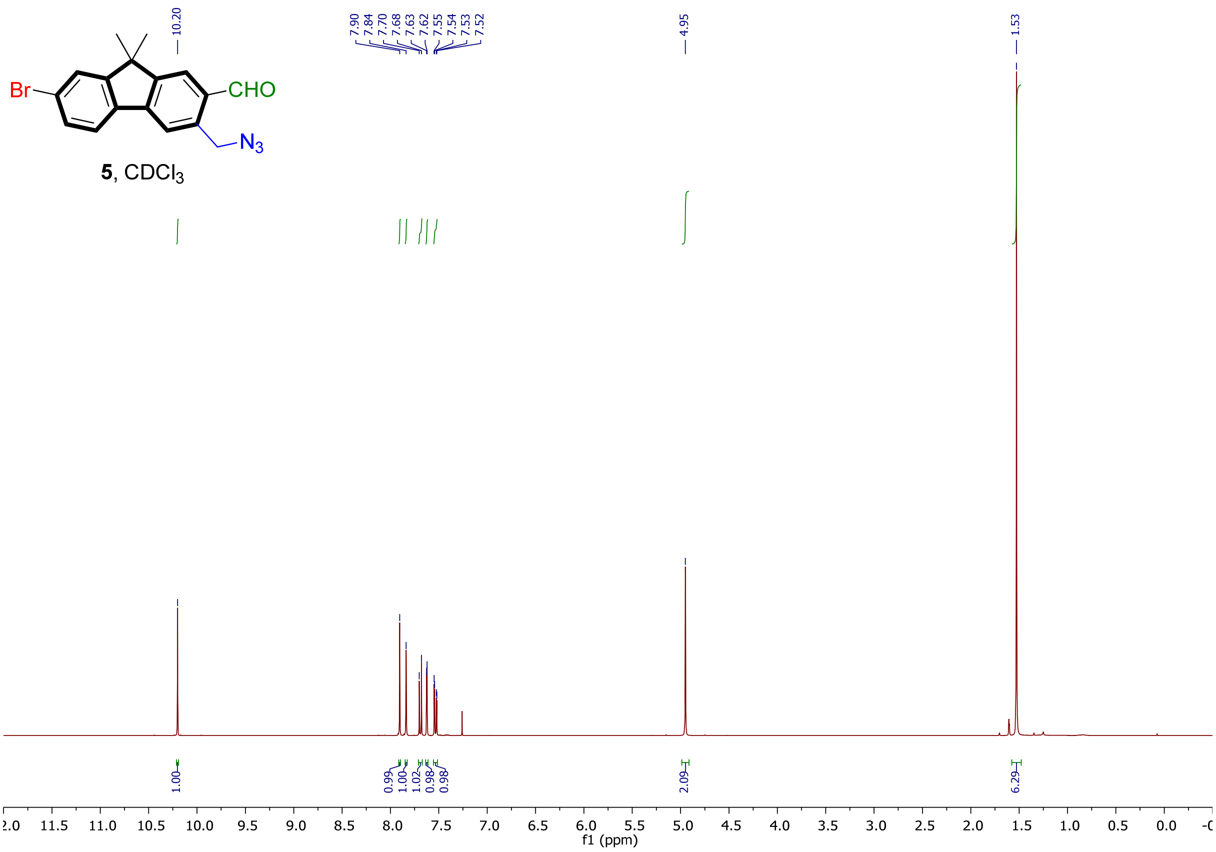

10.20
7.90
7.84
7.70
7.68
7.63
7.62
7.55
7.54
7.53
7.52
4.95
1.53
Br
CHO
N3
5, CDCl3
1.00
0.99
1.00
1.02
0.98
0.98
2.09
6.29
f1 (ppm)

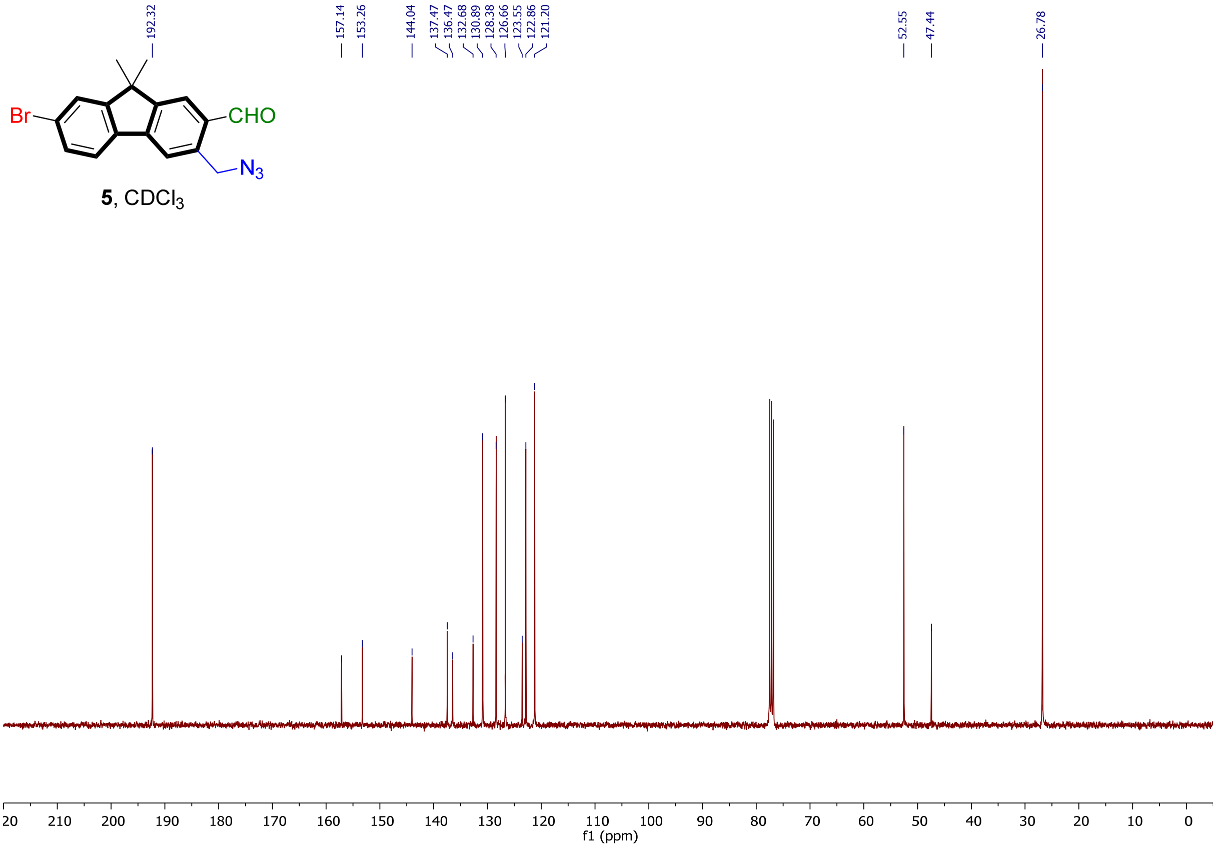

192.32
157.14
153.26
144.04
137.47
136.47
132.68
130.89
128.38
126.66
123.55
122.86
121.20
52.55
47.44
26.78
Br
CHO
N3
5, CDCl3
f1 (ppm)

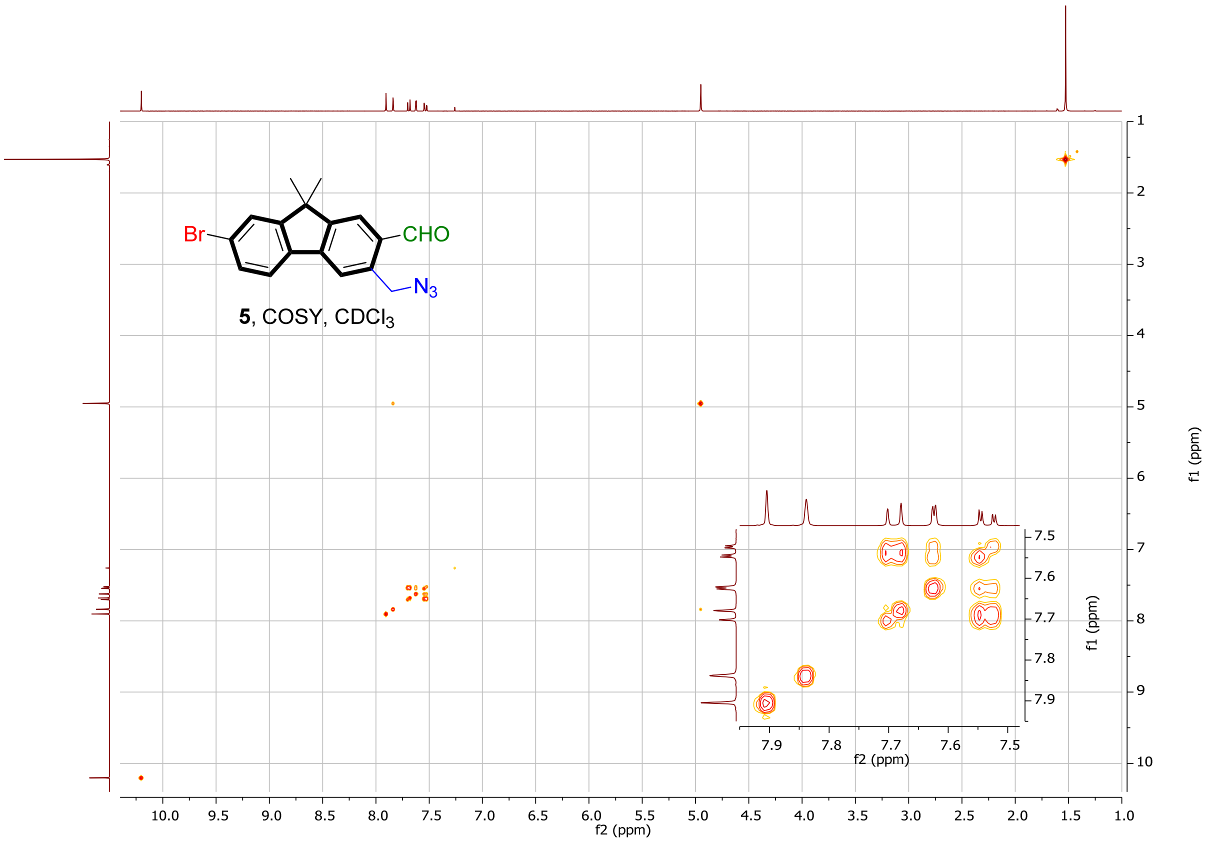
Br
CHO
N3
5, COSY, CDCl3
f2 (ppm)
f1 (ppm)

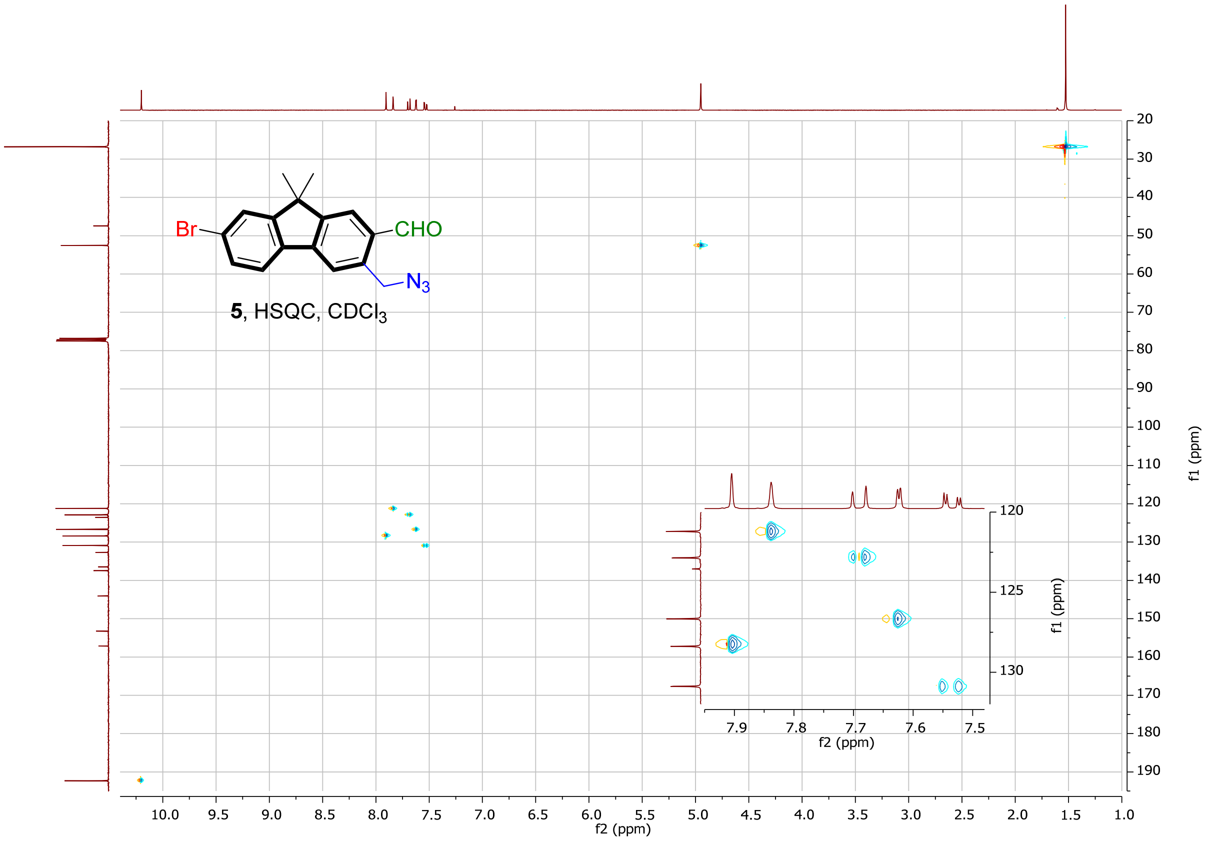
Br
CHO
N3
5, HSQC, CDCl3
f2 (ppm)
f1 (ppm)

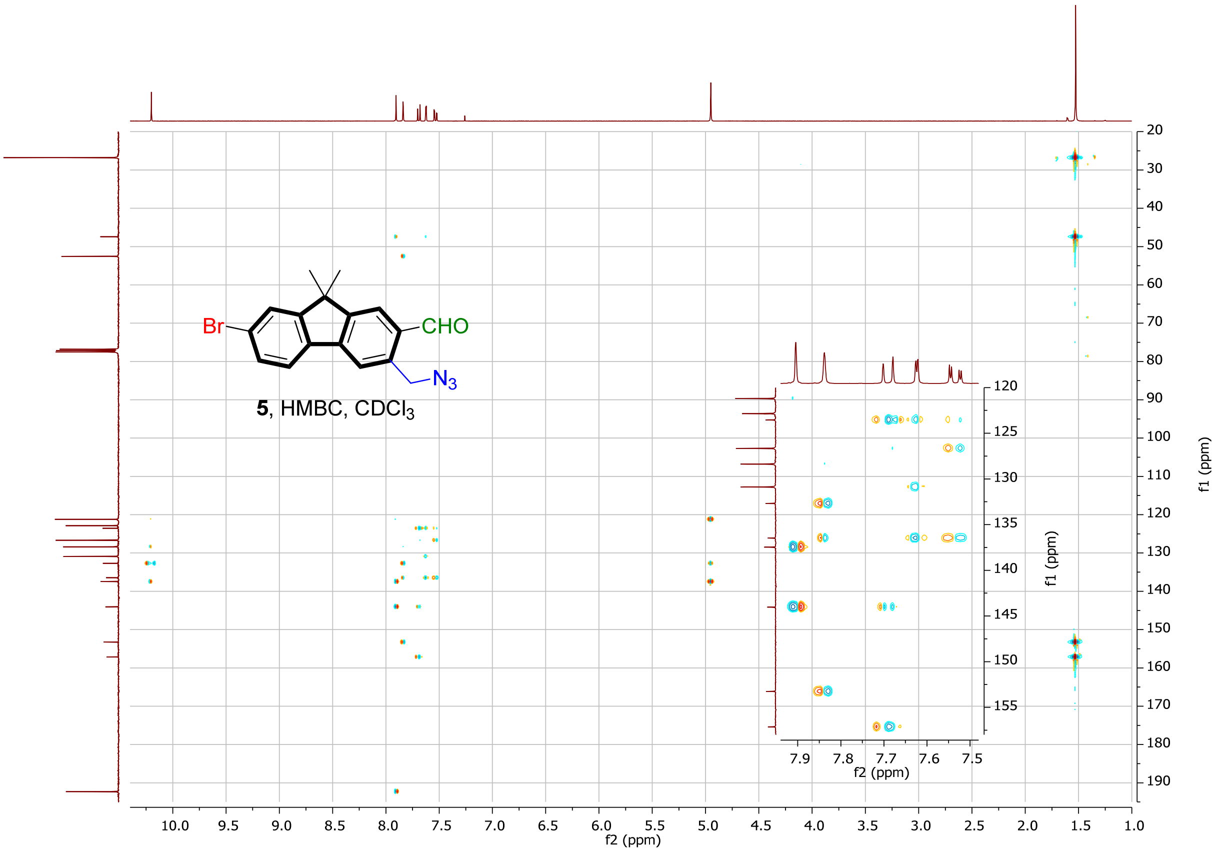
Br
CHO
N3
5, HMBC, CDCl3
f2 (ppm)
f1 (ppm)

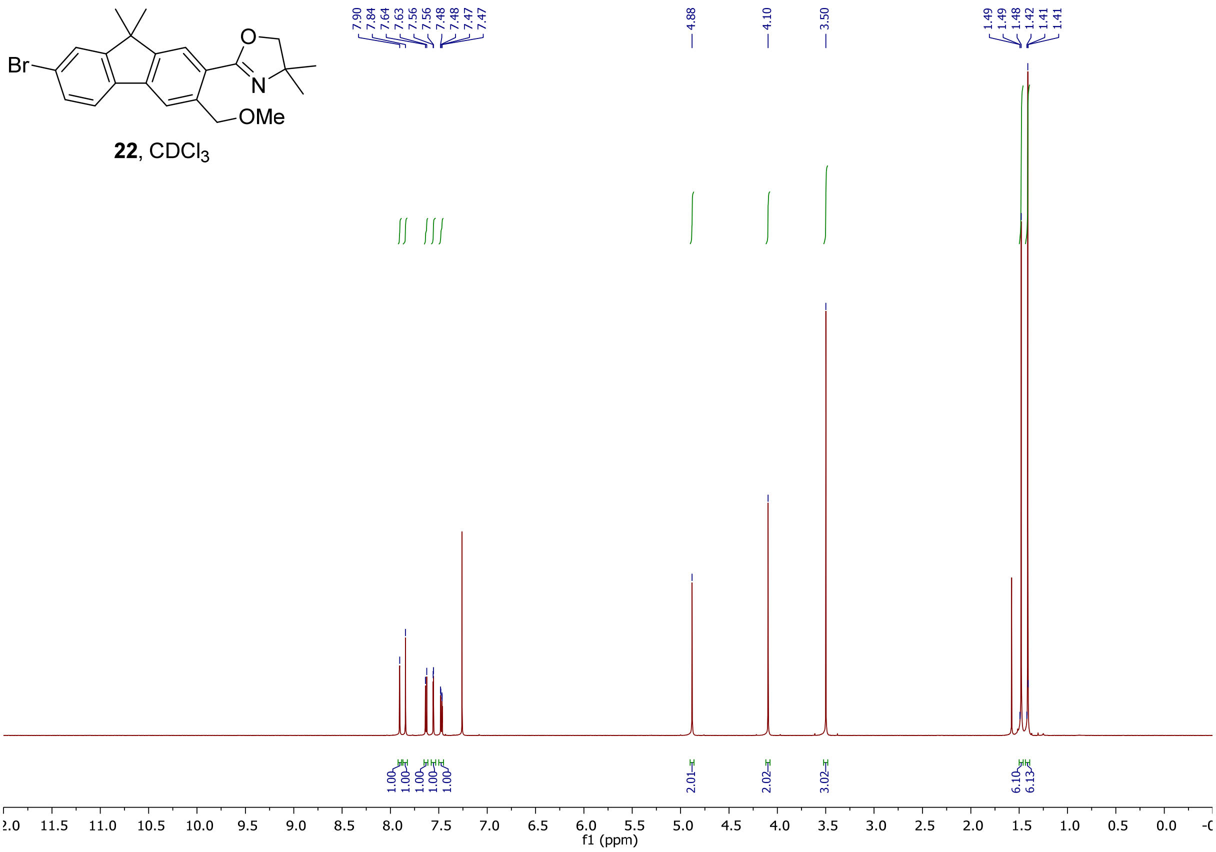
Br
O
N
OMe
22, CDCl3
7.90
7.84
7.64
7.63
7.56
7.48
7.48
7.47
7.47
4.88
4.10
3.50
1.49
1.49
1.48
1.42
1.41
1.41
1.00
1.00
1.00
1.00
1.00
2.01
2.02
3.02
6.10
6.13
f1 (ppm)

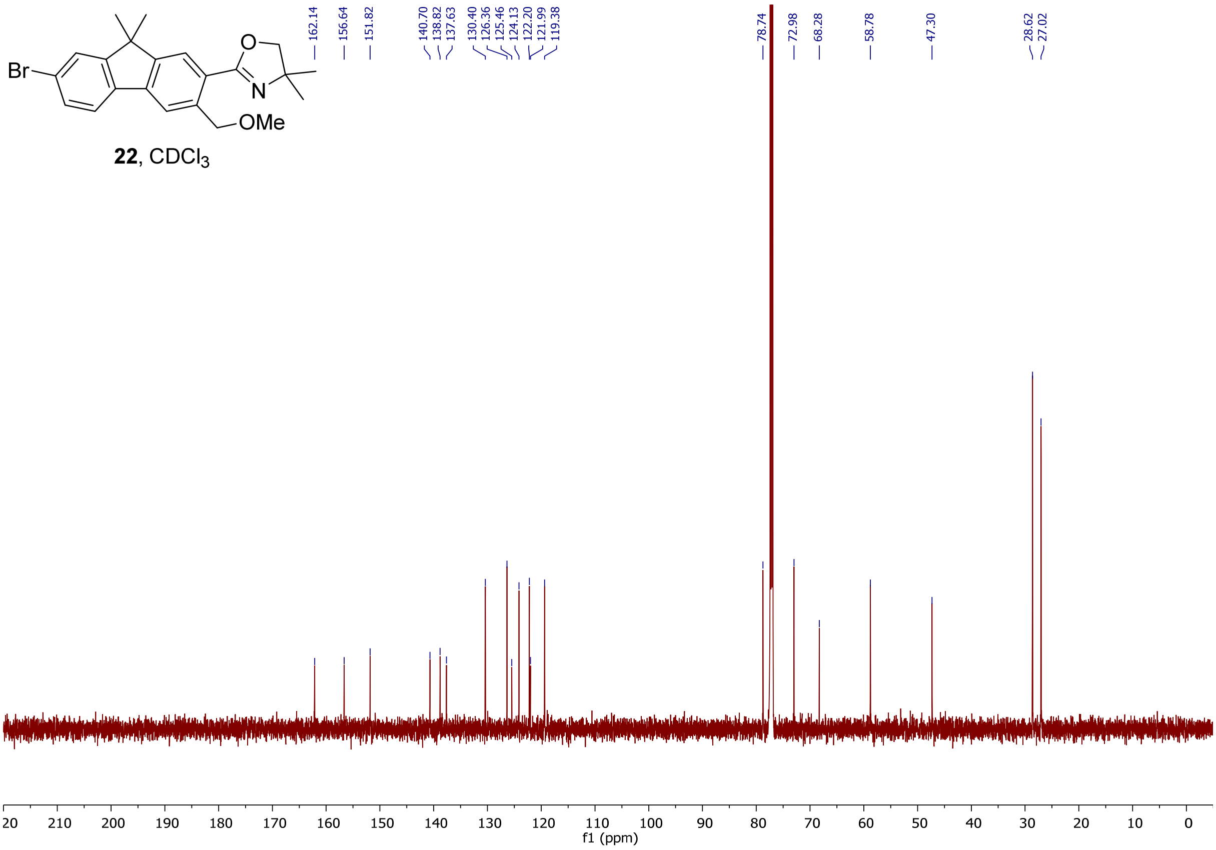
Br
O
N
OMe
22, CDCl3
162.14
156.64
151.82
140.70
138.82
137.63
130.40
126.36
125.46
124.13
122.20
121.99
119.38
78.74
72.98
68.28
58.78
47.30
28.62
27.02
f1 (ppm)

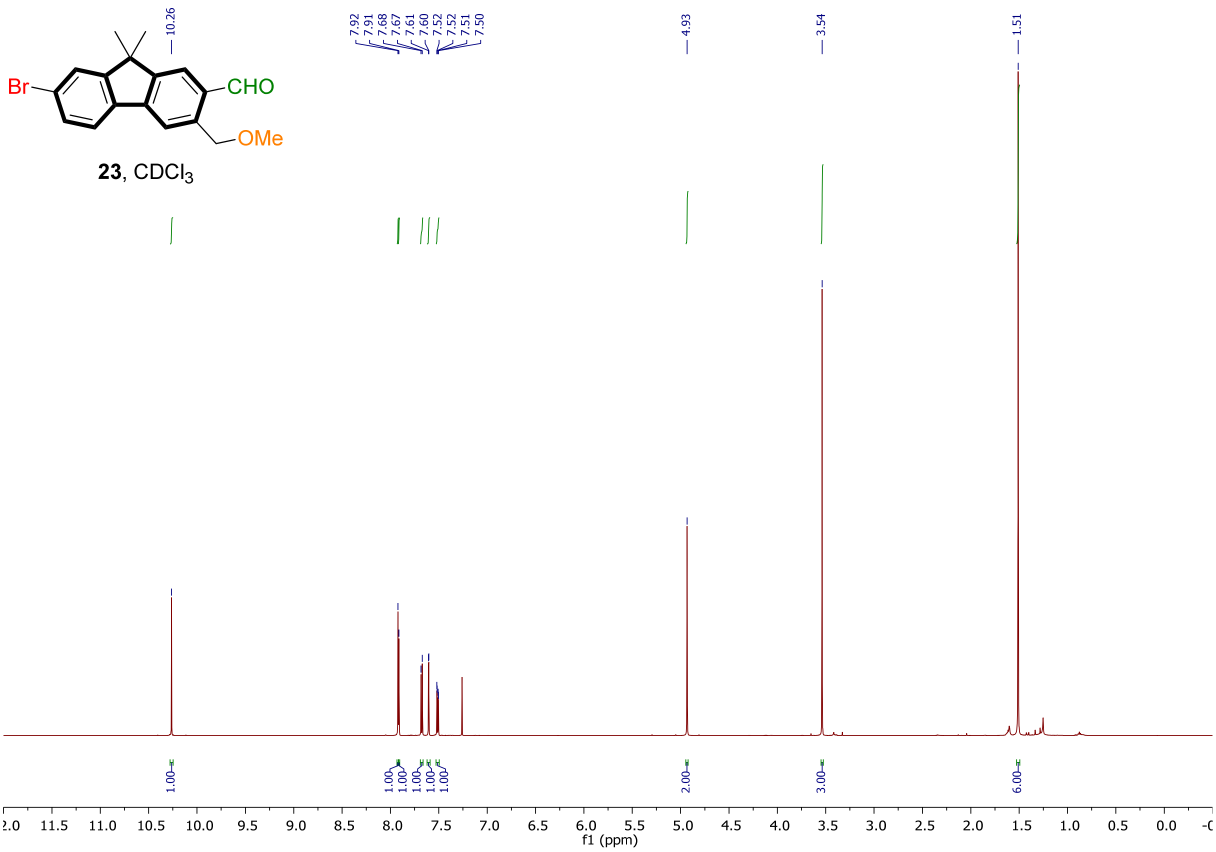

23, $CDCl_3$

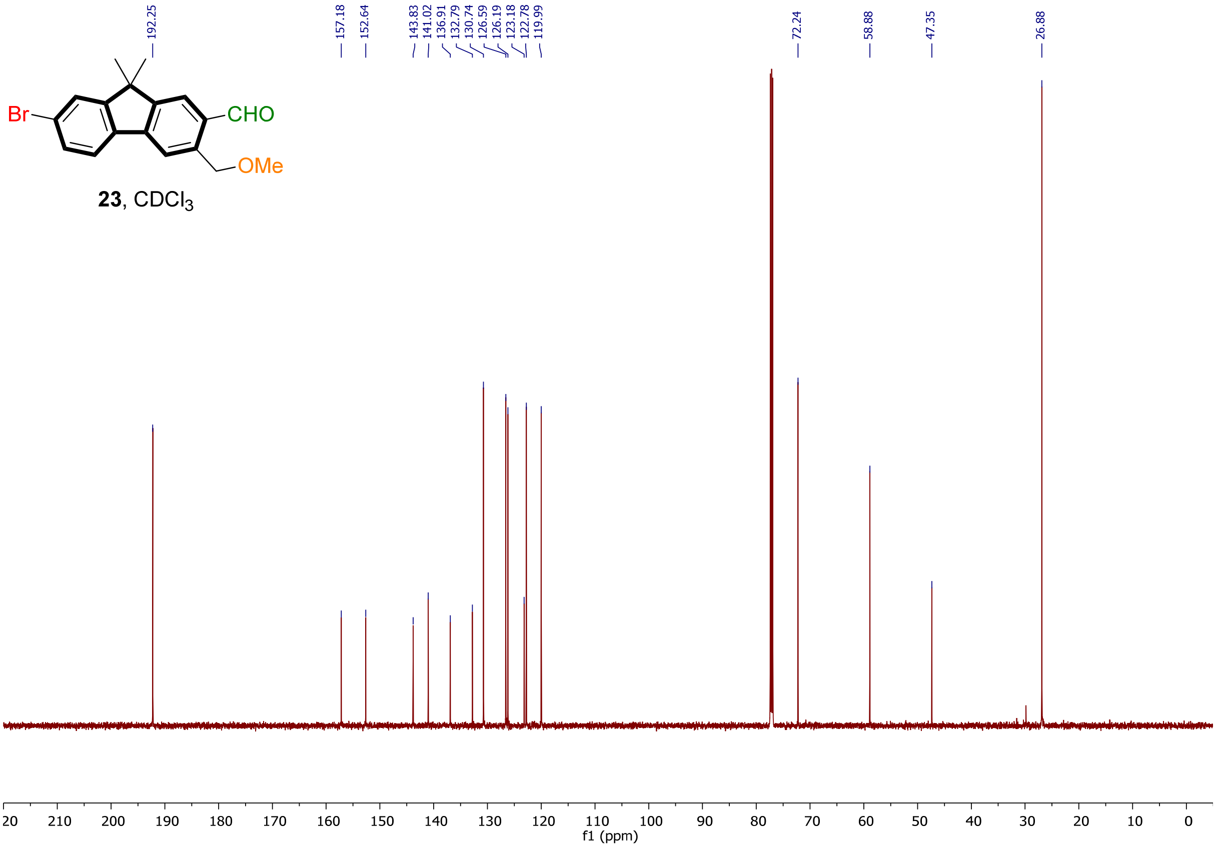

23, $CDCl_3$

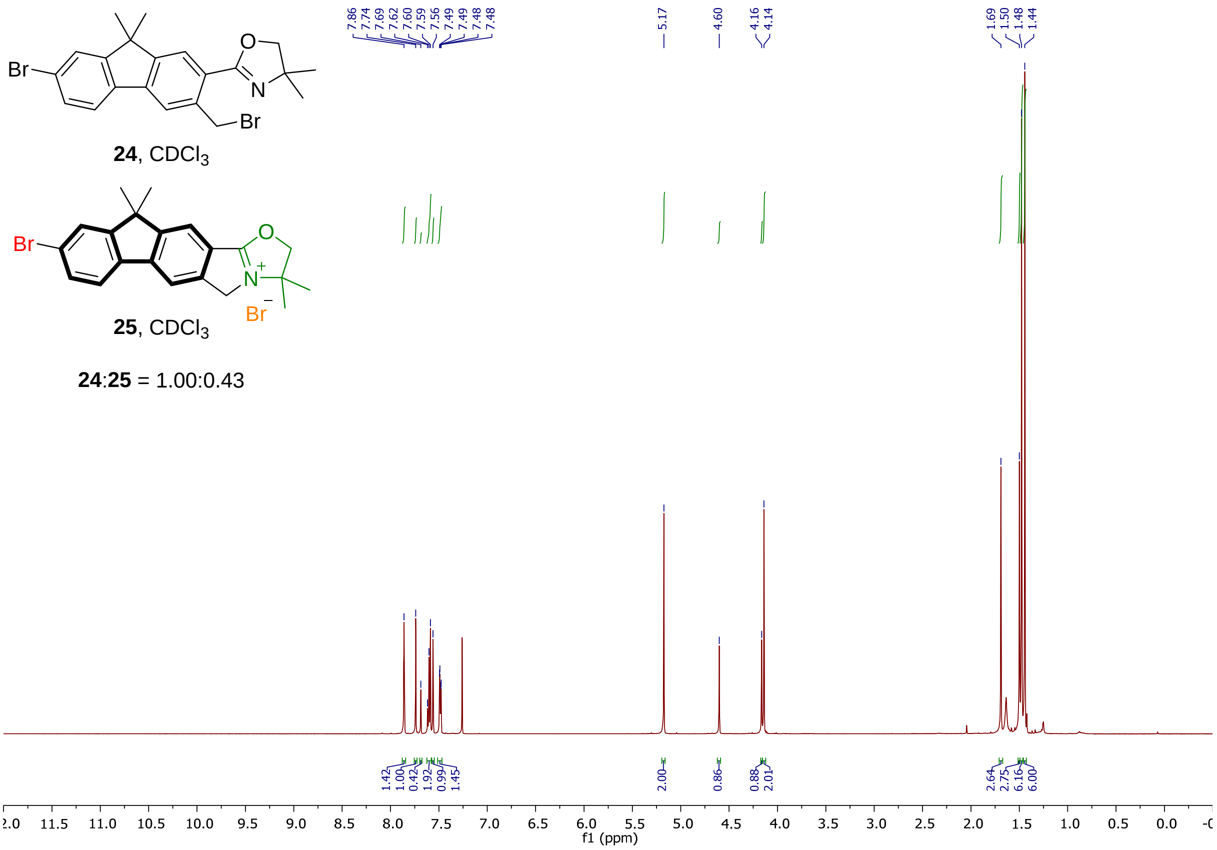
7.86 7.74 7.69 7.62 7.60 7.60 7.59 7.56 7.49 7.49 7.48 7.48
5.17
4.60
4.16 4.14
1.69 1.50 1.48 1.44
24, CDCl3
25, CDCl3
24:25 = 1.00:0.43
1.42 1.00 0.42 1.92 0.99 1.45
2.00
0.86
0.88 2.01
2.64 2.75 6.16 6.00
f1 (ppm)

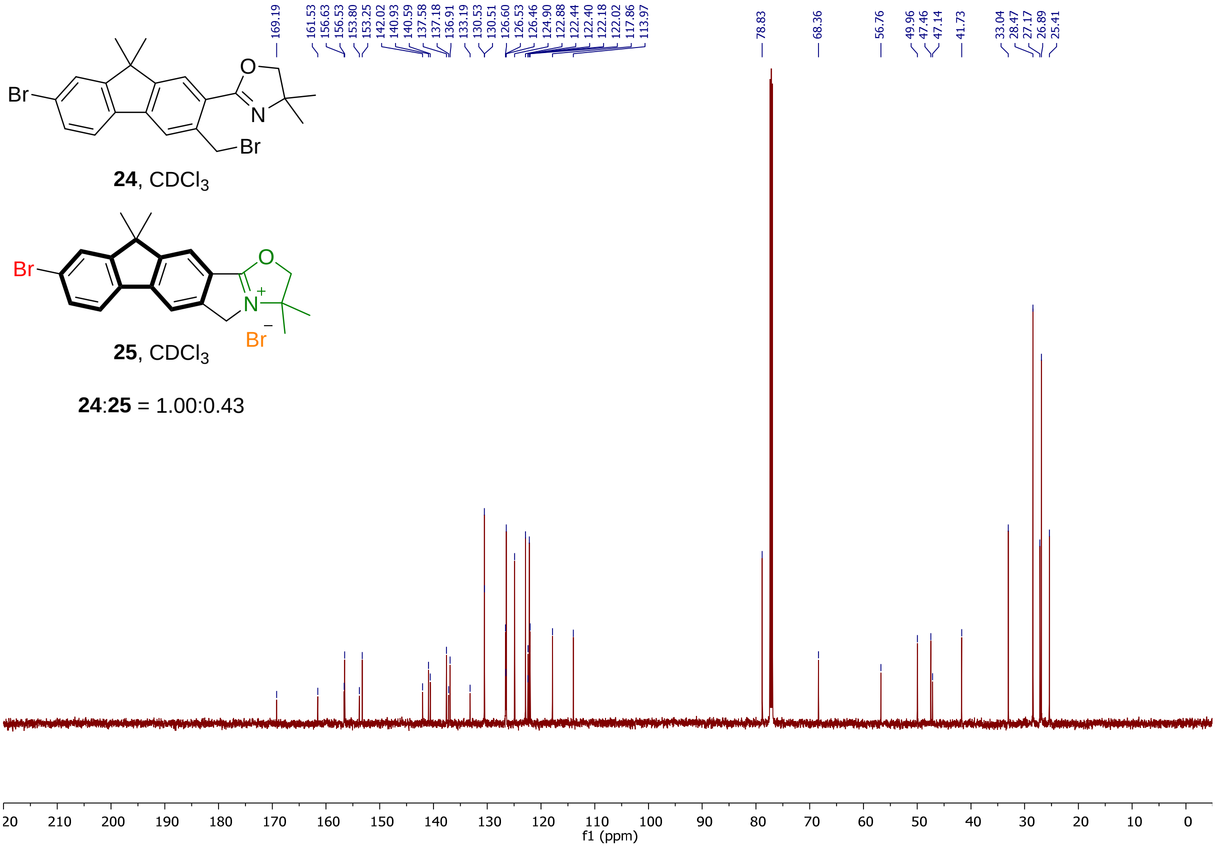
169.19
161.53 156.63 156.53 153.80 153.25 142.02 140.93 140.59 137.58 137.18 136.91 133.19 130.53 130.51 126.60 126.53 126.46 124.90 122.88 122.44 122.40 122.18 122.02 117.86 113.97
78.83
68.36
56.76
49.96 47.46 47.14
41.73
33.04 28.47 27.17 26.89 25.41
24, CDCl3
25, CDCl3
24:25 = 1.00:0.43
f1 (ppm)

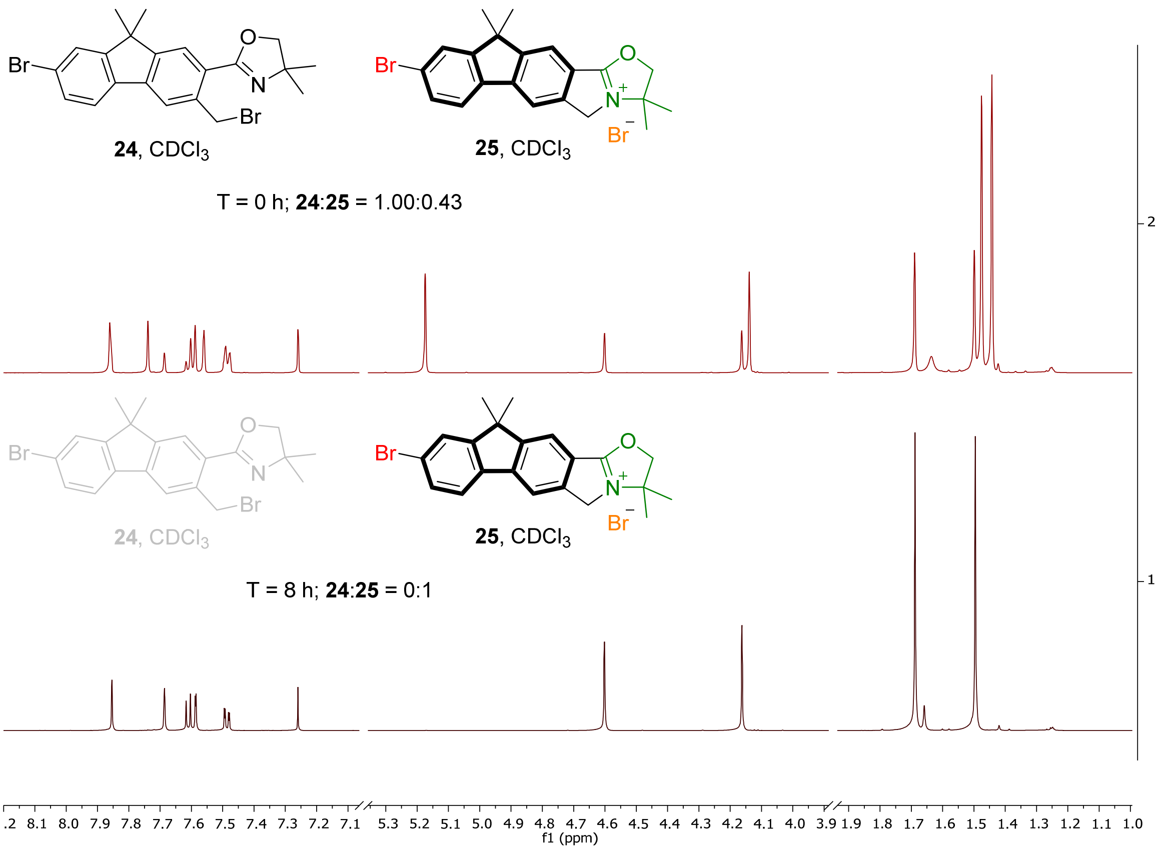
24, CDCl3
25, CDCl3
T = 0 h; 24:25 = 1.00:0.43
T = 8 h; 24:25 = 0:1
f1 (ppm)

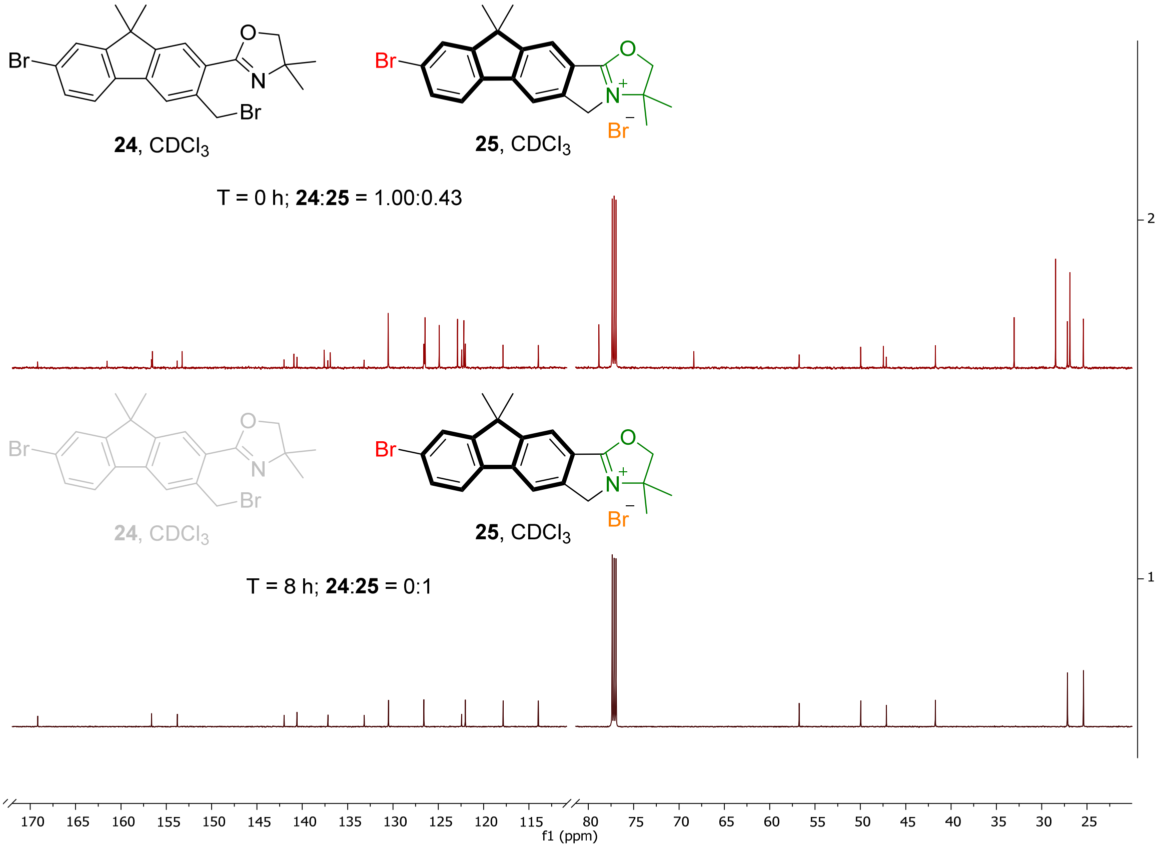
24, CDCl3
25, CDCl3
T = 0 h; 24:25 = 1.00:0.43
T = 8 h; 24:25 = 0:1
f1 (ppm)

25, $CDCl_3$

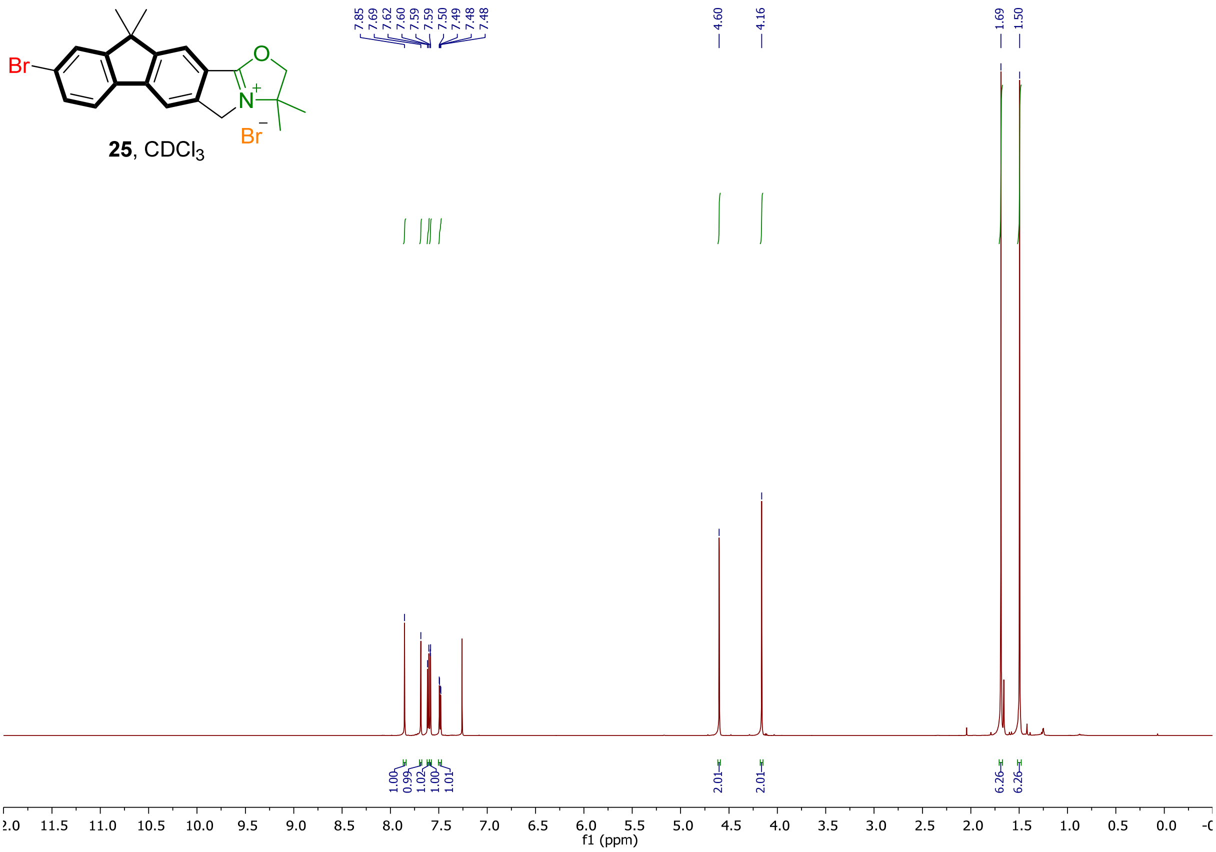

25, $CDCl_3$

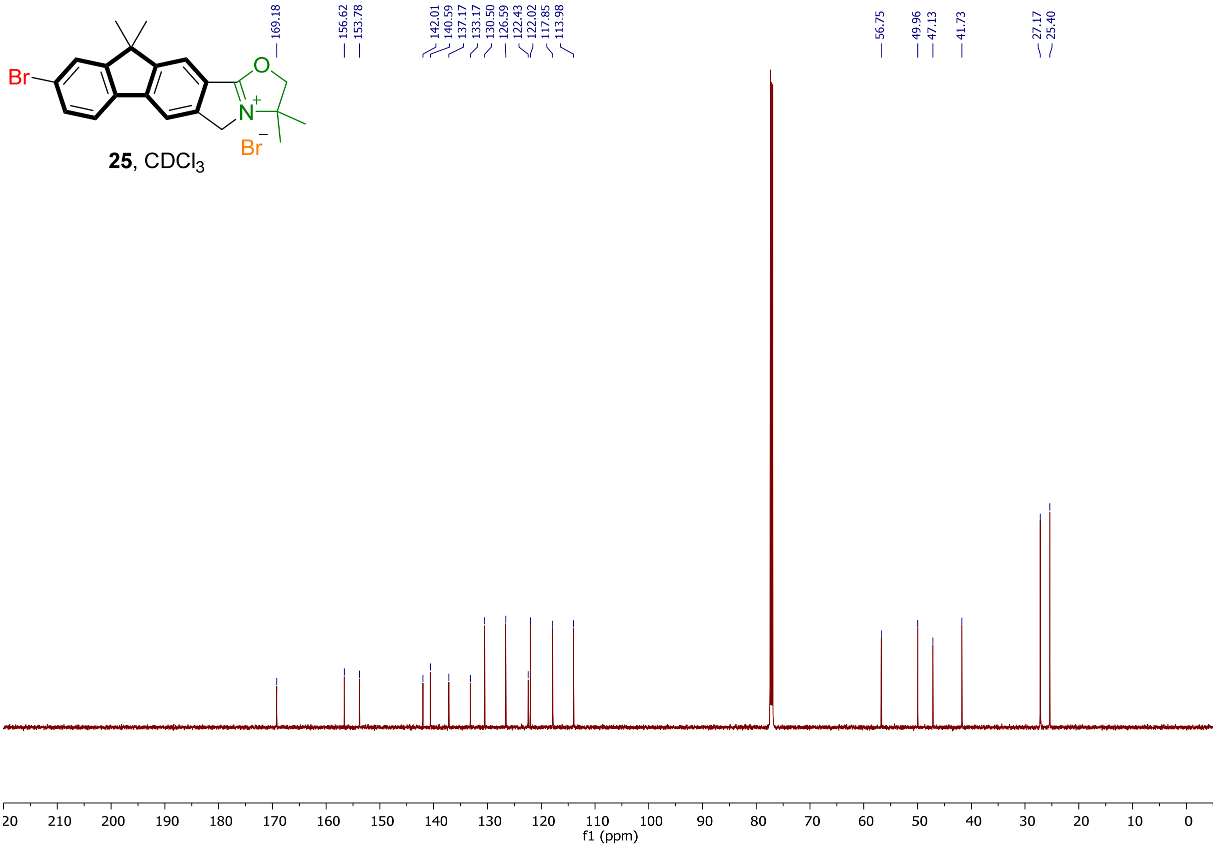

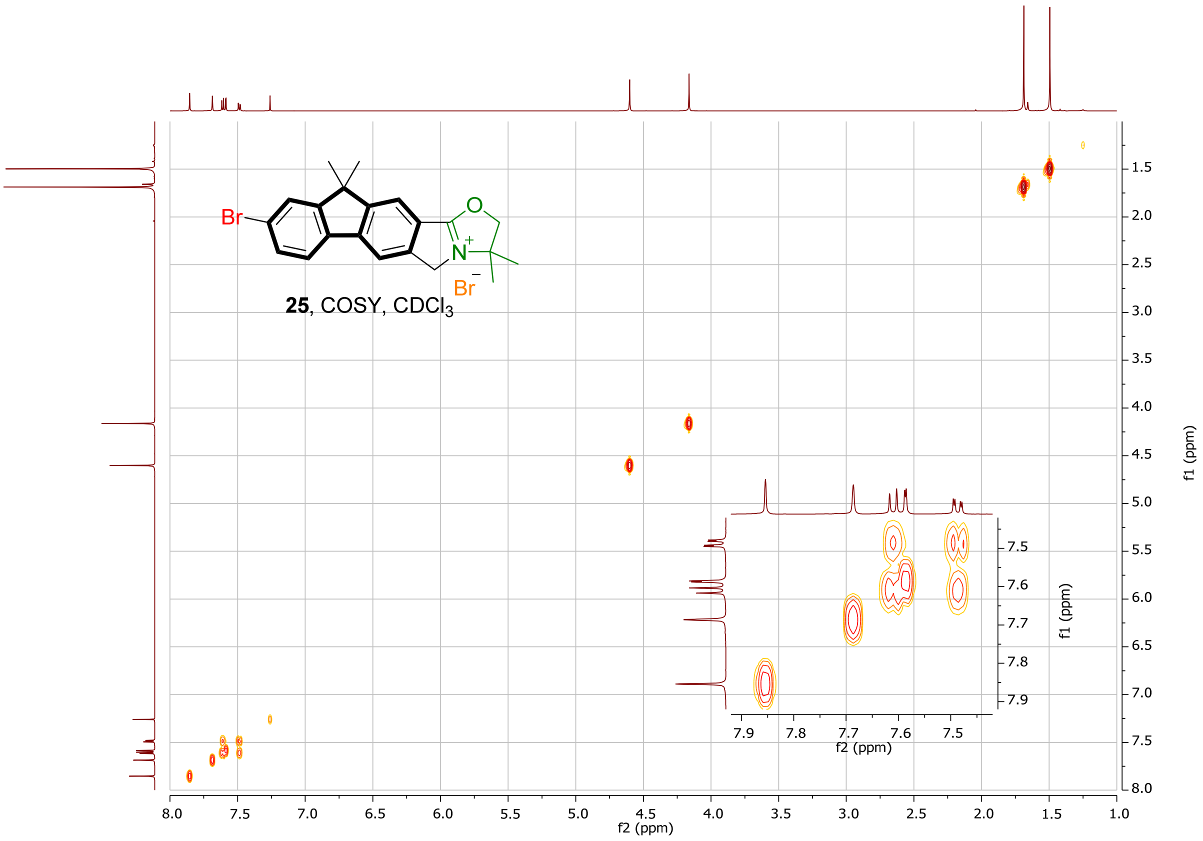
Br
O
N
+
Br
−
25, COSY, CDCl3
f2 (ppm)
f1 (ppm)

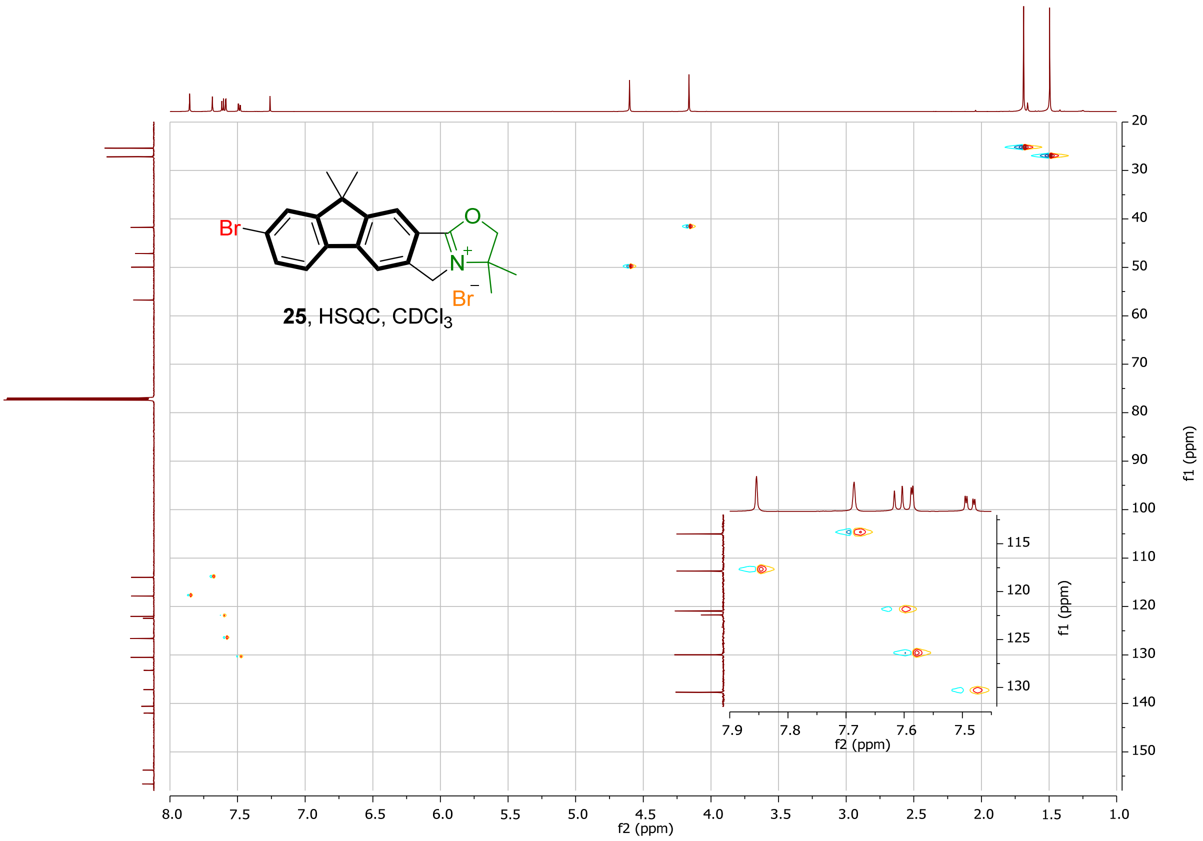
Br
O
N
+
Br
−
25, HSQC, CDCl3
f2 (ppm)
f1 (ppm)

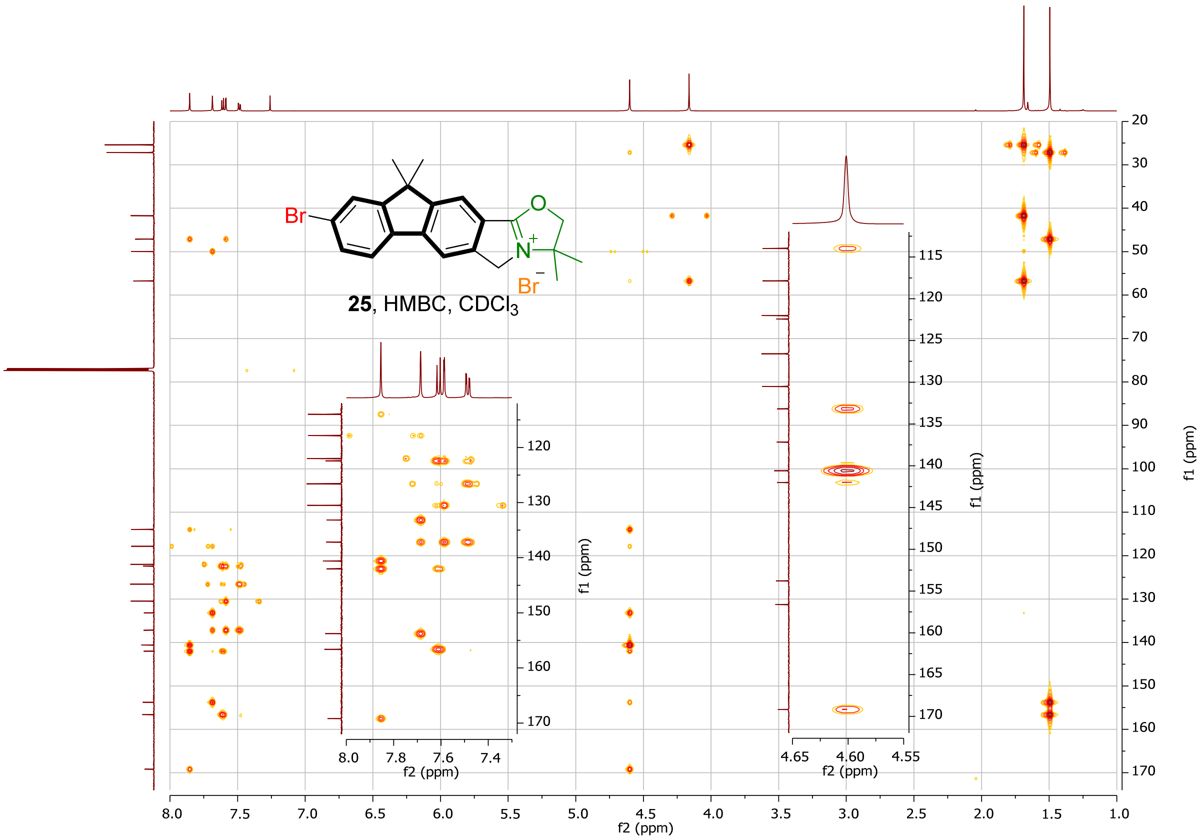
Br
O
N+
Br−
25, HMBC, CDCl3
f2 (ppm)
f1 (ppm)

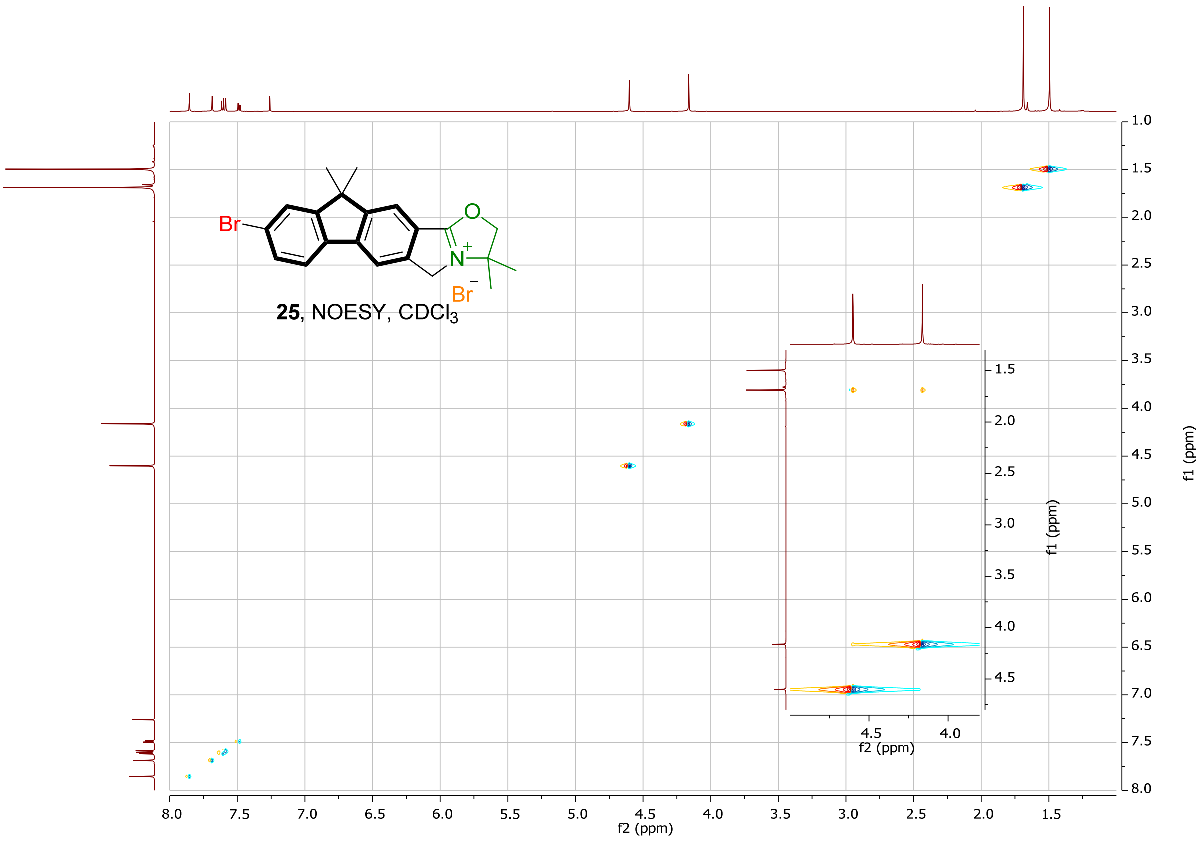
Br
O
N+
Br−
25, NOESY, CDCl3
f2 (ppm)
f1 (ppm)

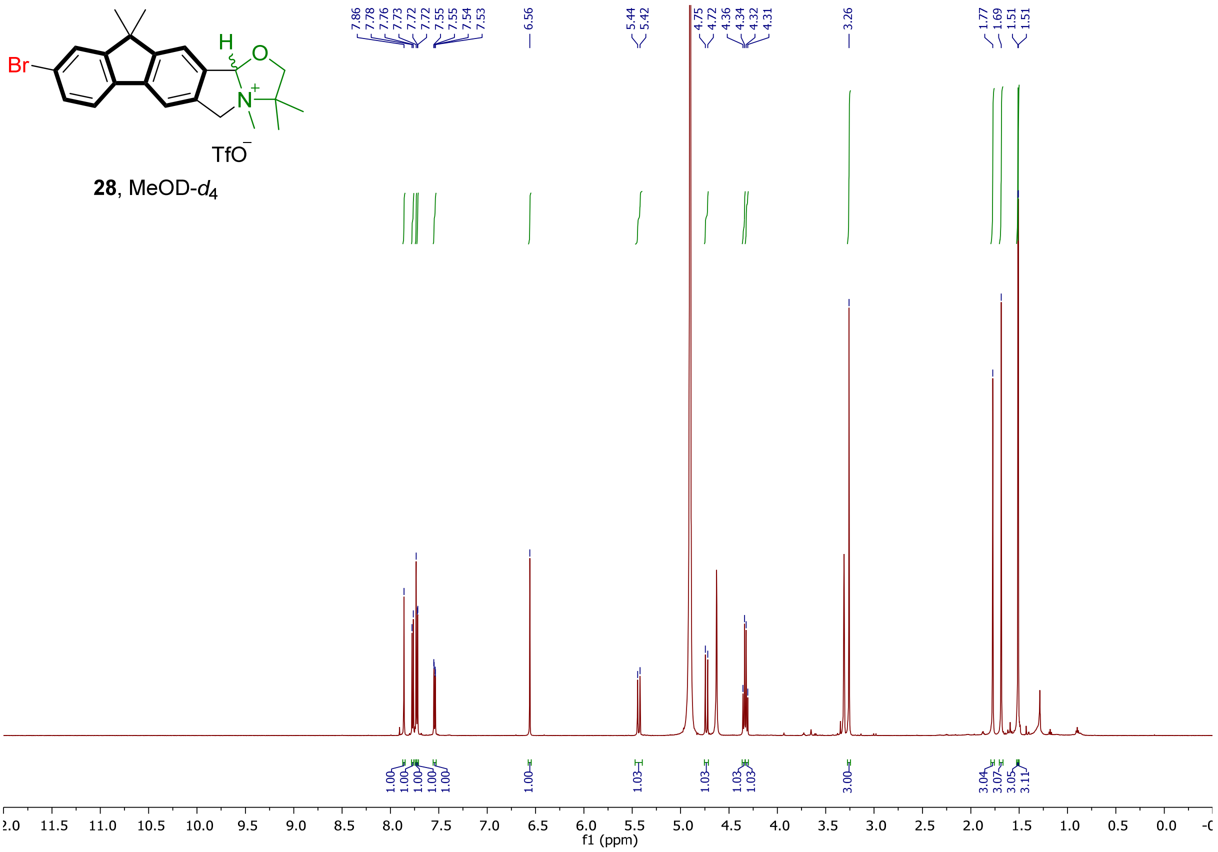
Br
H
O
N
+
TfO
−
28, MeOD-d_4
7.86
7.78
7.76
7.73
7.72
7.72
7.55
7.55
7.54
7.53
6.56
5.44
5.42
4.75
4.72
4.36
4.34
4.32
4.31
3.26
1.77
1.69
1.51
1.51
1.00
1.00
1.00
1.00
1.00
1.00
1.03
1.03
1.03
1.03
3.00
3.04
3.07
3.05
3.11
f1 (ppm)

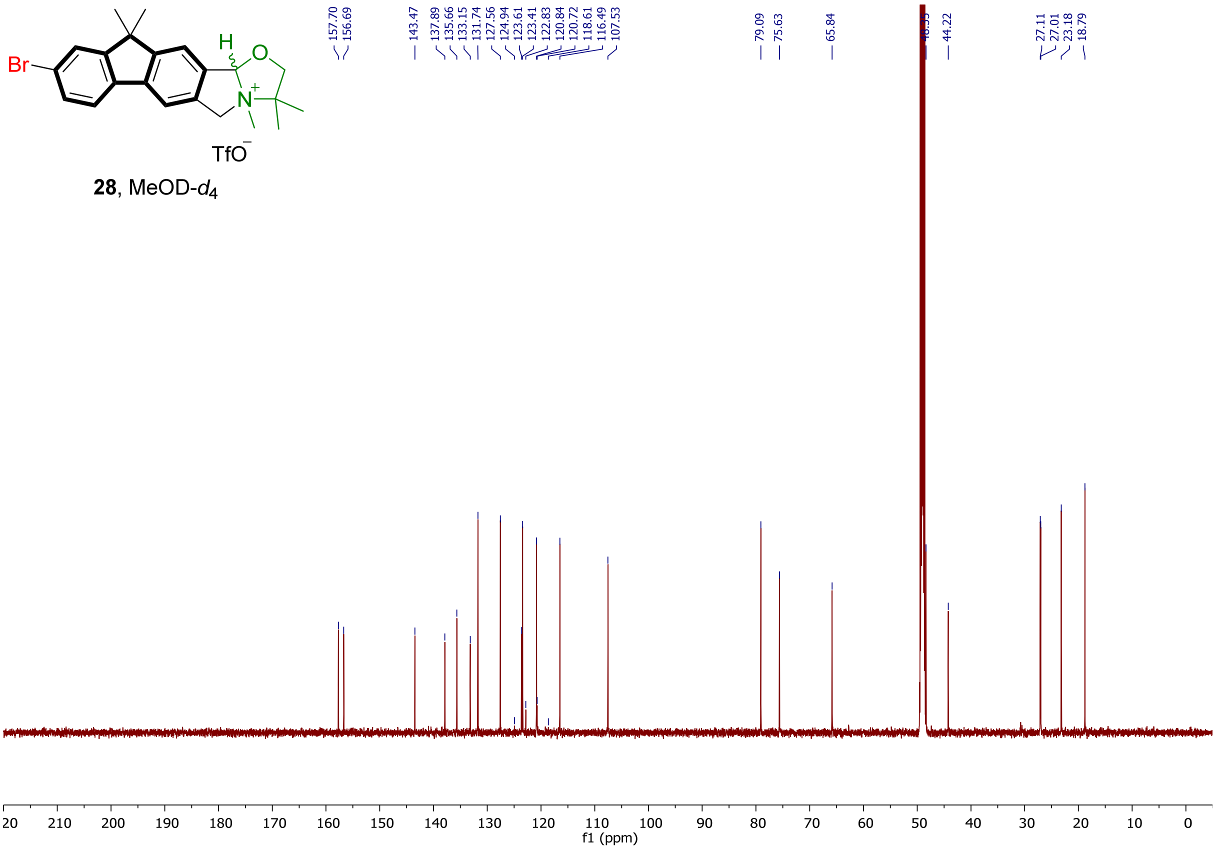
Br
H
O
N
+
TfO
−
28, MeOD-d_4
157.70
156.69
143.47
137.89
135.66
133.15
131.74
127.56
124.94
123.61
123.41
122.83
120.84
120.72
118.61
116.49
107.53
79.09
75.63
65.84
44.22
27.11
27.01
23.18
18.79
f1 (ppm)

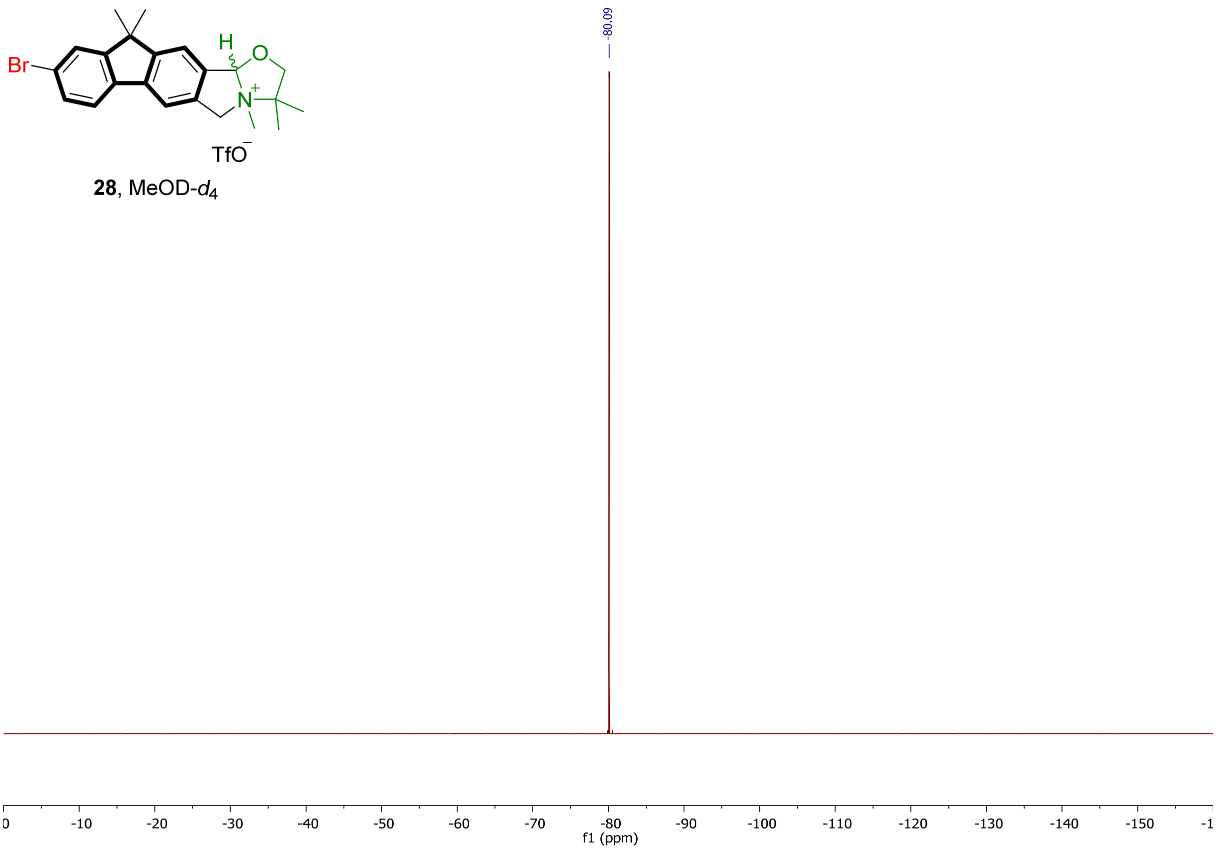

Br
H
O
N
+
TfO−
28, MeOD-d4
-80.09
f1 (ppm)

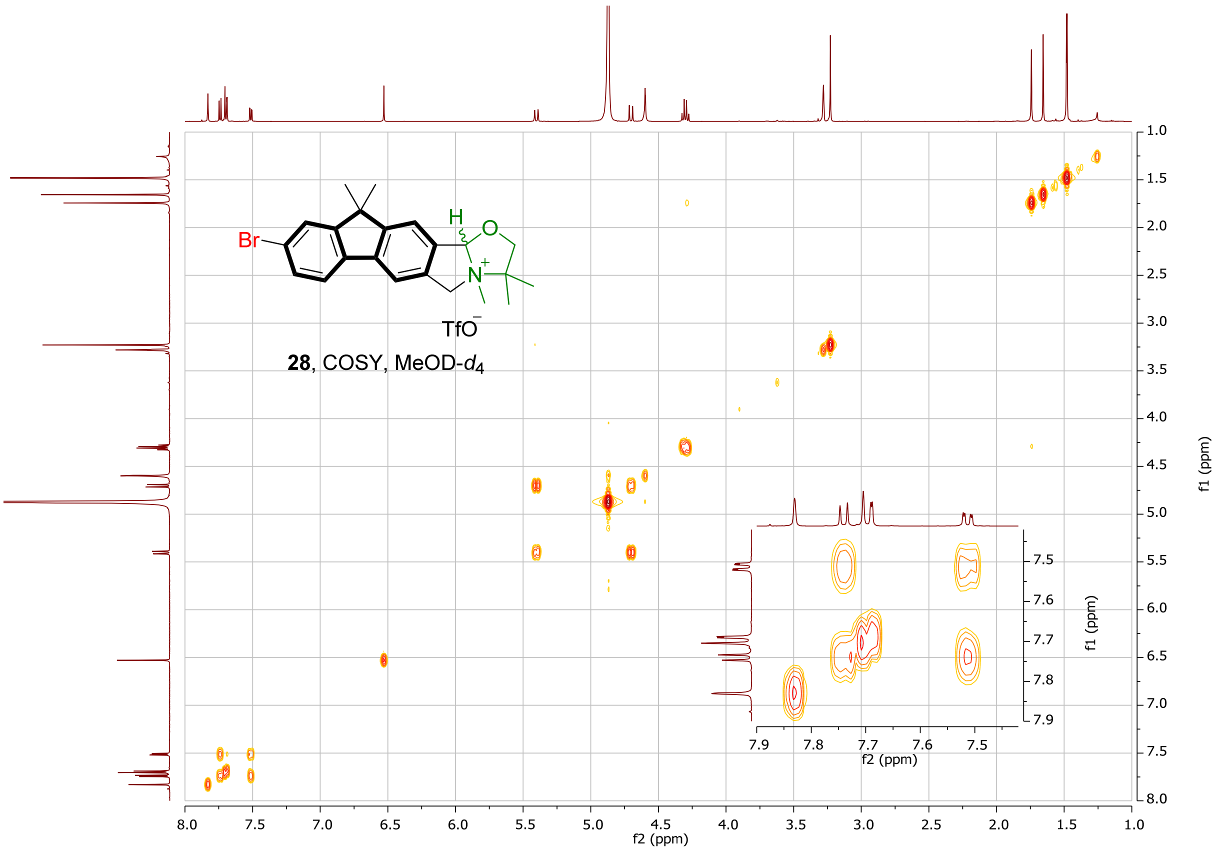

Br
H
O
N
+
TfO−
28, COSY, MeOD-d4
f2 (ppm)
f1 (ppm)

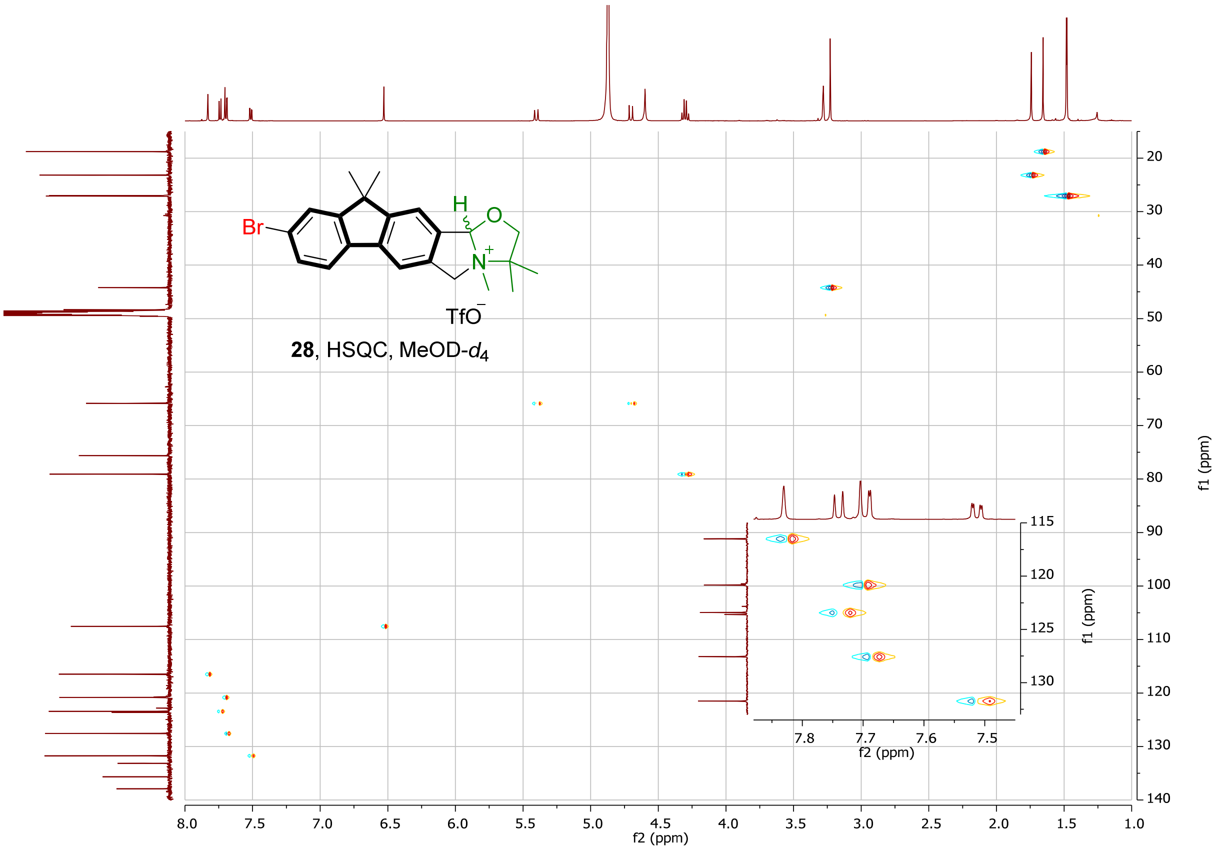
Br
H
O
N
TfO
28, HSQC, MeOD-d4
f2 (ppm)
f1 (ppm)

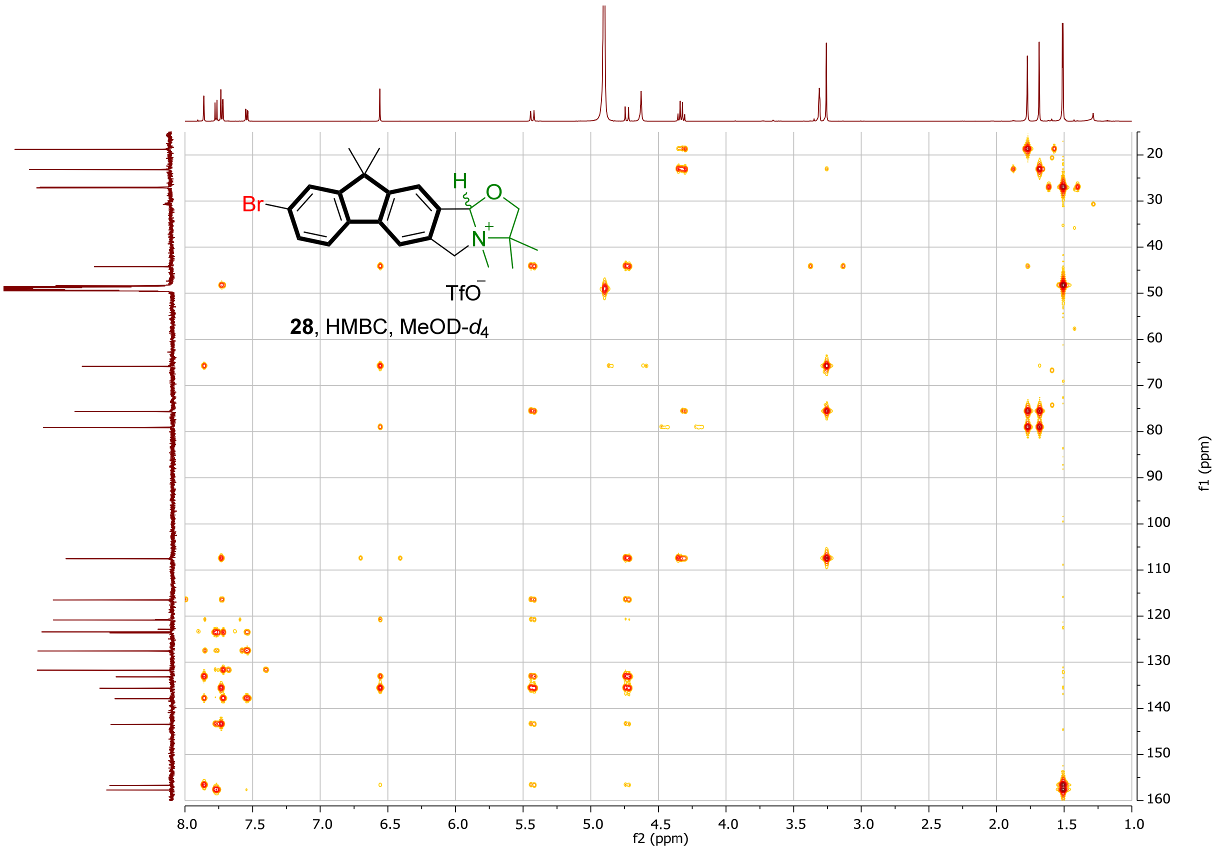
Br
H
O
N
TfO
28, HMBC, MeOD-d4
f2 (ppm)
f1 (ppm)

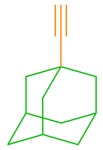

31, $CDCl_3$

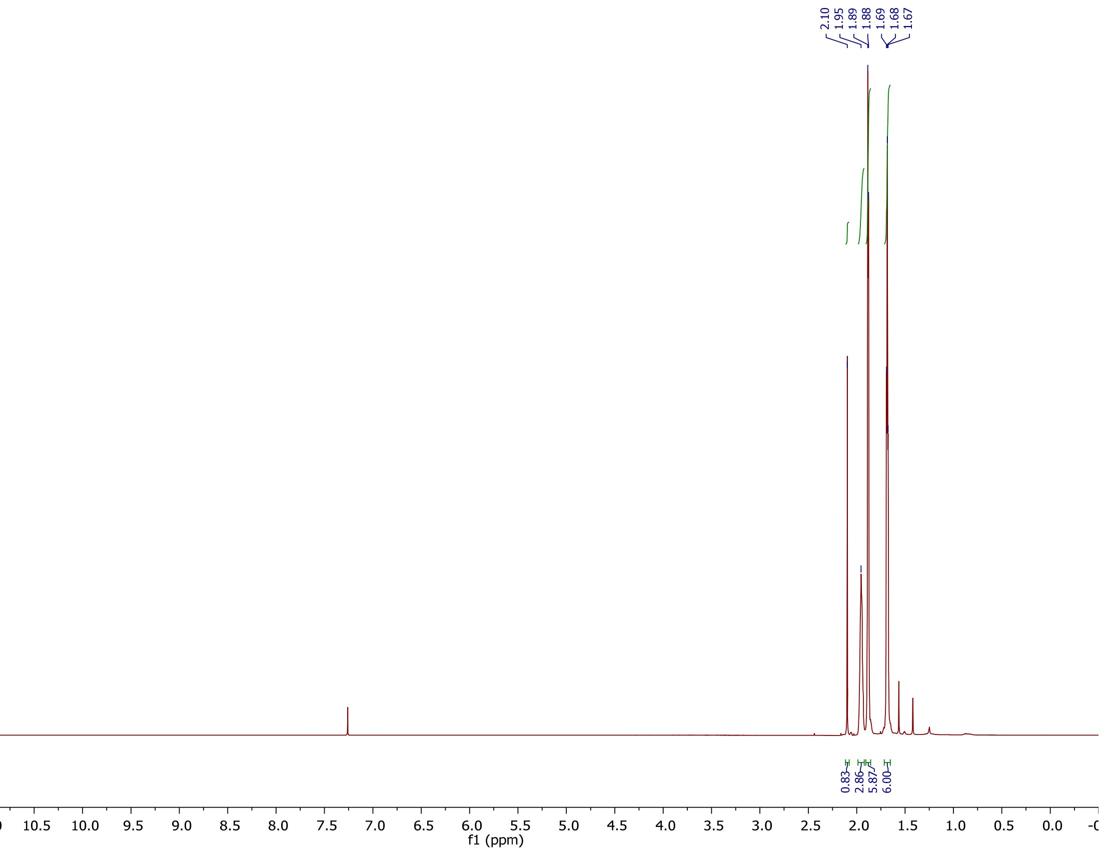

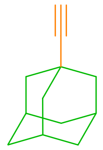

31, $CDCl_3$

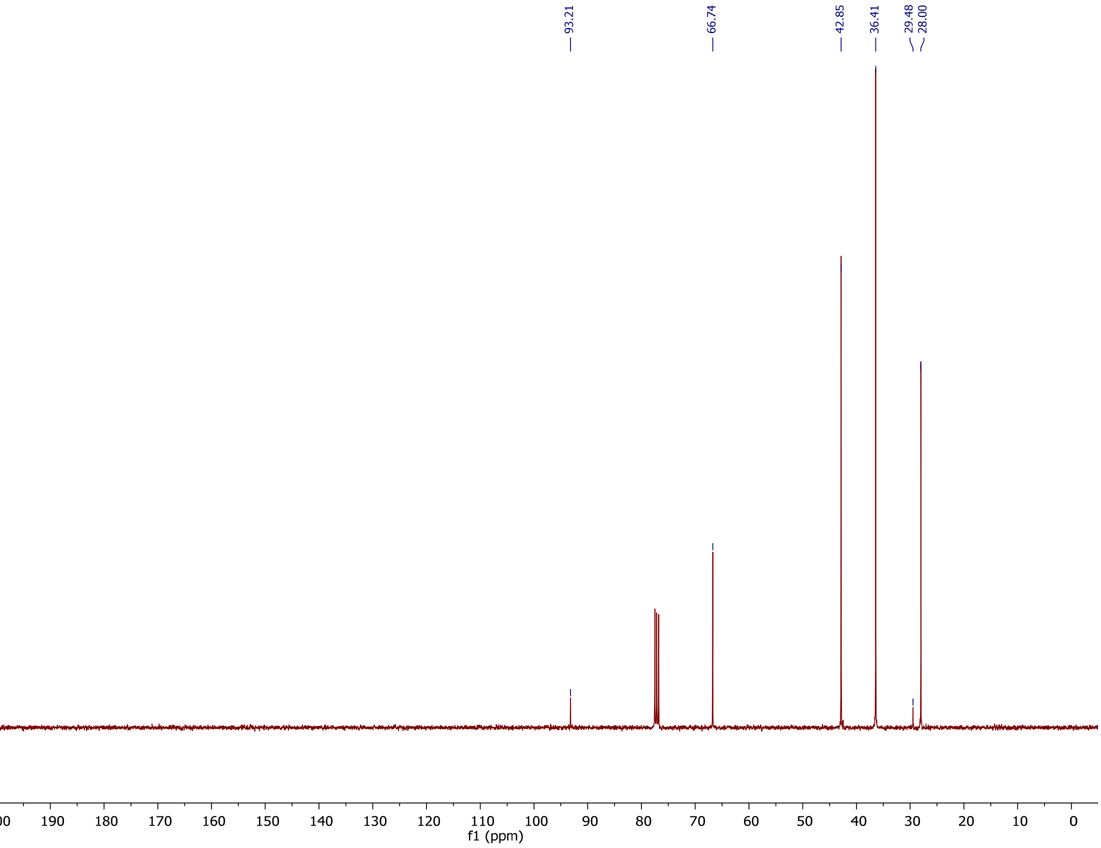

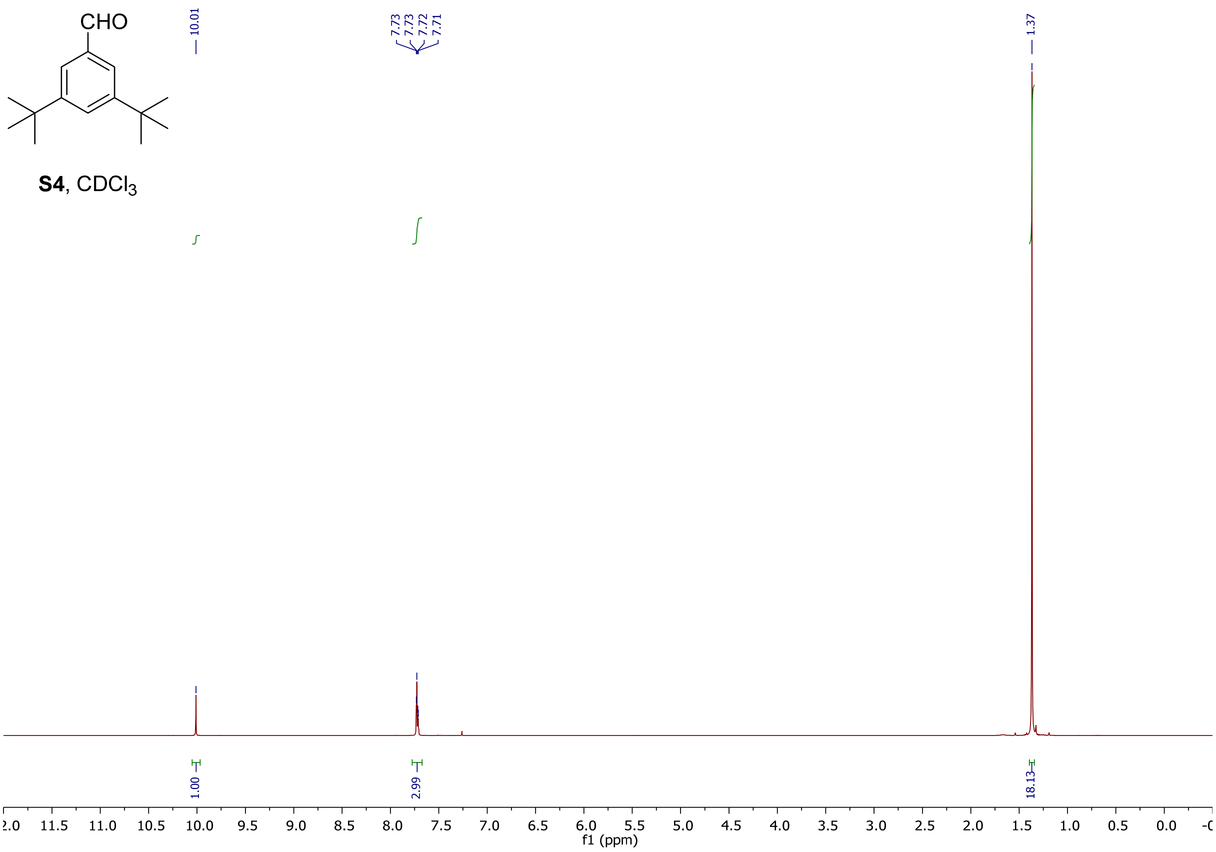
CHO
S4, CDCl3
10.01
7.73
7.73
7.72
7.71
1.37
1.00
2.99
18.13
f1 (ppm)

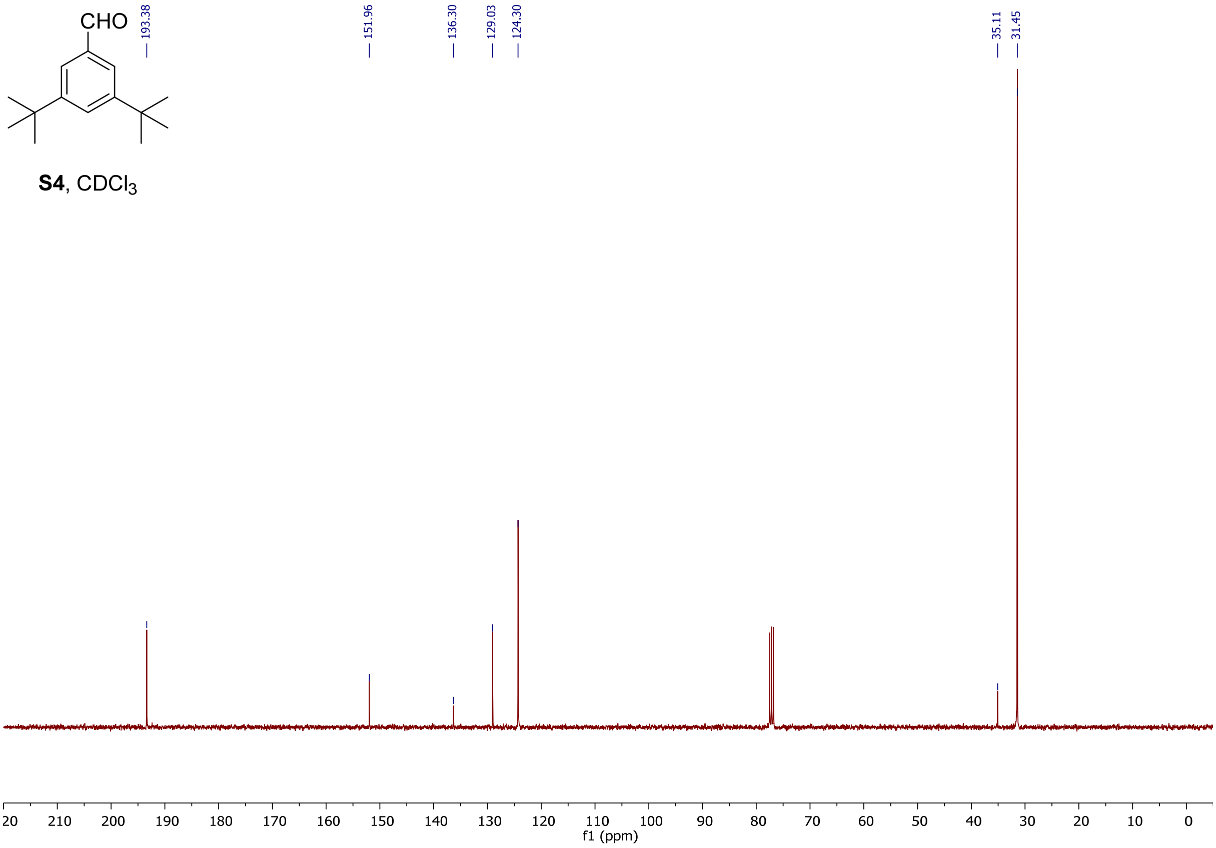
CHO
S4, CDCl3
193.38
151.96
136.30
129.03
124.30
35.11
31.45
f1 (ppm)

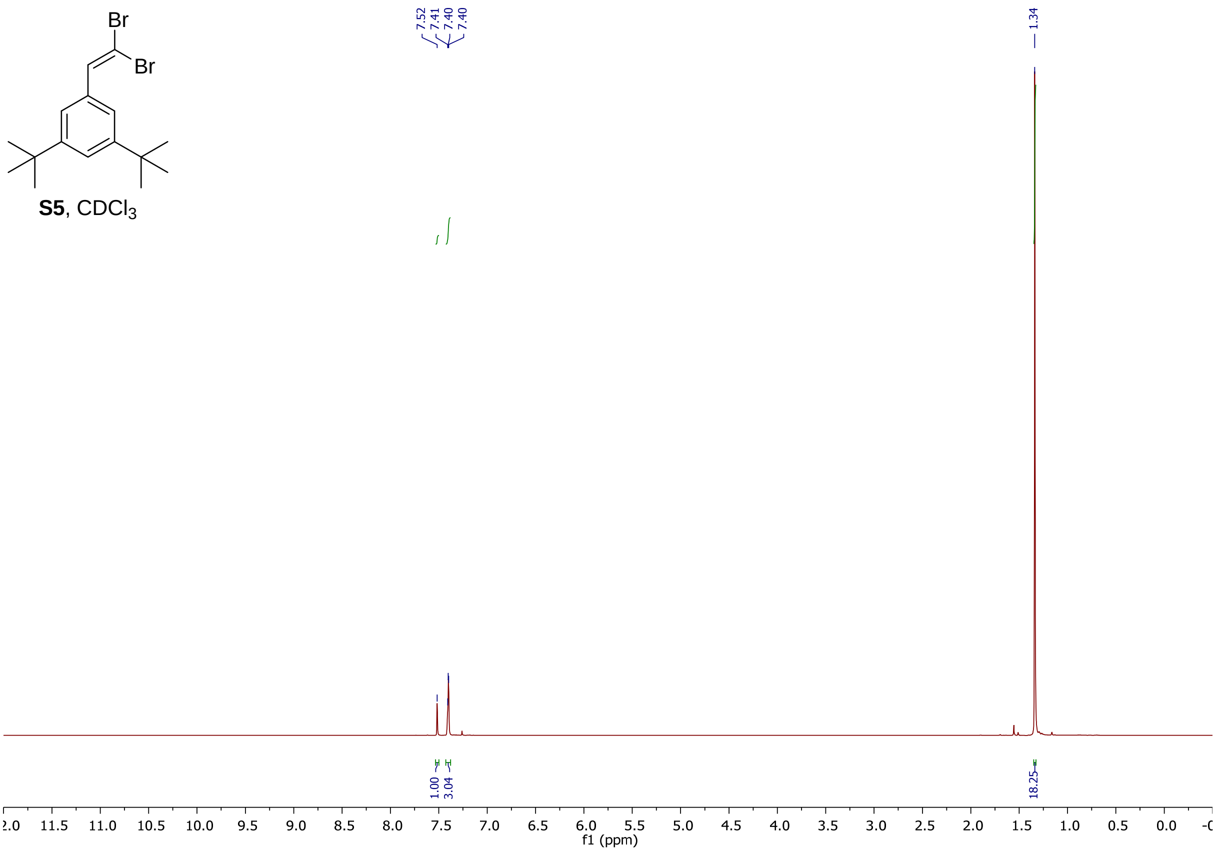
Br
Br
S5, CDCl3
7.52
7.41
7.40
7.40
1.34
1.00
3.04
18.25
2.0
11.5
11.0
10.5
10.0
9.5
9.0
8.5
8.0
7.5
7.0
6.5
6.0
5.5
5.0
4.5
4.0
3.5
3.0
2.5
2.0
1.5
1.0
0.5
0.0
f1 (ppm)

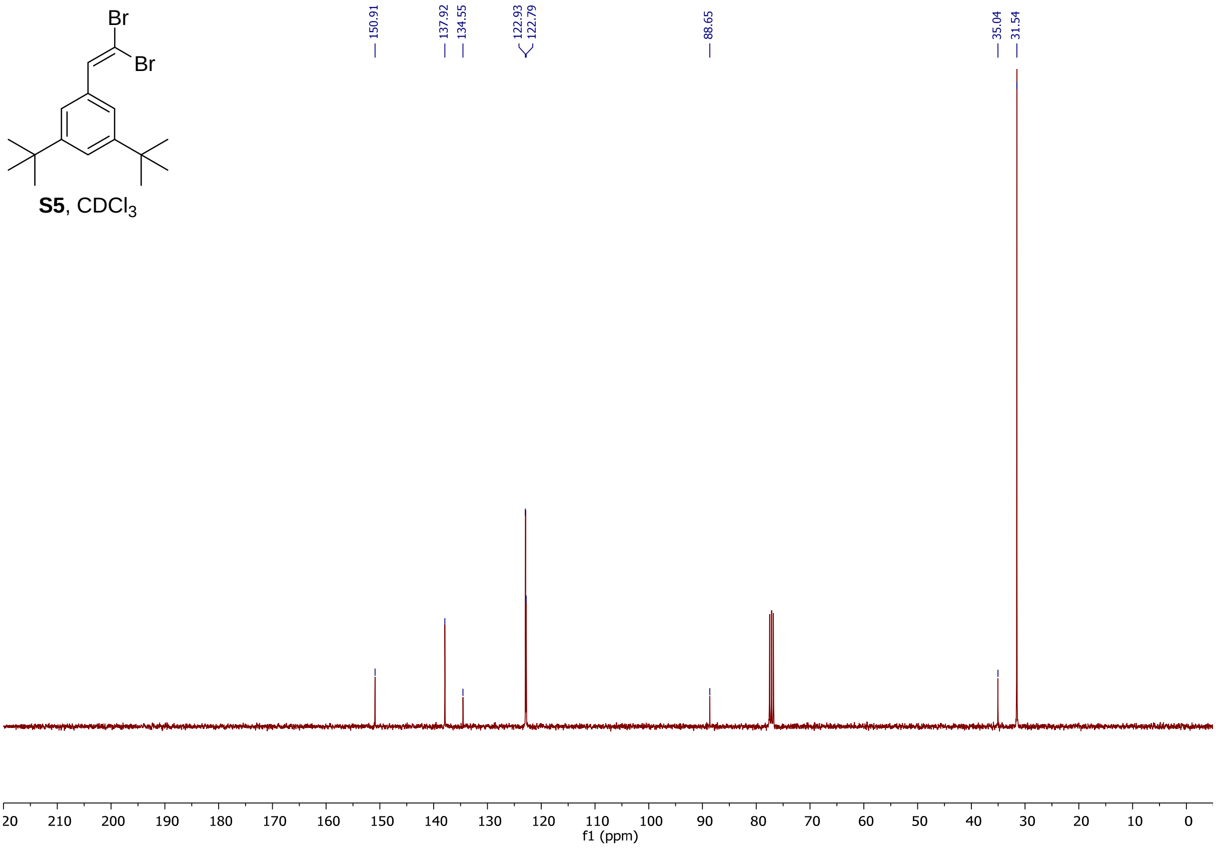
Br
Br
S5, CDCl3
150.91
137.92
134.55
122.93
122.79
88.65
35.04
31.54
20
210
200
190
180
170
160
150
140
130
120
110
100
90
80
70
60
50
40
30
20
10
0
f1 (ppm)

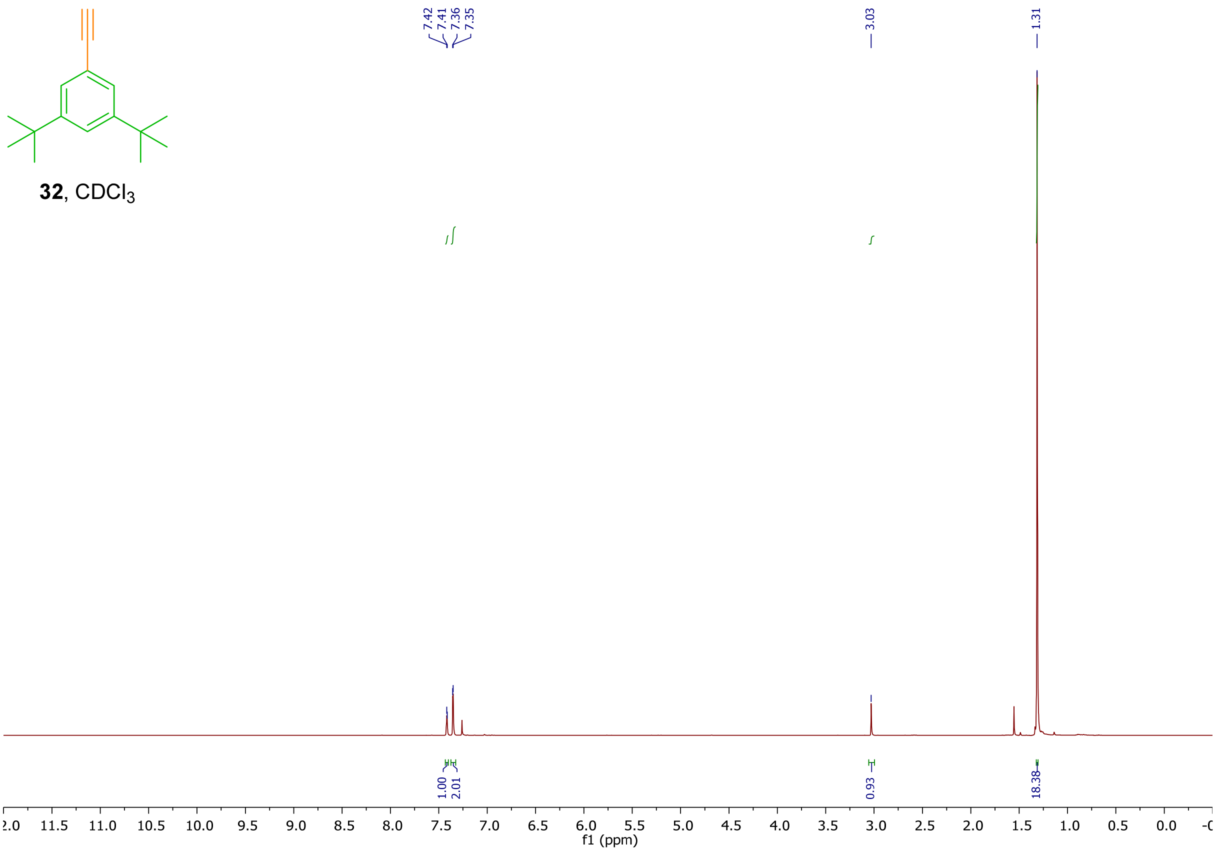
32, CDCl3
7.42
7.41
7.36
7.35
3.03
1.31
1.00
2.01
0.93
18.38
f1 (ppm)

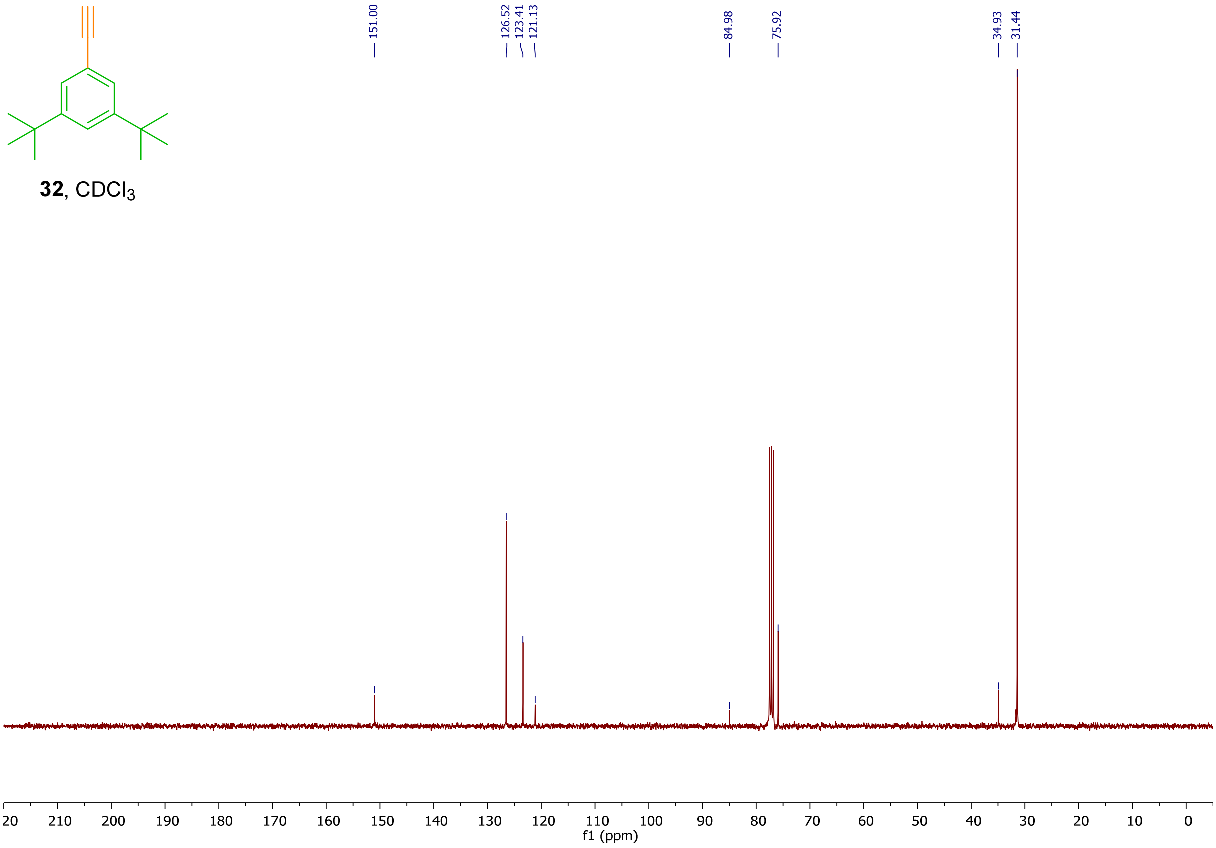
32, CDCl3
151.00
126.52
123.41
121.13
84.98
75.92
34.93
31.44
f1 (ppm)

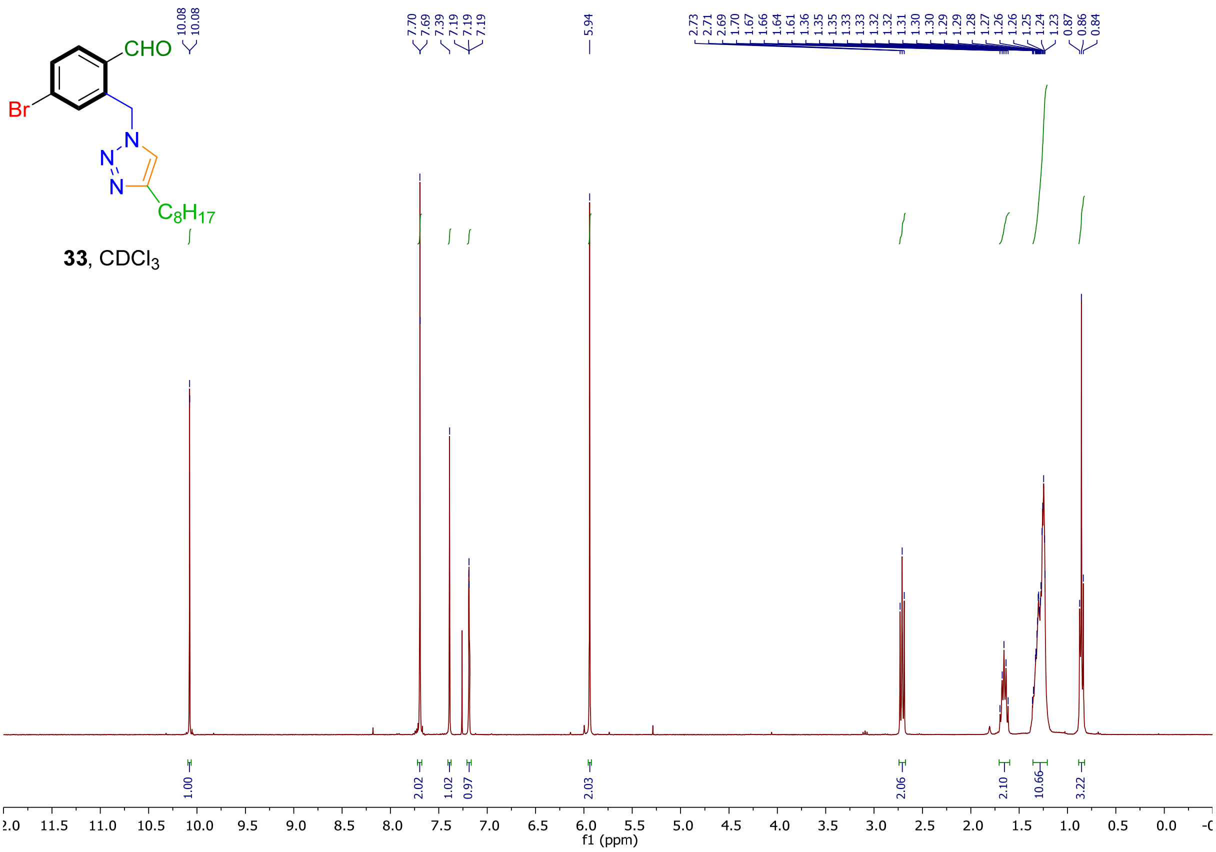

CHO
Br
N
N
N
C8H17
33, CDCl3
10.08
10.08
7.70
7.69
7.39
7.19
7.19
7.19
5.94
2.73
2.71
2.69
1.70
1.67
1.66
1.64
1.61
1.36
1.35
1.35
1.33
1.33
1.32
1.32
1.31
1.30
1.30
1.29
1.29
1.28
1.27
1.26
1.26
1.25
1.24
1.23
0.87
0.86
0.84
1.00
2.02
1.02
0.97
2.03
2.06
2.10
10.66
3.22
f1 (ppm)

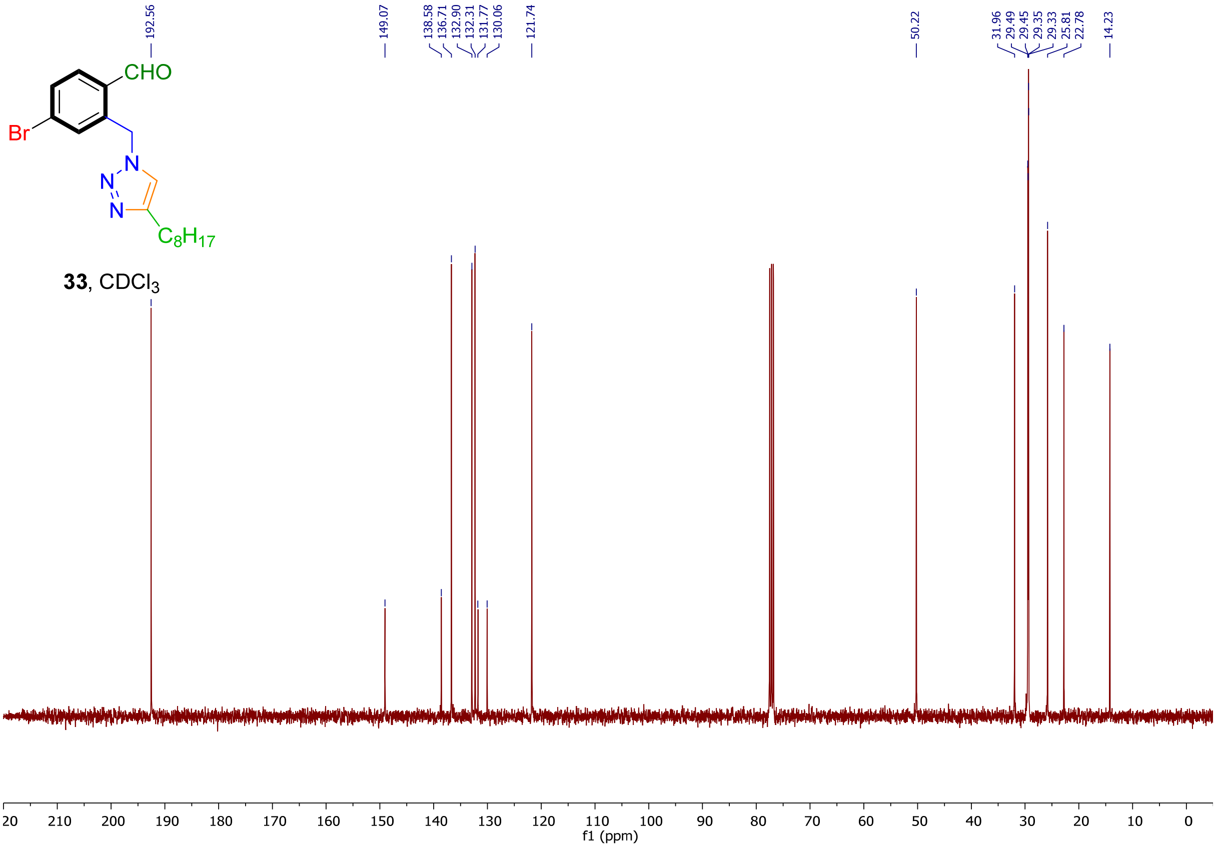

CHO
Br
N
N
N
C8H17
33, CDCl3
192.56
149.07
138.58
136.71
132.90
132.31
131.77
130.06
121.74
50.22
31.96
29.49
29.45
29.35
29.33
25.81
22.78
14.23
f1 (ppm)

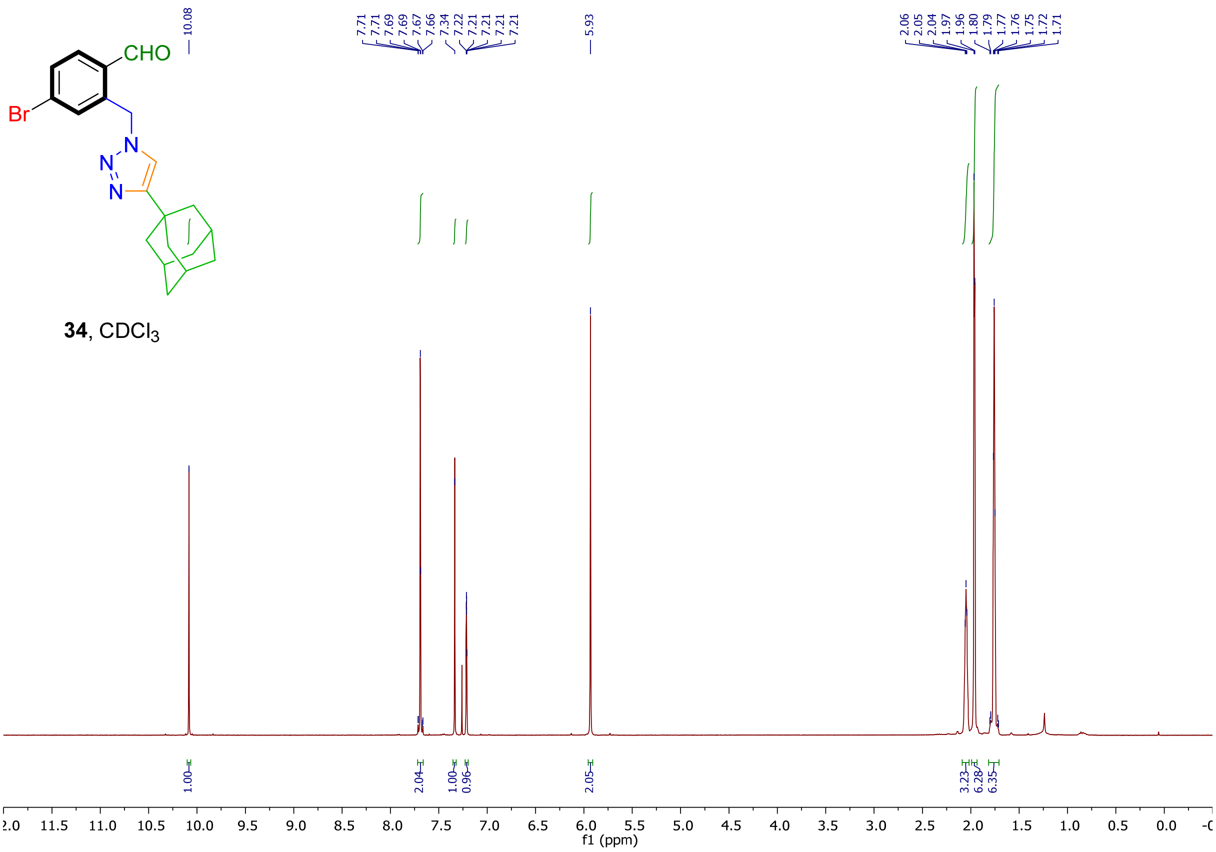
CHO
Br
N
N
N
34, CDCl3
10.08
7.71 7.71 7.69 7.67 7.66 7.34 7.22 7.21 7.21 7.21 7.21
5.93
2.06 2.05 2.04 1.97 1.96 1.80 1.79 1.77 1.76 1.75 1.72 1.71
1.00
2.04
1.00
0.96
2.05
3.23
6.28
6.35
f1 (ppm)

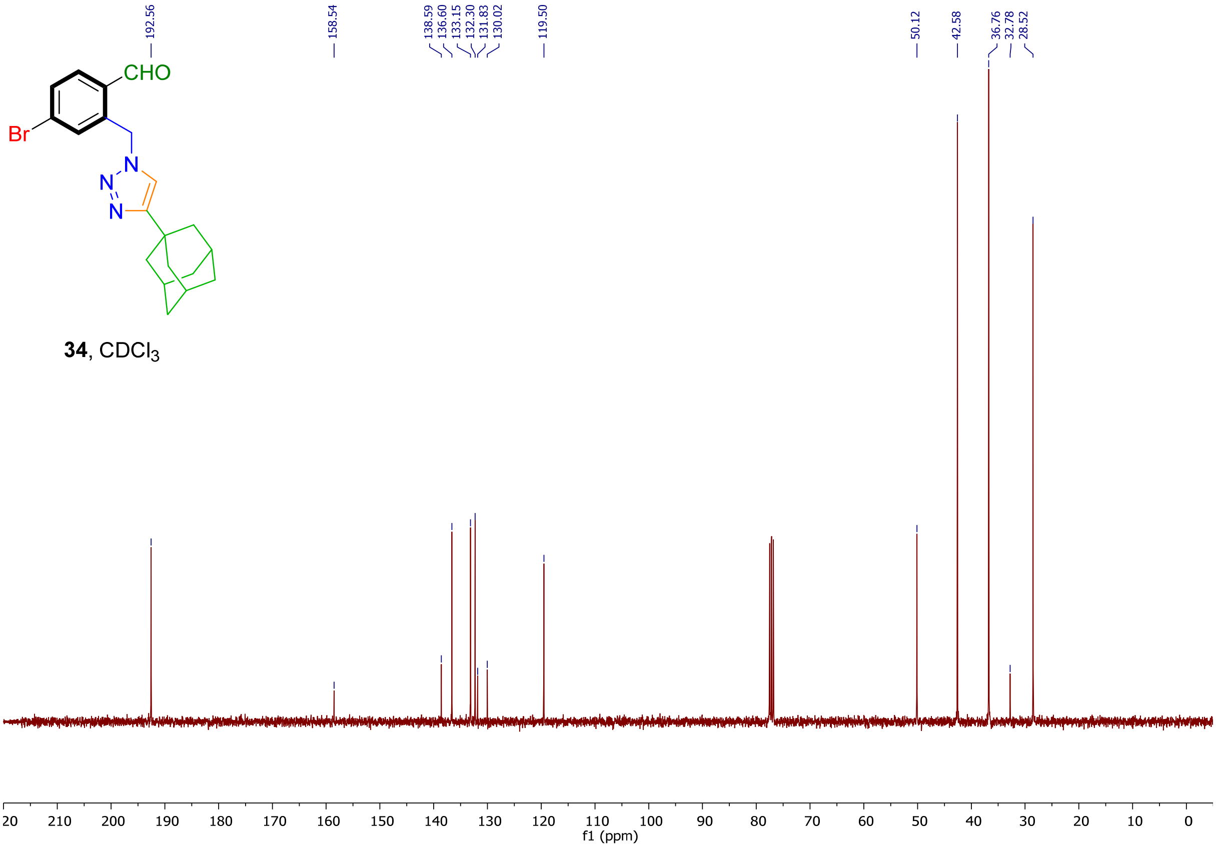
CHO
Br
N
N
N
34, CDCl3
192.56
158.54
138.59 136.60 133.15 132.30 131.83 130.02
119.50
50.12
42.58
36.76
32.78
28.52
f1 (ppm)

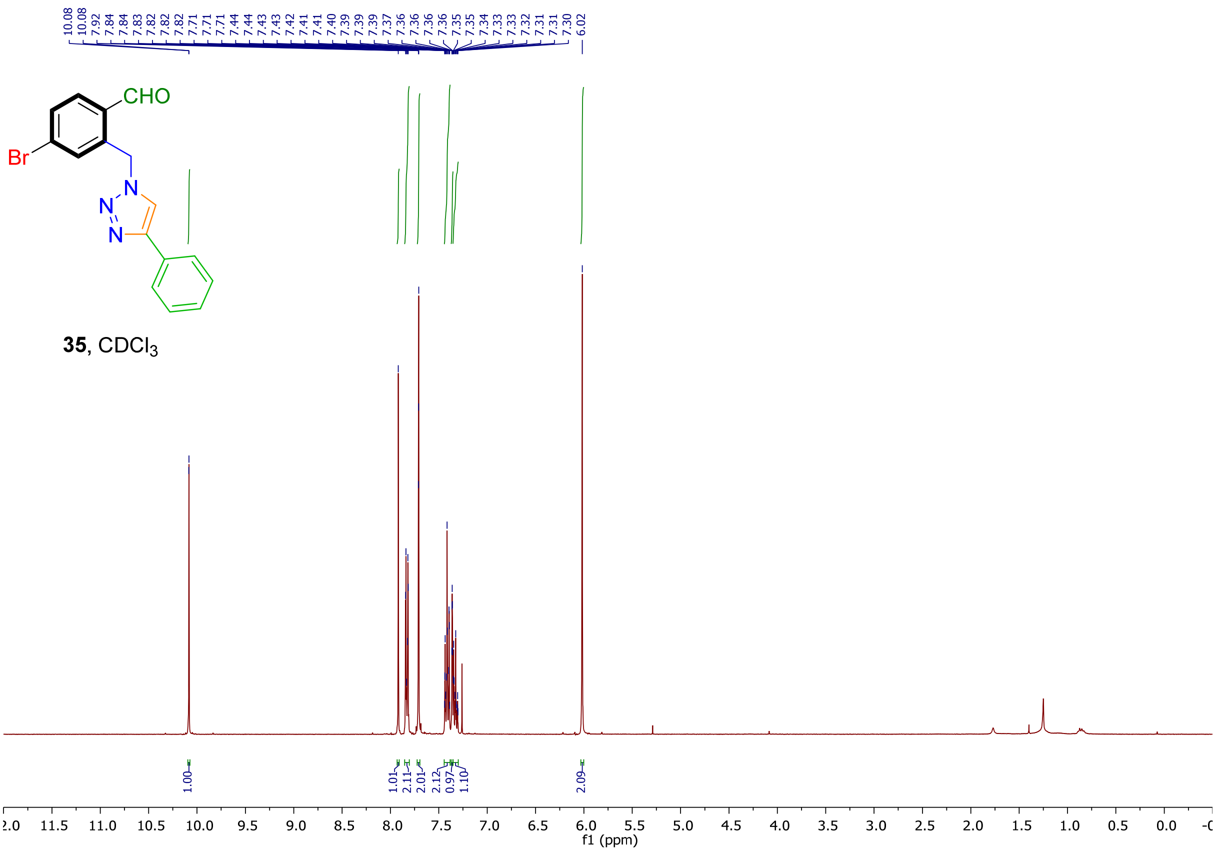
10.08
10.08
7.92
7.84
7.84
7.83
7.82
7.82
7.82
7.71
7.71
7.71
7.44
7.44
7.43
7.43
7.42
7.41
7.41
7.40
7.39
7.39
7.39
7.37
7.36
7.36
7.36
7.35
7.35
7.34
7.33
7.33
7.32
7.31
7.31
7.30
6.02
CHO
Br
N
N
N
35, CDCl3
1.00
1.01
2.11
2.01
2.12
0.97
1.10
2.09
f1 (ppm)

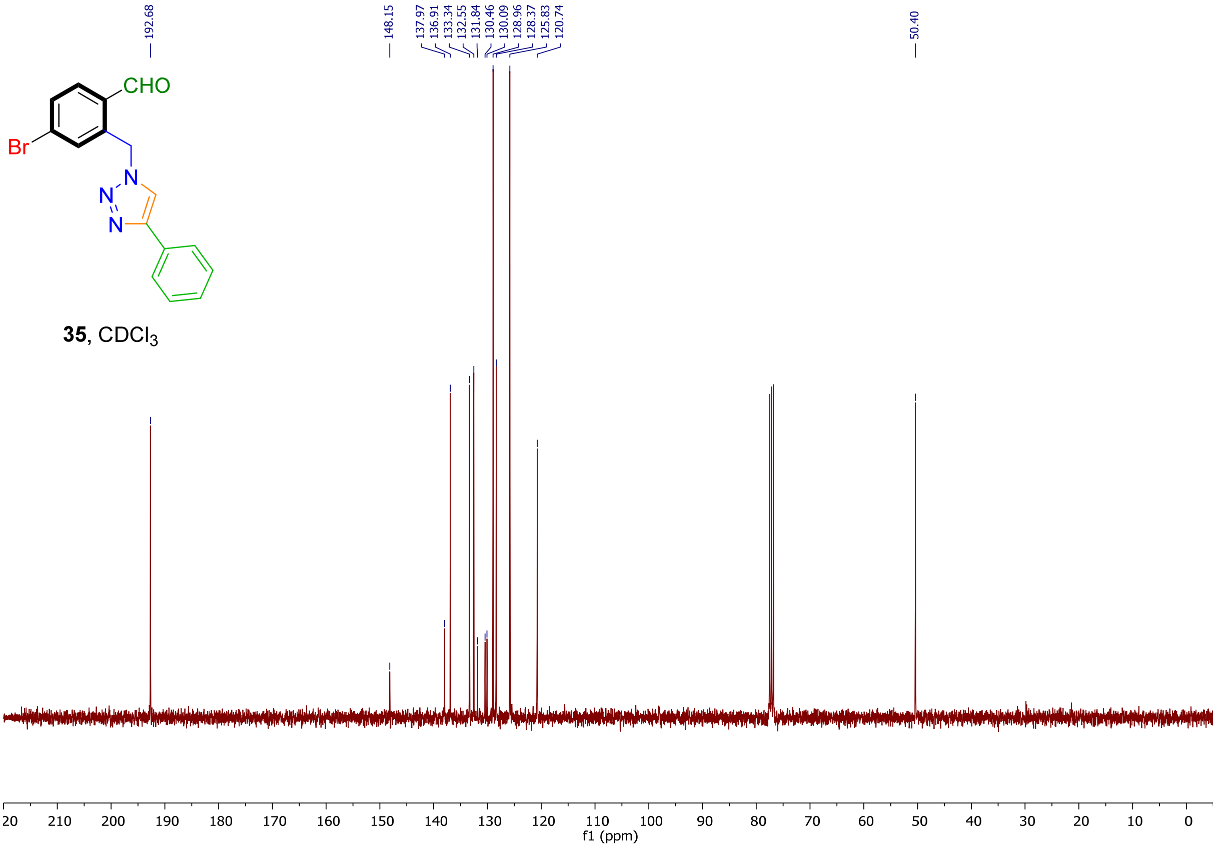
192.68
148.15
137.97
136.91
133.34
132.55
131.84
130.46
130.09
128.96
128.37
125.83
120.74
50.40
CHO
Br
N
N
N
35, CDCl3
f1 (ppm)

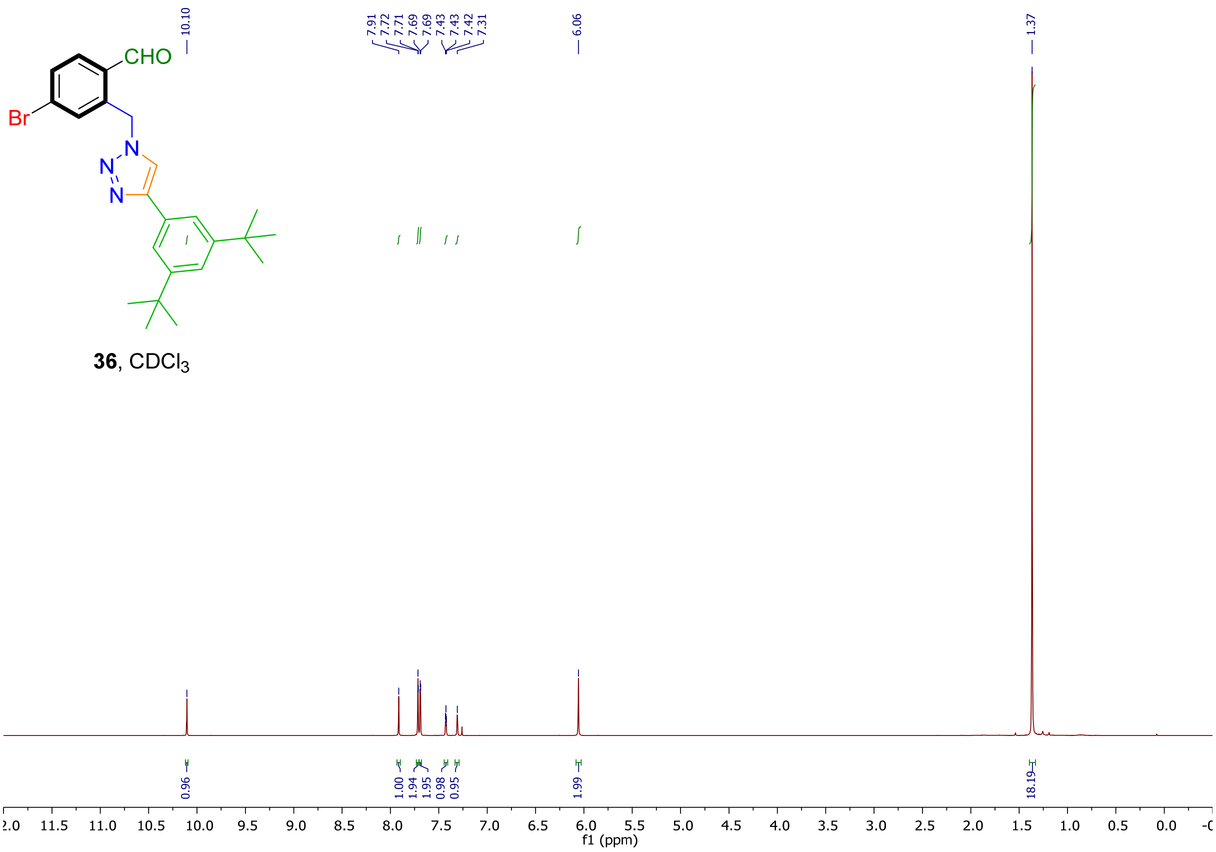

36, $CDCl_3$

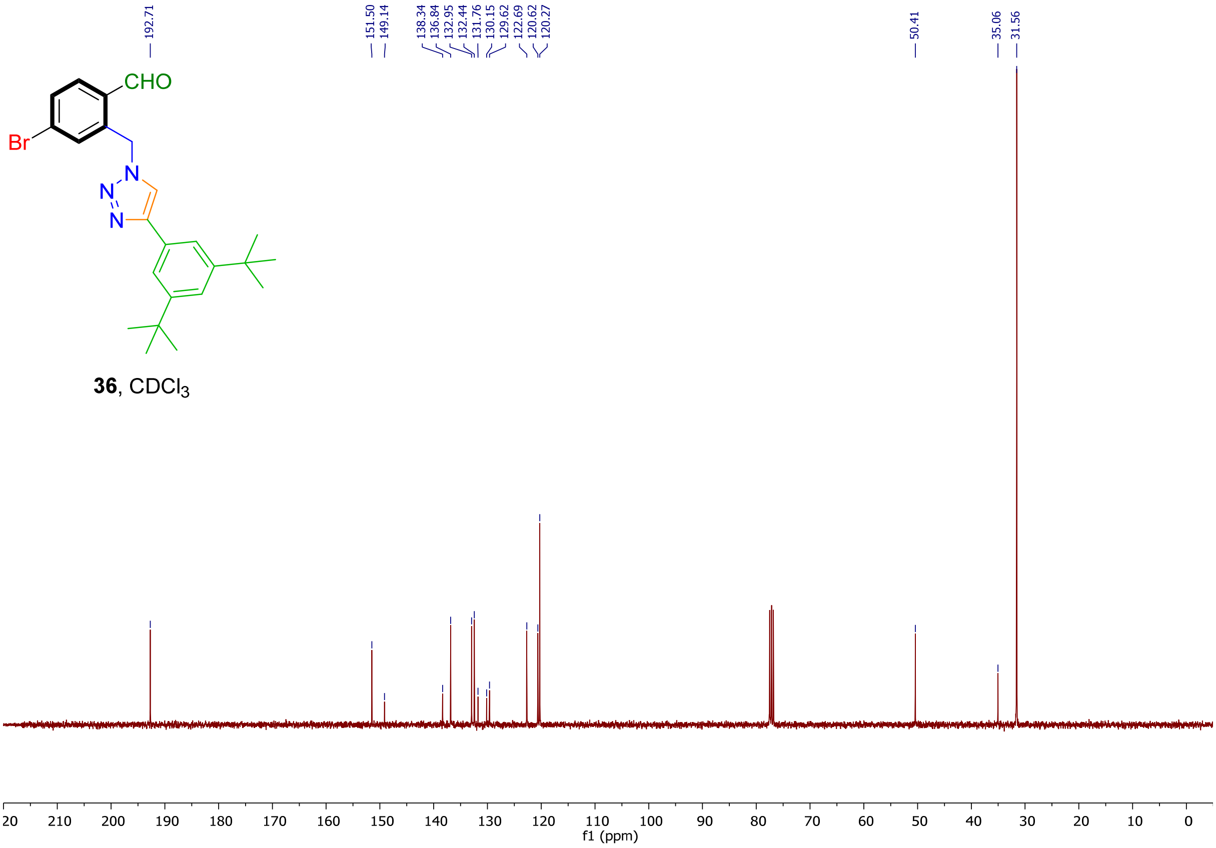

36, $CDCl_3$

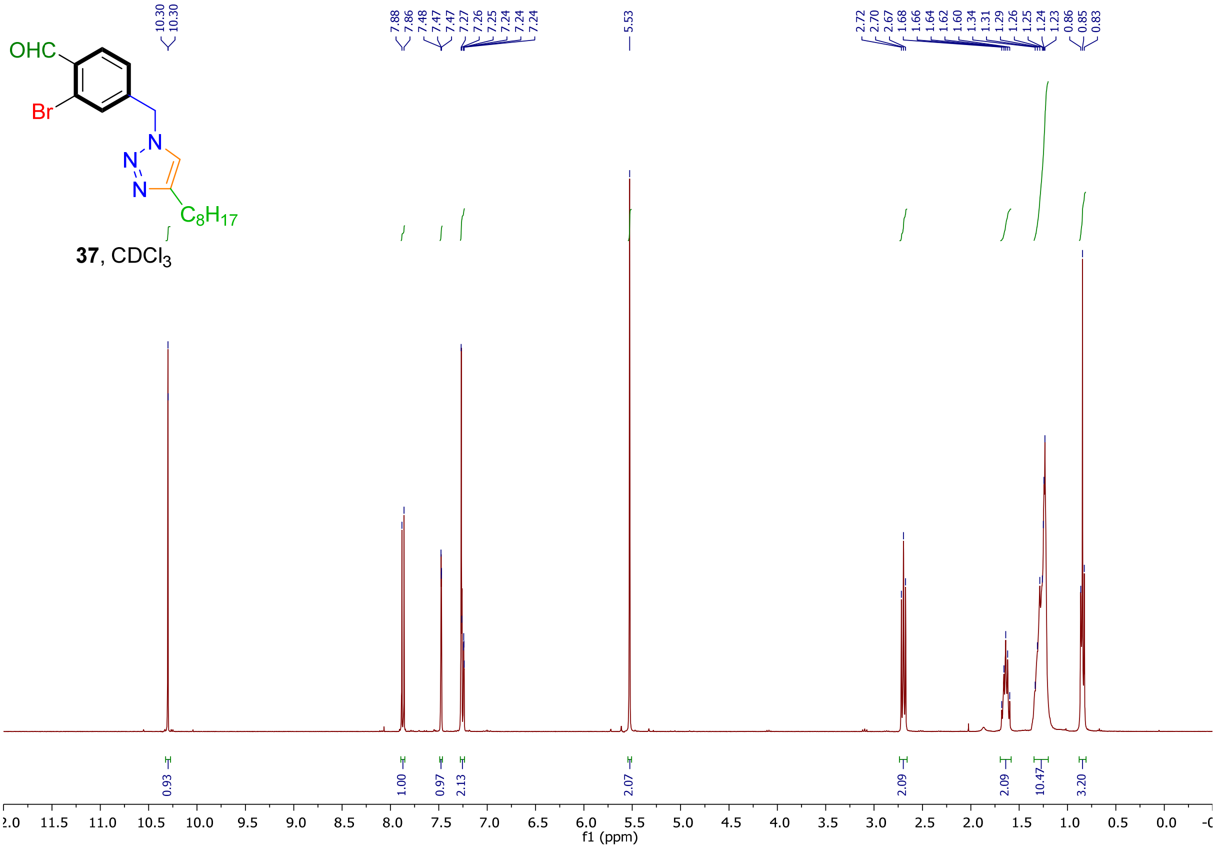
10.30
10.30
7.88
7.86
7.48
7.47
7.47
7.27
7.26
7.25
7.24
7.24
7.24
5.53
2.72
2.70
2.67
1.68
1.66
1.64
1.62
1.60
1.34
1.31
1.29
1.26
1.25
1.24
1.23
0.86
0.85
0.83
OHC
Br
N
N
N
C8H17
37, CDCl3
0.93
1.00
0.97
2.13
2.07
2.09
2.09
10.47
3.20
f1 (ppm)

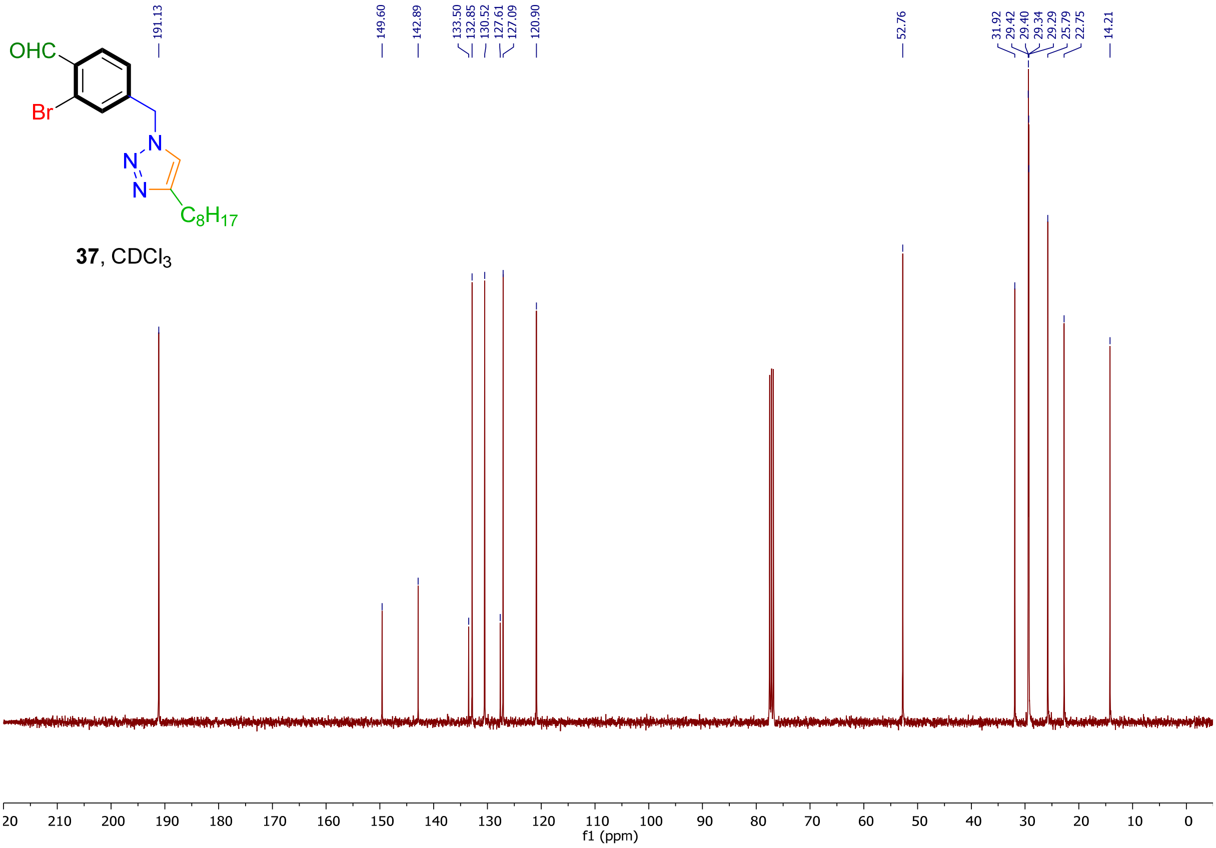
191.13
149.60
142.89
133.50
132.85
130.52
127.61
127.09
120.90
52.76
31.92
29.42
29.40
29.34
29.29
25.79
22.75
14.21
OHC
Br
N
N
N
C8H17
37, CDCl3
f1 (ppm)

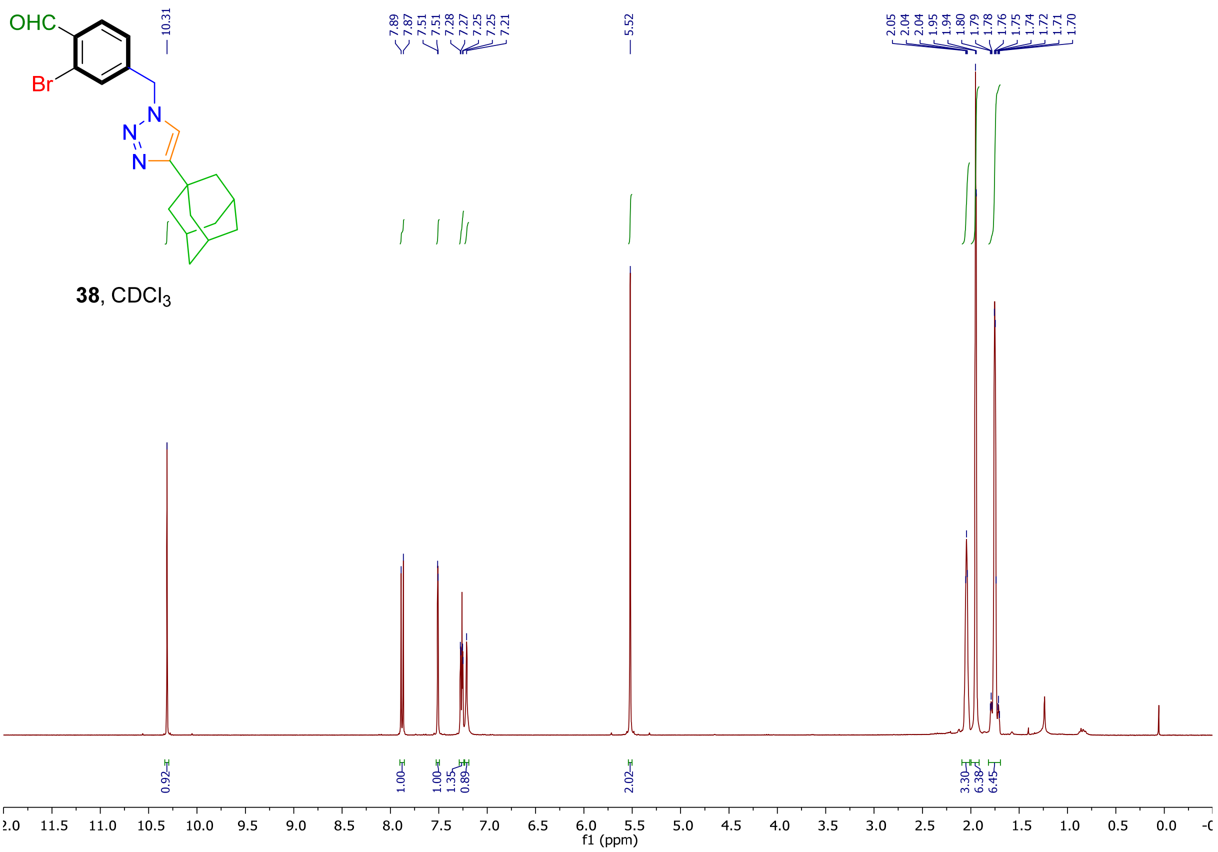
OHC
Br
N
N
N
38, CDCl3
10.31
7.89
7.87
7.51
7.51
7.28
7.27
7.25
7.25
7.21
5.52
2.05
2.04
2.04
1.95
1.94
1.80
1.79
1.78
1.76
1.75
1.74
1.72
1.71
1.70
0.92
1.00
1.00
1.35
0.89
2.02
3.30
6.38
6.45
f1 (ppm)

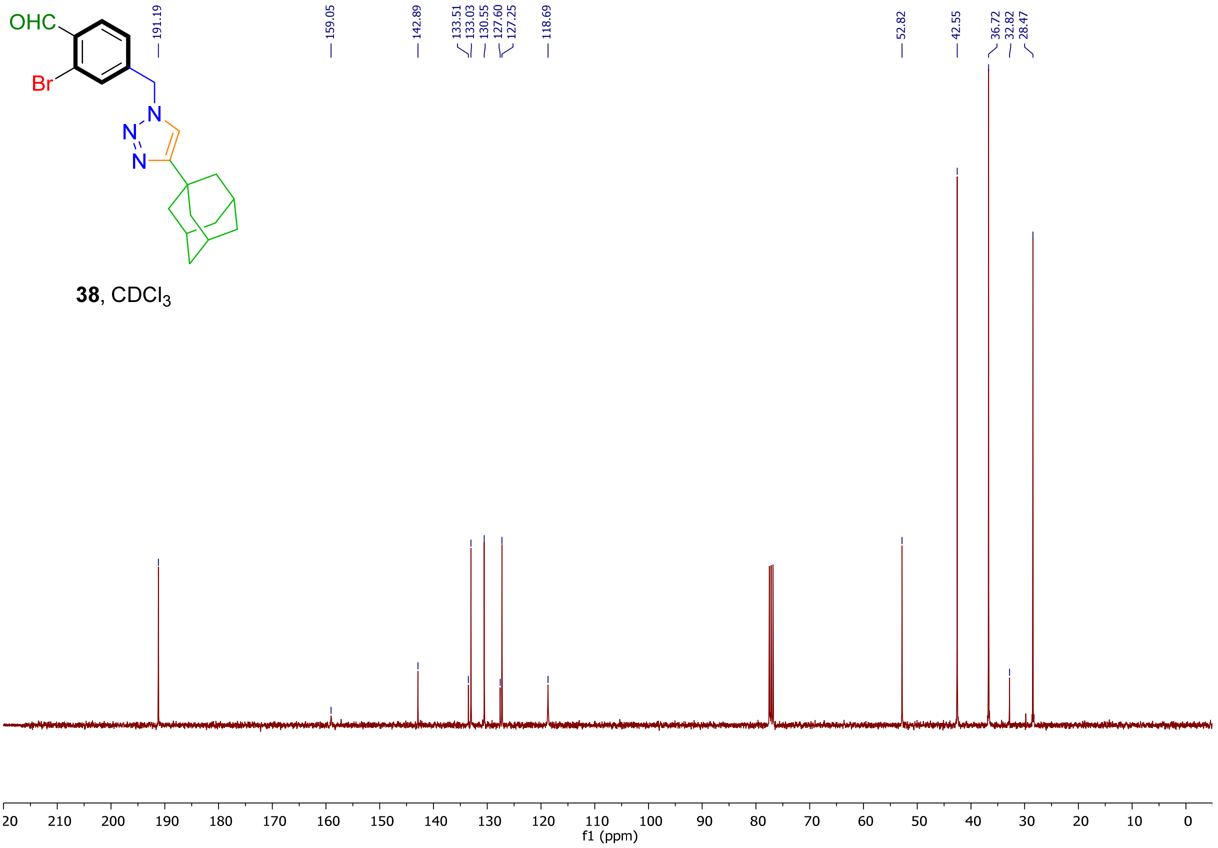
OHC
Br
N
N
N
38, CDCl3
191.19
159.05
142.89
133.51
133.03
130.55
127.60
127.25
118.69
52.82
42.55
36.72
32.82
28.47
f1 (ppm)

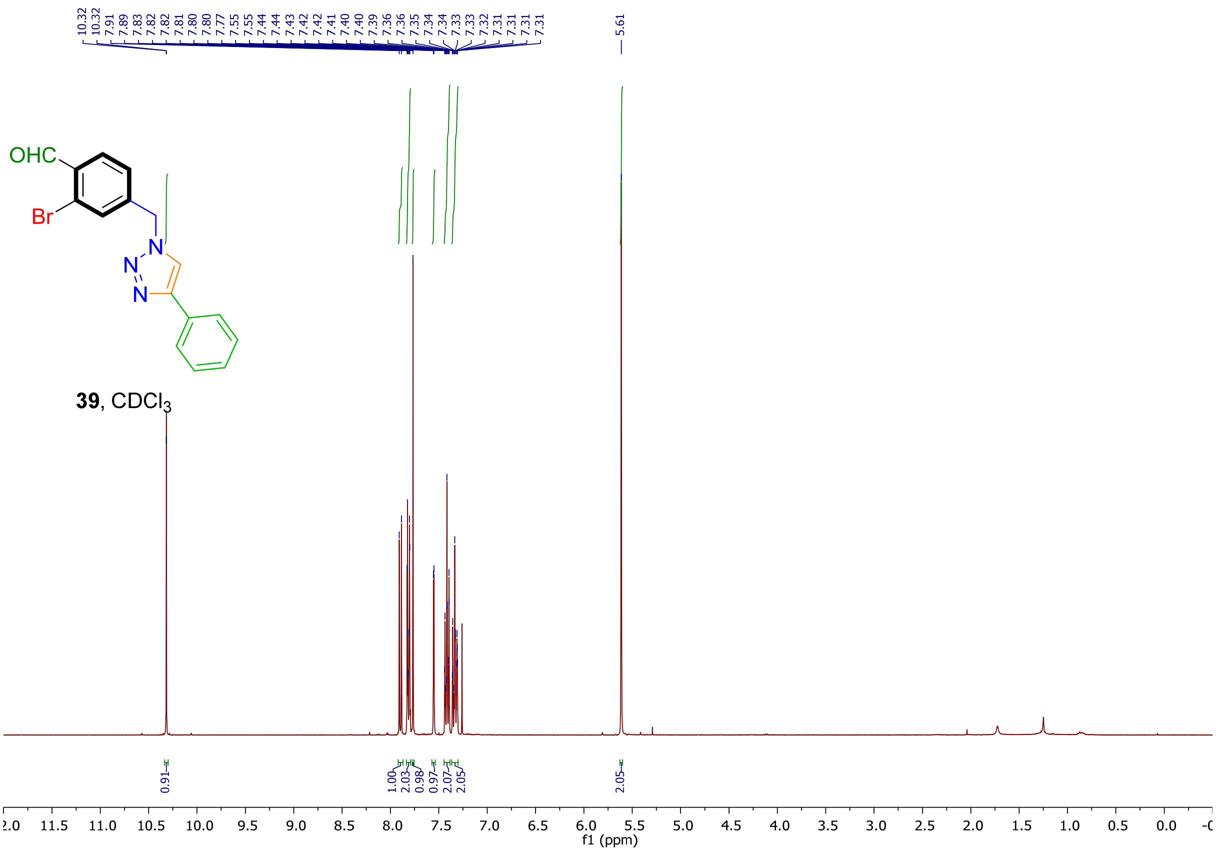

39, $CDCl_3$

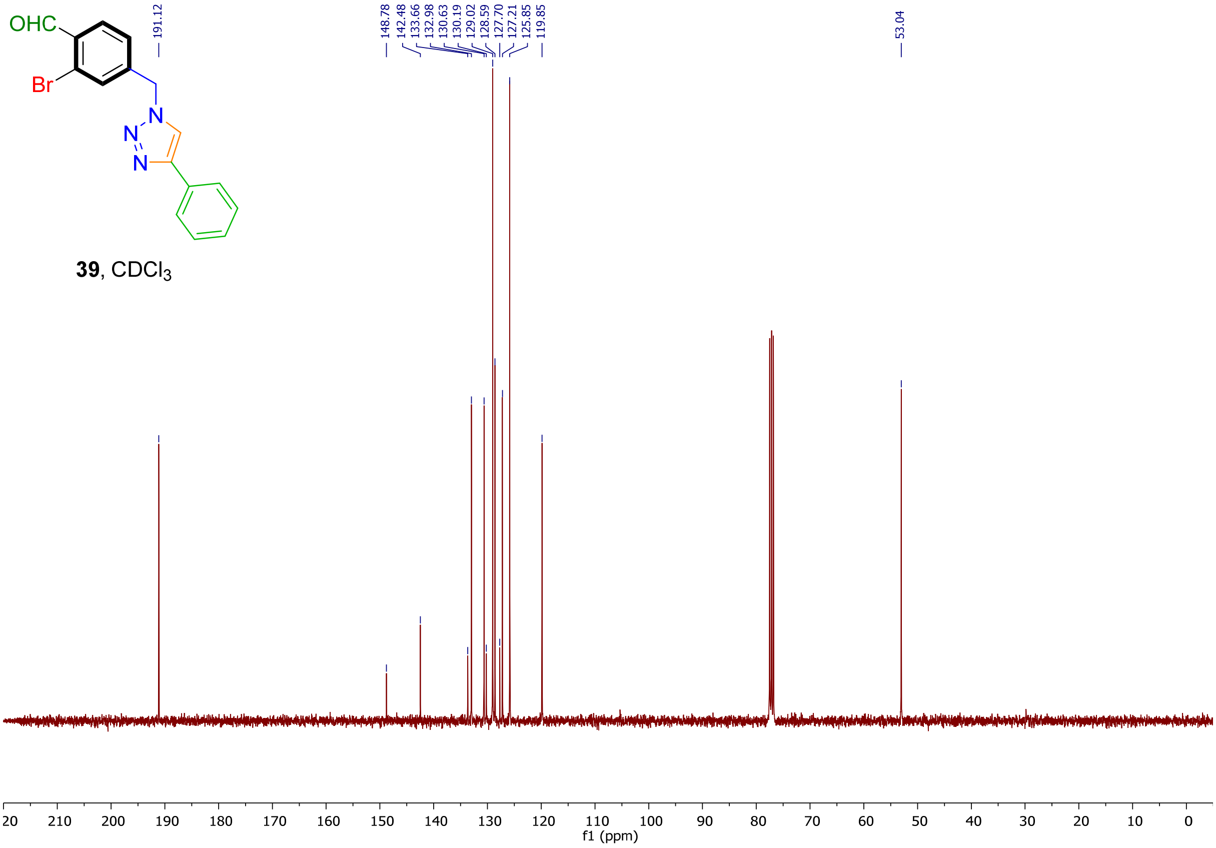

39, $CDCl_3$

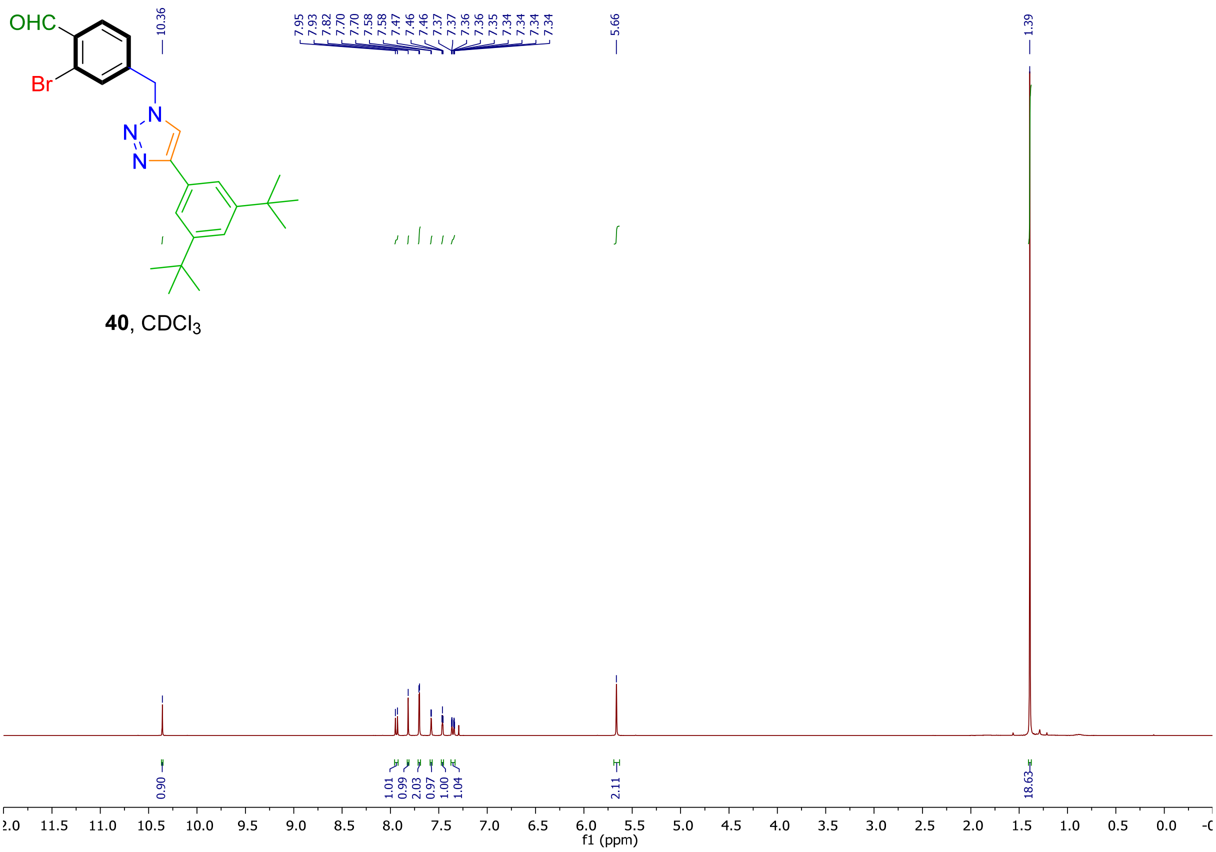

40, $CDCl_3$

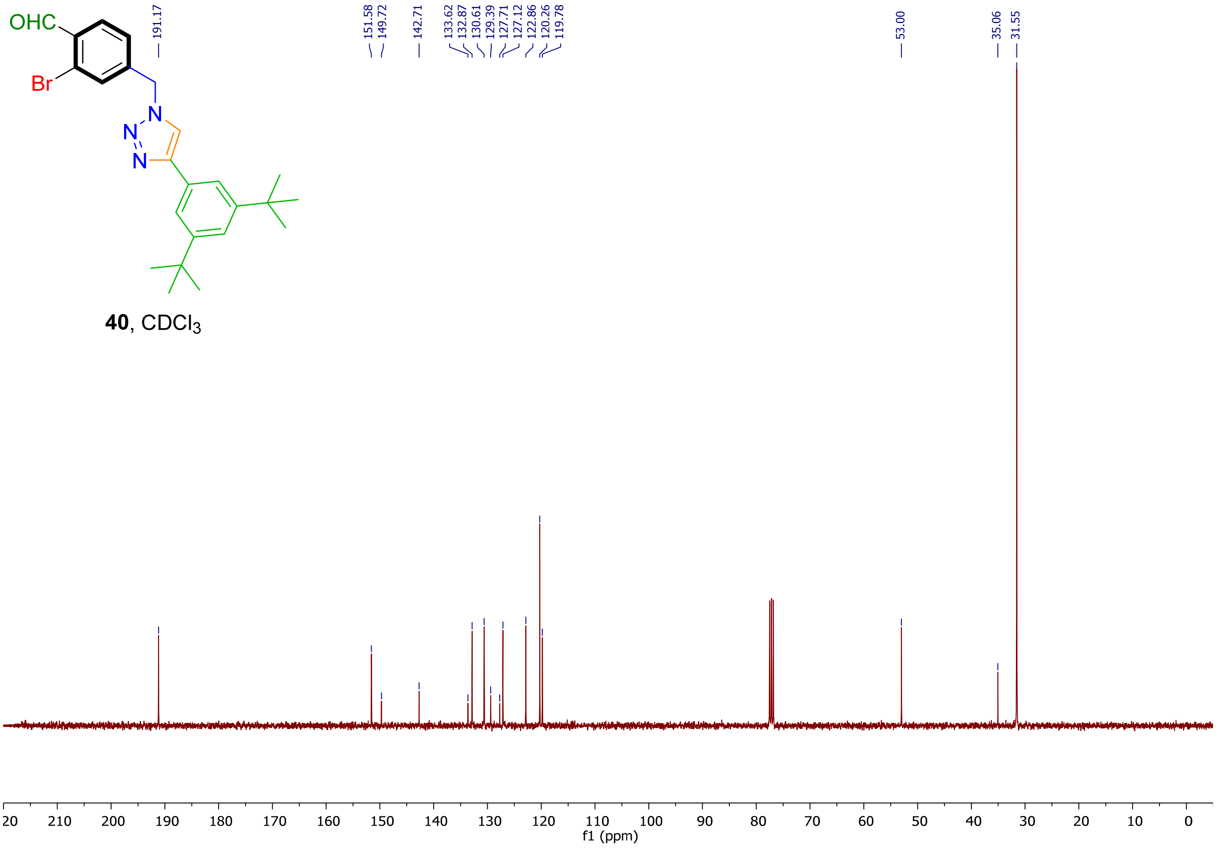

40, $CDCl_3$

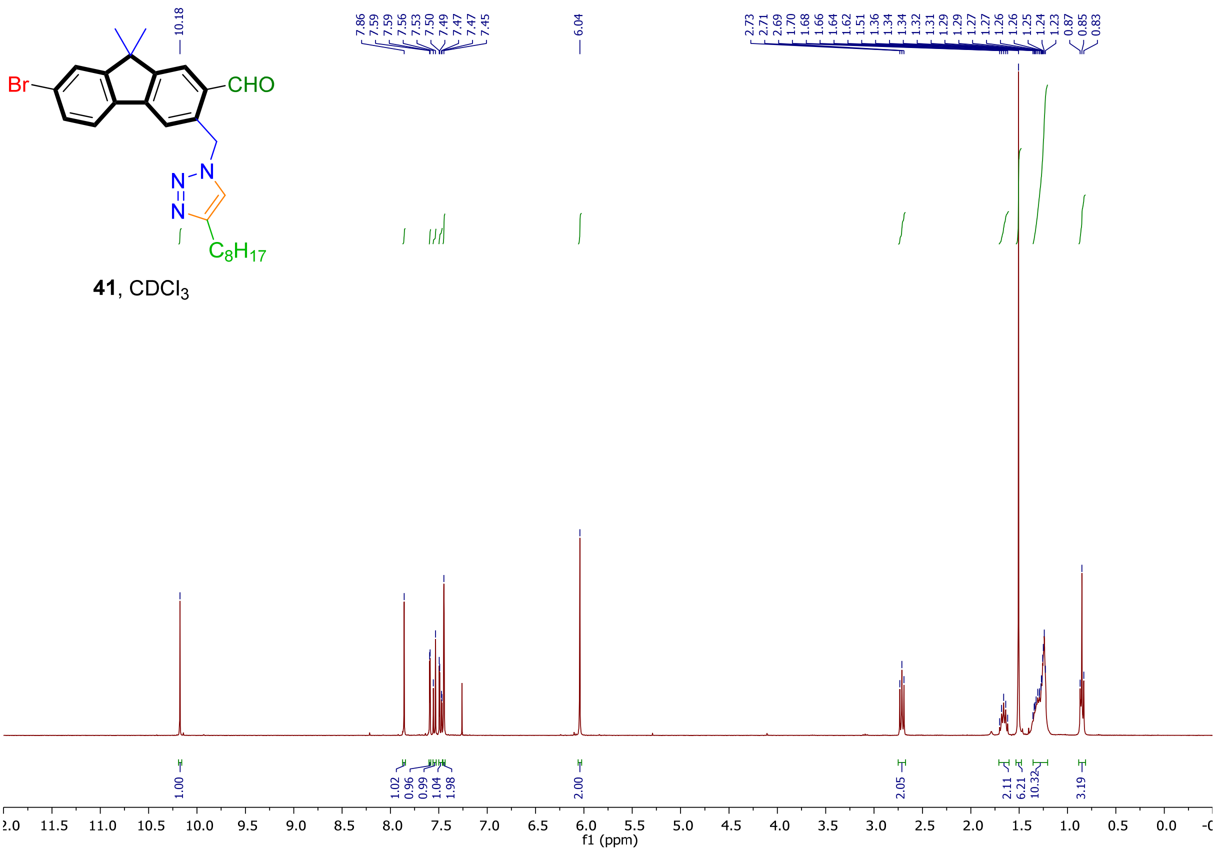
41, CDCl3
f1 (ppm)

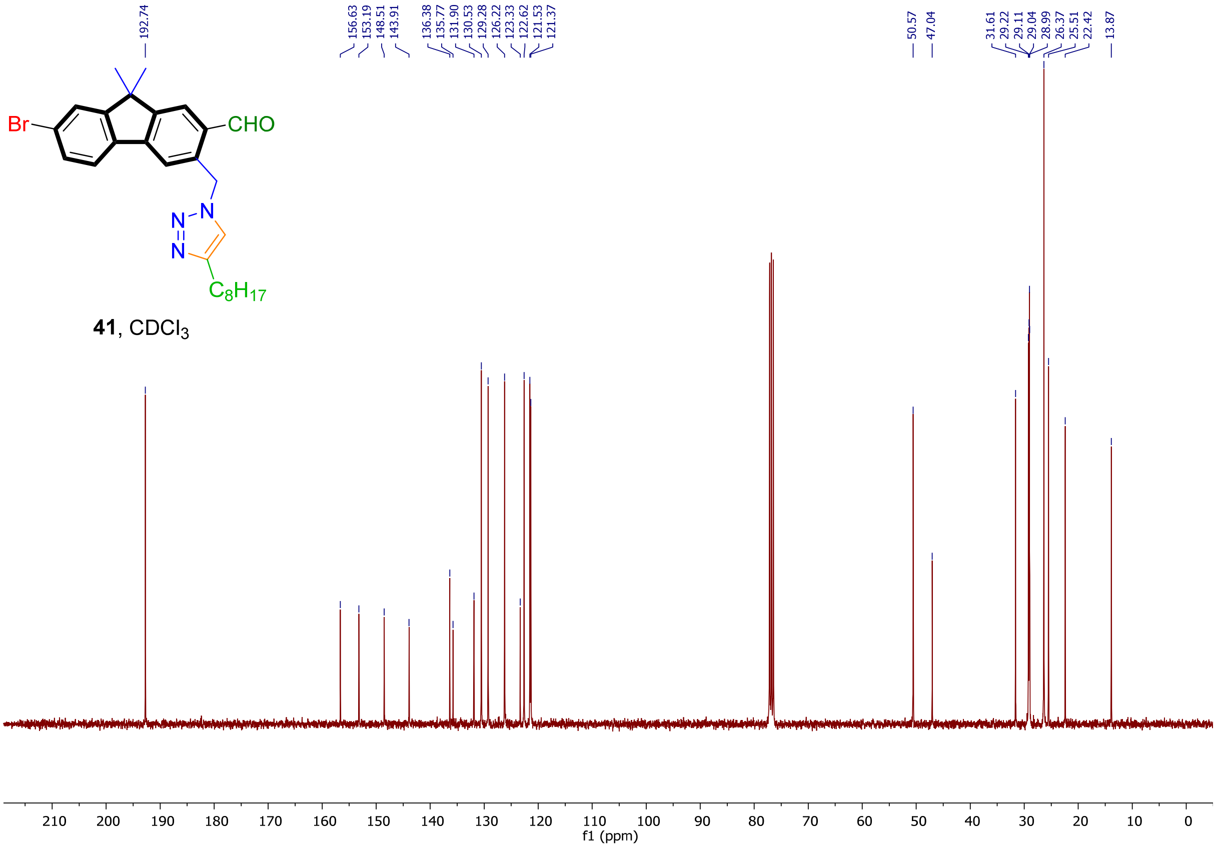
41, CDCl3
f1 (ppm)

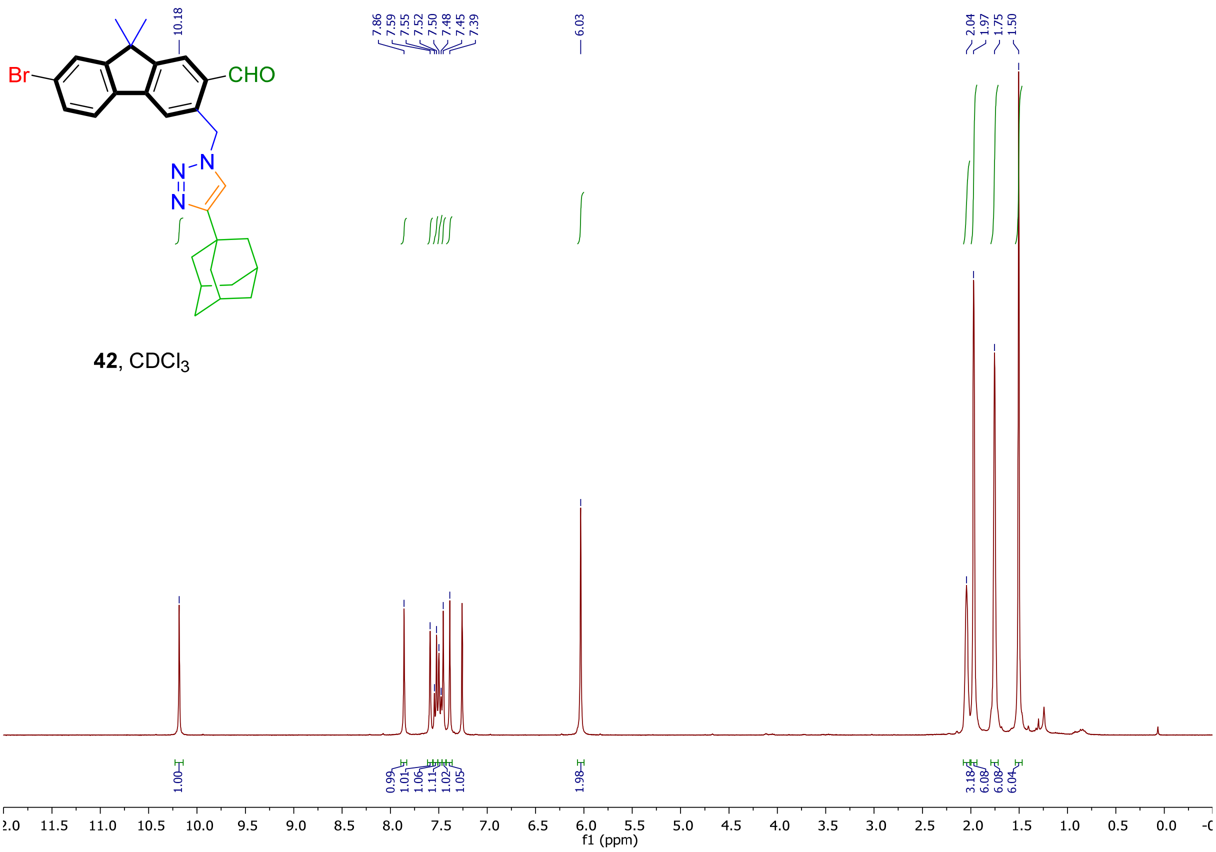

10.18
7.86
7.59
7.55
7.52
7.50
7.48
7.45
7.39
6.03
2.04
1.97
1.75
1.50
Br
CHO
42, CDCl3
1.00
0.99
1.01
1.06
1.11
1.02
1.05
1.98
3.18
6.08
6.08
6.04
f1 (ppm)

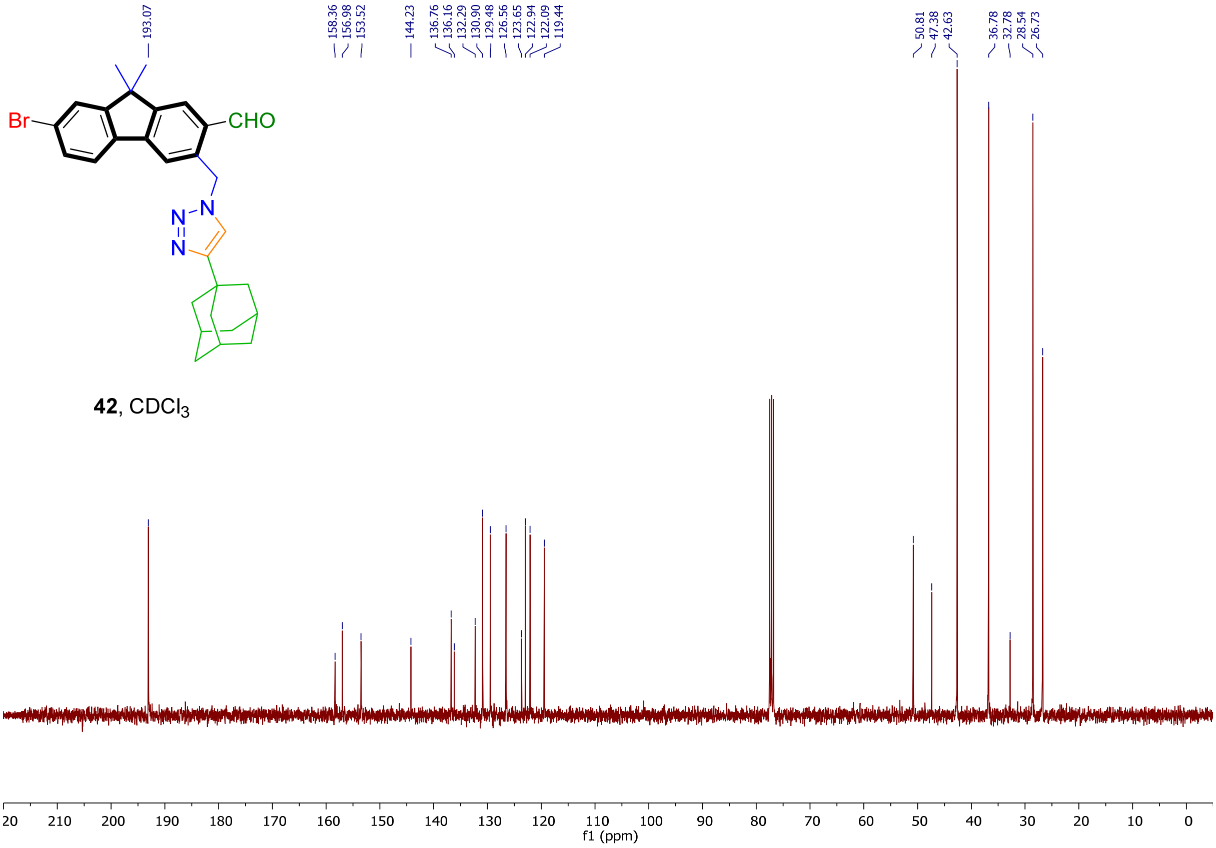

193.07
158.36
156.98
153.52
144.23
136.76
136.16
132.29
130.90
129.48
126.56
123.65
122.94
122.09
119.44
50.81
47.38
42.63
36.78
32.78
28.54
26.73
Br
CHO
42, CDCl3
f1 (ppm)

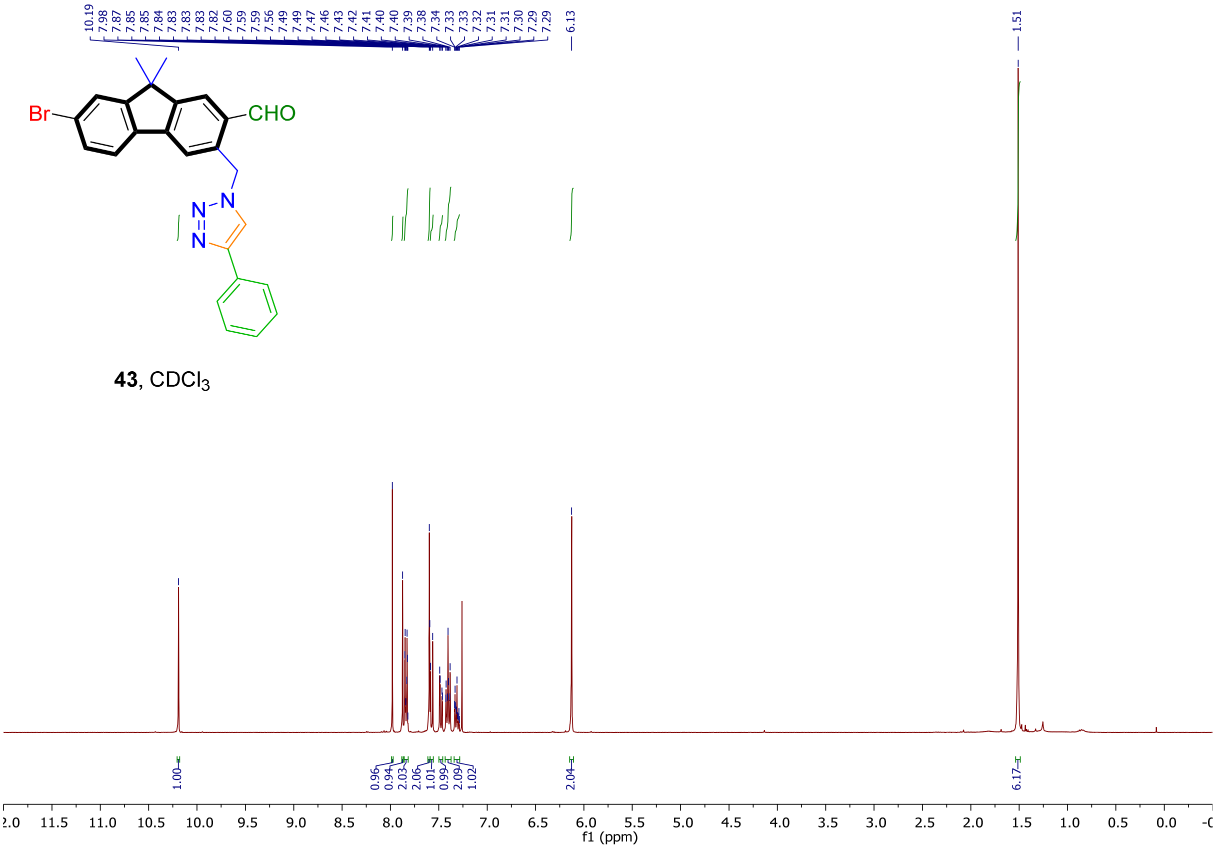
Br
CHO
N
N
N
43, CDCl$_3$
f1 (ppm)

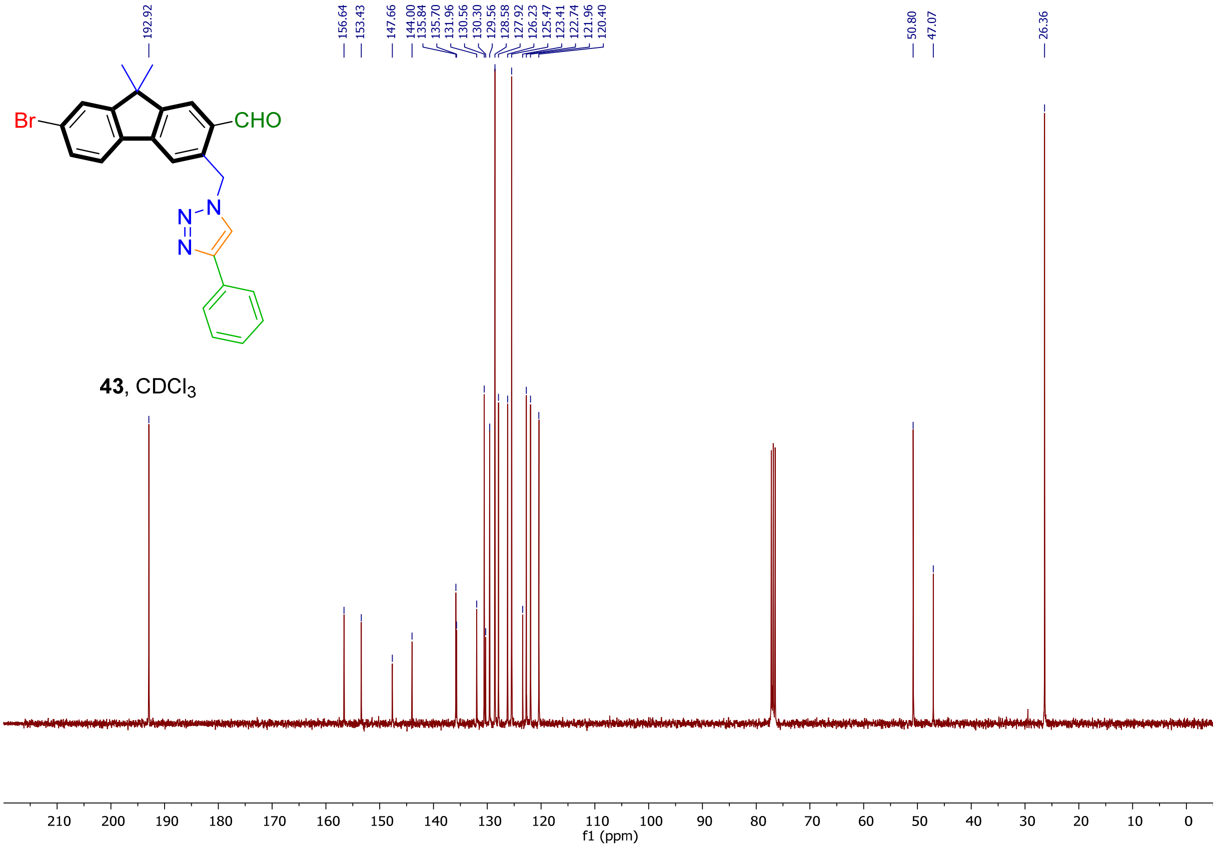
Br
CHO
N
N
N
43, CDCl$_3$
f1 (ppm)

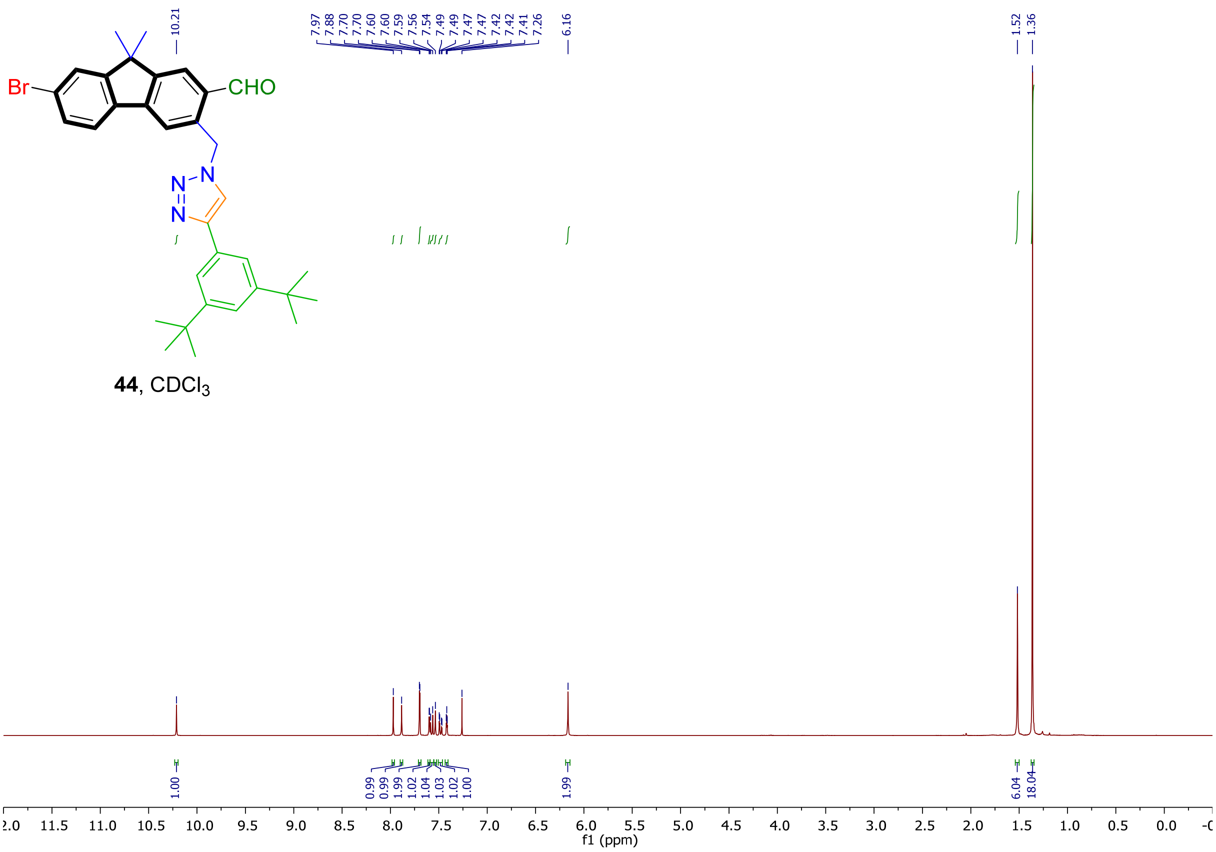
10.21
7.97
7.88
7.70
7.70
7.60
7.60
7.59
7.56
7.54
7.49
7.49
7.47
7.47
7.42
7.42
7.41
7.26
6.16
1.52
1.36
Br
CHO
N
N
N
44, CDCl3
1.00
0.99
0.99
1.99
1.02
1.04
1.03
1.02
1.00
1.99
6.04
18.04
f1 (ppm)

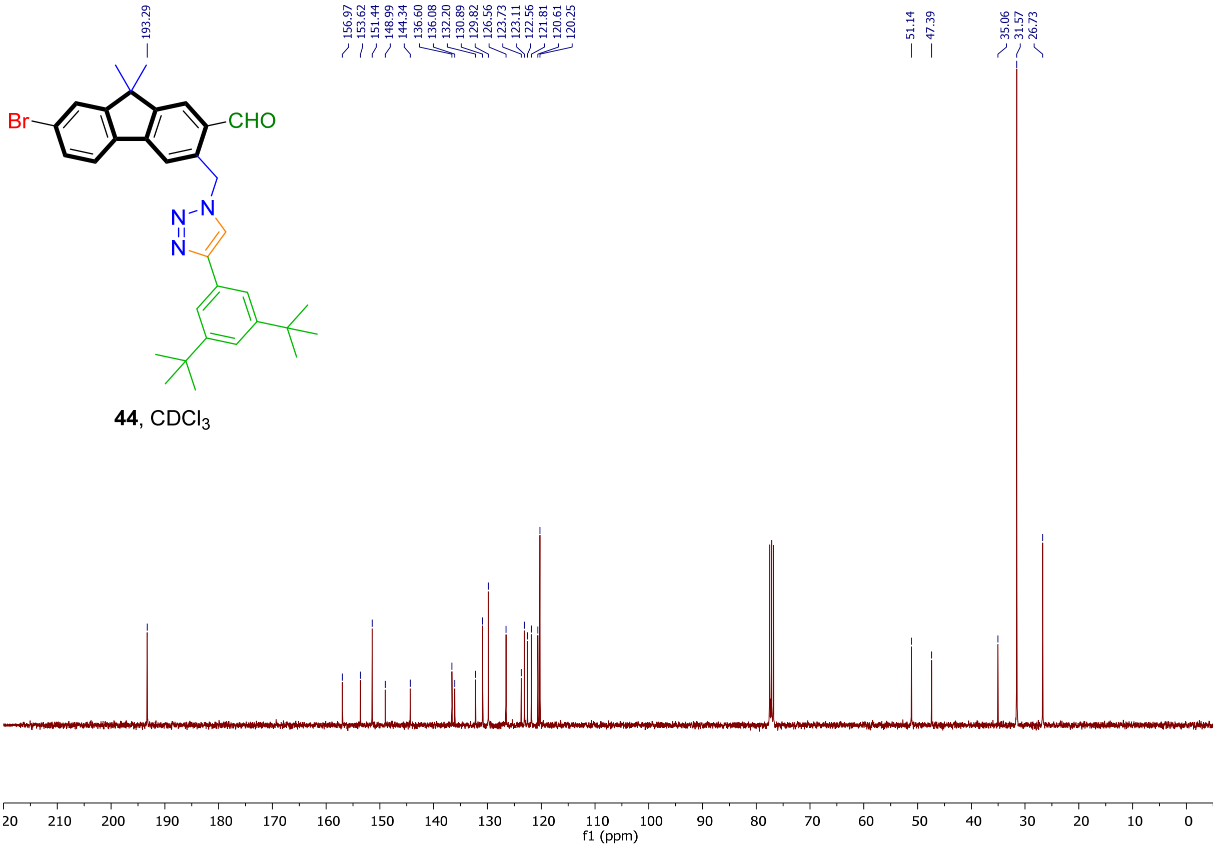
193.29
156.97
153.62
151.44
148.99
144.34
136.60
136.08
132.20
130.89
129.82
126.56
123.73
123.11
122.56
121.81
120.61
120.25
51.14
47.39
35.06
31.57
26.73
Br
CHO
N
N
N
44, CDCl3
f1 (ppm)

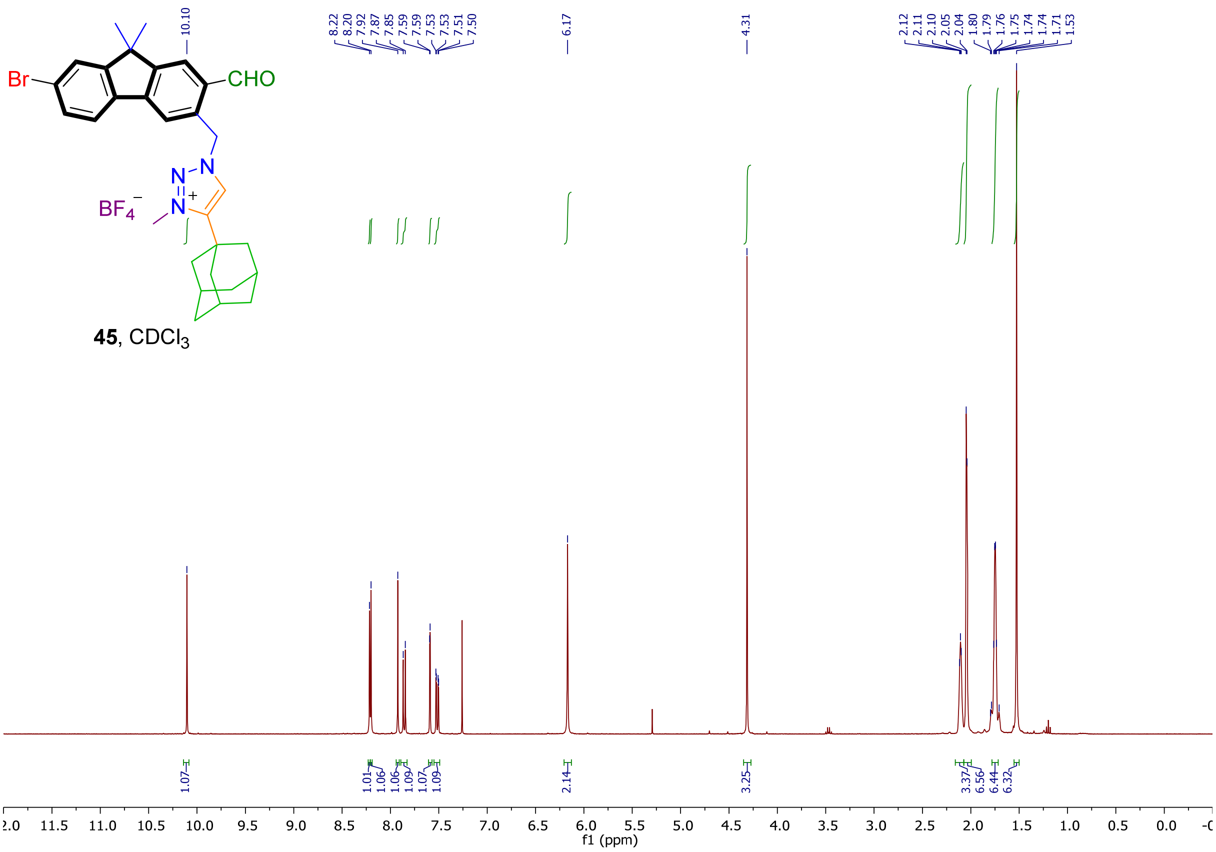

10.10
8.22
8.20
7.92
7.87
7.85
7.59
7.59
7.53
7.53
7.51
7.50
6.17
4.31
2.12
2.11
2.10
2.05
2.04
1.80
1.79
1.76
1.75
1.74
1.74
1.71
1.53
Br
CHO
BF4
45, CDCl3
1.07
1.01
1.06
1.06
1.09
1.07
1.09
2.14
3.25
3.37
6.56
6.44
6.32
f1 (ppm)

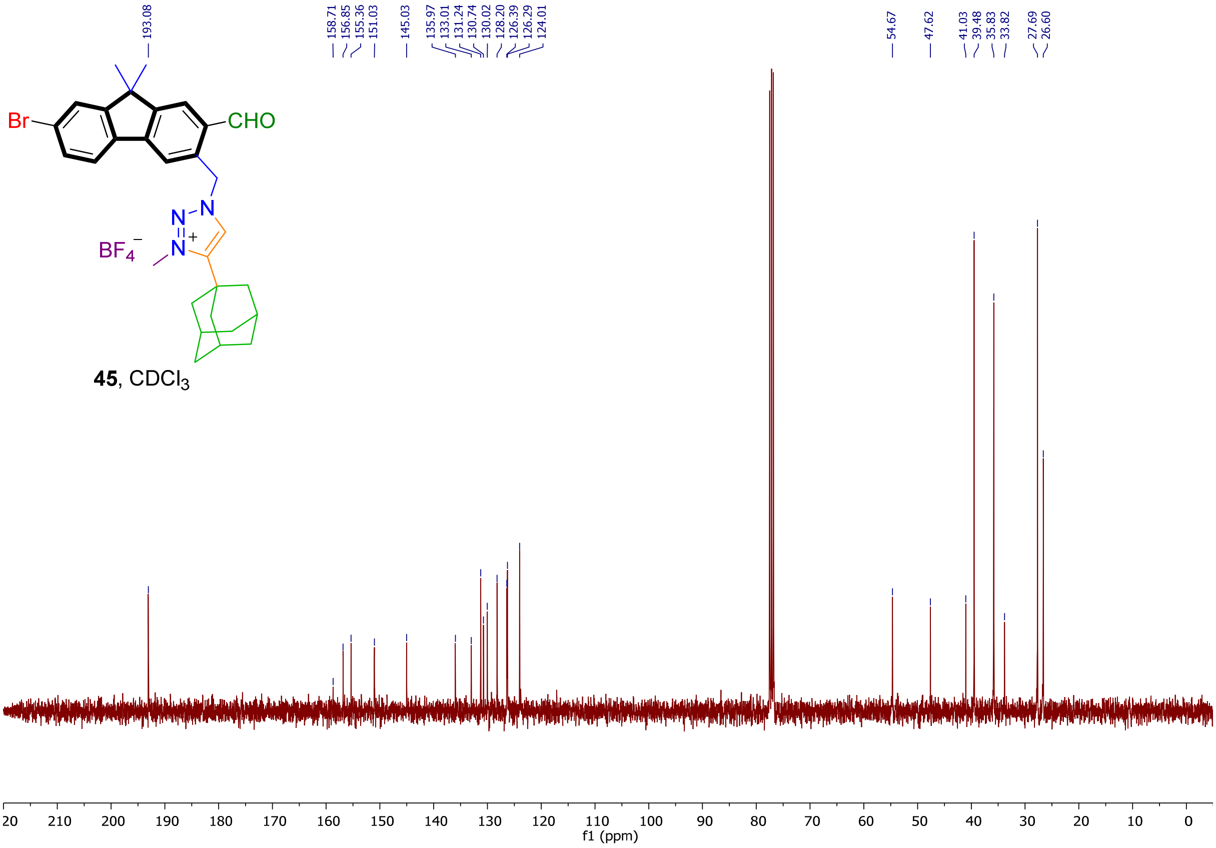

193.08
158.71
156.85
155.36
151.03
145.03
135.97
133.01
131.24
130.74
130.02
128.20
126.39
126.29
124.01
54.67
47.62
41.03
39.48
35.83
33.82
27.69
26.60
Br
CHO
BF4
45, CDCl3
f1 (ppm)
